# Biomimetic oxidative copolymerization of hydroxystilbenes and monolignols

**DOI:** 10.1126/sciadv.ade5519

**Published:** 2023-03-08

**Authors:** Hoon Kim, Jorge Rencoret, Thomas J. Elder, José C. del Río, John Ralph

**Affiliations:** ^1^ Department of Energy Great Lakes Bioenergy Research Center, Wisconsin Energy Institute, University of Wisconsin-Madison, Madison, WI 53726, USA.; ^2^ Instituto de Recursos Naturales y Agrobiología de Sevilla (IRNAS), CSIC, Avenida de la Reina Mercedes, 10, 41012, Seville, Spain.; ^3^ USDA-Forest Service, Southern Research Station 521 Devall Dr. Auburn, AL 36849, USA.; ^4^ Department of Biochemistry, University of Wisconsin-Madison, Madison, WI 53706, USA.

## Abstract

Hydroxystilbenes are a class of polyphenolic compounds that behave as lignin monomers participating in radical coupling reactions during the lignification. Here, we report the synthesis and characterization of various artificial copolymers of monolignols and hydroxystilbenes, as well as low-molecular-mass compounds, to obtain the mechanistic insights into their incorporation into the lignin polymer. Integrating the hydroxystilbenes, resveratrol and piceatannol, into monolignol polymerization in vitro, using horseradish peroxidase to generate phenolic radicals, produced synthetic lignins [dehydrogenation polymers (DHPs)]. Copolymerization of hydroxystilbenes with monolignols, especially sinapyl alcohol, by in vitro peroxidases notably improved the reactivity of monolignols and resulted in substantial yields of synthetic lignin polymers. The resulting DHPs were analyzed using two-dimensional NMR and 19 synthesized model compounds to confirm the presence of hydroxystilbene structures in the lignin polymer. The cross-coupled DHPs confirmed both resveratrol and piceatannol as authentic monomers participating in the oxidative radical coupling reactions during polymerization.

## Teaser

Hydroxystilbene monomers participate in radical coupling reactions during the lignification as non-canonical lignin monomers.

## INTRODUCTION

**Fig. 1.**
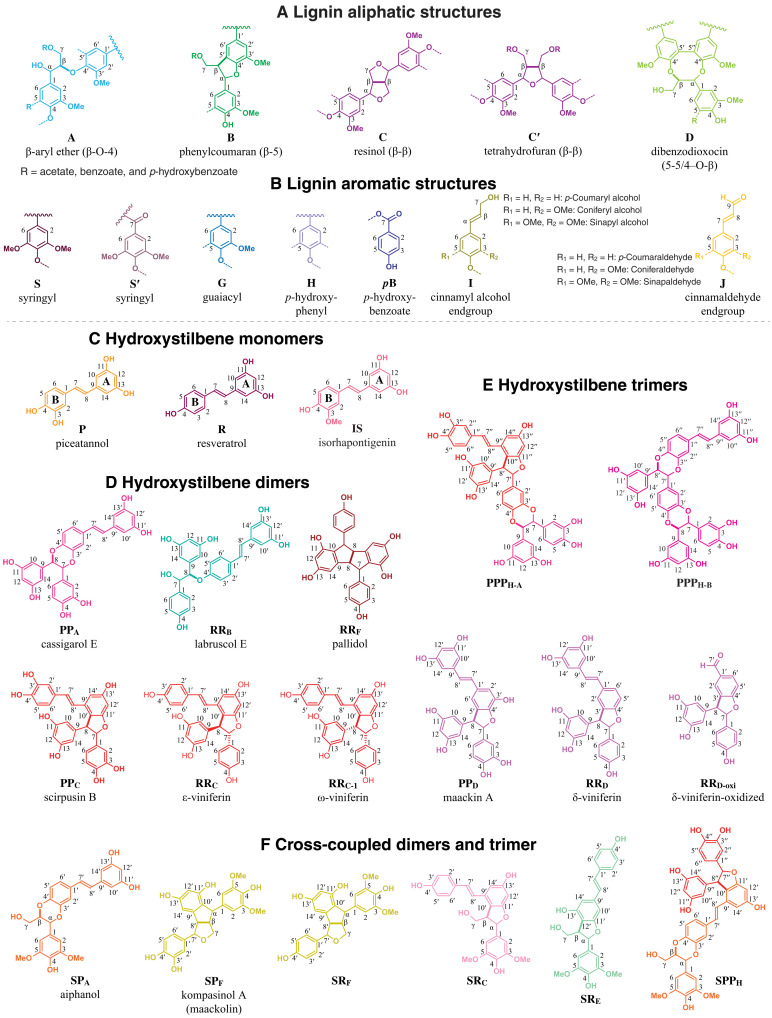
Lignin and hydroxystilbene structures. (**A**) Conventional aliphatic structures of lignin units **A** to **D** from the various combinatorial coupling modes; the bonds formed in the radical coupling step are bolded. Some of the lignin units may be acylated at the γ-OH (R = acetate, benzoate, and *p*-hydroxybenzoate). (**B**) Lignin aromatic units are characterized by their methoxy substitution on the aromatic ring as **H**, **G**, and **S**. (**C**) The three most common hydroxystilbenes (piceatannol **P**, resveratrol **R**, and isorhapontigenin **IS**). (**D** and **E**) Hydroxystilbene dimers and trimers were synthesized under oxidative conditions, separated, and characterized. (**F**) Cross-coupling reaction between hydroxystilbenes and sinapyl alcohol produced various hybrid structures.

Lignin is one of the essential natural polymers in terrestrial plants and represents roughly 30% of the annual carbon sequestration in plant materials (*1*, *2*). It is considered to be the most abundant renewable phenolic biomaterial and has the potential to replace current fossil-derived petrochemicals. Traditionally, lignin has been envisioned to be made up of three major structural units, guaiacyl **G**, syringyl **S**, and *p*-hydroxyphenyl **H**, that result from the oxidative radical coupling of the three building blocks, the monolignols coniferyl, sinapyl, and *p*-coumaryl alcohols, respectively (Fig. 1) (*1*, *3*, *4*).

General lignin structure/composition differs greatly depending on the plant species, tissue, stage of development, and environmental conditions (*5*, *6*). One of the main contributions to such structural variation is the plasticity of the lignification process that allows plants to increase the relative abundance of trace subunits or to add noncanonical components to lignin in many cases (*7*). To date, more than 35 different phenolic compounds have been found to participate in radical coupling reactions during lignification (*8*–*10*). These compounds are found in many plant species as the result of natural or induced mutations (*11*) or genetically engineered modifications (*12*). The search for potential lignin precursors and the characterization of the resulting chemical structures in the polymer are crucial steps in understanding the properties of lignins.

**Fig. 2.**
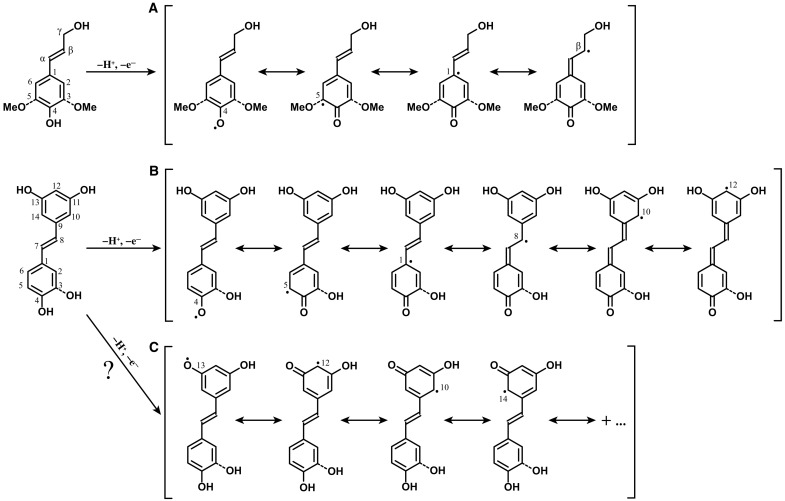
Monolignol and hydroxystilbene radical formation and the resonance structures. (**A**) One-electron oxidation of 4-hydroxycinnamyl alcohols produces the phenolic radical. The resonance forms help us to understand how the combinatorial coupling products are produced in the radical coupling reactions. Coupling occurs at the β-, 4–O-, and 5-positions of *p*-coumaryl and coniferyl alcohols, but sinapyl alcohol couples only at its β- and 4–O- positions as it has a methoxy group at the 5-position. (**B**) Hydroxystilbenes can, similarly to monolignols, each generate the phenolic radical, and the resonance forms showed how coupling can occur at the 4–O-, 5-, 8-, 10-, and 12-positions. (**C**) The resorcinol moiety may theoretically produce a radical from the 11- or 13-OH, but the single-electron density cannot locate, by resonance, to the other moiety. We do not evidence coupling from these phenolic radicals and contend that the 12-, 10/14-coupled products arise from the phenolic radical in B.

One of the most recent and notable examples of newly found lignin components is hydroxystilbenes (or stilbenoids) in palm fruit endocarp lignins (*13*). Hydroxystilbenes occur within a limited range of plant species because the key enzyme, stilbene synthase, does not universally exist (*14*). Hydroxystilbenes are synthesized in a manner similar to flavonoids, and both share a common intermediate, *p*-coumaroyl-CoA (*p*CA-CoA), with monolignol biosynthesis (*15*, *16*). Hydroxystilbenes are widely known as phytoalexins due to their antioxidant properties and their roles in plant protection against pathogens (*17*–*19*). There are more than 1000 hydroxystilbenes that have been isolated and identified (*20*). The most widely known hydroxystilbene in the popular press is resveratrol, which exists in grapes, berries, and several medicinal plant species (*16*, *21*, *22*). It exhibits antioxidant, antiallergic, and antiaging activity and displays neuroprotective effects (*23*). Piceatannol is generally found in berries, grapes, rhubarb, passionfruit, white tea, and Japanese knotweed and is also known as a powerful antioxidant (*19*, *24*, *25*). Isorhapontigenin also occurs in grapes and some Asian medicinal plant species (e.g., *Gnetum cleistostachyum*) (*26*, *27*). Hydroxystilbenes exist not only as monomers but also as dimers and oligomers. The monomers can be oxidized to form radicals that, like the monolignols, can dehydrodimerize or cross-couple with different hydroxystilbenes to produce various dimers, trimers, and oligomers (Fig. 2 and fig. S1) (*13*, *28*–*32*). It is also known that the hydroxystilbenes react with monolignols in radical cross-coupling reactions to produce hybrid lignans, stilbenolignans, that have two phenylpropanoid units connected together through diverse linkages; they have been found in a wide range of plants from different families and, as lignans, are presumably optically active (fig. S1) (*33*–*36*). All of these examples of stilbenolignans demonstrate the proclivity of hydroxystilbene radicals to undergo radical cross-coupling reactions and suggest that they could also participate in lignification, the process producing the plant cell wall polymer lignin from phenolic monomers. Although only recently discovered, it is therefore not unexpected to have found hydroxystilbenes in the lignins of macaúba (*Acrocomia aculeata*), carnauba (*Copernicia prunifera*), and coconut (*Cocos nucifera*) palm fruit endocarps (*13*, *28*). Hydroxystilbene glucosides (piceid, isorhapontin, and astringin) were also found in the lignin of Norway spruce bark (*37*, *38*).

The main purposes of this research were to elucidate the detailed structures of the newly found hydroxystilbenes in lignins and to investigate their cross-coupling products generated by oxidative radical reactions during lignification. We initially reported the lignin structures of palm fruits endocarp from the research primarily focused on the modest verification of the newly discovered lignin structures with limited structural validation using crude dimerization reactions (*13*), but the contentions admittedly fell short of robust structural elucidation supporting the incorporation of hydroxystilbenes into lignin polymers by cross-coupling. It was therefore essential to provide a more extensive investigation into the details by a systematic approach to support (or refute) the oxidative radical coupling mechanism and the enhanced claims of plasticity in lignification. Here, we first prepared dehydrogenation polymers (DHPs) by slowly adding the hydroxystilbene monomers and monolignols to solutions containing horseradish peroxidase and H_2_O_2_. The DHP studies support the argument that certain monolignol substitutes can be efficiently incorporated into lignins and may result in changes in cell wall properties. The DHPs were examined using gel permeation chromatography (GPC), derivatization followed by reductive cleavage (DFRC) analysis, and nuclear magnetic resonance (NMR) spectroscopy to establish that hydroxystilbenes are incorporated into the synthetic lignin polymers. The NMR data from the DHPs and the lignins from different palm fruit endocarps were then compared side-by-side for structural verification in the latter. We also prepared one-pot cross-coupling reactions between hydroxystilbenes and monolignols to isolate dimeric and trimeric compounds from the crude mixtures of low–molecular weight (MW) materials. Prepared model compounds that would assist the elucidation of the hydroxystilbene structures in the lignins are suitable for supporting a mechanistic study of stilbenolignan production. In addition, density functional theory (DFT) calculations were performed to evaluate the energetics of quinone methide formation and the final rearomatization step, and the results were compared to the observations of the structures produced in short time cross-coupling reactions.

## RESULTS AND DISCUSSION

### Biomimetic preparation of low–molecular weight model compounds

In the current study, we synthesized hydroxystilbene monomers and various piceatannol and resveratrol dimers (Fig. 1, C to F) using different radical reaction conditions including horseradish peroxidase and H_2_O_2_ and inorganic oxidants, MnO_2_, FeCl_3_, Ag_2_O, and AgOAc, in different organic solvent systems. The synthetic details for the collected low–molecular weight compounds, their structural elucidation, and radical coupling mechanisms for their formation are discussed in the Supplementary Materials, and the yields of the collected dimers and trimers are presented in Table 1. The product yields depend on the reaction conditions; details are in the Supplementary Materials. Most of these compounds have been previously isolated as natural compounds from various plants, but some of them were prepared in vitro in this study for the first time.

**Table 1.** The yield of collected dimers and trimers from radical reactions in various conditions.

Starting materials	Piceatannol P (300 mg)	Resveratrol R (227 mg)	Piceatannol P (300 mg) + sinapyl alcohol S (258 mg)	Resveratrol R (300 mg) + sinapyl alcohol S (276 mg)
A. Dimers	**PP_A_** (43 mg, 21.5%)		**SP_A_** (5.6 mg, 1.0%)	
		**RR_B_** (6.2 mg, 2.7%)		
	**PP_C_** (10.3 mg, 5.8%)	**RR_C_** (47.4 mg, 24.1%)		**SR_C_** (12.3 mg, 2.1%)
		**RR_C-1_** (8.4 mg, 4.3%)		
	**PP_D_** (16 mg, 8.0%)	**RR_D_** (96.3 mg, 42.4%)		
		**RR_D-oxi_** (2.4 mg, 1.1%)		
				**SR_E_** (6.1 mg, 1.1%)
		**RR_F_** (7.5 mg, 3.8%)	**SP_F_** (4.2 mg, 0.8%)	
B. Trimers	**PPP_H-A_** (22.4 mg, 11.2%)		**SPP_H_** (7.3 mg, 1.3%)	
	**PPP_H-B_** (13.9 mg, 9.3%)			

### Biomimetic preparation of piceatannol trimers

**Fig. 3.**
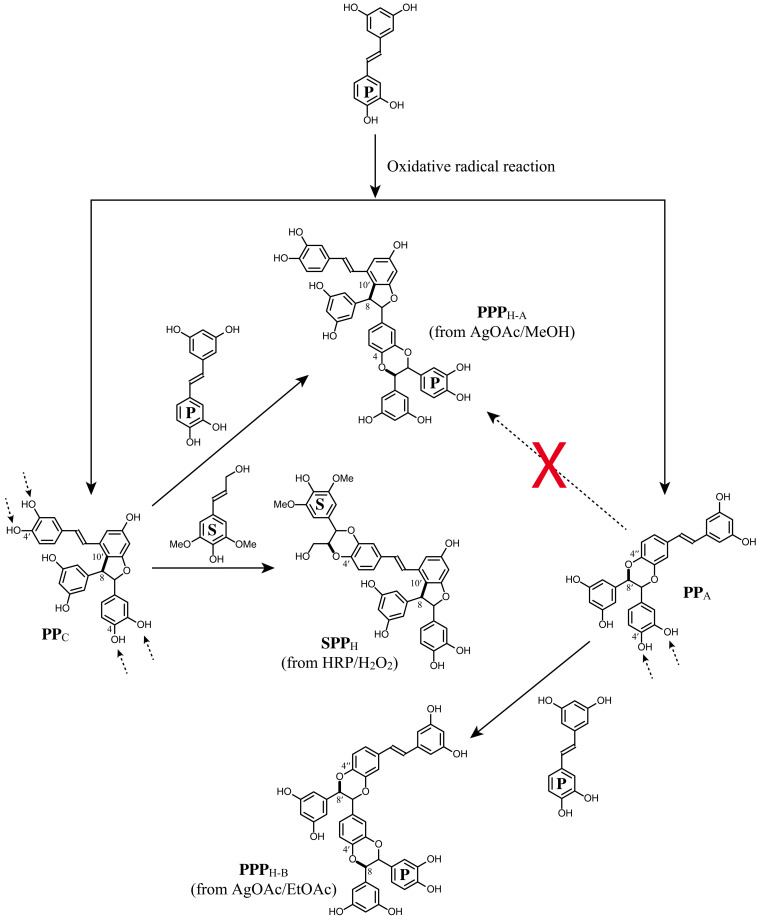
Formation of two piceatannol trimers (PPP_H-A_ and PPP_H-B_) and a cross-coupled trimer (SPP_H_) between piceatannol P and sinapyl alcohol S.

Unlike other reaction conditions that made mostly dimers as the major products, the AgOAc reaction produced trimers and dimers, but the outcome differed depending on the solvent system. When MeOH was used for the AgOAc reaction, trimers can arise from **PP**_**C**_, which has two available catechyl units to add a piceatannol and form benzodioxane structures. One product is **PPP**_**H-A**_ (Fig. 3) that logically arises from coupling of **PP**_**C**_ at its 4–O-position with another piceatannol at its 8-position. The NMR data from this trimer showed both the characteristic peaks of benzodioxane and phenylcoumaran structures from **PP**_**A**_ and **PP**_**C**_. Similarly, **PP**_**A**_ can 4–O-couple with another piceatannol (at its 8-position) to produce trimer **PPP**_**H-B**_. This occurred with the AgOAc reaction in EtOAc. The ^1^H–^13^C correlation peaks in the two-dimensional (2D) heteronuclear single-quantum coherence (HSQC) NMR data from the two pairs of 7 and 8 positions from the two benzodioxane moieties were close together in the neighboring area of the spectra but were nevertheless well resolved (table S2).

### Biomimetic preparation of cross-coupled dimers and a trimer of hydroxystilbenes and sinapyl alcohol

Similarly to the dehydrogenation of monolignols that generates the phenolic radicals, hydroxystilbene monomers can also be oxidized by peroxidases to form resonance-stabilized radicals, with single-electron density distributed around the aromatic ring and at the 8-position, as readily seen by drawing resonance structures (Fig. 2 and fig. S1) (*10*, *39*, *40*). The oxidative radical coupling is not necessarily limited to the same phenolic species. Unlike lignans, which are generally defined as dimeric compounds from radical coupling of two monolignols, nonconventional lignans can be formed between monolignols and other phenolic metabolites, such as stilbenes, coumarins, and flavonoids (*33*). Stilbenolignans are hybrid compounds formed via cross-coupling between monolignols and hydroxystilbenes through radical reactions. We used sinapyl alcohol here to examine the cross-coupling reaction with hydroxystilbenes and produced four different dimeric structures. Sinapyl alcohol has an extra methoxyl group on the aromatic ring compared to coniferyl alcohol that limits the range of possible combinatorial products produced. As will be demonstrated, the scope of products is already substantial; coniferyl alcohol and *p*-coumaryl alcohol will be examined in a future study.

**Fig. 4.**
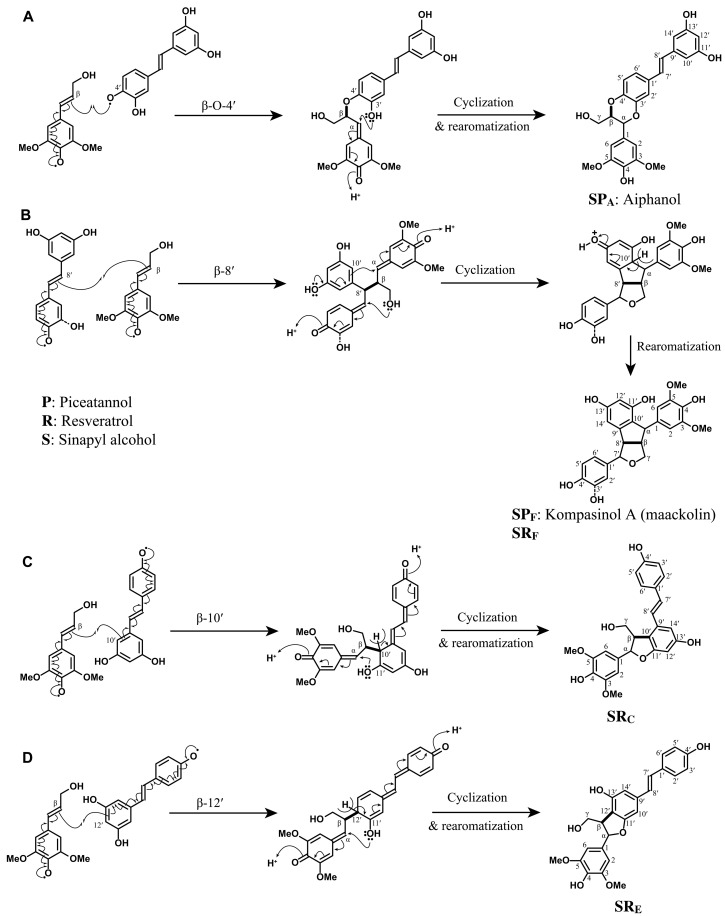
Radical coupling mechanism for cross-coupling of hydroxystilbenes with sinapyl alcohol. (**A**) Cross-coupling of sinapyl alcohol and piceatannol via β–O–4′ coupling to produce the stilbenolignol **SP**_**A**_, aiphanol, which has a six-membered ring benzodioxane structure. (**B**) Cross-coupling of sinapyl alcohol with piceatannol via β–8′ (or 8′–β) coupling to give a stilbenolignol, kompasinol A (maackolin). (**C**) Cross-coupling of sinapyl and resveratrol via β–10′ coupling to provide the stilbenolignol **SR**_**C**_, which has a five-membered ring phenylcoumaran structure. (**D**) Cross-coupling of sinapyl alcohol and resveratrol via β–12′ coupling to produce another stilbenolignol five-membered ring coumaran structure, **SR**_**E**_. Note that we isolated the racemic compounds from the radical reactions.

**SP**_**A**_ (aiphanol) is one of the well-studied stilbenolignans (Fig. 4A), and we were able to synthesize the racemic mixture thereof using peroxidase in vitro. Unlike plants that can produce optically active metabolites as defense compounds, only racemic compounds can be produced by in vitro methods that do not explicitly use chiral synthetic methods. Racemic products are also likely to be formed during lignification in plants, a process that does not involve proteinaceous control (*41*). **SP**_**A**_ is a cross-coupled dimer of piceatannol and sinapyl alcohol and has a six-membered benzodioxane ring structure that also appeared in **PP**_**A**_ (cassigarol E). The cross-coupling of sinapyl alcohol and piceatannol was initiated by β–O–4′ formation, which is similar to the 8–O–4′ ether structure of **PP**_**A**_ (fig. S5-1A). The quinone methide intermediate was quenched by intramolecular trapping with the 3′-hydroxyl group to give the cyclized structure **SP**_**A**_ as in **PP**_**A**_, but the NMR chemical shifts are different. As the sidechain of sinapyl alcohol becomes part of the benzodioxane ring structure, the distinctive NMR chemical shifts of the α- and β-positions are more similar to those in the benzodioxane structures produced during radical coupling of caffeyl and 5-hydroxyconiferyl alcohols than **PP**_**A**_, appearing at δ_C_/δ_H_ 76.2/4.99 and 78.3/4.23 in the 2D HSQC NMR spectrum. Natural aiphanol was isolated from the seeds of *A. aculeata* (*42*) and was later biomimetically prepared using Ag_2_CO_3_ (*43*).

Another cross-coupled stilbenolignan structure between sinapyl alcohol and piceatannol collected from the peroxidase radical reaction was **SP**_**F**_, known as kompasinol A or maackolin, and has a β–8′-coupled nonsymmetrical structure (Fig. 4B) (*33*, *44*). After the radical coupling at the β-position of sinapyl alcohol and the 8′-position of piceatannol, both quinone methides were internally trapped, by the resorcinol moiety of piceatannol and the γ-OH of sinapyl alcohol, and cyclized. As it is a hybrid product of piceatannol and sinapyl alcohol, the 2D NMR shows a mixed pattern common to both syringaresinol **C** and pallidol (**RR**_**F**_) moieties in the aliphatic area. Kompasinol A (maackolin) has been isolated from various plants, *Koompassia malaccensis*, *Syagrus romanzoffiana*, and *Maackia amurensis* (*44*–*46*). The combination of piceatannol and sinapyl alcohol also resulted in the trimer **SPP**_**H**_. As with **PPP**_**H-A**_, a piceatannol trimer, **SPP**_**H**_ can be formed from **PP**_**C**_ (scirpusin B). By adding a sinapyl alcohol to the **PP**_**C**_, a benzodioxane structure was formed (Fig. 3), and the 2D HSQC NMR shows the peak pattern common to both **PP**_**C**_ and **SP**_**A**_ (table S3).

Cross-coupling experiments with sinapyl alcohol and resveratrol resulted in two cross-coupled hybrid structures, **SR**_**C**_ and **SR**_**E**_, that have not been reported to date. **SR**_**C**_ is a β–10′-coupled phenylcoumaran structure and was produced similarly to **PP**_**C**_ and **RR**_**C**_ that have 8–10′ phenylcoumaran structures (Fig. 4C). However, the α- and β-positions, which are comparable to the 7- and 8-positions of **PP**_**C**_ and **RR**_**C**_ (fig. S5-1C), appeared to be more similar to the β–5′ phenylcoumaran from hydroxycinnamyl alcohols **B** at δ_C_/δ_H_ 86.6/5.74 and 52.6/3.69 in the 2D NMR spectrum, and the γ-position appeared at δ_C_/δ_H_ 63.8/3.85 and 63.8/3.49. **SR**_**E**_ is the other cross-coupled phenylcoumaran of sinapyl alcohol and resveratrol (Fig. 4D), with a β–12′-coupled structure similar to **PP**_**E**_ (gneafricanin C) and **RR**_**E**_ (gnetin C) structures (fig. S4-2) that are 8–12′ connected homo-coupled hydroxystilbenes (fig. S5-2E); **PP**_**E**_ and **RR**_**E**_ are known natural compounds, but we were unable to produce them from our radical reactions in this research. Cross-coupled **SR**_**C**_ and **SR**_**E**_ show a similar peak pattern for the sinapyl alcohol moiety in the 2D NMR, but the resveratrol moiety has its own distinctive peaks. Although these cross-coupled phenylcoumaran structures **SR**_**C**_ and **SR**_**E**_ were not authenticated in the milled wood lignins (MWLs) in fig. S2, the explicit identification of the structures as radical coupled products nevertheless showed the chemical compatibility between hydroxystilbenes and sinapyl alcohol in the oxidative conditions and strongly supports the possibility of forming such structures during lignification in vivo.

### In vitro synthetic lignin polymerization and structural analysis

To demonstrate the potential polymerization of hydroxystilbenes and their inclusion into lignin polymers, mimicking lignification in vitro by producing DHPs is a logical approach. In our previous work on palm fruit endocarp lignins, we prepared synthetic lignin oligomers as a preliminary approach for the characterization of lignin structures (*13*). In this study, various DHPs were prepared from the monolignols, coniferyl alcohol **G** and sinapyl alcohol **S**, with hydroxystilbenes piceatannol **P** and resveratrol **R**, individually and combinatorially. They were prepared under similar radical reaction conditions as for the low–molecular weight dimer/trimers but using a slow-addition technique, as described in the original “Zutropf” method (*47*). This study delivers substantial evidence for lignification’s receptivity toward hydroxystilbenes.

### Copolymer DHPs and their molecular weight distributions

Acidic reaction conditions in acetate buffer (pH 3.5) were used for the peroxidase-catalyzed polymerization, and reasonable polymer yields were successfully obtained (Table 2). The initial test for the reaction condition with coniferyl and sinapyl alcohol and the lower pH (3.5) resulted in better polymer yields compared to the higher pH (6.0). The exception to this was the sinapyl alcohol–only polymerization, which did not produce any collectible precipitates under any conditions unlike previous DHP studies that reported measurable homopolymerization of sinapyl alcohol (*48*, *49*). During the polymerization reaction, the synthesized DHPs precipitated from the solution due to the hydrophobicity of the polymers, similar to natural lignins. Each DHP solution exhibited a different color and transparency based on the supplied monomerics (fig. S6). The solution of monolignol DHPs were a cloudy beige color, with the coniferyl alcohol–only DHP being the most nontransparent. All DHPs with piceatannol provided good yields of polymers after filtration, all having dark brown coloration. Piceatannol itself also made a suitable polymer with a high yield (92%). A resveratrol-only reaction did not produce a good yield of DHP compared to the others, but cross-coupled DHPs of resveratrol and monolignols produced reasonably high yield DHPs with light brown coloration. The DHPs from sinapyl alcohol with hydroxystilbenes had slightly lower yields than other DHPs, yet we were able to collect the precipitates unlike for the sinapyl alcohol–only polymerization. Adding hydroxystilbenes in the reaction appeared to help to generate sinapyl alcohol radicals by radical transfer, a potential role that has been suggested for *p*-coumarate units in grass lignification (*50*).

**Table 2.** Weight-average (*M*_w_) and number-average (*M*_n_) molecular weights (g mol^−1^) and polydispersity (*M*_w_/*M*_n_) of DHPs. (A) Yields, *M*_w_ and *M*_n_ (g mol^−1^), and polydispersity (*M*_w_/*M*_n_) of various DHPs obtained in acetate buffer (pH 3.5). DHP yields were lower at pH 6: The G-DHP was obtained 66.5% yield, the GS-DHP was obtained only 2.8% yield, and no polymer was obtained for the attempted S-DHP. (B) *M*_w_, *M*_n_, and *M*_w_/*M*_n_ of MWLs and ELs. **G**, coniferyl alcohol; **S**, sinapyl alcohol; **P**, piceatannol; **R**, resveratrol; MWL, milled wood lignin; EL, enzyme lignin.

A. DHP samples	DHP yield (pH = 3.5)	*M*_w_	*M*_n_	*M*_w_/*M*_n_
**G** (300 mg)	231.8 mg (77.3%)	4570	3243	1.41
**S** (300 mg)	No polymer	–	–	–
**GS** (150 + 175 mg)	73.1 mg (23.1%)	6670	3498	1.91
**P** (300 mg)	276.1 mg (92.0%)	2910	1177	2.47
**PG** (300 + 221 mg)	496.1 mg (95.2%)	5594	2591	2.16
**PS** (300 + 258 mg)	345.0 mg (61.8%)	4445	1726	2.58
**PGS** (300 + 221 + 258 mg)	625.8 mg (80.2%)	5022	2511	2.00
**R** (300 mg)	60.5 mg (20.2%)	3656	1998	1.83
**RG** (300 + 237 mg)	443.3 mg (82.6%)	3206	2200	1.46
**RS** (300 + 276 mg)	226.9 mg (39.4%)	3840	1968	1.95
**RGS** (300 + 237 + 276 mg)	497.4 mg (61.2%)	3434	2125	1.62
**PRGS** (280 + 300 + 258 + 221 mg)	736.1 mg (72.0%)	4200	2135	1.97
**PR** (300 + 280 mg)	359.2 mg (61.9%)	6538	2324	2.81
**B. Lignin samples**	**DHP yield (pH = 3.5)**	***M*_w_**	***M*_n_**	***M*_w_/*M*_n_**
Macaúba MWL	39.8%*	6261	2953	2.12
Carnauba MWL	38.8%*	6089	2789	2.18
Coconut MWL	33.2%*	6407	2940	2.18
Macaúba EL	56.3%	7302	2036	3.59
Carnauba EL	53.8%	7128	1982	3.60
Coconut EL	54.0%	7614	2400	3.17

*MWL yields were measured in previous study (*13*).

To measure and evaluate the polymer sizes and heterogeneity, we examined the prepared DHPs along with MWLs and enzyme lignins (ELs) for the number-average molecular weights (*M*_n_), weight-average molecular weights (*M*_w_), and polydispersity indices (*M*_w_/*M*_n_) (Table 2). The weight-average molecular weights (*M*_w_) of G-DHP and GS-DHP were 4570 and 6670 g/mol, respectively, which are in the range of fractionated MWLs from previously published studies (*51*, *52*), and the polydispersities are relatively lower than MWLs in this study. It indicates that these DHPs are more uniform polymers than MWLs. The DHPs with piceatannol **P** showed slightly larger polydispersities than the DHPs generated with resveratrol **R**. Although the DHPs involving piceatannol were relatively nonuniform, they had higher molecular weights than DHPs with resveratrol. PG- and PGS-DHPs displayed similar molecular weights to the monolignol DHPs, and the obtained yields of PG- and PGS-DHPs were higher than others. DHPs with resveratrol exhibited lower molecular weights and polymer yields. In general, DHPs with piceatannol were more highly polymerized than the DHPs with resveratrol, but overall, hydroxystilbenes were successfully incorporated into the polymerization process; adding hydroxystilbenes into the DHP reaction resulted in equivalent or even enhanced polymerization compared to conventional DHPs from monolignols only. Compared to ELs, DHPs and MWLs resulted in lower molecular weights as the ELs represent the entire lignin from the cell walls. ELs are also less uniform than other polymers because they contain residual polysaccharides after the cellulase treatment of whole cell wall material.

Overall, the synthesized DHPs produced under slightly acid conditions were successfully collected and had decent *M_w_* and *M*_n_. The result implies that the lignins of palm fruit endocarps are reasonably homogeneous and predominantly composed of high–molecular weight polymers. Thus, it supports our argument that hydroxystilbenes are well-incorporated and exist as part of the lignin polymer in palm fruit endocarps.

### NMR (2D HSQC) analysis of hydroxystilbenes in DHPs

**Fig. 5.**
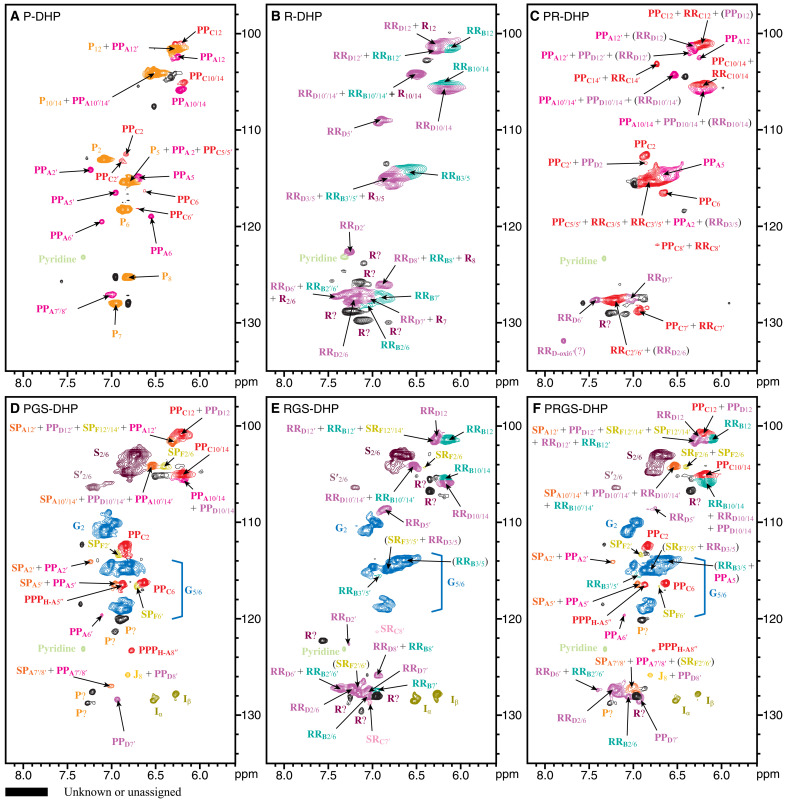
Aromatic regions of the 2D HSQC NMR spectra of the DHPs. Structures and coloring are as defined in Fig. 1. (**A**) P-DHP from piceatannol-only biomimetic coupling. (**B**) R-DHP from resveratrol only. (**C**) PR-DHP from piceatannol and resveratrol combined. (**D**) PGS-DHP from piceatannol, coniferyl alcohol, and sinapyl alcohol cross-coupling. (**E**) RGS-DHP from resveratrol, coniferyl alcohol, and sinapyl alcohol cross-coupling. (**F**) PRGS-DHP from piceatannol, resveratrol, coniferyl alcohol, and sinapyl alcohol cross-coupling. All spectra were collected in DMSO-*d*_6_:pyridine-*d*_5_ (4:1, v/v). The DHPs from piceatannol and monolignol provided evidence of the cross-coupling reactions to produce **SP**_**A**_ and **SP**_**F**_, moieties that are evident in all lignin spectra at trace levels (fig. S2). DHPs involving resveratrol also showed traces of cross-coupled structures **SR**_**C**_ and **SR**_**F**_.

**Fig. 6.**
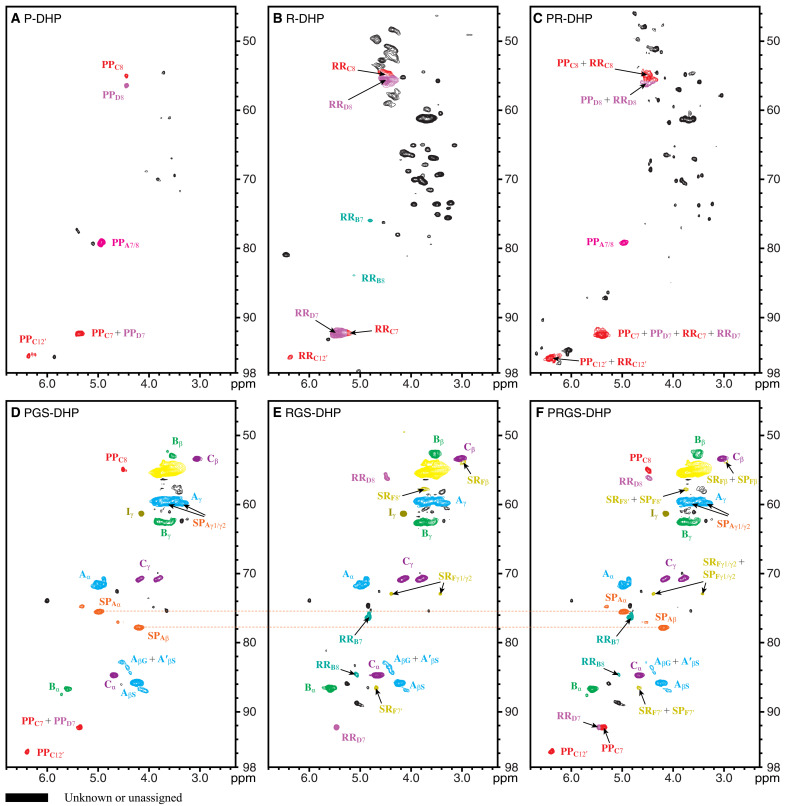
Aliphatic regions of the 2D HSQC NMR spectra of the DHPs. Structures and coloring are as defined in Fig. 1. (**A**) P-DHP from piceatannol-only biomimetic coupling. (**B**) R-DHP from resveratrol only. (**C**) PR-DHP of piceatannol and resveratrol combined. (**D**) PGS-DHP from piceatannol, coniferyl alcohol, and sinapyl alcohol cross-coupling. (**E**) RGS-DHP of resveratrol, coniferyl alcohol, and sinapyl alcohol cross-coupling. (**F**) PRGS-DHP from piceatannol, resveratrol, coniferyl alcohol, and sinapyl alcohol cross-coupling. All spectra were collected in DMSO-*d*_6_:pyridine-*d*_5_ (4:1, v/v). The DHPs of piceatannol and monolignol provided evidence of the cross-coupling reactions to produce **SP**_**A**_ and **SP**_**F**_, moieties that are evident in all lignin spectra at trace levels (fig. S2). DHPs involving resveratrol also showed traces of cross-coupled structure*s* **SR**_**C**_ and **SR**_**F**_.

The correlations in 2D HSQC NMR spectra were assigned on the basis of the model compounds described above (Figs. 5 and 6). Valuable information on the diverse types of linkages that arise when hydroxystilbenes are incorporated into the DHPs and lignin polymers is revealed.

#### Aromatic region of 2D HSQC NMR

The aromatic region of the spectra (δ_C_/δ_H_ 97 to 135/5.6 to 8.2) provides information on lignin monomer composition (Fig. 5). The characteristic peaks from hydroxystilbene aromatic units shared the same regions with those conventional units derived from monolignols but were nevertheless nicely dispersed. The piceatannol-only DHP (P-DHP) revealed a benzodioxane **PP**_**A**_ as the major unit type (from 8–O–4′ coupling) as well as minor phenylcoumaran **PP**_**C**_ from 8–10′ coupling (Fig. 5A). Piceatannol endgroups were prominent and could be on short polymers as the DHPs have lower *M*_n_ and *M*_w_ than other DHPs (Table 2). A less likely possibility is that piceatannol monomers might be trapped in the polymer matrix due to the poor solubility in the water-based solvent during the DHP preparation process. Even with the slow addition of monomers to produce lignin-like materials, the DHP is consistent with many short polymers that contain high levels of endgroups (*53*). The coupling and cross-coupling products that were found in the lignins were produced in different ratios depending on the reaction conditions (*54*). A resveratrol-only DHP (R-DHP) resulted in a more diverse polymer with a peak pattern correspondingly more complex than from the P-DHP. **RR**_**D**_, an 8–3′- (or 8–5′)-coupled phenylcoumaran was the major component (Fig. 5B). It is a structural analog of **PP**_**D**_ and shares the same 8–5′ phenylcoumaran structure. An 8–10′-coupled phenylcoumaran structure, **RR**_**C**_, was detected as a minor unit along with **RR**_**B**_, an 8–O–4′-coupled structure that is similar to the β–O–4′-coupled units in lignin. We were not able to confirm 8–8-coupled units of type **RR**_**F**_. A small area of δ_C_/δ_H_ 122 to 130/6.7 to 7.5 was congested with many peaks from endgroup double bonds and peaks from the phenol moiety of resveratrol and some unknown peaks. A copolymer DHP of piceatannol **P** and resveratrol **R** (PR-DHP) resulted in different components in the aromatic region (Fig. 5C). **PP**_**C**_ and **RR**_**C**_ were minor structures in each homo-coupling reaction of piceatannol and resveratrol, respectively, but became the major structures in this cross-coupling polymerization of **P** and **R**. The benzodioxane **PP**_**A**_ and the 8–O–4′ ether coupled **RR**_**B**_ were detected as minor structures along with **PP**_**D**_ and **RR**_**D**_. The resveratrol-related peaks at the δ_C_/δ_H_ 122 to 130/6.7 to 7.5 were slightly simpler than in the resveratrol-only R-DHP, but unknown peaks remained.

Adding hydroxystilbenes to monolignols in the radical polymerization process produced much more realistic lignin-like polymers, especially for the combination of piceatannol **P**, coniferyl alcohol **G**, and sinapyl alcohol **S**. The 2D HSQC spectrum of a PGS-DHP showed nicely resolved hydroxystilbene peaks in addition to the conventional **S** lignin peak at δ_C_/δ_H_ 104.1/6.76 and **S′** peak at δ_C_/δ_H_ 106.5/7.22 (Fig. 5D). The **G**_2_ peak appeared at δ_C_/δ_H_ 110.8/7.05, and **G**_5_ and **G**_6_ were observed at δ_C_/δ_H_ 114.9/6.86 and δ_C_/δ_H_ 118.9/6.87. Furthermore, the α and β correlations from cinnamyl alcohol endgroups appeared at δ_C_/δ_H_ 128.5/6.53 (**I**_α_) and 127.9/6.28 ppm (**I**_β_) (*55*, *56*). Typically, the hydroxycinnamyl alcohol endgroup peaks were more prominent in DHPs than in lignin samples due to the shorter polymer length. An 8–10′-coupled **PP**_**C**_ unit was the major hydroxystilbene structure in this PGS-DHP based on the aromatic area data. Massive peaks from **PP**_**C**_ appeared near the S-unit peaks at δ_C_*/*δ_H_ 100 to 107/6.0 to 6.8, and the cluster of peaks in that area also represents the resorcinol moiety of the **PP**_**A**_, **PP**_**D**_, **SP**_**F**_, and **SP**_**A**_ units. The catechol moiety peaks from piceatannol appeared in between the G-unit peaks. **PP**_**A**_ and **PP**_**C**_ were major products in the P-DHP (Fig. 5A), but **PP**_**A**_ became a minor product in this PGS-DHP whereas **PP**_**C**_ remained as the major structure (Fig. 5D). The **PPP**_**H-A**8″_ peak at δ_C_/δ_H_ 123.4/6.78 represents an unsaturated piceatannol structure. However, few unsaturated endgroup peaks of piceatannol were found at the δ_C_/δ_H_ 122 to 130/6.7 to 7.5, and only small peaks from **PPP**_**H-A**8″_, **PP**_**D**7_, **PP**_**D**8_, **SP**_**A**7′+8′_, and **PP**_**A**7′+8′_ were detected, along with two unknown peaks. This indicates that piceatannol was efficiently polymerized with monolignols to form a high-MW DHP, as supported by GPC measurements. The copolymer DHP of resveratrol **R** with coniferyl alcohol **G** and sinapyl alcohol **S** (RGS-DHP) showed a similar peak pattern as the PGS-DHP at the δ_C_/δ_H_ 100 to 108/5.8 to 6.6, but the resorcinol moiety peaks belong to the resveratrol components, primarily **RR**_**B**_ and **RR**_**D**_ (Fig. 5E). The area of **G**_2_, **G**_5_, and **G**_6_ was not congested, but the δ_C_/δ_H_ 122 to 130/6.7 to 7.5 was overcrowded with endgroup double bonds and the B-ring (phenolic moiety; Fig. 1C) of resveratrol as in the R-DHP in Fig. 5B. The peaks from **G** and **S** endgroups, **I**_α_ and **I**_β_, appeared to be elevated over their levels in the PGS-DHP. MW and the polymer yield of the RGS-DHP were also much lower than for the PGS-DHP (Table 2A), so the results suggest that the PGS-DHP better incorporates the range of monomers forming a longer polymer than for an RGS-DHP. The peaks of **RR**_**C**_, **RR**_**C-1**_, **RR**_**F**_, and **SR**_**C**_ were also detected near the noise level.

The last copolymer DHP (PRGS-DHP) we present in this study is a combination of all components, piceatannol **P**, resveratrol **R**, coniferyl alcohol **G**, and sinapyl alcohol **S** (Fig. 5F). The NMR data are much more complex than from other DHPs because it has all the components we found from the PGS- and RGS-DHPs. The crowded area at δ_C_/δ_H_ 100 to 108/5.8 to 6.6 was filled with correlations from all the resorcinol moieties (A-rings) of both piceatannol and resveratrol. Piceatannol’s catechol moiety (B-ring) appeared in the area of the G-unit peaks as in the PGS-DHP, and the phenolic moiety (B-ring) of resveratrol appeared at δ_C_/δ_H_ 122 to 130/6.7 to 7.5 as seen in the RGS-DHP. Both piceatannol **P** and resveratrol **R** were evenly polymerized with coniferyl alcohol **G** and sinapyl alcohol **S** without preference and formed decent polymers with relatively high molecular weights and reasonable polymer yields (Table 2A).

#### Aliphatic region of 2D HSQC NMR

The aliphatic region (δ_C_/δ_H_ 45 to 98/2.3 to 6.8) profiles the chemical connectivity of lignin (Fig. 6). This area generally includes the sidechain structures of lignins, β-aryl ethers **A**, phenylcoumarans **B**, resinols **C**, and cinnamyl alcohol endgroups **I** (*55*, *56*). Some of the newly identified correlations from hydroxystilbenes were discovered in this area too. The P-DHP showed **PP**_**A**_ (**PP**_**A**7+8_ at δ_C_/δ_H_ 79.3/4.94) with benzodioxane and **PP**_**C**_ with 8–10′-coupled phenylcoumaran structures in the aromatic region (Fig. 6A). A well-isolated **PP**_**C**12′_ peak from the resorcinol moiety also distinctively appeared at δ_C_/δ_H_ 95.8/6.37. In addition, the **PP**_**D**8_ peak at δ_C_/δ_H_ 56.5/4.45 was observed near the **PP**_**C**8_ peak. Trace levels of **PP**_**D**_ were identified in the aromatic region in Fig. 5A, but it is clearly visible in Fig. 6A along with **PP**_**C**_ in the aliphatic region. The R-DHP data unveiled **RR**_**D**_, the 8–3′-coupled phenylcoumaran, as the major structure in the aliphatic region (Fig. 6B), supporting the observations from the aromatic region (Fig. 5B). **RR**_**C**7_ and **RR**_**C**8_ have the same chemical shifts as **RR**_**D7**_ and **RR**_**D8**_, and the peaks could not be separated. However, the **RR**_**C**12′_ peak was found at δ_C_/δ_H_ 95.8/6.37 where **PP**_**C**12′_ peak appeared in the P-DHP (Fig. 6A). The unique **RR**_**B**_, 8–O–4′-coupled structure was detected weakly, and small peaks from **RR**_**B**7_ and **RR**_**B**8_ units were found at δ_C_/δ_H_ 76.1/4.81 and δ_C_/δ_H_ 84.0/5.13. The PR-DHP showed the combination of P-DHP and R-DHP components (Fig. 6C). With slightly predominant piceatannol peaks, **PP**_**A**_, **PP**_**C**_, and **PP**_**D**_, resveratrol components, **RR**_**C**_ and **RR**_**D**_, but not **RR**_**B**_, were detected, supporting observations from the aromatic region.

In the previous section, we noted that the NMR peak patterns in the aromatic region of PGS-DHP (Fig. 5D) were similar to those in the lignin data in fig. S2. Similarly, the aliphatic region of the spectrum of PGS-DHP also reflected the most realistic lignin-like polymer among the copolymer DHPs (Fig. 6D). **PP**_**C**_ was confirmed as the major piceatannol structure in the PGS-DHP, but there were slight alterations of the piceatannol compositions compared to the aromatic regions. **PP**_**D**_ was hardly visible, and the **PP**_**A**_ level was low. The aliphatic region of RGS-DHP also showed the normal lignin components and resveratrol compositional structures (Fig. 6E). **RR**_**D**_, the 8–3′-coupled phenylcoumaran remained as a major structure, and the **RR**_**B**_ peaks were clearly observable, but **RR**_**C**_ was only found at a trace level. The aliphatic region of PRGS-DHP (Fig. 6F) is another combination of features from both the PGS-DHP and the RGS-DHP, as noted in the aromatic region. The NMR spectrum became more complex compared to those from other DHP data, largely because it has all the compositions combined. The aliphatic area of all DHPs consistently showed all components, piceatannol **P**, resveratrol **R**, coniferyl alcohol **G**, and sinapyl alcohol **S**, and the results agree with the aromatic region data.

### Cross-coupled structures in DHPs from hydroxystilbenes and monolignols

An important goal of this study was to provide tangible evidence for the incorporation of hydroxystilbenes into lignin polymers. Stilbenolignins and stilbenolignan extractives can result from radical cross-coupling between hydroxystilbenes and monolignols. Yet, only a handful of stilbenolignans has been reported previously (*10*), mostly involving piceatannol **P** and isorhapontigenin **IS**. The most common products are benzodioxanes, phenylcoumarans, and the 3-oxabicyclo-[3.3.0]-octane ring structures. We have synthesized and collected products using piceatannol **P** and resveratrol **R** and used them to identify the structures in the DHPs and lignins. Aiphanol **SP**_**A**_, a benzodioxane, is the most well-known cross-coupled structure between piceatannol and sinapyl alcohol, and we were able to isolate it from the peroxidase reaction in this study. In the aromatic region, most **SP**_**A**_ peaks were superimposed on other hydroxystilbene correlations, but the benzodioxane peaks from **SP**_**A**_ (trans-stereoisomer) at δ_C_/δ_H_ 75.7/4.98 (**SP**_**A**α_) and δ_C_/δ_H_ 78.0/4.19 (**SP**_**A**β_) were identified from the aliphatic region of the PGS-DHP and PRGS-DHP data (Fig. 6). The cis-isomer also appeared as smaller correlations at δ_C_/δ_H_ 74.9/5.31 (**SP**_**A**α_) and δ_C_/δ_H_ 77.2/4.61 (**SP**_**A**β_) near the prominent trans-isomer peaks. Both trans- and cis-benzodioxanes result from the ring closure and rearomatization following radical coupling, whereas only the trans-isomers, which may be different from the benzodioxanes produced by radical coupling, are produced by Diels-Alder (4 + 2 cycloaddition) reactions via the corresponding *o*-quinone. For example, Diels-Alder reactions between coniferyl alcohol and the *o*-quinone from methyl 5-hydroxyvanillate produced a trans-benzodioxane structure with a different regiochemistry than the isomer produced via radical coupling, and a unique oxatricyclo structure when the quinone’s diene participated in the Diels-Alder reaction (*57*). However, we could not find the evidence for such products from lignins. We expect analogous results from piceatannol. The level of **PP**_**A**_, which also has a benzodioxane structure between piceatannols, was severely reduced in the copolymer DHPs (Fig. 6, D and F) compared to the P-DHP; apparently, there is competition between the stilbenes to produce the different benzodioxane structures, **SP**_**A**_ and **PP**_**A**_, during the polymerization process.

Phenylcoumaran (β–5′-coupled) stilbenolignans involving isorhapontigenin in gnetofuran A–like structures were naturally found in plants (fig. S4-1) (*10*). In this study, we could not isolate the β–5′ cross-coupling reaction between sinapyl alcohol and piceatannol or resveratrol. However, we did isolate two cross-coupled phenylcoumaran structures, **SR**_**C**_ (β–10′) and **SR**_**E**_ (β–12′), between sinapyl alcohol and resveratrol (Fig. 1F). Despite the tiny correlation peaks, we were able to locate the **SR**_**C**7′_ at δ_C_/δ_H_ 128.9./7.04 and **SR**_**C**8′_ at δ_C_/δ_H_ 121.5/6.95 from the aromatic area of RGS-DHP data (Fig. 5E). Another possible β–12′ cross-coupled structure, phenylcoumaran **SR**_**E**_ (Fig. 1F), was not detected in the DHPs or lignins but may still possibly exist as a minor component in the polymers.

Identifying the β–8′-coupled nonsymmetrical structure between sinapyl alcohol and piceatannol, kompasinol A (maackolin) **SP**_**F**_, in DHPs was an expected outcome. Despite most **SP**_**F**_ peaks in the aromatic region being obscured by other major peaks, including **SP**_**F**2/6_ at δ_C_/δ_H_ 104.3/6.41, **SP**_**F**2′_ at δ_C_/δ_H_ 113.5/6.91, and **SP**_**F**6′_ at δ_C_/δ_H_ 116.7/6.71 (Fig. 5D), it was possible to identify the structure as a trace component in the aliphatic region at the noise level (Fig. 6D). In addition, an unexpected kompasinol A–like β–8′ cross-coupled structure **SR**_**F**_ (Fig. 1F), an analog of **SP**_**F**_ between resveratrol and sinapyl alcohol, was found in the RGS-DHP (Figs. 5E and 6E). We were not able to isolate compound **SR**_**F**_ from the radical reactions but identified the related peaks from the RGS-DHP and the PRGS-DHP as the chemical shifts of the sidechain peaks of **SR**_**F**_ matched those of **SP**_**F**_ because they have similar structures (except for the 3′-OH). In the aliphatic area (Fig. 6E), peaks **SR**_**F**7′_ at δ_C_/δ_H_ 86.7/4.69, **SR**_**F**8′_ at δ_C_/δ_H_ 57.9/3.76, **SR**_**F**α_ at δ_C_/δ_H_ 49.6/4.14, **SR**_**F**β_ at δ_C_/δ_H_ 53.9/2.98, and **SR**_**F**γ1/γ2_ at δ_C_/δ_H_ 73.0/4.39 and 3.43 appeared stronger in the RGS-DHP and PRGS-DHP than did **SP**_**F**_ peaks in the PGS-DHP. The **SR**_**F**_ aromatic peaks appeared to be complicated by other major peaks, especially for the 2′/6′ and 3′/5′ correlations from resveratrol units (Fig. 5E). This DHP study has provided evidence that resveratrol, like piceatannol, has the potential to be incorporated into lignin polymers.

### DFRC releases hydroxystilbene monomers from DHPs and lignin polymers

To provide further evidence for the inclusion of hydroxystilbenes into the lignin polymer, we analyzed the DHPs and MWLs by the DFRC method, a degradative analytical technique that cleaves β-ether bonds in the lignin polymer (Table 3) (*58*). The conventional (acetylated) G and S lignin monomers were released as cis- and trans-isomers. From the DHPs, piceatannol and resveratrol were obtained as bothcis- and trans-isomers and confirmed by the piceatannol and resveratrol standards that were nicely resolved from other standard compounds and identified using gas chromatography–mass spectrometry (GC-MS; fig. S7). A high level of resveratrol was detected from the RGS-DHP and the PRGS-DHP, but only small amounts of piceatannol were released from the PGS-DHP and PRGS-DHP (Table 3A), although all DHPs showed highly incorporated hydroxystilbenes by NMR (Figs. 5 and 6) for the reason noted below. Among the structures in Fig. 1, the only DFRC-cleavable hydroxystilbene structure is the resveratrol 8–O–4′-coupled structure **RR**_**B**_. Other possibly releasable structures can exist as ether bonded structures, **SR**_**A**_, **PR**_**B**_, **SP**_**B**_, and **SR**_**B**_ (fig. S4-1), but we could not confirm such structures in this study. Other structures are C—C connected and cannot be cleaved by DFRC. Piceatannol dimeric units **PP**_**A**_ and cross-coupled **SP**_**A**_ are also known as noncleavable structures under the DFRC degradation conditions from a previous study of benzodioxane structures produced via 5-hydroxyconiferyl alcohol (*59*). As a result, only a small amount of piceatannol was released from the limited 8–O–4′ cross-coupled structures, but a large amount of resveratrol from the 8–O–4′ structures **RR**_**B**_ was detected from the DHPs (fig. S7 and Table 3A). The DHP result can be directly compared to the low levels of hydroxystilbene products of MWLs by DFRC (Table 3A). Although we confirmed that all MWLs have high piceatannol contents by NMR (fig. S2), the DFRC method released small amount of piceatannol; the low levels of the releasable monomers might be due to the difficulty of degrading the polymers.

**Table 3.** DFRC analysis of DHPs and MWLs. (A) Lignin monomers and hydroxystilbenes released from DHPs and MWLs by reductive cleavage of β–O–4′ or 8–O–4′ ether structures (determined by GC-MS). (B) After the DFRC reaction, the entire reaction products were collected and examined by 2D HSQC NMR and semiquantified for the components (see text). **H**, *p*-coumaryl alcohol; **G**, coniferyl alcohol; **S**, sinapyl alcohol; **CA-*p*BA**, coniferyl *p*-hydroxybezoate; **SA-*p*BA**, sinapyl *p*-hydroxybezoate; **SA-BA**, sinapyl benzoate.

A. DFRC results of DHPs and MWLs (*n* = 2)									
		%H	%G	%S	%Piceatannol	%Resveratrol	%CA-*p*BA	%SA-*p*BA	%SA-BA
DHPs	PGS		66.0 ± 0.2	33.8 ± 0.2	0.1 ± 0.0	–	–	–	–
	RGS	–	40.1 ± 0.5	25.8 ± 0.0	–	34.1 ± 0.5	–	–	–
	PRGS	–	47.9 ± 0.6	25.4 ± 0.1	1.0 ± 0.1	25.6 ± 0.8	–	–	–
MWLs	Carnauba	2.4 ± 0.5	70.9 ± 2.3	11.4 ± 0.3	1.1 ± 0.4	0.3 ± 0.3	0.4 ± 0.0	13.4 ± 1.9	0.0 ± 0.0
	Macaúba	1.0 ± 0.2	58.3 ± 1.8	10.6 ± 0.2	0.8 ± 0.1	0.1 ± 0.0	2.2 ± 0.0	26.4 ± 1.5	0.5 ± 0.0
	Coconut	5.4 ± 0.1	67.3 ± 0.3	14.4 ± 0.4	0.3 ± 0.1	0.0 ± 0.0	0.2 ± 0.0	12.3 ± 0.1	0.0 ± 0.0
**B. NMR estimation of DFRC products (*n* = 2)**									
		%H	%G	%S	%Piceatannol	%Resveratrol	%Total	*%p*BA*	%BA*
DHPs	PGS	–	71.6 ± 0.2	28.4 ± 0.2	0.0 ± 0.0	–	100	–	–
	RGS	–	41.6 ± 0.8	17.4 ± 1.0	–	41.0 ± 0.2	100	–	–
	PRGS	–	45.3 ± 0.9	22.0 ± 1.0	0.0 ± 0.0	32.7 ± 0.1	100	–	–
MWLs	Carnauba	0.7 ± 0.4	54.4 ± 2.0	37.3 ± 2.9	7.5 ± 1.3	0.0 ± 0.0	100	54.4 ± 0.4	0.0 ± 0.0
	Macaúba	0.5 ± 0.1	53.8 ± 0.5	42.6 ± 0.6	3.1 ± 0.2	0.0 ± 0.0	100	68.1 ± 3.5	1.4 ± 0.1
	Coconut	3.2 ± 0.8	62.4 ± 1.3	34.3 ± 2.2	0.0 ± 0.0	0.0 ± 0.0	100	35.2 ± 0.2	0.0 ± 0.0

*% to total lignin content.

To confirm and support the GC-MS analysis of the DFRC products, we examined the entire crude mixture by 2D HSQC NMR and semiquantitatively estimated the monomeric product compositions (Table 3B). The produced resveratrol monomer contents from DHPs were high, but the piceatannol was hardly detected, in agreement with the results from GC-MS. Slightly higher percentages of piceatannol products from MWLs were noted, but they remained at low levels. The NMR quantification results support the DFRC-GC-MS analysis of MWLs, and we recognize that the actual amounts of hydroxystilbenes being incorporated into the lignins in plants are plausibly higher than those easily detected by their release.

Both results from GC-MS and NMR showed that the lignin from carnauba had the highest content of hydroxystilbenes, followed by the lignins from macaúba and coconut. The trace amounts of resveratrol released from MWLs presumably indicates simply its low content in the lignins.

Although both piceatannol and resveratrol have similar reactivity in radical reactions, there are substantial differences noted in the levels in various lignins. As both hydroxystilbenes can be readily polymerized under the DHP synthetic conditions in vitro, the radical reactivity between piceatannol and resveratrol should be comparable. That we have always found significantly more piceatannol than resveratrol in natural lignins presumably implicates a higher availability of piceatannol than resveratrol in the cell wall during lignification. It is not clear what unknown factors may affect the wall-destined production of the various hydroxystilbenes.

### DFT study supports the compatibility of hydroxystilbenes with lignification

To better understand the radical coupling behavior between hydroxystilbenes and monolignols, the thermodynamic preferences of the reactions were examined using DFT calculations by evaluating the energetics of quinone methide formation, rearomatization, and dehydrogenation. This approach has been broadly applied to earlier studies of lignin reactions and kinetics (*60*–*65*).

We recently reported Gibbs free energy for some of the hydroxystilbene model structures to validate the radical coupling. Radical spin densities, quinone methide intermediates formation, and rearomatization to produce the final products were examined to understand the radical coupling and cross-coupling propensities and the possible selectivity of the reactions (*66*). In the current work, the Gibbs free energy of the initial dehydrogenation reaction for the monomers was calculated, and the hydrogen abstraction from the catechol moiety (not resorcinol) of piceatannol was similar to that from the monolignols. The calculations were performed for the combinatorial dimeric model structures of piceatannol, resveratrol, and sinapyl alcohol (figs. S4-1 and 4-2). Most of the radical couplings forming quinone methides are exergonic reactions (Table 4A), but the Gibbs free energy results from most of the cross-coupling were more exergonic. The quinone methide formation reactions under examination in this study do not match perfectly with the actual radical reaction products, but a notable observation is that the quinone methide intermediates formed in the C—O—C ether–coupled structures (A and B) show more exergonic reaction values than C—C bonded structures (C, D, E, and F). The overall energies and the final products can be affected by the following rearomatization step, which delivers a significant driving force. Table 4B shows the Gibbs free energy for the rearomatization reactions of the quinone methides. Overall, rearomatization energies for these reaction values are large, and the rearomatization of C—C coupled structures are more exergonic than the C—O—C ether bonded structures, reversing the trend observed in the radical reaction forming quinone methides. The same trend was found in the total Gibbs free energy of forming quinone methides and rearomatization (Table 4C). Overall, structures that composed of hydroxystilbenes had exergonic reaction values regardless of homo- and cross-coupling and indicate that forming hydroxystilbene polymers is a highly favorable radical reaction.

**Table 4.** Reactions of piceatannol P, resveratrol R, and sinapyl alcohol S. (A) Gibbs free energies of reaction for quinone methide (QM) formation. (B) Gibbs free energies of rearomatization by quenching QM (ring formation or nucleophilic addition of water). (C) Total Gibbs free energies of QM formation and rearomatization. (D and E) Gibbs free energies of dehydrogenation. All values are in kcal mol^−1^. The combination of row and column indicates the hydroxystilbene structures in figs. S4-1 and S4-2, e.g., the combination of **PP** and **A** is indicated as **PP**_**A**_.

A. QM (quinone methide) formation						
	PP	RP	PR	RR	SP	SR
**A** (8– or β–O–4′)	−24.65	−19.47			−23.19	−16.81
	−24.65	−19.47	−21.5	−15.92	−24.72	−17.76
**C** (8– or β–10′)	−2.56	0.32	0.55	5.87	−4.45	1.17
**D** (8– or β–5′)	−17.82	−12.97	−14.01	−9.03	−21.58	−17.87
**E** (8– or β–12′)	−9.69	−3.83	−0.23	5.9	−6.33	−1.84
**F** (8– or β–8)				−11.7	−20.11	−16.15
**B. Rearomatization**						
	**PP**	**RP**	**PR**	**RR**	**SP**	**SR**
**A** (8– or β–O–4′)	−20.56	−20.84			−23.08	−22.05
**B** (8– or β–O–4′)	−15.97	−18.69	−16.32	−20.53	−13.32	−16.14
**C** (8– or β–10′)	−52.9	−54.36	−52.99	−57.2	−49.61	−53.99
**D** (8– or β–5′)	−33.53	−37.07	−37.73	−41.27	−30.91	−34.7
**E** (8– or β–12′)	−44.93	−49.43	−52.85	−57.8	−50.21	−53.19
**F** (8– or β–8)				−54.29	−40.42	−43.58
**C. Total (QM formation + rearomatization)**						
	**PP**	**RP**	**PR**	**RR**	**SP**	**SR**
**A** (8– or β–O–4′)	−45.21	−40.31			−46.27	−38.86
**B** (8– or β–O–4′)	−40.62	−38.16	−37.82	−36.45	−38.04	−33.9
**C** (8– or β–10′)	−55.46	−54.04	−52.44	−51.33	−54.06	−52.82
**D** (8– or β–5′)	−51.35	−50.04	−51.74	−50.3	−52.49	−52.57
**E** (8– or β–12′)	−54.62	−53.26	−53.08	−51.9	−56.54	−55.03
**F** (8– or β–8)				−65.99	−60.53	−59.73
**D. Dehydrogenation to stilbene dimers (4–O-)**						
	**PP**	**RP**	**PR**	**RR**	**SP**	**SR**
**A** (8– or β–O–4′)	79.78	80.14			74.73	73.96
**B** (8– or β–O–4′)	75.82	80.22	78.91	78.93	76.86	78.16
**C** (8– or β–10′)	79.52	79.58	79.44	79.09	74.38	74.49
**D** (8– or β–5′)	79.58	79.52	79.43	79.24	74.86	74.47
**E** (8– or β–12′)	79.47	79.39	79.19	79.28	74.56	74.69
**F** (8– or β–8)				79.46	74.37	74.22
**E. Dehydrogenation to stilbene dimers (4′–O-)**						
	**PP**	**RP**	**PR**	**RR**	**SP**	**SR**
**C** (8– or β–10′)	76.62	76.87	75.82	75.72	76.88	75.91
**E** (8– or β–12′)	76.24	76.30	75.15	75.20	76.24	74.66

In addition, the dehydrogenation of the dimeric structures was examined (Table 4D). Measuring dissociation energies of hydrogen from the hydroxyl group of phenols can show that the dimers can feasibly participate in the lignin polymerization or as the initiation site for polymerization. The dehydrogenations of the cross-coupled dimers are thermodynamically preferred over the hydroxystilbene dimers. Likewise, the β–10′ and the β–12′ phenylcoumaran dimers can also react from the 4′–O-end, and these are more internally consistent between the homo-coupled hydroxystilbenes and cross-coupled sinapyl alcohol and hydroxystilbenes structures (Table 4E). Regardless of the structural features, these reactions would be competitive with the 4′–O-positions, and the following polymerization steps in lignification should be able to proceed on both sides.

Although some estimations of hydroxystilbene formation were not entirely consistent with the yields of our synthesized models, and as the compounds were synthesized under various reaction conditions using different catalysts to obtain various products, this estimation of the dehydrogenation energies for the homo-coupled and cross-coupled products revealed that, in general, the thermodynamics of the reaction are comparable to those of the monolignol dimers. This DFT result strongly supports the compatibility of hydroxystilbenes with lignification and their potential for incorporation into the growing polymer. The thermodynamics correlated with formation and reactions involving hydroxystilbenes are comparable with those of the monolignols during the lignification.

We have shown here that polymers and copolymers of hydroxystilbenes with traditional monolignols can be successfully produced in vitro under peroxidase-catalyzed conditions. Furthermore, low–molecular weight homo-coupled compounds from the hydroxystilbenes, resveratrol and piceatannol, and cross-coupled compounds between hydroxystilbenes and sinapyl alcohol, a monolignol, were successfully produced using various catalysts under oxidative reactions. On the basis of this model study, the structures of DHPs with hydroxystilbenes were elucidated, and the MWLs of palm fruit endocarp were also examined. The structural investigation of DHPs and lignins provided tangible evidence for the incorporation of hydroxystilbenes into the lignin polymers. All the model and DHP data collected here support the hypothesis of chemically controlled lignin polymerization based on a radical coupling process (*3*, *41*, *67*). This study indicates that there are tremendous opportunities to engineer plants to produce new kinds of biomass with different characteristics and possibly enhanced value. As piceatannol- and resveratrol-containing components could provide additional antioxidant properties to the endocarp because they have antiviral, antibacterial, and antioxidant properties (*13*), they are expected to contribute to plant disease resistance (*17*–*19*). Hydroxystilbenes can be obtained from agricultural and forest residues in low-value lignocellulose processing mills, from palm fruit shells and spruce bark, affording new opportunities for the valorization of these currently underused residues (*9*, *10*). We anticipate that bioengineering approaches for partial monolignol substitution with hydroxystilbenes in the future could provide unique characteristics to biomass.

## MATERIALS AND METHODS

### General

Most solvents, chemicals, and enzymes were purchased from Sigma-Aldrich (Milwaukee, WI, USA). Piceatannol was purchased from Cayman Chemical (Ann Arbor, MI, USA), and resveratrol was purchased from Xi’an Zelong Biotech Co. Ltd. (Shaanxi, China). Japanese knotweed was purchased from Nuherbs Organics (Oakland, CA, USA). Thin-layer chromatography was performed on 1- and 2-mm precoated glass plates (Silica Gel GF, UV254) from Analtech (Newark, DE, USA).

### Plant materials

Cell wall samples were collected as previously described (*13*, *28*). Macaúba (*A. aculeata*) and carnauba (*C. prunifera*) palm fruits were collected from Mirabela, Minas Gerais, Brazil. The coconut (*C. nucifera*) samples were from India. The fruit endocarps were separated manually using a knife and dried in an oven at 40°C. The dried samples were milled using a knife mill and extracted using a Soxhlet for 8 hours with acetone (8 hours) and hot water (3 hours at 100°C). The reported Klason lignin contents were 39.8, 38.8, and 33.2% in macaúba, carnauba, and coconut endocarp, respectively.

### Lignin preparation from palm fruit endocarp

The MWLs from macaúba, carnauba, and coconut fruit endocarps were isolated using the experimental conditions previously described (*13*, *68*). Briefly, ~40 g of extractive-free samples was ball-milled in a PM100 mill (Restch, Haan, Germany) for 6 hours, at 400 rpm, in a 500-ml agate jar and using agate ball bearings (20 × 20 mm). The finely powdered samples were extracted with 1 liter of dioxane:water 96:4 (v/v) with stirring in the dark for 24 hours. The solution was centrifuged, and the supernatant was collected by decantation. The extraction process was repeated two more times, using fresh dioxane:water each time, and the collected supernatants were combined and evaporated to dryness on a rotary evaporator at 40°C. The residue obtained (crude lignin) was then purified as described elsewhere (*68*). The final yields were about 15% of the Klason lignin. The lignin preparation obtained in this way, known as “milled wood lignin”, preserves intact the main structural characteristics of lignin in its native form (*69*).

The ELs were prepared from extractive-free cell walls as detailed in a previous publication (*70*). Briefly, the extractive-free ball-milled cell walls were treated with cellulases (Cellulysin, EC 3.2.1.4; activity, >10,000 units/g; Calbiochem) from *Trichoderma viride*. The cell walls (450 mg) were suspended in NaOAc buffer (pH 5), and 22.5 mg of Cellulysin was added. The reaction mixture was shaken on a rotary incubator shaker at 35°C for 48 hours. The residue was collected by centrifugation (8000 rpm, 30 min), and the enzyme digestion process was repeated three times. The collected residue was sonicated and washed with deionized water (20 ml) three times and lyophilized. The collected EL contents were 56.3 and 53.8% for macaúba and carnauba, respectively (Table 2B).

### DHP preparation

The synthesis of the DHPs was performed as previously reported (*70*, *71*). Different combinations of hydroxystilbenes, piceatannol and resveratrol, and monolignols, coniferyl and sinapyl alcohols, were used to produce structurally diverse DHPs (Table 2A). Hydroxystilbenes and hydroxycinnamyl alcohols were dissolved together in acetone/water (1:2, v/v). Horseradish peroxidase (EC 1.11.1.7, 150 purpurogallin units per mg solid, type II, from Sigma-Aldrich) was prepared in acetate buffer (pH 3.5), and hydrogen peroxide (H_2_O_2_, 30%) was prepared in reverse-osmosis (RO) water in a separate flask. The three solutions were slowly added over 24 hours using a peristaltic pump simultaneously to a 1-liter flask containing 200 ml of RO water at room temperature with stirring. The precipitated synthetic lignin was isolated by filtration through a 0.8-μm nylon membrane filter. The polymer was washed with excess RO water to remove remaining enzymes, and the retained solids were lyophilized.

### NMR experiments

NMR spectra were acquired on a Bruker Biospin (Billerica, MA) Avance NEO 700 MHz spectrometer equipped with a 5-mm QCI ^1^H/^31^P/^13^C/^15^N cryoprobe with inverse geometry (proton coils closest to the sample) or on an Avance III 500 MHz spectrometer equipped with a 5-mm TCI ^1^H/^13^C/^15^N cryoprobe. Model compounds were examined in acetone-*d*_6_, MeOH-*d*_4_, and dimethyl sulfoxide (DMSO)–*d*_6_:pyridine-*d*_5_ (4:1, v/v). The central acetone peak (δ_C_ 29.8, δ_H_ 2.04 ppm), methanol peak (δ_C_ 49.0, δ_H_ 3.30 ppm), and DMSO solvent peak (δ_C_ 39.5, δ_H_ 2.49 ppm) were used as the internal references. The lignin and DHP samples were examined in DMSO-*d*_6_:pyridine-*d*_5_ (4:1, v/v) as previously described (*55*, *56*). Isotopically enriched pyridine-*d*_5_ (“100”; ≧ 99.94 atom% D) was used to avoid interference between the residual solvent peaks and correlations from aromatic moieties. All NMR experiments used Bruker’s standard pulse programs; an adiabatic ^1^H–^13^C 2D HSQC experiment (hsqcetgpsisp2.2; phase-sensitive gradient-edited 2D HSQC using adiabatic pulse sequences for inversion and refocusing) was used to collect the main data (*72*). For the lignin samples, the HSQC experiments were acquired from 11.5 to −0.5 ppm (12 ppm spectral width) in F2 (^1^H) with 3366 data points (acquisition time, 200 ms) and 215 to −5 ppm (220 ppm spectral width) in F1 (^13^C) with 620 increments (F1 acquisition time, 8.0 ms) of 32 scans with a 1-s interscan delay (D1); the d24 delay was 0.86 ms (1/8J, *J* = 145 Hz). The total acquisition time for a sample was 5.5 hours. Similar conditions were used for whole cell wall samples but acquired with 1682 data points (acquisition time of 100 ms) in F2 and 620 increments (acquisition time of 8 ms) in F1. The number of scans (NS) was 56 with a 500-ms interscan delay (D1). The total acquisition time for each was 5 hours. DHP samples were examined with 3448 data points (acquisition time of 200 ms) in F2 and 618 increments (acquisition time of 8 ms) in F1. NS was 30 with a 1-s D1. The total acquisition time for each was 6 hours. The spectra were processed using Gaussian apodization (GB = 0.001) and line broadening (LB = −0.5) in F2 and squared cosine bell and 32 coefficients of linear prediction (LPfc) in F1. Volume integration of contours in HSQC plots was carried out using TopSpin 4.1.1 (Mac version) software. For quantification of H/G/S distributions, the H2/6 and S2/6 correlations were used, and the G2 integrals were doubled to be on the same atom basis. For relative estimation of the various interunit linkage types, the well-resolved α-C/H contours were measured, and the percentages are reported. However, they are semiquantitative as the more mobile endgroups are overrepresented. Hence, the data are presented on an S + S′ + G + H + P = 100% basis (fig. S2, P is for piceatannol units) as *p*CA, *p*B, and BA units are always considered to be terminal groups and are also overestimated. For the aromatic integration, S2/6, G2, and H2/6 were estimated for the normal lignin units as usual. **PP**_**C**6_ and **PP**_**A**6_ peaks were used for hydroxystilbenes because **PP**_**C**2_ and **PP**_**A**2_ were not resolved from normal G-unit peaks. The *p*-hydroxybenzoate (*p*B) peak was not included in the sum of total lignin and is expressed simply as a percentage of that total. In fig. S2, the various lignin units in the aliphatic area were relatively quantified via the volume integrals of the **A**_α_, **B**_α_, **C**_α_, **C′**_α_, **PP**_**A**7_, **PP**_**C**7_, and **SP**_**A**α_ correlation peaks.

For the structural elucidation and assignment authentication of the isolated compounds, the NS was adjusted depending on the amount and the signal to noise required from a sample. The standard Bruker implementations of the traditional suite of 1D and 2D NMR experiments (DEPT-135, COSY, HSQC, HSQC-TOCSY, and HMBC) were used for all compounds.

### Gel permeation chromatography

For the GPC analyses, 5 mg of MWL or DHP was acetylated with acetic anhydride/pyridine (1:1, v/v) and dissolved in 1 ml of tetrahydrofuran (THF) without a stabilizer. A 1-μl aliquot of the solution was injected and analyzed in a Prominence-i LC-2030 3D GPC system (Shimadzu, Kyoto, Japan) equipped with a photodiode array (PDA) detector, using the following conditions: column, PLgel 5 μm MIXED-D, 7.5 mm by 300 mm (Agilent Technologies, United Kingdom); THF as eluent; flow rate, 0.5 ml min^−1^; temperature, 40°C; sample detection, PDA response at 280 nm. The data acquisition and computation used LabSolution GPC software version 5.82 (Shimadzu). The molecular weight calibration was via polystyrene standards (MW ranged from 5.8 × 102 up to 3.24 × 106 Da; Agilent Technologies).

### Derivatization followed by reductive cleavage

DFRC was performed according to the original protocol (*13*, *58*, *73*). MWLs (30-mg scale) and DHPs (20-mg scale) were examined, and two replicates (*n* = 2) were used. The degradation products were analyzed by GC-MS (Shimadzu GC-2010 with mass spectrometer: GCMS-QP2010 Plus) fitted with a fused silica high-temperature capillary column [Phenomenex Zebron ZB-5HT Inferno Column, 15 m, 0.25 mm inside diameter (ID), 0.25-μm film thickness (d_f_)]. An aliquot of the product solution (1 μl) was injected at a split ratio of 20:1. Helium was used as the carrier gas at a linear velocity of 55 cm/s. The oven was heated from 100°C and held for 1 min, then ramped at 10°C/min to 300°C, and held for 15 min at that temperature. The injector was set at 250°C, and the transfer line was kept at 300°C. The acetylated DFRC standard compounds were prepared and identified on the basis of their mass spectra and relative retention times and quantified using acetylated 1,1-bis-(4-hydroxyphenyl)ethane (BPO), 1,1-bis-(4-acetoxyphenyl)ethane (BPA), as an internal standard. The ratios for the components were calculated using the peak areas for each batch and used the response factors.

### DFT study for radical coupling

With both the initial coupling and rearomatization, the products can have numerous rotational degrees of freedom. To address this, a conformational search was performed using a 1000-step Monte Carlo procedure, with Merck Molecular Force Field (MMFF) minimization as implemented in Spartan’16. The unique conformations from this step were next optimized using the PM6 semi-empirical method in Spartan’16. The 10 resulting lowest-energy conformations were further refined with the M06-2X density functional method, the 6- 31 + G(d) basis set, ultrafine integration grid, and GD3 empirical dispersion. Last, the lowest-energy conformation was submitted to M06-2X/6-311++G(d,p) optimization, again with the ultrafine integration grid, GD3 empirical dispersion, and the determination of harmonic vibrational frequencies to verify the identification of a stationary point and for thermal corrections to the electronic energy for the determination of Gibbs free energy. The DFT calculations were all performed with Gaussian 16, Revision A.03 using the default optimization criteria throughout.

### Supplementary Materials

Experimental details on the general experiments and methods, DHP and lignin preparation, organic synthesis of monomeric hydroxystilbenes, synthesis and the structural elucidation (including mechanisms) of the collected dimeric and trimeric compounds, ^1^H and ^13^C NMR data for all compounds, and NMR analysis of lignin.

## Supplementary Materials

### This PDF file includes:

Supplementary Text Figs. S1 to S7 Tables S1 to S3 Legend for data S1 References

## Notes

View/request a protocol for this paper from *Bio-protocol*.
