# Supplementary material for: Biomimetic oxidative copolymerization of hydroxystilbenes and monolignols
Source: Sci Adv. 2023 Mar 8;9(10):eade5519. doi: 10.1126/sciadv.ade5519 (PMC9995074; doi:10.1126/sciadv.ade5519)
Supplement: Supplementary file 1 — Supplementary Text Figs. S1 to S7 Tables S1 to S3 Legend for data S1 References [file sciadv.ade5519_sm.pdf]

Science Advances
AAAS

Supplementary Materials for

Biomimetic oxidative copolymerization of hydroxystilbenes and monolignols

Hoon Kim *et al.*

Corresponding author: Hoon Kim, hoonkim@wisc.edu

Sci. Adv. **9**, eade5519 (2023)
DOI: 10.1126/sciadv.ade5519

The PDF file includes:

Supplementary Text
Figs. S1 to S7
Tables S1 to S3
Legend for data S1
References

Other Supplementary Material for this manuscript includes the following:

Data S1

1. Synthesis of hydroxystilbene compounds

1.1 Hydroxystilbene monomers

Synthesis of hydroxystilbene monomers via an (*E*)-selective Wittig–Horner reaction was performed based on previous work for piceatannol synthesis and the NMR data of the intermediates were compared (fig. S3) (*74*). To generate the common resorcinol moiety of the hydroxystilbenes, 3,5-dihydroxybenzoic acid **1** in acetonitrile was refluxed for 24 h with benzyl bromide and potassium carbonate (K_2CO_3) to produce the tribenzylated ester **2**. The ester was reduced with $LiAlH_4$ (LAH) in tetrahydrofuran (THF) at room temperature for 1.5 h to give 3,5-dibenzylated benzyl alcohol **3**. The benzyl alcohol was brominated to **4** with PBr_3 in acetonitrile. The reaction was stirred at 0 °C (2 h) and then room temperature (2 h). Phosphonation was then performed on the benzyl bromide with triethyl phosphite in toluene and refluxed for 15 h to obtain diethyl phosphonate **5**. To obtain piceatannol **P**, benzylated 3,4-dihydroxybenzaldehyde **6** was coupled with diethyl phosphonate **5** using NaH in THF under reflux to exclusively produce the (*E*)-isomer of benzyl-protected piceatannol **9**. Lastly, debenzylation of the protected piceatannol **9** using $AlCl_3$ and *N*,*N*-dimethylaniline in CH_2Cl_2 at room temperature gave piceatannol. Isorhapontigenin **IS** was synthesized using the same procedure using benzylated vanillin **7**. The debenzylation of the benzyl-protected isorhapontigenin **10** used $AlCl_3$ and anisole in CH_2Cl_2. Resveratrol could be produced from benzylated *p*-hydroxybenzaldehyde **8** but, as noted, it was simply purchased.

1.1.1 Benzyl 3,5-dibenzyloxybenzoate

Yield: quantitative; ^{1}H NMR (acetone-d_6): δ 7.49–7.29 (m, 15H, benzyl groups), 7.28 (d, *J* = 2.37 Hz, 2H, 2 & 6), 6.93 (t, *J* = 2.37 Hz, 1H, 4), 5.35 (s, 2H, 7-benzyl CH_2), 5.15 (s, 4H, 3,5-benzyl CH_2); ^{13}C NMR (acetone-d_6): δ 166.2 (7), 160.9 (3 & 5), 137.9 (3,5-benzyl 1), 137.3 (7-benzyl 1), 133.0 (1), 130.0–128.5 (benzyl groups), 109.1 (2 & 6), 107.5 (4), 70.7 (3,5-benzyl CH_2), 67.2 (7-benzyl CH_2).

1.1.2 3,5-Dibenzyloxybenzyl Alcohol

Yield: 83%; ^{1}H NMR (acetone-d_6): δ 7.46–7.32 (m, 10H, benzyl groups), 6.65 (d, *J* = 2.35 Hz, 2H, 2 & 6), 6.54 (t, *J* = 2.35 Hz, 1H, 4), 5.08 (s, 4H, 3,5-benzyl CH_2), 4.57 (d, *J* = 6.00 Hz, 2H, 7), 4.20 (t, *J* = 6.00 Hz, 1H, 7-OH); ^{13}C NMR (acetone-d$_6$): δ 160.9 (3 & 5), 146.0 (1), 138.4 (3,5-benzyl 1), 129.2 (3,5-benzyl 3 & 5), 128.5 (3,5-benzyl 4), 128.4 (3,5-benzyl 2 & 6), 106.1 (2 & 6), 101.1 (4), 70.3 (3,5-benzyl CH_2), 64.5 (7).

1.1.3 3,5-Dibenzyloxybenzyl Bromide

Yield: quantitative; ^{1}H NMR (acetone-d_6): δ 7.47–7.32 (m, 10H, benzyl groups), 6.74 (d, J = 2.29 Hz, 2H, 2 & 6), 6.63 (t, J = 2.29 Hz, 1H, 4), 5.10 (s, 4H, 3,5-benzyl CH_2), 4.56 (s, 2H, 7); ^{13}C NMR (acetone-d_6): δ 161.0 (3 & 5), 141.2 (1), 138.1 (3,5-benzyl 1), 129.3 (3,5-benzyl 3 & 5), 128.7 (3,5-benzyl 4), 128.5 (3,5-benzyl 2 & 6), 109.1 (2 & 6), 102.6 (4), 70.6 (3,5-benzyl CH_2), 34.3 (7).

1.1.4 Diethyl 3,5-dibenzyloxybenzylphosphonate

Yield: quantitative; ^{1}H NMR (acetone-d_6): δ 7.46–7.31 (m, 10H, benzyl groups), 6.70 (t, J = 2.47 Hz, 2H, 2 & 6), 6.59 (q, J = 2.26 Hz, 1H, 4), 5.11 (s, 4H, 3,5-benzyl CH2), 4.10 (q, J = 7.20 Hz, 4H, $OC\underline{H_2}CH_3$), 3.32 (d, J_{HP} = 22.13 Hz, 2H, 7), 1.21 (t, J = 7.20 Hz, 6H, $OCH_2C\underline{H_3}$); ^{13}C NMR (acetone-d_6): δ 160.8 (d, J_{CP} = 2.99 Hz, 3 & 5), 138.2 (3,5-benzyl 1), 133.9 (d, J_{CP} = 9.31 Hz, 1), 129.2 (3,5-benzyl 3 & 5), 128.6 (3,5-benzyl 4), 128.4 (3,5-benzyl 2 & 6), 109.9 (d, J_{CP} = 6.74 Hz, 2 & 6), 101.7 (d, J_{CP} = 3.55 Hz, 4), 70.5 (3,5-benzyl CH2), 64.3 (d, J_{CP} = 7.07 Hz, $OC\underline{H_2}CH_3$), 33.4 (d, J_{CP} = 138.18 Hz, 7), 16.5 (d, J_{CP} = 6.13 Hz, $OCH_2C\underline{H_3}$),

1.1.5 (*E*)-4,11,13-Tribenzyloxy-3-methoxystilbene

Diethyl 3,5-dibenzyloxybenzylphosphonate and 4-benzyloxy-3-methoxybenzaldehyde were combined to produce the tri-benzylated isorhapontigenin. Yield: 65.5%; ^{1}H NMR (acetone-d_6): δ 7.49–7.39 (m, 15H, benzyl groups), 7.26 (d, J = 1.85 Hz, 1H, 2), 7.20 (d, J = 16.30 Hz, 1H, 7), 7.07 (d, J = 16.30 Hz, 1H, 8), 7.07 (dd, J = 8.30, 1.85 Hz, 1H, 6), 7.01 (d, J = 8.30 Hz, 1H, 5), 6.86 (d, J = 2.20 Hz, 2H, 10 & 14), 6.58 (t, J = 2.20 Hz, 1H,12), 5.13 (s, 4H, 11,13-benzyl CH_2), 5.12 (s, 2H, 4-benzyl CH_2), 3.87 (s, 3H, OMe); ^{13}C NMR (acetone-d_6): δ 161.1 (11 & 13), 151.0 (3), 149.4 (4), 140.8 (9), 138.4 (4-benzyl 1), 138.3 (11,13-benzyl 1), 131.7 (1), 129.9 (7), 129.2 (11,13-benzyl 3 & 5), 129.2 (4-benzyl 3 & 5), 128.6 (11,13-benzyl 4), 128.6 (4-benzyl 4), 128.5 (4-benzyl 2 & 6), 128.4 (11,13-benzyl 2 & 6), 127.4 (8), 120.8 (6), 114.8 (5), 110.5 (2), 106.2 (10 & 14), 101.9 (12), 71.3 (4-benzyl CH_2), 70.5 (11,13-benzyl CH_2), 56.1 (OMe).

1.1.6 (*E*)-3,4,11,13-Tetrabenzyloxystilbene

Diethyl 3,5-dibenzyloxybenzylphosphonate and 3,4-dibenzyloxybenzaldehyde were combined to produce the tetra-benzylated piceatannol. Yield: 85%; ^{1}H NMR (acetone-d_6): δ 7.51–7.33 (m, 20H, benzyl groups), 7.37 (d, J = 1.90 Hz, 1H, 2), 7.18 (d, J = 16.38 Hz, 1H, 7), 7.11 (dd, J = 8.30, 1.90 Hz, 1H, 6), 7.06 (d, J = 8.30 Hz, 1H, 5), 7.04 (d, J = 16.38 Hz, 1H, 8), 6.84 (d, J = 2.18 Hz, 2H, 10 & 14), 6.58 (t, J = 2.18 Hz, 1H, 12), 5.21 (s, 2H, 3-benzyl CH_2), 5.18 (s, 2H, 4-benzyl CH_2), 5.14 (s, 4H, 11,13-benzyl CH_2); ^{13}C NMR (acetone-d_6): δ 161.1 (11 & 13), 150.1 (3), 150.0 (4), 140.7 (9), 138.6 (3-benzyl 1), 138.5 (4-benzyl 1), 138.3 (11,13-benzyl 1), 131.9 (1), 129.8 (7), 129.3 (11,13-benzyl 3 & 5), 129.2 (3- or 4-benzyl 3 & 5), 129.2 (3- or 4-benzyl 3 & 5), 128.6

(11,13-benzyl 4), 128.6 (3- or 4-benzyl 4), 128.5 (3- or 4-benzyl 4), 128.5 (11,13-benzyl 2 & 6), 128.4 (3- or 4-benzyl 2 & 6), 128.3 (3- or 4-benzyl 2 & 6), 127.6 (8), 121.4 (6), 115.6 (5), 113.2 (2), 106.3 (10 & 14), 102.0 (12), 71.5 (3-benzyl CH_2), 71.4 (4-benzyl CH_2), 70.5 (11,13-benzyl CH_2).

1.1.7 Isorhapontigenin

(*E*)-4,11,13-Tribenzyloxy-3-methoxystilbene was debenzylated and a typical reaction with $AlCl_3$-anisole is as follows (fig. S3) (*75, 76*): (*E*)-4,11,13-Tribenzyloxy-3-methoxystilbene (64.6 mg, 0.12 mmol) was dissolved in CH_2C1_2 (2 mL). Anisole (0.16 ml, 1.47 mmol) and powdered $AlCl_3$ (144 mg, 1.08 mmol) was added to the reaction solution at room temperature. Stirred for 30 min, and the reaction was quenched by the addition of 1 M HCl (3 mL). The reaction solution was poured in EtOAc (100 mL) and the organic layer was washed (×3) with water, 5% $NaHCO_3$ solution (×1), and sat. NH_4Cl (×1). The EtOAc solution was dried over anhydrous Na_2SO_4, and the solvent was removed under reduced pressure. The crude product was purified with TLC ($CHCl_3$:EtOAc (v/v) = 1: 1) to obtain isorhapontigenin (12.0 mg, 39%). ^{1}H NMR (acetone-d_6): δ 8.20 (s, 2H, 11,13-OH), 7.12 (s, 1H, 4-OH), 7.21 (d, *J* = 1.26 Hz, 1H, 2), 7.01 (d, *J* = 16.36 Hz, 1H, 7), 7.00 (dd, *J* = 8.12, 1.26 Hz, 1H, 6), 6.92 (d, *J* = 16.36 Hz, 1H, 8), 6.80 (d, *J* = 8.12 Hz, 1H, 5), 6.53 (d, *J* = 1.94 Hz, 2H, 10 & 14), 6.26 (t, *J* = 1.94 Hz, 1H, 12), 3.88 (s, 3H, OMe); ^{13}C NMR (acetone-d_6): δ 159.5 (11 & 13), 148.5 (3), 147.5 (4), 140.8 (9), 130.4 (1), 129.4 (7), 126.9 (8), 121.2 (6), 115.9 (5), 110.0 (2), 105.6 (10 & 14), 102.6 (12), 56.2 (OMe).
^{1}H NMR (DMSO-d_6:pyridine-d_5, 4:1, v/v): δ 9.24 (s, 2H, 11,13-OH), 9.40 (s, 1H, 4-OH), 7.21 (d, *J* = 1.81 Hz, 1H, 2), 7.01 (d, *J* = 16.30 Hz, 1H, 7), 6.99 (dd, *J* = 8.13, 1.81 Hz, 1H, 6), 6.96 (d, *J* = 16.30 Hz, 1H, 8), 6.83 (d, *J* = 8.13 Hz, 1H, 5), 6.54 (d, *J* = 2.00 Hz, 2H, 10 & 14), 6.29 (t, *J* = 2.00 Hz, 1H, 12), 3.81 (s, 3H, OMe); ^{13}C NMR (DMSO-d_6:pyridine-d_5, 4:1, v/v): δ 158.9 (11 & 13), 147.9 (3), 146.8 (4), 139.4 (9), 128.7 (1), 128.3 (7), 126.0 (8), 120.2 (6), 115.6 (5), 109.9 (2), 104.4 (10 & 14), 101.9 (12), 55.6 (OMe).

1.1.8 Piceatannol

(*E*)-3,4,11,13-Tetrabenzyloxystilbene was debenzylated by using *N,N*-dimethylaniline (*76*). Yield: 45%; ^{1}H NMR (acetone-d_6): δ 8.19 (s, 2H, 11,13-OH), 8.04 (s, 1H, 4-OH), 7.91 (s, 1H, 3-OH), 7.06 (d, *J* = 1.89 Hz, 1H, 2), 6.94 (d, *J* = 16.20 Hz, 1H, 7), 6.90 (dd, *J* = 8.00, 1.89 Hz, 1H, 6), 6.81 (d, *J* = 16.20 Hz, 1H, 8), 6.80 (d, *J* = 8.00 Hz, 1H, 5), 6.52 (d, *J* = 2.06 Hz, 2H, 10 & 14), 6.25 (t, *J* = 1.86 Hz, 1H, 12); ^{13}C NMR (acetone-d_6): δ 159.5 (11 & 13), 146.1 (3), 146.0 (4), 140.8 (9), 130.6 (1), 129.3 (7), 126.8 (8), 119.9 (6), 116.2 (5), 113.8 (2), 105.6 (10 & 14), 102.6 (12).

^1H NMR (DMSO-d_6:pyridine-d_5, 4:1, v/v): δ 9.44 (s, 2H, 11,13-OH), 9.40 (br s, 1H, 4-OH), 9.24 (br s, 1H, 3-OH), 7.09 (d, *J* = 2.02 Hz, 1H, 2), 6.96 (d, *J* = 16.24 Hz, 1H, 7), 6.88 (dd, *J* = 8.22, 2.02 Hz, 1H, 6), 6.82 (d, *J* = 16.24 Hz, 1H, 8), 6.81 (d, *J* = 8.22 Hz, 1H, 5), 6.52 (d, *J* = 2.10 Hz, 2H, 10 & 14), 6.28 (t, *J* = 2.10 Hz, 1H, 12); ^{13}C NMR (DMSO-d_6:pyridine-d_5, 4:1, v/v): δ 158.7 (11 & 13), 145.7 (3), 145.6 (4), 139.4 (9), 128.7 (1), 128.3 (7), 125.6 (8), 118.6 (6), 115.8 (5), 113.4 (2), 104.4 (10 & 14), 101.9 (12).

1.1.9 Resveratrol

Resveratrol **R** was not chemically synthesized. The NMR data was collected using the purchased compound. ^1H NMR (acetone-d_6): δ 8.47 (s, 1H, 4-OH), 8.22 (s, 2H, 11,13-OH), 7.41 (d, *J* = 8.55 Hz, 2H, 2 & 6), 7.01 (d, *J* = 16.31 Hz, 1H, 7), 6.89 (d, *J* = 16.31 Hz, 1H, 8), 6.83 (d, *J* = 8.55 Hz, 1H, 3 & 5), 6.55 (d, *J* = 2.10 Hz, 2H, 10 & 14), 6.28 (t, *J* = 2.10 Hz, 1H, 12); ^{13}C NMR (acetone-d_6): δ 159.4 (11 & 13), 158.0 (4), 140.8 (9), 129.8 (1), 129.0 (7), 128.7 (2 & 6), 126.7 (8), 116.3 (3 & 5), 105.6 (10 & 14), 102.5 (12).

^1H NMR (DMSO-d_6:pyridine-d_5, 4:1, v/v): δ 9.79 (s, 1H, 4-OH), 9.47 (s, 2H, 11,13-OH), 7.41 (d, *J* = 8.54 Hz, 2H, 2 & 6), 7.01 (d, *J* = 16.40 Hz, 1H, 7), 6.90 (d, *J* = 16.40 Hz, 1H, 8), 6.83 (d, *J* = 8.54 Hz, 1H, 3 & 5), 6.54 (d, *J* = 2.00 Hz, 2H, 10 & 14), 6.29 (t, *J* = 2.00 Hz, 1H, 12); ^{13}C NMR (DMSO-d_6:pyridine-d_5, 4:1, v/v): δ 158.8 (11 & 13), 157.4 (4), 139.5 (9), 128.2 (1), 128.0 (7), 127.9 (2 & 6), 125.8 (8), 115.6 (3 & 5), 104.5 (10 & 14), 102.0 (12).

1.2 Hydroxystilbene homo-dimerization and cross-coupling with monolignols – dimers and trimers

Oxidative radical coupling of piceatannol or resveratrol with sinapyl alcohol using various oxidative reagents, such as horseradish peroxidase (HRP), silver(I) oxide (Ag_2O), silver(I) acetate (AgOAc), manganese(IV) oxide (MnO_2), or ferric chloride ($FeCl_3$•$6H_2O$), were carried out to synthesize homo- and cross-coupled dimers and trimers. All reactions were performed at room temperature, and the reaction time was varied depending on the reaction. Reaction monitoring was achieved via aluminum-backed silica-gel TLC. Each collected crude product was separated by preparative TLC using $CHCl_3$/MeOH/formic acid (85:15:3, v/v/v) (*77*). Most products were dark solids.

1.2.1 Piceatannol dimers and trimers

Radically coupled products of piceatannol were prepared using various reagents in different solvent systems. The HRP reaction produced dimers $\mathbf{PP_A}$ and $\mathbf{PP_C}$ (Fig. 1D). Piceatannol (300 mg, 1.23 mmol) was dissolved in 10 mL of acetone and poured into acetate buffer (pH 5, 100 mL). HRP (27.4 mg) and 30% H_2O_2 (576.4 μL) were added into the reaction solution and stirred

at room temperature for 20 min. The product solution was poured into EtOAc (100 mL), and the organic layer was washed with saturated NH_4Cl and water. The organic solution was dried over anhydrous $MgSO_4$ and evaporated after filtration. The components in the crude product were relatively quantified by ^1H NMR – $\mathbf{PP_A}$ (47.2%) and $\mathbf{PP_C}$ (52.8%). MnO_2 (or Ag_2O) produced only $\mathbf{PP_A}$. Piceatannol (181.4 mg) was dissolved in 20 mL of acetone and stirred at room temperature for 3 h after an excess of MnO_2 was added. The crude product solution was filtered using a sintered glass filter. The acetone was evaporated, and the crude product was separated on TLC to collect $\mathbf{PP_A}$ (44.7 mg, 24.6%). Oxidation with $FeCl_3$•$6H_2O$ produced only $\mathbf{PP_C}$. Piceatannol (179.0 mg) was dissolved in 20 mL of acetone and stirred at room temperature for 3 h after $FeCl_3$•$6H_2O$ was added. The crude product solution was poured into EtOAc (100 mL), and the organic layer was washed with sat. NH_4Cl and water. The organic solution was dried over anhydrous $MgSO_4$ and evaporated after filtration. The crude product was separated by TLC to collect $\mathbf{PP_C}$ (10.3 mg, 5.8%). AgOAc produced dimers $\mathbf{PP_A}$ and $\mathbf{PP_D}$ and also different types of trimers depending on the solvent system (Fig. 1, D and E). The reaction condition and work-up procedures were similar to that for the MnO_2 reaction. For the AgOAc reaction in MeOH, 200 mg of piceatannol was used, and the reaction time was 1 h. $\mathbf{PP_A}$ (43.0 mg, 21.5%), $\mathbf{PP_D}$ (16.0 mg, 8.0%), and $\mathbf{PPP_{H\text{-}A}}$ (22.4 mg, 11.2%) were obtained after TLC separation. The reaction in EtOAc was stirred overnight and produced one different trimer $\mathbf{PPP_{H\text{-}B}}$ (13.9 mg, 9.3%) along with $\mathbf{PP_A}$ (65.9 mg, 43.9%) and $\mathbf{PP_D}$ (6.2 mg, 4.1%) from 150 mg of piceatannol.

1.2.1.1 $\mathbf{PP_A}$ (Cassigarol E)

(*E*)-4-(3-(3,5-Dihydroxyphenyl)-7-(3,5-dihydroxystyryl)-2,3-dihydrobenzo[*b*][1,4]dioxin-2-yl)benzene-1,2-diol. ^1H NMR (MeOH-d_4): δ 7.12 (d, *J* = 2.0 Hz, 1H, 2′), 7.05 (dd, *J* = 8.5, 2.0 Hz, 1H, 6′), 6.95 (d, *J* = 16.9 Hz, 1H, 7′), 6.93 (d, *J* = 8.5 Hz, 1H, 5′), 6.84 (d, *J* = 16.9 Hz, 1H, 8′), 6.65 (br s, 1H, 2), 6.64 (d, *J* = 8.8 Hz, 1H, 5), 6.47 (d, *J* = 2.1 Hz, 2H, 10′ & 14′), 6.46 (dd, *J* = 8.8, 2.0 Hz, 1H, 6), 6.18 (t, *J* = 2.1 Hz, 1H, 12′), 6.15 (t, *J* = 2.2 Hz, 1H, 12), 6.09 (d, *J* = 2.2 Hz, 2H, 10 & 14), 4.71 (d, *J* = 8.0 Hz, 1H, 7), 4.69 (d, *J* = 8.0 Hz, 1H, 8); ^{13}C NMR (MeOH-d_4): δ 159.6 (11′ & 13′), 159.2 (11 & 13), 146.6 (4), 146.0 (3), 145.4 (3′), 144.9 (4′), 141.0 (9′), 140.1 (9), 132.5 (1′), 129.4 (1), 129.0 (7′), 128.3 (8′), 121.0 (6′), 120.7 (6), 118.1 (5′), 115.9 (2), 115.7 (5), 115.6 (2′), 107.4 (10 & 14), 105.9 (10′ & 14′), 103.5 (12), 102.8 (12′), 82.2 (8), 81.7 (7). ^1H NMR (DMSO-d_6:pyridine-d_5, 4:1, v/v): δ 7.24 (d, *J* = 1.8 Hz, 1H, 2′), 7.11 (dd, *J* = 8.4, 1.8 Hz, 1H, 6′), 7.05 (d, *J* = 16.3 Hz, 1H, 7′), 6.99 (d, *J* = 16.3 Hz, 1H, 8′), 6.95 (d, *J* = 8.4 Hz, 1H, 5′), 6.80 (d, *J* = 1.9 Hz, 1H, 2), 6.69 (d, *J* = 8.1 Hz, 1H, 5), 6.56 (d, *J* = 1.6 Hz, 2H, 10′ & 14′), 6.55 (dd, *J* = 8.1, 1.9 Hz, 1H, 6), 6.31 (t, *J* = 2.0 Hz, 1H, 12′), 6.25 (t, *J* = 2.0 Hz, 1H, 12), 6.22 (d, *J* = 2.0 Hz, 2H, 10 & 14), 4.95 (d, *J* = 8.0 Hz, 1H, 8), 4.93 (d, *J* = 8.0 Hz, 1H, 7); ^{13}C NMR (DMSO-d_6:pyridine-d_5, 4:1, v/v): δ 158.8 (11′ & 13′), 158.2 (11 & 13), 145.7 (4), 145.1 (3), 144.0

(3′), 143.5 (4′), 139.1 (9′), 138.5 (9), 130.7 (1′), 127.5 (7′), 127.4 (1 & 8′), 119.9 (6′), 119.3 (6), 116.9 (5′), 115.4 (2), 115.2 (5), 114.5 (2′), 106.2 (10 & 14), 104.6 (10′ & 14′), 102.8 (12), 102.2 (12′), 79.8 (8), 79.4 (7). LC-QTOF-MS: m/z calculated for $C_{28}H_{21}O_8$ $[M – H]^–$ 485.1242, found 485.1244.

1.2.1.2 PP_C (Scirpusin B)

(*E*)-4-(2-(2-(3,4-Dihydroxyphenyl)-3-(3,5-dihydroxyphenyl)-6-hydroxy-2,3-dihydrobenzofuran-4-yl)vinyl)benzene-1,2-diol. ^1H NMR (MeOH-d_4): δ 6.76 (d, *J* = 16.3 Hz, 1H, 7′), 6.74 (br s, 1H, 2), 6.73 (d, *J* = 8.3 Hz, 1H, 5), 6.69 (d, *J* = 1.9 Hz, 1H, 2′), 6.63 (d, *J* = 8.3 Hz, 1H, 6 & 5′), 6.61 (d, *J* = 1.9 Hz, 1H, 14′), 6.57 (dd, *J* = 8.3, 1.9 Hz, 1H, 6′), 6.53 (d, *J* = 16.3 Hz, 1H, 8′), 6.24 (d, *J* = 1.9 Hz, 1H, 12′), 6.16 (t, *J* = 2.0 Hz, 1H,12), 6.14 (d, *J* = 2.0 Hz, 2H, 10 & 14), 5.27 (d, *J* = 5.9 Hz, 1H, 7), 4.32 (d, *J* = 5.9 Hz, 1H, 8); ^{13}C NMR (MeOH-d_4): δ 162.9 (11′), 160.0 (11 & 13), 159.8 (13′), 147.7 (9), 146.6 (4′), 146.5 (3), 146.4 (4), 146.3 (3′), 137.0 (9′), 134.9 (1), 130.9 (1′), 130.9 (7′), 123.6 (8′), 120.0 (6′), 119.8 (10′), 118.4 (6), 116.3 (5′), 116.2 (5), 114.0 (2′), 113.6 (2), 107.3 (10 & 14), 104.4 (14′), 102.2 (12), 96.8 (12′), 94.9 (7), 58.1 (8).

^1H NMR (DMSO-d_6:pyridine-d_5, 4:1, v/v): δ 6.87 (d, *J* = 16.4 Hz, 1H, 7′), 6.86 (d, *J* = 1.9 Hz, 1H, 2′), 6.83 (d, *J* = 2.0 Hz, 1H, 2), 6.78 (d, *J* = 8.1 Hz, 1H, 5), 6.73 (d, *J* = 8.2 Hz, 1H, 5′), 6.72 (d, *J* = 1.8 Hz, 1H, 14′), 6.68 (dd, *J* = 8.2, 1.9 Hz, 1H, 6′), 6.64 (d, *J* = 16.4 Hz, 1H, 8′), 6.62 (dd, *J* = 8.1, 2.0 Hz, 1H, 6), 6.36 (d, *J* = 1.8 Hz, 1H, 12′), 6.22 (t, *J* = 2.0 Hz, 1H, 12), 6.18 (d, *J* = 2.0 Hz, 2H, 10 & 14), 5.34 (d, *J* = 4.4 Hz, 1H, 7), 4.44 (d, *J* = 4.4 Hz, 1H, 8); ^{13}C NMR (DMSO-d_6:pyridine-d_5, 4:1, v/v): δ 161.1 (11′), 158.9 (11 & 13), 158.7 (13′), 146.5 (9), 145.9 (4′), 145.6 (3), 145.5 (3′), 145.5 (4), 135.1 (9′), 133.0 (1), 129.7 (7′), 128.6 (1′), 121.9 (8′), 118.3 (6′), 118.2 (10′), 116.7 (6), 115.7 (5′), 115.5 (5), 113.8 (2′), 112.7 (2), 105.4 (10 & 14), 103.3 (14′), 101.2 (12), 96.0 (12′), 92.6 (7), 55.8 (8). LC-QTOF-MS: m/z calculated for $C_{28}H_{21}O_8$ $[M – H]^–$ 485.1242, found 485.1240.

1.2.1.3 PP_D (Maackin A)

(*E*)-4-(3-(3,5-Dihydroxyphenyl)-5-(3,5-dihydroxystyryl)-7-hydroxy-2,3-dihydrobenzofuran-2-yl)benzene-1,2-diol. ^1H NMR (MeOH-d_4): δ 6.92 (d, *J* = 1.2 Hz, 1H, 2′), 6.89 (d, *J* = 16.2 Hz, 1H, 7′), 6.81 (d, *J* = 2.0 Hz, 1H, 2), 6.75 (d, *J* = 8.1 Hz, 1H, 5), 6.73 (d, *J* = 16.2 Hz, 1H, 8′), 6.68 (dd, *J* = 8.1, 2.0 Hz, 1H, 6), 6.67 (br s, 1H, 6′), 6.40 (d, *J* = 2.2 Hz, 2H, 10′ & 14′), 6.17 (t, *J* = 2.2 Hz, 1H, 12), 6.13 (t, *J* = 2.2 Hz, 1H, 12′), 6.13 (d, *J* = 2.2 Hz, 2H, 10 & 14), 5.34 (d, *J* = 8.4 Hz, 1H, 7), 4.37 (d, *J* = 8.4 Hz, 1H, 8); ^{13}C NMR (MeOH-d_4): δ 159.9 (11 & 13), 159.6 (11′ & 13′), 148.6 (4′), 146.6 (4), 146.4 (3), 145.5 (9), 142.4 (3′), 141.1 (9′), 133.6 (1), 133.2 (5′), 133.1 (1′), 129.6 (7′), 127.4 (8′), 119.1 (6), 116.2 (5), 115.9 (6′), 114.7 (2′), 114.2 (2), 107.7 (10 & 14), 105.8 (10′ & 14′), 102.7 (12′), 102.4 (12), 95.2 (7), 59.4 (8).

^{1}H NMR (DMSO-d_6:pyridine-d_5, 4:1, v/v): δ 7.03 (br s, 1H, 2′), 6.96 (d, *J* = 16.3 Hz, 1H, 7′), 6.90 (d, *J* = 2.0 Hz, 1H, 2), 6.80 (d, *J* = 16.3 Hz, 1H, 8′), 6.79 (d, *J* = 8.1 Hz, 1H, 5), 6.73 (br s, 1H, 6′), 6.67 (dd, *J* = 8.1, 2.0 Hz, 1H, 6), 6.50 (d, *J* = 1.8 Hz, 2H, 10′ & 14′), 6.28 (t, *J* = 1.8 Hz, 1H, 12′), 6.27 (t, *J* = 2.1 Hz, 1H, 12), 6.18 (d, *J* = 2.1 Hz, 2H, 10 & 14), 5.39 (d, *J* = 7.7 Hz, 1H, 7), 4.45 (d, *J* = 7.7 Hz, 1H, 8); ^{13}C NMR (DMSO-d_6:pyridine-d_5, 4:1, v/v): δ 158.9 (11 & 13), 158.7 (11′ & 13′), 147.0 (4′), 145.8 (4), 145.6 (3), 144.2 (9), 141.5 (3′), 139.3 (9′), 132.0 (1), 131.5 (5′), 131.1 (1′), 128.3 (7′), 126.2 (8′), 117.7 (6), 115.5 (5), 114.3 (2′), 114.1 (6′), 113.7 (2), 106.1 (10 & 14), 104.5 (10′ & 14′), 102.0 (12′), 101.4 (12), 92.8 (7), 56.8 (8). LC-QTOF-MS: m/z calculated for $C_{28}H_{21}O_8$ [M – H]$^-$ 485.1242, found 485.1239.

1.2.1.4 PPP$_{\mathbf{H\text{-}A}}$

(*E*)-4-(2-(3-(3,5-Dihydroxyphenyl)-2-(3-(3,4-dihydroxyphenyl)-2-(3,5-dihydroxyphenyl)-2,3-dihydrobenzo[*b*][1,4]dioxin-6-yl)-6-hydroxy-2,3-dihydrobenzofuran-4-yl)vinyl)benzene-1,2-diol (see table S1). LC-QTOF-MS: m/z calculated for $C_{42}H_{31}O_{12}$ [M – H]$^-$ 727.1821, found 727.1823.

1.2.1.5 PPP$_{\mathbf{H\text{-}B}}$

(*E*)-5,5'-(3'-(3,4-Dihydroxyphenyl)-7-(3,5-dihydroxystyryl)-2,2',3,3'-tetrahydro-[2,6'-bibenzo[*b*][1,4]dioxine]-2',3-diyl)bis(benzene-1,3-diol) (See table S2). LC-QTOF-MS: m/z calculated for $C_{42}H_{31}O_{12}$ [M – H]$^-$ 727.1821, found 727.1814.

1.2.2 Resveratrol dimers

Resveratrol dimers were also prepared using various catalysts, HRP, $FeCl_3•6H_2O$, and AgOAc. The HRP reaction of resveratrol (227 mg) produced dimers **RR$_{\mathbf{B}}$** (6.2 mg, 2.7%), **RR$_{\mathbf{D}}$** (96.3 mg, 42.4%), and **RR$_{\mathbf{D\text{-}oxi}}$** (2.4 mg, 1.1%) after TLC separation. Resveratrol was initially dissolved in 10 mL of acetone and poured into 20 mL of H_2O without using a buffer. The reaction proceeded in the same manner as for the piceatannol reaction. The overnight reaction of AgOAc in EtOAc produced **RR$_{\mathbf{D}}$** (39.8 mg, 20.0%) exclusively from 199.0 mg of resveratrol. **RR$_{\mathbf{C}}$** (47.4 mg, 24.1%), **RR$_{\mathbf{C\text{-}1}}$** (8.4 mg, 4.3%), and **RR$_{\mathbf{F}}$** (7.5 mg, 3.8%) were obtained from 196.7 mg of resveratrol under $FeCl_3•6H_2O$ in acetone and the reaction was allowed to stir overnight.

1.2.2.1 RR$_{\mathbf{B}}$ (Labruscol)

(*E*)-5-(4-(1-(3,5-Dihydroxyphenyl)-2-hydroxy-2-(4-hydroxyphenyl)ethoxy)styryl)benzene-1,3-diol. ^{1}H NMR (MeOH-d_4): δ 7.32 (d, *J* = 8.8 Hz, 2H, 2′ & 6′), 7.01 (d, *J* = 8.5 Hz, 2H, 2 & 6), 6.91 (d, *J* = 16.3 Hz, 1H, 7′), 6.88 (d, *J* = 8.8 Hz, 2H, 3′ & 5′), 6.78 (d, *J* = 16.3 Hz, 1H, 8′), 6.64 (d, *J* = 8.5 Hz, 2H, 3 & 5), 6.41 (d, *J* = 2.2 Hz, 2H, 10′ & 14′), 6.13 (t, *J* = 2.2 Hz, 1H, 12′), 6.09 (d, *J* = 2.2 Hz, 2H, 10 & 14), 6.05 (t, *J* = 2.2 Hz, 1H, 12), 4.97 (d, *J* = 7.4 Hz, 1H, 8), 4.78 (d, *J* =

7.4 Hz, 1H, 7); ^{13}C NMR (MeOH-d_4): δ 159.7 (11′ & 13′), 159.3 (4′), 159.2 (11 & 13), 158.0 (4), 141.9 (9), 141.1 (9′), 132.5 (1), 131.6 (1′), 129.7 (2 & 6), 129.2 (7′), 128.4 (2′ & 6′), 127.8 (8′), 117.4 (3′ & 5′), 115.7 (3 & 5), 107.2 (10 & 14), 105.8 (10′ & 14′), 102.9 (12), 102.7 (12′), 86.4 (8), 79.0 (7).

^{1}H NMR (DMSO-d_6:pyridine-d_5, 4:1, v/v): δ 7.36 (d, *J* = 8.7 Hz, 2H, 2′ & 6′), 7.08 (d, *J* = 8.5 Hz, 2H, 2 & 6), 6.93 (d, *J* = 16.3 Hz, 1H, 7′), 6.88 (d, *J* = 8.7 Hz, 2H, 3′ & 5′), 6.87 (d, *J* = 16.3 Hz, 1H, 8′), 6.67 (d, *J* = 8.5 Hz, 2H, 3 & 5), 6.51 (d, *J* = 2.0 Hz, 2H, 10′ & 14′), 6.28 (t, *J* = 2.0 Hz, 1H, 12′), 6.20 (d, *J* = 2.0 Hz, 2H, 10 & 14), 6.15 (t, *J* = 2.0 Hz, 1H, 12), 5.71 (br s, 1H, 7-OH), 5.12 (d, *J* = 6.5 Hz, 1H, 8), 4.81 (d, *J* = 6.5 Hz, 1H, 7); ^{13}C NMR (DMSO-d_6:pyridine-d_5, 4:1, v/v): δ 158.7 (11′ & 13′), 158.0 (11 & 13), 157.9 (4′), 156.4 (4), 140.7 (9), 139.2 (9′), 132.0 (1), 129.5 (1′), 128.4 (2 & 6), 128.0 (7′), 127.5 (2′ & 6′), 126.6 (8′), 116.0 (3′ & 5′), 114.4 (3 & 5), 105.8 (10 & 14), 104.5 (10′ & 14′), 102.1 (12′), 101.9 (12), 84.3 (8), 76.3 (7). LC-QTOF-MS: m/z calculated for $C_{28}H_{23}O_7$ $[M - H]^-$ 471.1449, found 471.1451.

1.2.2.2 RR$_C$ (ε-Viniferin)

(*E*)-5-(6-Hydroxy-2-(4-hydroxyphenyl)-4-(4-hydroxystyryl)-2,3-dihydrobenzofuran-3-yl)benzene-1,3-diol. ^{1}H NMR (MeOH-d_4): δ 7.14 (d, *J* = 8.5 Hz, 2H, 2 & 6), 7.03 (d, *J* = 8.6 Hz, 2H, 2′ & 6′), 6.82 (d, *J* = 16.3 Hz, 1H, 7′), 6.76 (d, *J* = 8.5 Hz, 2H, 3 & 5), 6.65 (d, *J* = 8.6 Hz, 2H, 3′ & 5′), 6.63 (d, *J* = 2.0 Hz, 1H, 14′), 6.57 (d, *J* = 16.3 Hz, 1H, 8′), 6.25 (d, *J* = 2.0 Hz, 1H, 12′), 6.18 (t, *J* = 2.1 Hz, 1H, 12), 6.16 (d, *J* = 2.1 Hz, 2H, 10 & 14), 5.36 (d, *J* = 6.6 Hz, 1H, 7), 4.35 (d, *J* = 6.6 Hz, 1H, 8); ^{13}C NMR (MeOH-d_4): δ 162.7 (11′) 160.0 (11 & 13), 159.7 (13′), 158.5 (4), 158.3 (4′), 147.3 (9), 136.8 (9′), 133.8 (1), 130.3 (1′), 130.3 (7′), 128.7 (2′ & 6′), 128.2 (2 & 6), 123.6 (8′), 120.0 (10′), 116.3 (3′ & 5′), 116.2 (3 & 5), 107.4 (10 & 14), 104.3 (14′), 102.1 (12), 96.8 (12′), 94.8 (7), 58.2 (8).

^{1}H NMR (DMSO-d_6:pyridine-d_5, 4:1, v/v): δ 7.18 (d, *J* = 8.9 Hz, 2H, 2′ & 6′), 7.16 (d, *J* = 8.5 Hz, 2H, 2 & 6), 6.92 (d, *J* = 16.4 Hz, 1H, 7′), 6.81 (d, *J* = 8.5 Hz, 2H, 3 & 5), 6.74 (d, *J* = 2.3 Hz, 1H, 14′), 6.74 (d, *J* = 8.9 Hz, 2H, 3′ & 5′), 6.71 (d, *J* = 16.4 Hz, 1H, 8′), 6.38 (br s, 1H, 12′), 6.23 (br s, 3H, 12, 10 & 14), 5.43 (d, *J* = 5.1 Hz, 1H, 7), 4.50 (d, *J* = 5.1 Hz, 1H, 8);

^{13}C NMR (DMSO-d_6:pyridine-d_5, 4:1, v/v): δ 160.9 (11′) 158.9 (11 & 13), 158.7 (13′), 157.5 (4′), 157.4 (4), 146.3 (9), 135.0 (9′), 131.9 (1), 129.1 (7′), 128.1 (1′), 127.8 (2′ & 6′), 127.1 (2 & 6), 122.1 (8′), 118.6 (10′), 115.6 (3′ & 5′), 115.3 (3 & 5), 105.6 (10 & 14), 103.3 (14′), 101.2 (12), 96.1 (12′), 92.5 (7), 55.4 (8). LC-QTOF-MS: m/z calculated for $C_{28}H_{21}O_6$ $[M - H]^-$ 453.1344, found 453.1345.

1.2.2.3 RR$_{C-1}$ (ω-Viniferin)

5-((2*S*,3*R*)-6-Hydroxy-2-(4-hydroxyphenyl)-4-((*E*)-4-hydroxystyryl)-2,3-dihydrobenzofuran-3-yl)benzene-1,3-diol. ^{1}H NMR (MeOH-d_4): δ 7.12 (d, *J* = 8.6 Hz, 2H, 2′ & 6′), 6.96 (d, *J* = 8.6 Hz, 2H, 2 & 6), 6.86 (d, *J* = 16.5 Hz, 1H, 7′), 6.66 (d, *J* = 8.6 Hz, 2H, 3′ & 5′), 6.65 (d, *J* = 16.5 Hz, 1H, 8′), 6.63 (d, *J* = 2.0 Hz, 1H, 14′), 6.55 (d, *J* = 8.6 Hz, 2H, 3 & 5), 6.30 (d, *J* = 2.0 Hz, 1H, 12′), 5.92 (t, *J* = 2.0 Hz, 1H, 12), 5.82 (d, *J* = 8.2 Hz, 1H, 7), 5.75 (d, *J* = 2.0 Hz, 2H, 10 & 14), 4.58 (d, *J* = 8.2 Hz, 1H, 8); ^{13}C NMR (MeOH-d_4): δ 162.7 (11′) 159.5 (13′), 158.8 (11 & 13), 158.5 (4′), 157.6 (4), 143.8 (9), 136.9 (9′), 130.8 (7′), 130.3 (1′), 129.7 (1), 129.2 (2 & 6), 128.8 (2′ & 6′), 123.9 (8′), 121.7 (10′), 116.4 (3′ & 5′), 115.2 (3 & 5), 109.1 (10 & 14), 105.1 (14′), 101.7 (12), 97.2 (12′), 90.9 (7), 53.6 (8).
^{1}H NMR (DMSO-d_6:pyridine-d_5, 4:1, v/v): δ 7.22 (d, *J* = 8.7 Hz, 2H, 2′ & 6′), 7.01 (d, *J* = 8.6 Hz, 2H, 2 & 6), 6.96 (d, *J* = 16.5 Hz, 1H, 7′), 6.76 (d, *J* = 8.7 Hz, 2H, 3′ & 5′), 6.75 (d, *J* = 2.0 Hz, 1H, 14′), 6.74 (d, *J* = 16.5 Hz, 1H, 8′), 6.60 (d, *J* = 8.6 Hz, 2H, 3 & 5), 6.43 (d, *J* = 2.0 Hz, 1H, 12′), 5.95 (t, *J* = 2.0 Hz, 1H, 12), 5.84 (d, *J* = 8.0 Hz, 1H, 7), 5.81 (d, *J* = 2.0 Hz, 2H, 10 & 14), 4.67 (d, *J* = 8.0 Hz, 1H, 8); ^{13}C NMR (DMSO-d_6:pyridine-d_5, 4:1, v/v): δ 160.9 (11′), 158.3 (13′), 157.8 (11 & 13), 157.6 (4′), 156.4 (4), 142.3 (9), 134.8 (9′), 129.4 (7′), 128.0 (1′), 127.9 (2 & 6), 127.8 (2′ & 6′), 127.5 (1), 122.3 (8′), 120.5 (10′), 115.6 (3′ & 5′), 114.3 (3 & 5), 107.2 (10 & 14), 104.0 (14′), 100.9 (12), 96.5 (12′), 89.0 (7), 51.3 (8). LC-QTOF-MS: m/z calculated for $C_{28}H_{21}O_6$ $[M – H]^–$ 453.1344, found 453.1348.

1.2.2.4 RR$_D$ (δ-Viniferin)

(*E*)-5-(2-(3-(3,5-Dihydroxyphenyl)-2-(4-hydroxyphenyl)-2,3-dihydrobenzofuran-5-yl)vinyl)benzene-1,3-diol. ^{1}H NMR (MeOH-d_4): δ 7.34 (dd, *J* = 8.3, 1.5 Hz, 1H, 6′), 7.17 (br s, 1H, 2′), 7.16 (d, *J* = 8.6 Hz, 2H, 2 & 6), 6.96 (d, *J* = 16.3 Hz, 1H, 7′), 6.82 (d, *J* = 8.3 Hz, 1H, 5′), 6.78 (d, *J* = 8.6 Hz, 2H, 3 & 5), 6.77 (d, *J* = 16.3 Hz, 1H, 8′), 6.44 (d, *J* = 2.1 Hz, 2H, 10′ & 14′), 6.21 (t, *J* = 2.2 Hz, 1H, 12), 6.16 (t, *J* = 2.1 Hz, 1H, 12′), 6.13 (d, *J* = 2.2 Hz, 2H, 10 & 14), 5.37 (d, *J* = 8.5 Hz, 1H, 7), 4.39 (d, *J* = 8.5 Hz, 1H, 8); ^{13}C NMR (MeOH-d_4): δ 160.9 (4′), 159.8 (11 & 13), 159.5 (11′ & 13′), 158.6 (4), 145.3 (9), 141.1 (9′), 132.7 (1), 132.3 (1′), 132.2 (3′), 129.4 (7′), 128.7 (2 & 6), 128.7 (6′), 127.3 (8′), 124.1 (2′), 116.3 (3 & 5), 110.3 (5′), 107.7 (10 & 14), 105.8 (10′ & 14′), 102.6 (12′), 102.4 (12), 94.8 (7), 58.6 (8).
^{1}H NMR (DMSO-d_6:pyridine-d_5, 4:1, v/v): δ 7.42 (dd, *J* = 8.3, 1.2 Hz, 1H, 6′), 7.27 (br s, 1H, 2′), 7.21 (d, *J* = 8.6 Hz, 2H, 2 & 6), 7.05 (d, *J* = 16.2 Hz, 1H, 7′), 6.90 (d, *J* = 8.3 Hz, 1H, 5′), 6.90 (d, *J* = 16.2 Hz, 1H, 8′), 6.83 (d, *J* = 8.6 Hz, 2H, 3 & 5), 6.53 (d, *J* = 2.0 Hz, 2H, 10′ & 14′), 6.30 (t, *J* = 2.0 Hz, 1H, 12′), 6.29 (t, *J* = 2.1 Hz, 1H, 12), 6.19 (d, *J* = 2.1 Hz, 2H, 10 & 14), 5.46 (d, *J* = 7.9 Hz, 1H, 7), 4.50 (d, *J* = 7.9 Hz, 1H, 8); ^{13}C NMR (DMSO-d_6:pyridine-d_5, 4:1, v/v): δ 159.1 (4′), 159.0 (11 & 13), 158.7 (11′ & 13′), 157.7 (4), 143.9 (9), 139.3 (9′), 131.3 (3′), 130.5 (1), 130.5 (1′), 127.9 (2, 6 & 7′), 127.7 (6′), 126.4 (8′), 123.0 (2′), 115.4 (3 & 5), 109.4 (5′), 106.2 (10 & 14),

104.6 (10′ & 14′), 102.1 (12′), 101.6 (12), 92.8 (7), 56.0 (8). LC-QTOF-MS: m/z calculated for $C_{28}H_{21}O_6$ [M – H]⁻ 453.1344, found 453.1341.

1.2.2.5 RR$_{D\text{-}oxi}$ (δ-Viniferin, oxidized form)

3-(3,5-Dihydroxyphenyl)-2-(4-hydroxyphenyl)-2,3-dihydrobenzofuran-5-carbaldehyde. ^{1}H NMR (MeOH-*d*$_4$): δ 9.78 (s, 1H, 7′), 7.83 (ddd, *J* = 8.4, 1.8, 0.4 Hz, 1H, 6′), 7.58 (dd, *J* = 1.8, 1.4 Hz, 1H, 2′), 7.17 (d, *J* = 8.6 Hz, 2H, 2 & 6), 7.03 (d, *J* = 8.4 Hz, 1H, 5′), 6.78 (d, *J* = 8.6 Hz, 2H, 3 & 5), 6.18 (t, *J* = 2.2 Hz, 1H, 12), 6.08 (d, *J* = 2.2 Hz, 2H, 10 & 14), 5.53 (d, *J* = 8.4 Hz, 1H, 7), 4.47 (d, *J* = 8.4 Hz, 1H, 8); ^{13}C NMR (MeOH-*d*$_4$): δ 192.7 (7′), 166.7 (4′), 160.1 (11 & 13), 159.0 (4), 144.6 (9), 134.0 (6′), 133.6 (3′), 132.4 (1′), 132.0 (1), 128.7 (2 & 6), 128.1 (2′), 116.4 (3 & 5), 110.9 (5′), 107.5 (10 & 14), 102.6 (12), 96.0 (7), 57.8 (8).
^{1}H NMR (DMSO-*d*$_6$:pyridine-*d*$_5$, 4:1, v/v): δ 9.83 (s, 1H, 7′), 9.53 (br s, 2H, 11- & 13-OH), 7.83 (dd, *J* = 8.3, 1.4 Hz, 1H, 6′), 7.56 (br s, 1H, 2′), 7.23 (d, *J* = 8.5 Hz, 2H, 2 & 6), 7.08 (d, *J* = 8.3 Hz, 1H, 5′), 6.83 (d, *J* = 8.5 Hz, 2H, 3 & 5), 6.29 (t, *J* = 2.0 Hz, 1H, 12), 6.17 (d, *J* = 2.0 Hz, 2H, 10 & 14), 5.63 (d, *J* = 8.2 Hz, 1H, 7), 4.59 (d, *J* = 8.2 Hz, 1H, 8); ^{13}C NMR (DMSO-*d*$_6$:pyridine-*d*$_5$, 4:1, v/v): δ 191.0 (7′), 164.2 (4′), 159.0 (11 & 13), 158.0 (4), 143.0 (9), 132.7 (6′), 132.3 (3′), 130.6 (1′), 129.6 (1), 128.1 (2 & 6), 126.2 (2′), 115.4 (3 & 5), 109.8 (5′), 106.1 (10 & 14), 101.7 (12), 93.6 (7), 54.8 (8). LC-QTOF-MS: m/z calculated for $C_{21}H_{15}O_5$ [M – H]⁻ 347.0925, found 347.0931.

1.2.2.6 RR$_F$ (Pallidol)

5,10-bis(4-Hydroxyphenyl)-4b,5,9b,10-tetrahydroindeno[2,1-*a*]indene-1,3,6,8-tetraol. ^{1}H NMR (MeOH-*d*$_4$): δ 6.91 (d, *J* = 8.6 Hz, 4H, 2 & 6), 6.65 (d, *J* = 8.6 Hz, 4H, 3 & 5), 6.51 (d, *J* = 2.0 Hz, 2H, 14), 6.09 (d, *J* = 2.0 Hz, 2H, 12), 4.45 (br s, 2H, 7), 3.71 (br s, 2H, 8); ^{13}C NMR (MeOH-*d*$_4$): δ 159.3 (13), 156.3 (4), 155.5 (11), 150.8 (9), 138.4 (1), 129.2 (2 & 6), 123.8 (10), 115.9 (3 & 5), 103.3 (14), 102.5 (12), 60.9 (8), 54.7 (7).
^{1}H NMR (DMSO-*d*$_6$:pyridine-*d*$_5$, 4:1, v/v): δ 9.32 (br s, 2H, 4-OH), 9.29 (br s, 2H, 13-OH), 9.17 (br s, 2H, 11-OH), 6.93 (d, *J* = 8.5 Hz, 4H, 2 & 6), 6.69 (d, *J* = 8.5 Hz, 4H, 3 & 5), 6.55 (d, *J* = 1.8 Hz, 2H, 14), 6.22 (d, *J* = 1.8 Hz, 2H, 12), 4.47 (br s, 2H, 7), 3.71 (br s, 2H, 8); ^{13}C NMR (DMSO-*d*$_6$:pyridine-*d*$_5$, 4:1, v/v): δ 158.3 (13), 155.4 (4), 154.5 (11), 148.9 (9), 136.3 (1), 128.0 (2 & 6), 121.8 (10), 115.0 (3 & 5), 102.0 (14), 101.5 (12), 59.1 (8), 52.6 (7). LC-QTOF-MS: m/z calculated for $C_{28}H_{21}O_6$ [M – H]⁻ 453.1344, found 453.1353.

1.2.3 Piceatannol and sinapyl alcohol cross-coupled dimers and trimer

The cross-coupled products between piceatannol and sinapyl alcohol were prepared using HRP under similar conditions as for the above homo-coupling reactions of resveratrol and piceatannol.

The reaction of piceatannol (300 mg) and sinapyl alcohol (258 mg) produced crossed dimers **SP$_A$** (5.6 mg, 1.0%), **SP$_F$** (4.2 mg, 0.8%), and a trimer **SPP$_H$** (7.3 mg, 1.3%) after TLC separation.

1.2.3.1 SP$_A$ (Aiphanol)

(*E*)-5-(2-(3-(4-Hydroxy-3,5-dimethoxyphenyl)-2-(hydroxymethyl)-2,3-dihydrobenzo[*b*][1,4]-dioxin-6-yl)vinyl)benzene-1,3-diol. ^{1}H NMR (MeOH-*d$_4$*): δ 7.09 (d, *J* = 2.0 Hz, 1H, 2′), 7.04 (dd, *J* = 8.4, 2.0 Hz, 1H, 6′), 6.94 (d, *J* = 8.4 Hz, 1H, 5′), 6.94 (d, *J* = 16.0 Hz, 1H, 7′), 6.83 (d, *J* = 16.0 Hz, 1H, 8′), 6.75 (br s, 2H, 2 & 6), 6.44 (d, *J* = 2.2 Hz, 2H, 10′ & 14′), 6.15 (t, *J* = 2.2 Hz, 1H, 12′), 4.90 (d, *J* = 8.0 Hz, 1H, α), 4.07 (m, 1H, β), 3.86 (s, 6H, OMe), 3.71 (dd, *J* = 12.4, 2.5 Hz, 1H, γ1), 3.49 (dd, *J* = 12.4, 4.3 Hz, 1H, γ2); ^{13}C NMR (MeOH-*d$_4$*): δ 159.7 (11′ & 13′), 149.4 (3 & 5), 145.4 (3′), 144.8 (4′), 141.0 (9′), 137.1 (4), 132.5 (1′), 129.0 (7′), 128.6 (1), 128.4 (8′), 121.1 (6′), 118.1 (5′), 115.6 (2′), 105.9 (2, 6, 10′, 14′), 102.9 (12′), 80.1 (β), 78.0 (α), 62.1 (γ), 56.8 (OMe).

^{1}H NMR (DMSO-*d$_6$*:pyridine-*d$_5$*, 4:1, v/v): δ 7.23 (d, *J* = 2.0 Hz, 1H, 2′), 7.10 (dd, *J* = 8.4, 2.0 Hz, 1H, 6′), 7.00 (d, *J* = 19.0 Hz, 1H, 7′), 7.00 (d, *J* = 19.0 Hz, 1H, 8′), 6.96 (d, *J* = 8.4 Hz, 1H, 5′), 6.80 (br s, 2H, 2 & 6), 6.54 (d, *J* = 2.0 Hz, 2H, 10′ & 14′), 6.30 (t, *J* = 2.0 Hz, 1H, 12′), 4.99 (d, *J* = 8.0 Hz, 1H, α), 4.23 (m, 1H, β), 3.77 (s, 6H, OMe), 3.64 (d, *J* = 12.0 Hz, 1H, γ1), 3.45 (dd, *J* = 12.0, 5.0 Hz, 1H, γ2); ^{13}C NMR (DMSO-*d$_6$*:pyridine-*d$_5$*, 4:1, v/v): δ 158.7 (11′ & 13′), 148.0 (3 & 5), 143.8 (3′), 143.2 (4′), 139.1 (9′), 136.2 (4), 130.5 (1′), 127.4 (7′), 127.3 (8′), 126.6 (1), 120.0 (6′), 116.8 (5′), 114.4 (2′), 105.3 (2 & 6), 104.6 (10′ & 14′), 102.2 (12′), 78.3 (β), 76.2 (α), 60.3 (γ), 56.0 (OMe). LC-QTOF-MS: m/z calculated for $C_{25}H_{23}O_8$ [M – H]$^-$ 451.1398, found 451.1403.

1.2.3.2 SP$_F$ (Kompasinol A, maackolin)

3-(3,4-Dihydroxyphenyl)-8-(4-hydroxy-3,5-dimethoxyphenyl)-3,3a,8,8a-tetrahydro-1*H*-indeno-[1,2-*c*]furan-5,7-diol. ^{1}H NMR (MeOH-*d$_4$*): δ 6.84 (d, *J* = 1.7 Hz, 1H, 2′), 6.76 (d, *J* = 8.1 Hz, 1H, 5′), 6.74 (dd, *J* = 8.1, 1.7 Hz, 1H, 6′), 6.31 (br s, 2H, 2 & 6), 6.23 (d, *J* = 2.0 Hz, 1H, 14′), 6.17 (d, *J* = 2.0 Hz, 1H, 12′), 4.64 (d, *J* = 4.6 Hz, 1H, 7′), 4.46 (t, *J* = 8.7 Hz, 1H, γ1), 4.12 (br s, 1H, α), 3.75 (m, 1H, 8′), 3.72 (s, 6H, OMe), 3.53 (t, *J* = 8.7 Hz, 1H, γ2), 3.02 (m, 1H, β); ^{13}C NMR (MeOH-*d$_4$*): δ 160.1 (13′) 156.3 (11′), 149.1 (3 & 5), 148.6 (9′), 146.5 (3′), 146.1 (4′), 137.9 (1), 135.2 (1′), 134.6 (4), 122.9 (10′), 119.0 (6′), 116.2 (5′), 114.5 (2′), 105.4 (2, 6), 103.2 (14′), 102.8 (12′), 89.4 (7′), 75.0 (γ), 59.8 (8′), 56.6 (OMe), 56.5 (β), 52.0 (α).

^{1}H NMR (DMSO-*d$_6$*:pyridine-*d$_5$*, 4:1, v/v): δ 6.92 (d, *J* = 1.8 Hz, 1H, 2′), 6.81 (d, *J* = 8.0 Hz, 1H, 5′), 6.71 (dd, *J* = 8.0, 1.8 Hz, 1H, 6′), 6.34 (br s, 2H, 2 & 6), 6.32 (d, *J* = 2.0 Hz, 1H, 14′), 6.31 (d, *J* = 2.0 Hz, 1H, 12′), 4.65 (d, *J* = 4.1 Hz, 1H, 7′), 4.38 (t, *J* = 8.5 Hz, 1H, γ1), 4.12 (br s, 1H, α), 3.75 (m, 1H, 8′), 3.62 (s, 6H, OMe), 3.41 (t, *J* = 8.5 Hz, 1H, γ2), 2.94 (m, 1H, β); ^{13}C NMR

(DMSO-d_6:pyridine-d_5, 4:1, v/v): δ 158.8 (13′) 155.1 (11′), 147.8 (3 & 5), 147.1 (9′), 145.4 (3′), 144.7 (4′), 136.1 (1), 134.0 (1′), 133.9 (4), 121.2 (10′), 117.0 (6′), 115.4 (5′), 113.7 (2′), 104.8 (2, 6), 101.8 (14′ or 12′), 101.8 (14′ or 12′), 87.0 (7′), 73.2 (γ), 58.2 (8′), 55.8 (OMe), 54.4 (β), 50.2 (α). LC-QTOF-MS: m/z calculated for $C_{25}H_{23}O_8$ $[M – H]^–$ 451.1398, found 451.1396.

1.2.3.3 SPP$_H$

(*E*)-4-(3-(3,5-Dihydroxyphenyl)-6-hydroxy-4-(2-(3-(4-hydroxy-3,5-dimethoxyphenyl)-2-(hydroxymethyl)-2,3-dihydrobenzo[*b*][1,4]dioxin-6-yl)vinyl)-2,3-dihydrobenzofuran-2-yl)benzene-1,2-diol (see table S3). LC-QTOF-MS: m/z calculated for $C_{39}H_{33}O_{12}$ $[M – H]^–$ 693.1978, found 693.1973.

1.2.4 Resveratrol and sinapyl alcohol cross-coupled dimers

The cross-coupled products between resveratrol and sinapyl alcohol were prepared using HRP under similar conditions as for the above cross-coupling of piceatannol and sinapyl alcohol. The reaction of resveratrol (300 mg) and sinapyl alcohol (276 mg) produced dimers **SR$_C$** (12.3 mg, 2.1%) and **SR$_E$** (6.1 mg, 1.1%) after TLC separation.

1.2.4.1 SR$_C$

(*E*)-4-(3-(3,5-Dihydroxyphenyl)-6-hydroxy-4-(2-(3-(4-hydroxy-3,5-dimethoxyphenyl)-2-(hydroxymethyl)-2,3-dihydrobenzo[*b*][1,4]dioxin-6-yl)vinyl)-2,3-dihydrobenzofuran-2-yl)benzene-1,2-diol. ^{1}H NMR (MeOH-d_4): δ 7.35 (d, *J* = 8.6 Hz, 2H, 2′ & 6′), 6.98 (d, *J* = 16.2 Hz, 1H, 7′), 6.89 (d, *J* = 16.2 Hz, 1H, 8′), 6.75 (d, *J* = 8.6 Hz, 2H, 3′ & 5′), 6.61 (d, *J* = 1.8 Hz, 1H, 14′), 6.60 (s, 2H, 2 & 6), 6.24 (d, *J* = 1.8 Hz, 1H, 12′), 5.63 (d, *J* = 1.5 Hz, 1H, α), 3.89 (d, *J* = 7.4 Hz, 1H, γ1), 3.78 (s, 6H, OMe), 3.54 (br s, 1H, β), 3.53 (d, *J* = 7.4 Hz, 1H, γ2); ^{13}C NMR (MeOH-d_4): δ 162.7 (11′) 159.9 (13′), 158.7 (4′), 149.2 (3 & 5), 136.9 (9′), 135.9 (4), 135.0 (1), 131.2 (7′), 130.2 (1′), 129.0 (2′ & 6′), 123.2 (8′), 116.5 (3′ & 5′), 116.5 (10′), 104.3 (14′), 103.3 (2 & 6), 97.1 (12′), 88.6 (α), 65.4 (γ), 56.6 (OMe), 54.4 (β).

^{1}H NMR (DMSO-d_6:pyridine-d_5, 4:1, v/v): δ 7.43 (d, *J* = 8.6 Hz, 2H, 2′ & 6′), 7.04 (d, *J* = 16.3 Hz, 1H, 8′), 6.99 (d, *J* = 16.3 Hz, 1H, 7′), 6.83 (d, *J* = 8.6 Hz, 2H, 3′ & 5′), 6.72 (d, *J* = 1.7 Hz, 1H, 14′), 6.66 (s, 2H, 2 & 6), 6.38 (d, *J* = 1.7 Hz, 1H, 12′), 5.74 (d, *J* = 2.7 Hz, 1H, α), 3.85 (dd, *J* = 10.0, 4.1 Hz, 1H, γ1), 3.69 (m, 1H, β), 3.68 (s, 6H, OMe), 3.49 (t, *J* = 10.0 Hz, 1H, γ2); ^{13}C NMR (DMSO-d_6:pyridine-d_5, 4:1, v/v): δ 161.0 (11′) 158.7 (13′), 157.8 (4′), 148.1 (3 & 5), 135.2 (9′), 135.0 (4), 132.8 (1), 129.5 (7′), 128.0 (2′ & 6′), 128.0 (1′), 122.2 (8′), 115.6 (3′ & 5′), 115.5 (10′), 103.0 (14′), 102.7 (2 & 6), 96.2 (12′), 86.6 (α), 63.8 (γ), 55.9 (OMe), 52.6 (β). LC-QTOF-MS: m/z calculated for $C_{25}H_{23}O_7$ $[M – H]^–$ 435.1449, found 435.1457.

1.2.4.2 SR$_E$

(*E*)-2-(4-Hydroxy-3,5-dimethoxyphenyl)-3-(hydroxymethyl)-6-(4-hydroxystyryl)-2,3-dihydrobenzofuran-4-ol. ^{1}H NMR (MeOH-*d*$_4$): δ 7.36 (d, *J* = 8.6 Hz, 2H, 2′ & 6′), 6.99 (d, *J* = 16.3 Hz, 1H, 7′), 6.85 (d, *J* = 16.3 Hz, 1H, 8′), 6.76 (d, *J* = 8.6 Hz, 2H, 3′ & 5′), 6.67 (s, 2H, 2 & 6), 6.55 (s, 1H, 10′), 6.50 (s, 1H, 14′), 5.33 (d, *J* = 6.9 Hz, 1H, α), 3.91 (m, 1H, γ1), 3.82 (s, 6H, OMe), 3.79 (m, 1H, γ2), 3.56 (m, 1H, β); ^{13}C NMR (MeOH-*d*$_4$): δ 162.9 (12′) 158.4 (4′), 155.6 (13′), 149.4 (3 & 5), 141.6 (9′), 136.4 (4), 133.9 (1), 130.4 (1′), 129.4 (7′), 128.8 (2′ & 6′), 126.9 (8′), 116.5 (3′ & 5′), 113.5 (11′), 107.9 (14′), 104.1 (2 & 6), 99.3 (10′), 88.3 (α), 64.5 (γ), 56.7 (OMe), 54.2 (β).

^{1}H NMR (DMSO-*d*$_6$:pyridine-*d*$_5$, 4:1, v/v): δ 7.42 (d, *J* = 8.6 Hz, 2H, 2′ & 6′), 7.09 (d, *J* = 16.3 Hz, 1H, 7′), 6.95 (d, *J* = 16.3 Hz, 1H, 8′), 6.83 (d, *J* = 8.6 Hz, 2H, 3′ & 5′), 6.72 (s, 2H, 2 & 6), 6.69 (s, 1H, 10′), 6.61 (s, 1H, 14′), 5.52 (d, *J* = 6.4 Hz, 1H, α), 3.94 (m, 1H, γ1), 3.73 (m, 1H, γ2), 3.71 (s, 6H, OMe), 3.63 (m, 1H, β); ^{13}C NMR (DMSO-*d*$_6$:pyridine-*d*$_5$, 4:1, v/v): δ 161.4 (12′) 157.4 (4′), 154.5 (13′), 148.1 (3 & 5), 139.5 (9′), 135.5 (4), 131.8 (1), 128.2 (1′), 128.1 (7′), 127.9 (2′ & 6′), 125.5 (8′), 115.6 (3′ & 5′), 112.4 (11′), 107.0 (14′), 103.4 (2 & 6), 97.8 (10′), 86.6 (α), 62.2 (γ), 55.9 (OMe), 52.5 (β). LC-QTOF-MS: m/z calculated for $C_{25}H_{23}O_7$ [M – H]$^-$ 435.1449, found 435.1453.

2. Results of hydroxystilbene dimers and trimers

2.1 Synthesis of hydroxystilbenes

To prepare radical-coupled hydroxystilbene dimers and trimers, we first synthesized the hydroxystilbene monomers. The isolated monomers can be purchased, but the commercially available hydroxystilbenes are expensive, except for resveratrol, because of their limited supply from natural sources. As the diverse, powerful biological activities and medicinal properties of hydroxystilbenes have attracted general interest (*78*), many synthetic methods have been developed for hydroxystilbenes (*74*). These synthetic strategies can be useful for synthesizing different kinds of hydroxystilbenes, especially when isotope-labeled compounds are required. Overall the reactions were straightforward, but the most challenging part of the entire reaction step was the debenzylation at the very end of the synthesis. The previous recipe (*74*) used boron tribromide (BBr_3), but we were not successful with using the method. Instead, aluminum chloride ($AlCl_3$) and *N,N*-dimethylaniline proved most effective for producing the final products (*75, 76*); for the benzyl-protected isorhapontigenin **10**, anisole was used in place of *N,N*-dimethylaniline. We simply purchased resveratrol for this research as it was relatively cheap compared with other hydroxystilbenes.

2.1.1 Biomimetic preparation of piceatannol dimers

The horseradish peroxidase reaction in acetate buffer (pH 5) was carried out on pre-dissolved piceatannol in a small volume of acetone that was introduced into the buffer. The reaction was stirred for only 20 min at room temperature to avoid excessive polymerization. The reaction solution became dark immediately after the reaction was started by adding H_2O_2. NMR of the crude product confirmed that **PP$_A$** (cassigarol E) and **PP$_C$** (scirpusin B) were the main products produced in comparable amounts. The actual production of the piceatannol dimers and trimers was best achieved using inorganic oxidants, MnO_2, $FeCl_3$•$6H_2O$, Ag_2O, and AgOAc, in various organic solvents. **PP$_A$** (cassigarol E, Fig. 1D), widely distributed in nature and commonly isolated from many plants, such as *Cassia garrettiana, Maackia amurensis, and Cyperus longus* (*45, 79, 80*), was one of the main products from all reaction conditions except $FeCl_3$•$6H_2O$, and was essentially the sole product of MnO_2 and Ag_2O reactions. **PP$_A$** was formed via 8–O–4′ coupling (fig. S5-1A); the 8-position is analogous to the β-position of a monolignol. After the ether bond is formed, the quinone methide intermediate was quenched by intramolecular trapping with the 3′-OH group (*81*), resulting in a 6-membered benzodioxane structure from the catechol moiety of one piceatannol. The benzodioxane structure, which is similar to radical reaction products of caffeyl alcohol and 5-hydroxyconiferyl alcohol, was elucidated from NMR experiments, with the characteristic 7- and 8-positions appearing closely at δ_C/δ_H 79.4/4.93 and 79.8/4.95 in DMSO-d_6:pyridine-d_5 (4:1, v/v) in the diagnostic 2D HSQC NMR spectrum. Other correlation peaks appeared in the area of 100 to 108/6.0 to 6.7 ppm (δ_C/δ_H).

PP$_C$ (scirpusin B, Fig. 1D) has been isolated from plants, *Scirpus fluviatilis* and *Cassia garrettiana* (*79, 82*), but has not been reported previously as a product of biomimetic radical coupling reaction. **PP$_C$** was produced as essentially the only product from $FeCl_3$•$6H_2O$ in acetone. Two piceatannol molecules can participate in 8–10′ radical coupling to generate the coumaran (dihydrobenzofuran) ring structure following consequent 11′–O–7 bonding during rearomatization of the quinone methide intermediate (fig. S5-1C). The 11′-hydroxyl group is located on the resorcinol moiety of hydroxystilbenes such that both piceatannol and resveratrol 8-10′ coupling results in the 5-membered coumaran structure. Such coupling is achievable owing to the extended conjugation system of the hydroxystilbene structures, in which single-electron density from the radical produced on the 4-OH of hydroxystilbenes extends to 10′/14′ and 12′ positions of the resorcinol moiety. The internal trapping of the quinone methide through nearby hydroxyl groups is common as well known in lignin and in the β–5-dehydrodiconiferyl alcohol (phenylcoumaran) dimer **B** (Fig. 1A). **PP$_C$** showed distinctive 7- and 8-position peaks at δ_C/δ_H 92.6/5.34 and 55.8/4.44 in DMSO-d_6:pyridine-d_5 (4:1, v/v). Another distinctive 12′-position peak appeared at δ_C/δ_H 96.0/6.36 whereas the other resorcinol moiety peaks are again in the area of 100 to 108/6.0 to 6.7 ppm (δ_C/δ_H). The AgOAc reaction produced **PP$_A$** as one of the main products but did not generate **PP$_C$**. Instead, another phenylcoumaran structure, **PP$_D$** (maackin A), was

produced via 8-5′ coupling, and the quinone methide intermediate was quenched by intramolecular trapping to form the 4′–O–7 bond again producing a phenylcoumaran (fig. S5-2D). This structure more closely resembles dehydrodiconiferyl alcohol (the β–5 phenylcoumaran) **B** from coniferyl alcohol dimerization than $\mathbf{PP_C}$, and 3′-OH group remained as free as the radical coupling formed on 5′-position of catechyl moiety. The phenylcoumaran structure of $\mathbf{PP_D}$ showed a similar 7-position correlation at δ_C/δ_H 92.8/5.39 in DMSO-d_6:pyridine-d_5 (4:1, v/v), and the chemical shift is very close to the peak from $\mathbf{PP_C}$. The 8-position peaks at δ_C/δ_H 56.8/4.45 showed a slightly downfield carbon signal from $\mathbf{PP_C}$, but the difference is insignificant. The maackin A ($\mathbf{PP_D}$) structure was previously confirmed from a radical dehydrodimerization reaction of piceatannol using horseradish peroxidase, which also produced cassigarol E ($\mathbf{PP_A}$) (*81*). $\mathbf{PP_D}$ was also isolated from *Maackia amurensis* as a natural product (*83*). $\mathbf{PP_E}$ (gneafricanin C) is the other 5-membered coumaran ring structure that could be produced by coupling two piceatannol monomers (fig. S5-2E), but it was not obtained in this study. This compound is produced by 8-12′ coupling followed by 11′–O–7 bond formation during rearomatization and cyclization. Unfortunately, we could not isolate this structure. The hindered 12′-position is possibly the main reason that the 8–12′ coupling is less prevalent *in vitro* even though the natural compound, gneafricanin C ($\mathbf{PP_E}$), is known (*84*).

2.1.2 Biomimetic preparation of resveratrol dimers

Resveratrol structures have been more intensively studied in the previous literature compared to those from piceatannol (*30, 32*). Resveratrol was examined here under the same horseradish peroxidase reaction conditions as used for piceatannol, and as before the reaction solution became dark immediately upon the addition of H_2O_2; however, the structures of the main products were slightly different from the piceatannol products, with the collection of $\mathbf{RR_B}$ (labruscol), $\mathbf{RR_D}$ (δ-viniferin), and $\mathbf{RR_{D\text{-}oxi}}$ (oxidized δ-viniferin). $\mathbf{RR_B}$ was formed by 8–O–4′ coupling followed by quenching the quinone methide with the addition of water to the 7-position (fig. S5-1B); unlike in the analogous coupling on the way to $\mathbf{PP_A}$, there is no phenolic-OH group on the aromatic ring to internally trap the quinone methide intermediate. This 8–O–4′ coupled structure of $\mathbf{RR_B}$ closely resembles the β–O–4′ aryl ether structure **A** of lignin (or the analogous β-ether dimer from monolignols). The distinctive 7- and 8-positions appeared at δ_C/δ_H 76.3/4.81 and 84.3/5.12 respectively in the NMR spectrum. Labruscol ($\mathbf{RR_B}$) has been isolated from *Vitis labrusca*, a species of grapevine, and the structure was elucidated (*85*). The NMR data were collected in MeOH-d_4, and they match well with our data in this study with the same NMR solvent. Oxidative dimerization of a methylated analog of resveratrol, 3,5-dimethoxy-4′-hydroxystilbene (10,13-dimethoxy using the numbering here in Fig. 1C), using laccase in acetate buffer (pH 4.5) provided a methylated labruscol previously (*86*), illustrating that the resorcinol unit hydroxyls

(11- and 13-OH) are not involved in the radical coupling, only the 4-OH on the *p*-hydroxyphenyl moiety. We were able to obtain a proper form of labruscol from non-derivatized resveratrol here. **RR_D** (δ-viniferin) was the dominant product under the peroxidase reaction condition and essentially the only product isolated from the AgOAc reaction in agreement with previously reports (fig. S5-2D) (*87*, *88*). **RR_D** has the same 8–5′ phenylcoumaran structure as **PP_D** from piceatannol, and with similar chemical shifts of 7-position at δ_C/δ_H 92.8/5.46, but the 8-position peaks are closer to those in **PP_C**, the 8–10′ coupled coumaran structure, at δ_C/δ_H 56.0/4.50 in DMSO-d_6:pyridine-d_5 (4:1, v/v). During the peroxidase reaction, oxidative cleavage of the double bond between 7′ and 8′ of resveratrol monomer resulted in *p*-hydroxybenzaldehyde, and it cross-coupled with resveratrol monomer to form **$RR_{D\text{-}oxi}$** as a byproduct (fig. S5-2D-2). According to previous studies, **RR_C** (ε-viniferin), a 8–10′ coupled coumaran structure, was not produced from the peroxidase or AgOAc reactions, but appeared as the main product under the $FeCl_3$•$6H_2O$ reaction condition (*87*, *89*), and we also produced it from the same $FeCl_3$•$6H_2O$ reaction as the main product (fig. S5-1C). The 7- and 8-position peaks have similar chemical shifts to **RR_D** at δ_C/δ_H 92.5/5.43 and 55.4/4.50. **RR_C** is therefore a resveratrol analog of **PP_C** (scirpusin B), whereas **RR_D** shares the basic phenylcoumaran skeleton with **PP_D** (maackin A). From the same reaction, ω-viniferin (**$RR_{C\text{-}1}$**) was also collected as one of the minor products. It is an analog of **RR_C**, but the coumaran ring of **$RR_{C\text{-}1}$** has a *cis* form unlike **RR_C**, which has a *trans* form (fig. S5-1C). As a result, the 7- and 8-position peaks of the coumaran structure moved significantly to upfield to δ_C/δ_H 89.0/5.84 and 51.3/4.67, respectively. It was previously synthesized using ruthenium chloride, and the NMR data matches well (*90*). Both δ- (**RR_D**) and ε-viniferin (**RR_C**) commonly exist in grapevines, and they have been studied intensively for their health-promoting and biological properties, such as anti-inflammatory, anti-asthmatic, anti-cancer, anti-HIV, and so on (*77*, *91*). **RR_E** (gnetin C), an analog of **PP_E** (gneafricanin C) and exists as a natural compound, is a 5-membered coumaran structure could be produced by 8-12′-coupling position followed by 11′–O–7 bond formation during quinone methide intermediate rearomatization (fig. S5-2E and S4-2), but we were not able to collect this one in this study. The other minor product we collected from resveratrol in the $FeCl_3$•$6H_2O$ reaction was **RR_F** (pallidol). Pallidol-like products were produced from similar reaction conditions in previous studies (*88*), and pallidol was extracted from *Cissus pallida* (*92*). **RR_F** is a symmetrically formed dimer between two resveratrol molecules via 8–8-coupling, and resembles the formation of resinol structures in lignin (fig. S5-2F). The first quinone methide intermediates were quenched by intramolecular trapping via nucleophilic attack of the 10-positions of the resorcinol moieties to cyclize the molecule, and the second quinone methide intermediates were rearomatized by proton elimination from the 10-positions. The unique 8–8 coupled structure with a center of symmetry shows the 7- and 8-position peaks at δ_C/δ_H 52.6/4.47 and 59.1/3.71, respectively.

3. NMR results of hydroxystilbenes in palm fruit endocarp lignins

After we discovered the hydroxystilbene-containing lignins from macaúba, carnauba, and coconut palm fruit endocarps (*13*), subsequent studies disclosed additional information about the tissue-specific localization of hydroxystilbenes in macaúba fruit endocarp as well as the occurrence of their glucosides in the lignin of Norway spruce bark (*28, 37*). The identification of the newly found structures was achieved by NMR experiments with limited sources to hydroxystilbene models. Now we have 19 synthesized hydroxystilbene compounds and various combinations of DHPs to verify the lignin structures in this study.

Considerable differences exist in the spectra of these lignins from other mainstream lignins that we typically observe, especially in the aromatic region (fig. S2, A, B, and C). As described earlier, the endocarp lignins most closely resembled to the P + G + S DHP (Fig. 5D). The lignin's aromatic correlations showed G-units at δ_C/δ_H 110.8/7.05, S-units at δ_C/δ_H 104.1/6.76, oxidized S′-units δ_C/δ_H 106.5/7.22, and the *p*-hydroxybenzoates (*p*B) at δ_C/δ_H 131.2/7.75. The new strong peaks at δC/δH 100–107/6.0–6.8 belong to resorcinol moieties of hydroxystilbenes, and they are the main featured correlations that make the new lignins unique. The chemical shifts of $\mathbf{G}_2$, $\mathbf{G}_5$, and $\mathbf{G}_6$ also match with the ones of DHPs at δ_C/δ_H 110.8/7.05, δ_C/δ_H 114.9/6.86, and δ_C/δ_H 118.9/6.87. As the lignins have much higher Mw and longer polymer length than DHPs, the α and β of cinnamyl alcohol end-groups were hardly visible at δ_C/δ_H 128.5/6.53 ($\mathbf{I}_\alpha$) and 127.9/6.28 ppm ($\mathbf{I}_\beta$). The main hydroxystilbene components of the lignins are structurally more simpler than in DHPs. The hydroxystilbene peaks were more likely to correspond to piceatannol peaks of $\mathbf{PP_A}$ and $\mathbf{PP_C}$ than other structures, but many other piceatannols, such as $\mathbf{PP_D}$, $\mathbf{SP_F}$, $\mathbf{SP_A}$, and minor hydroxystilbenes can still potentially share the chemical shifts if they exist in the lignin. The cross-coupled structure between monolignols and piceatannol $\mathbf{SP_A}$, which has a benzodioxane structure, may be underneath other peaks in the aromatic area of the lignins, but it was not clear. Another cross-coupled structure $\mathbf{SP_F}$ (kompasinol A), instead, was detected in the lignin samples. The potential $\mathbf{SP_{F2'}}$ peak was identified from the lignin's aromatic data (fig. S2, A, B, and C), but the aliphatic peaks were weaker than the aromatic ones and appeared at only trace levels (fig. S2, D, E, and F). The β–8′ cross-coupled structure can function as an initiation site of lignin polymerization like monolignol β–β coupled structures (e.g., pinoresinol and syringaresinol). The area of unsaturated end-group peaks at the δ_C/δ_H 122–130/6.7–7.5 was nearly empty unlike in DHPs. This indicates that piceatannol was highly polymerized with monolignols to form high-Mw lignin polymers, and also that resveratrol was not actively involved in the lignification.

The aliphatic region (δ_C/δ_H 45–98/2.3–6.8) of the MWLs was also compared to the DHPs and the general sidechain structures of lignins, β-aryl ethers **A**, phenylcoumarans **B**, resinols **C**, and cinnamyl alcohol endgroups **I**, were nicely matched and confirmed (fig. S2). Besides the normal

sidechain peaks, the HSQC spectra also showed the γ-acylated structures of β-aryl ethers **A′**, phenylcoumarans **B′**, and cinnamyl alcohol endgroups **I′**. The resinol-related tetrahydrofuran substructure **C′**, resulting from the β−β coupling of two γ-acylated sinapyl alcohols, was also observed. This acylated sidechain with the *p*-hydroxybenzoates ***p*B**, observed in the aromatic region, occurs in palm lignins. Similarly, acylated structures with other functional groups, e.g., *p*-coumarates, ferulates, benzoates, and acetates, also exist in other species. These structures were carefully discussed in the detail in our previous works (*13*, *28*, *37*). Some hydroxystilbene peaks were detected in the aliphatic region along with the conventional lignin correlations, and they mostly belong to piceatannol, $\mathbf{PP_A}$, $\mathbf{PP_C}$, $\mathbf{PP_D}$, and $\mathbf{SP_A}$, as discussed earlier. $\mathbf{PP_{C12'}}$ peak was found at δ_C/δ_H 95.8/6.37. $\mathbf{PP_{C7}}$ and $\mathbf{PP_{D7}}$ were about at δ_C/δ_H 92.5/5.34, and $\mathbf{PP_{C8}}$ at δ_C/δ_H 55.1/4.45. $\mathbf{PP_{D8}}$ was found at δ_C/δ_H 56.5/4.45 as a trace. In all MWLs, homo-coupled piceatannol benzodioxane structure $\mathbf{PP_A}$ ($\mathbf{PP_{A7+8}}$ peak at δ_C/δ_H 79.3/4.94) was a major component, and cross-coupled benzodioxane $\mathbf{SP_A}$, which was detected as one of the main structures in the DHPs, was identified as a minor component. Although piceatannol favors forming the homo-coupled benzodioxane structure $\mathbf{PP_A}$, it also cross-coupled to form benzodioxane $\mathbf{SP_A}$ in MWLs (fig. S2, D, E, and F). The promising occurrence of $\mathbf{SP_A}$ in the aliphatic area with β–8′ $\mathbf{SP_F}$ in the aromatic area (fig. S2, A, B, and C) supports a contention that piceatannol can act as a lignin monomer participating during the lignification. The β–10′ phenylcoumaran cross-coupled $\mathbf{SR_C}$ and another β–8′ $\mathbf{SR_F}$ structures were not observed in the lignin by NMR, even though $\mathbf{SR_C}$ was collected from the radical dimerization reaction and also $\mathbf{SR_F}$ was detected from the DHPs (Fig. 6, E and F).

Resveratrol has a strong potential to be part of the lignin structure as such units were identified from the biomimetic oxidative radical reaction conditions. In fact, resveratrol has not been found as stilbenolignans in nature, although its dehydrodimers and higher oligomers have been discovered (*30*, *32*, *33*). It is also something of a mystery why we detect resveratrol only as a minor component in the lignins of palm fruit endocarps (fig. S7 and Table 3) (*13*). That suggests more investigations are needed for the wide range of plant species that could have hydroxystilbenes incorporated into their lignins.

The relative semi-quantitation of the lignin units and linkages by NMR is reported for both aromatics and aliphatics in fig. S2. In the aromatic region, the S/G ratios of carnauba and macaúba endocarp lignins were similar, but coconut had slightly lower S-units than the other two lignins. Coconut contained a relatively higher level of H-units than others whereas macaúba had the highest content of *p*B among them. Most importantly, carnauba lignin showed the most significant level of hydroxystilbenes that are mostly piceatannol, and macaúba and coconut followed next. The aliphatic area showed typical lignin sidechain compositions with a high level of β–O–4′ ethers. The relative measurement of hydroxystilbenes in this area is lower than in the

aromatic area, but carnauba lignin still showed the higher level of hydroxystilbenes than macaúba and coconut.

4. Supplementary figures and tables

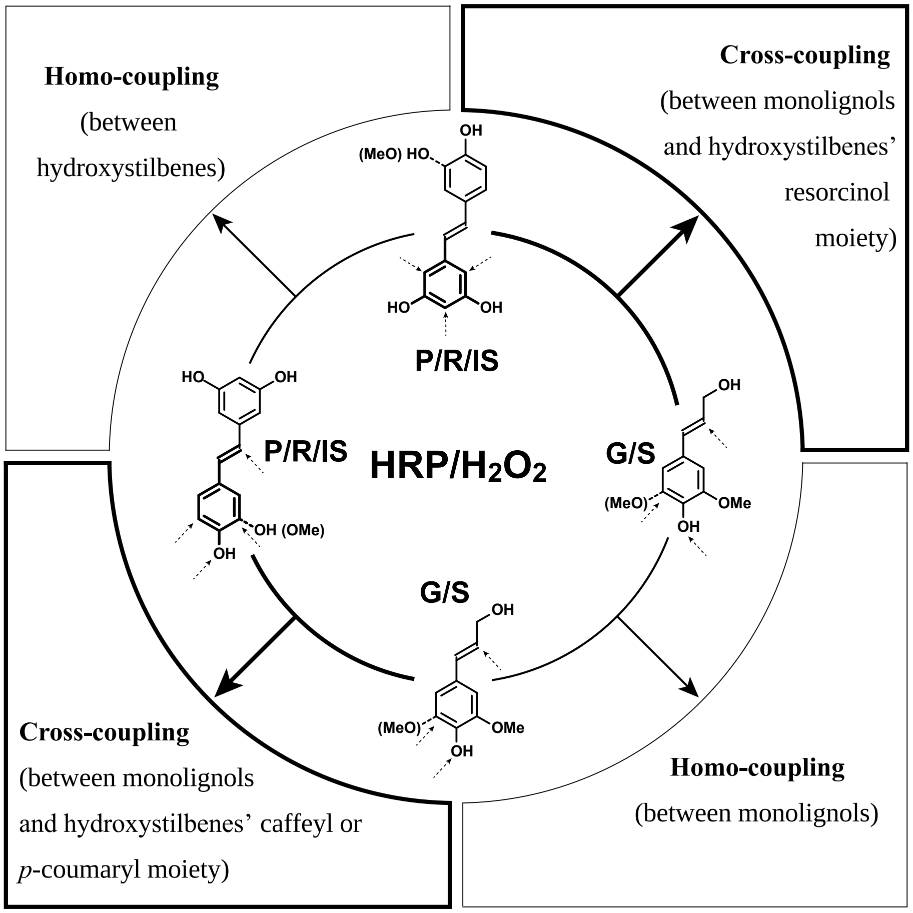

P, piceatannol; **R**, resveratrol; **IS**, isorhapontigenin; **G**, coniferyl alcohol; **S**, sinapyl alcohol

Fig. S1. Coupling and cross-coupling of monolignols and hydroxystilbenes. The combinatorial coupling reaction can produce various products, including homo-coupled products and cross-coupled products between monolignols and hydroxystilbenes. Hydroxystilbenes generally have two different aromatics, a *p*-hydroxyphenyl, catechyl, or guaiacyl moiety and a resorcinol moiety (Fig. 1), and provide more diverse products than monolignols. Radical coupling on the resorcinol moiety occurs via the phenolic radical from the 4-OH as shown in Fig. 2B. **G**, coniferyl alcohol; **S**, sinapyl alcohol; **P**, piceatannol; **R**, resveratrol; **IS**, isorhapontigenin.

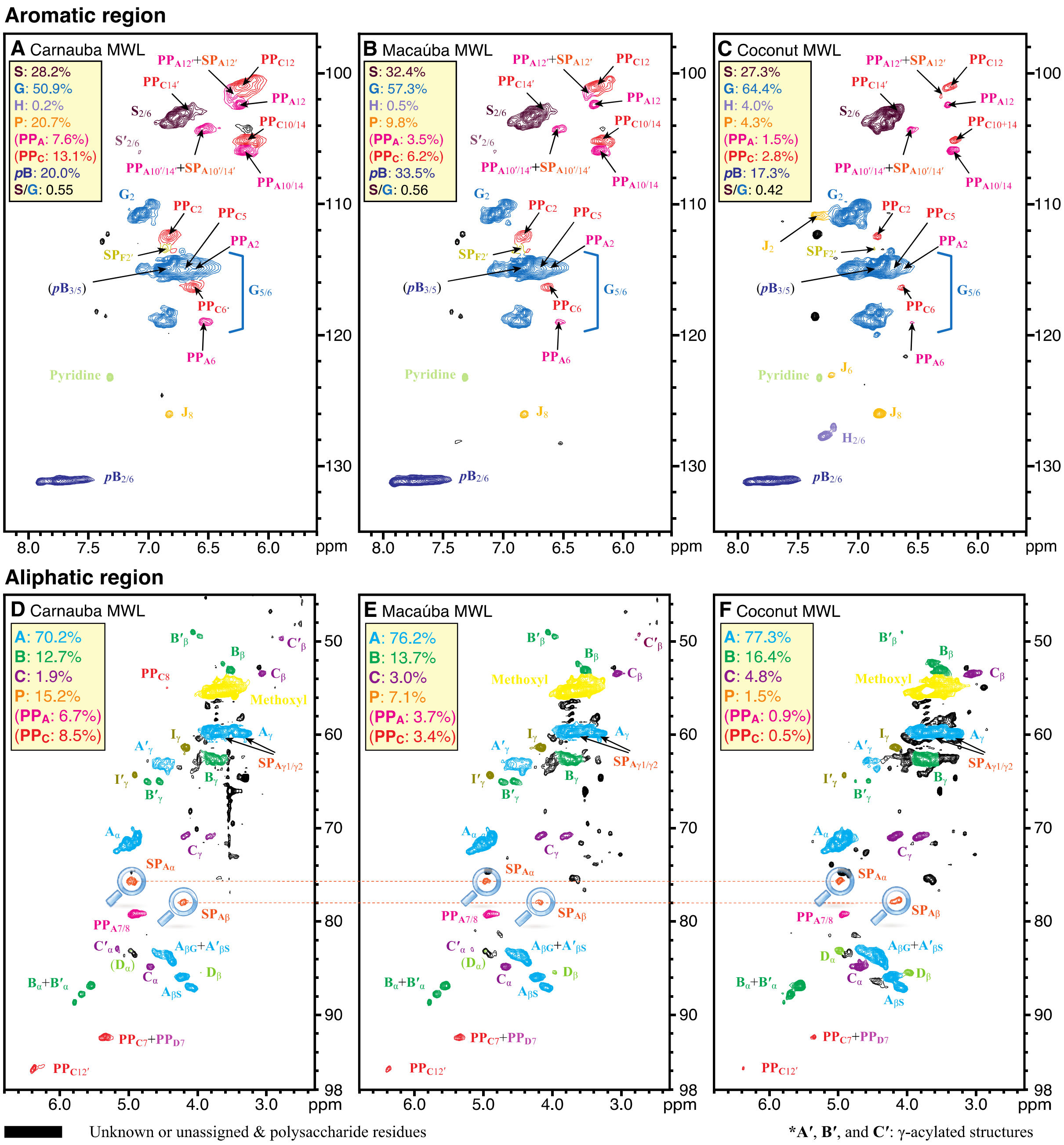

Fig. S2. 2D HSQC NMR spectra of various milled wood lignins (MWLs). Structures and coloring are as defined in Fig. 1. (A) Aromatic region of carnauba fruit endocarp MWL. (B) Aromatic region of macaúba fruit endocarp MWL. (C) Aromatic region of coconut endocarp MWL. (D) Aliphatic region of carnauba fruit endocarp MWL. (E) Aliphatic region of macaúba fruit endocarp MWL. (F) Aliphatic region of coconut endocarp MWL. The hydroxystilbene-derived (mainly piceatannol **P**) peaks in the palm endocarp MWLs are well-matched with those from the biomimetic *in vitro* DHP structures in Fig. 5 and 6. All spectra were collected in DMSO-d_6:pyridine-d_5 (4:1, v/v). Compositional percentages are on an S + G + H + P = 100% basis for the aromatics; the *p*-hydroxybenzoates are not included in this total estimation but are expressed as a percentage of that total because it is pendent on the lignin and potentially overestimated. Percentages for the various aliphatic units are also from volume-integration and total 100%. The piceatannol-monolignol DHPs provided evidence of the cross-coupling reactions to produce $\mathbf{SP_A}$ and $\mathbf{SP_F}$ (Fig. 5 and 6) that are evident in all lignin spectra here at trace levels. **A′**, **B′**, and **C′**: γ-acylated structures.

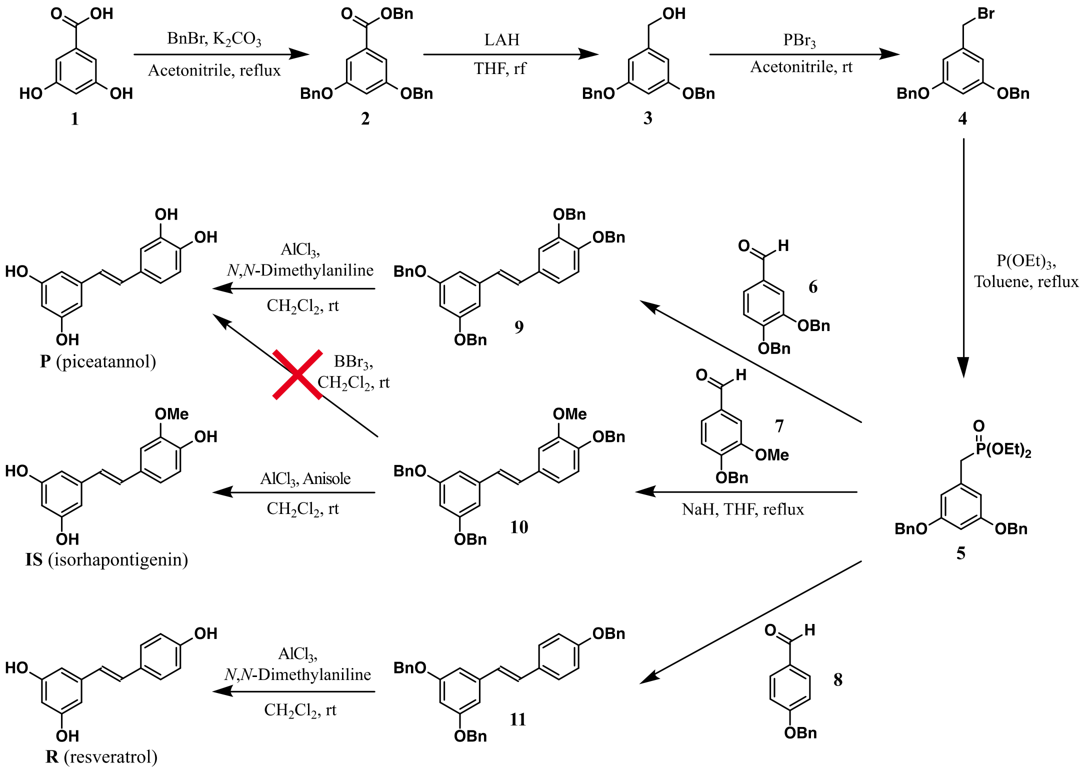

Fig. S3. Total synthesis of hydroxystilbenes, piceatannol, isorhapontigenin, and resveratrol. Different combinations of Wittig–Horner reaction between diethyl phosphonate **5** and hydroxy-benzaldehydes **6**, **7**, and **8** provide different hydroxystilbenes.

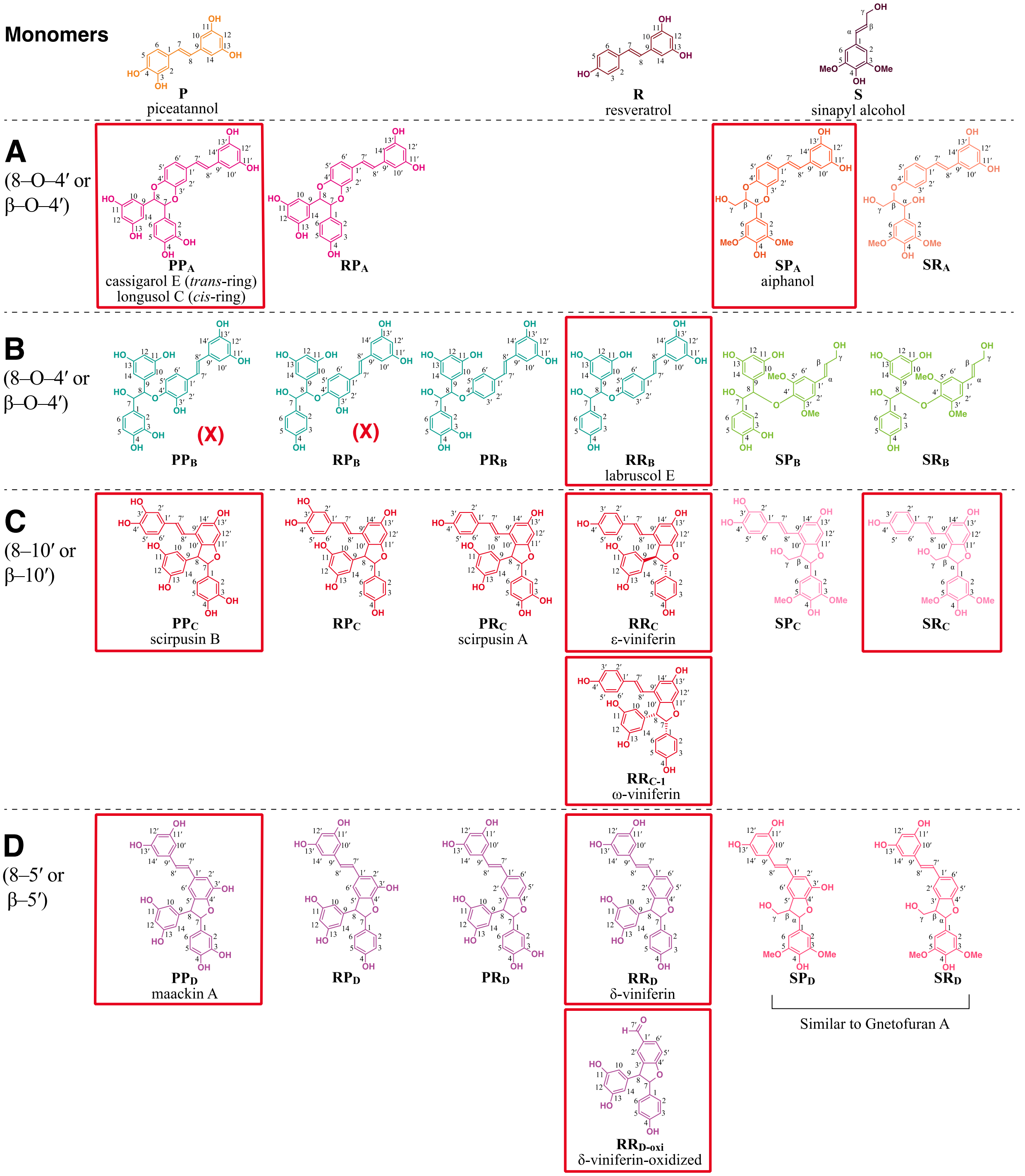

Fig. S4-1. Hydroxystilbene structures. Piceatannol and resveratrol can produce dimers and trimers by homo- and cross-coupling reactions. Cross-coupling also can occur between the hydroxystilbenes and monolignols. Here we isolated various hybrid structures from the cross-coupling of hydroxystilbenes and sinapyl alcohol. The red boxes are the compounds that we isolated and for which NMR data were collected to authenticate the DHP and lignin structures. (Continues in fig. S4-2).

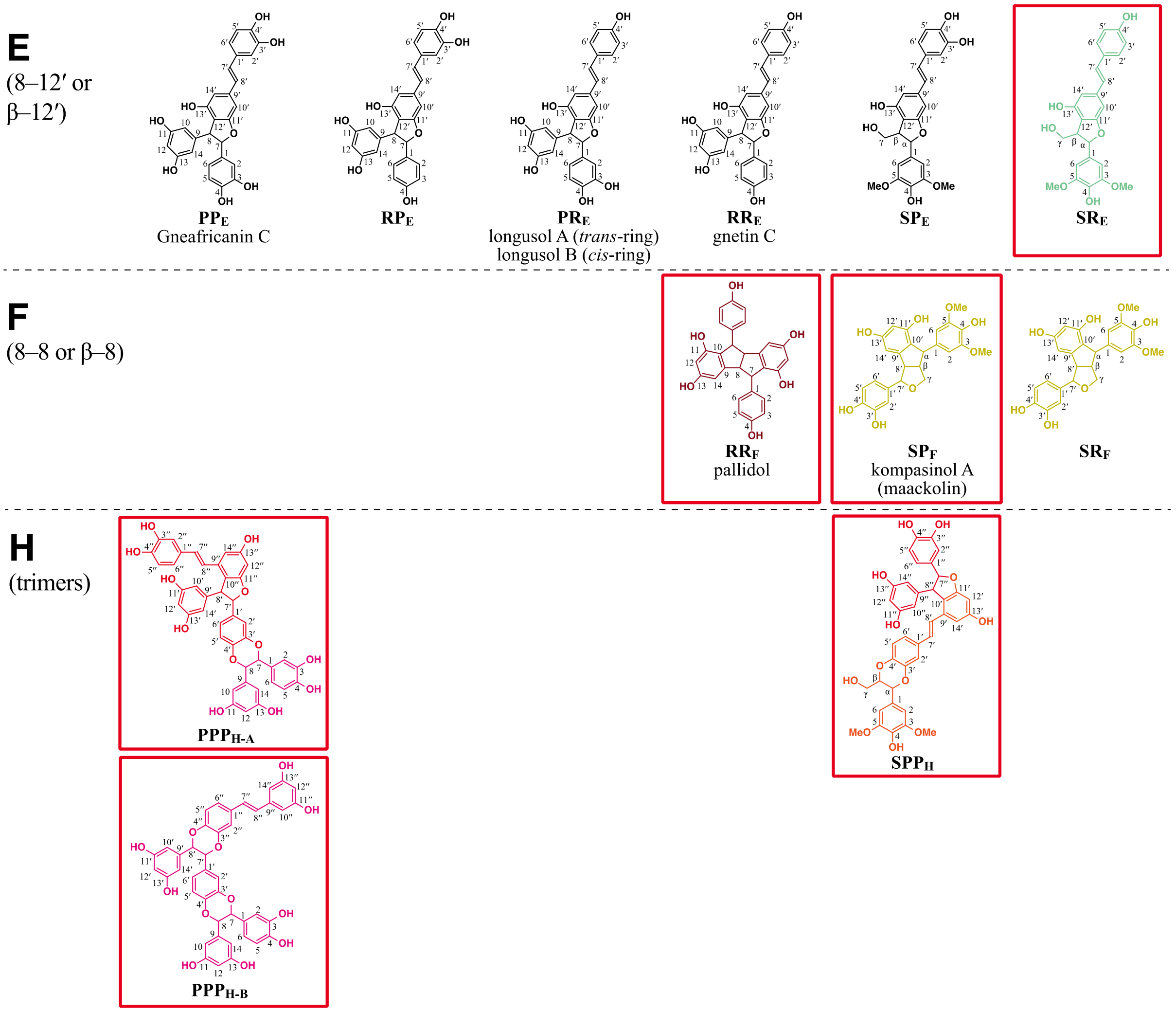

Fig. S4-2. Hydroxystilbene structures. (Continued from fig. S4-1)

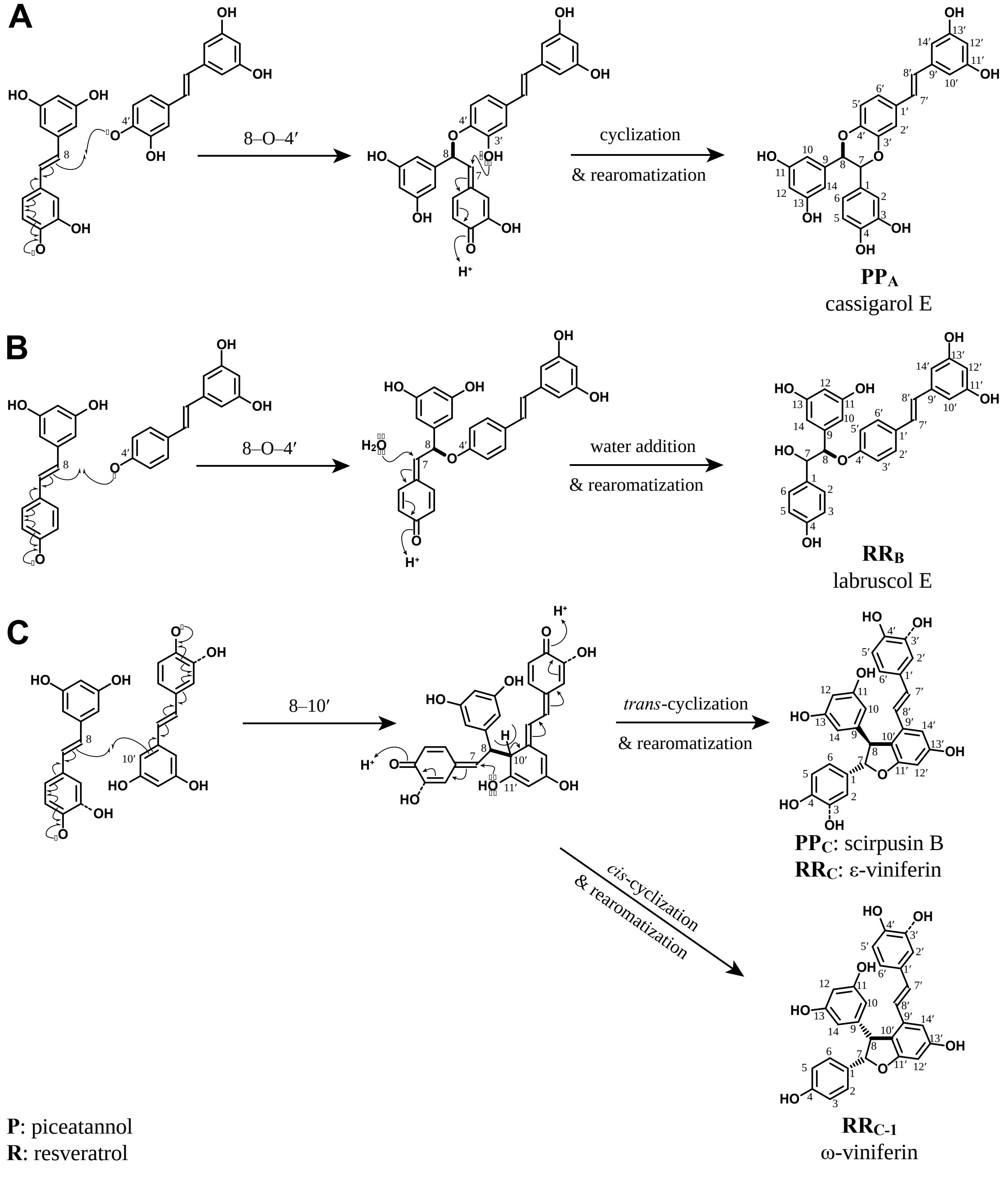

Fig. S5-1. Radical coupling mechanism to produce hydroxystilbene dimers. (A) Piceatannol 8–O–4′-coupling to produce **PP$_A$**, cassigarol E. (B) Resveratrol dimerization by 8–O–4′-coupling to generate **RR$_B$**, labruscol E. (C) Hydroxystilbene dimerization by 8–10′-coupling to generate **PP$_C$**, scirpusin B, from piceatannol and **RR$_C$**, ε-viniferin, from resveratrol. Although **RR$_C$** has a *trans*-form in its coumaran ring structure, **RR$_{C-1}$**, ω-viniferin, is the *cis* form.

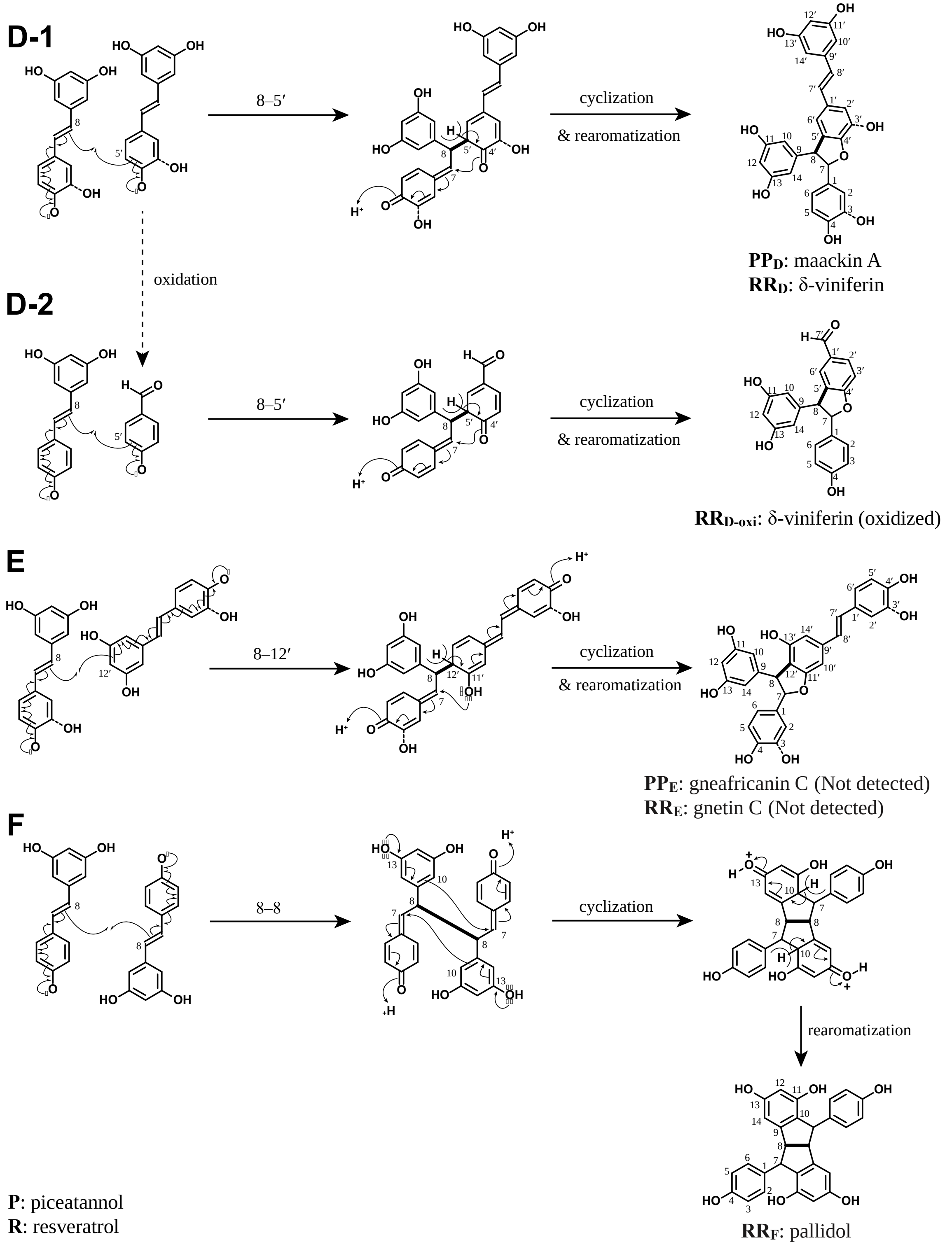

Fig. S5-2. Radical coupling mechanism to produce hydroxystilbene dimers (continued). (D-1) Hydroxystilbene dimerization by 8–5′-coupling to generate **PP$_{D}$**, maackin A, from piceatannol and **RR$_{D}$**, δ-viniferin, from resveratrol. (D-2) Resveratrol was oxidized and cleaved; the double bond between 7′ and 8′, and the *p*-hydroxybenzaldehyde couples with a resveratrol monomer to produce **RR$_{D\text{-}oxi}$**. (E) Hydroxystilbene dimerization by 8–12′-coupling to generate **PP$_{E}$**, gneafricanin C, from piceatannol and **RR$_{E}$**, gnetin C, from resveratrol. Both structures were not able to be isolated from the *in vitro* reaction in this study, but they have been found as natural compounds. (F) Reveratrol dimerization by 8–8-coupling to generate **RR$_{F}$**, pallidol, which has a symmetrical structure.

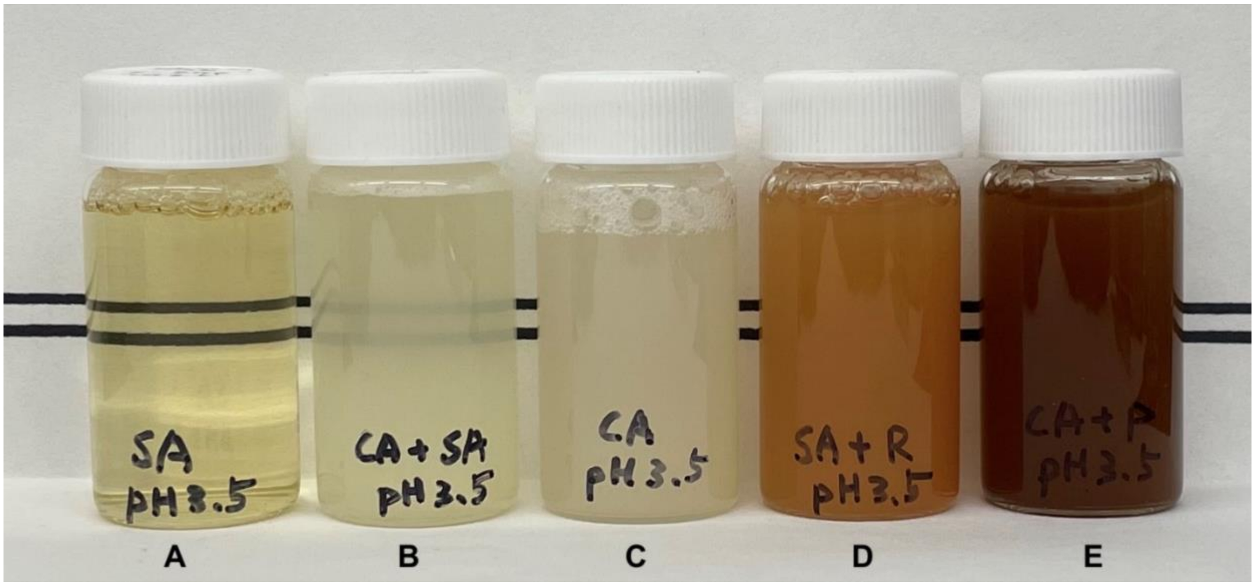

Fig. S6. DHPs (dehydrogenation polymers) from monolignols and hydroxystilbenes. (A) Attempted sinapyl alcohol-only S-DHP production did not generate collectible precipitate. (B and C) Monolignol DHPs have cloudy beige colors, with the coniferyl alcohol-only G-DHP presenting the cloudiest solution among them. (D and E) All DHPs of monolignols with hydroxystilbenes provided good polymers yields and were dark brown colors.

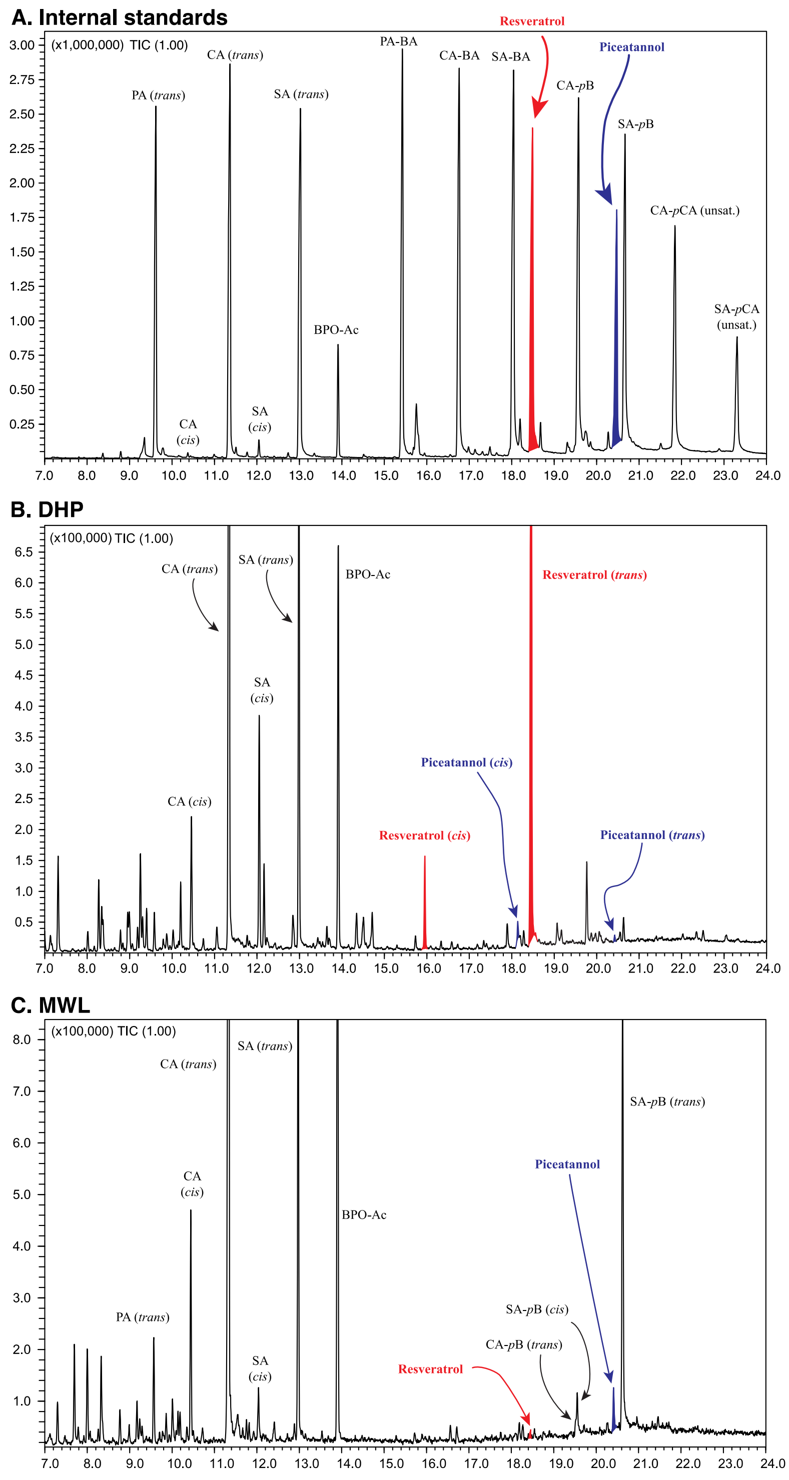

Fig. S7. Hydroxystilbenes released from lignins by DFRC (derivatization followed by reductive cleavage) analysis. (A) Internal standards chromatogram shows that hydroxystilbenes can be eluted differentially with good resolution from other peaks through GC/MS, along with the other standard compounds. (B) Total-ion chromatogram of the DFRC degradation products released from the PRGS-DHP. (C) The MWL from carnauba fruit endocarps shows the presence of piceatannol and resveratrol. All DFRC products are released as their acetate derivatives. **PA**, *p*-coumaryl alcohol; **CA**, coniferyl alcohol; **SA**, sinapyl alcohol; **BPO-Ac**, acetylated 4,4′-ethylidenebisphenol; **PA-BA**, *p*-coumaryl benzoate; **CA-BA**, coniferyl benzoate; **SA-BA**, sinapyl benzoate; **CA-*p*B**, coniferyl *p*-hydroxybenzoate; **SA-*p*B**, sinapyl *p*-hydroxybenzoate; **CA-*p*CA**, coniferyl *p*-coumarate; **SA-*p*CA**, sinapyl *p*-coumarate; **P**, piceatannol; **R**, resveratrol.

Table S1. PPP$_{H\text{-}A}$: (*E*)-4-(2-(3-(3,5-Dihydroxyphenyl)-2-(3-(3,4-dihydroxyphenyl)-2-(3,5-dihydroxyphenyl)-2,3-dihydrobenzo[*b*][1,4]dioxin-6-yl)-6-hydroxy-2,3-dihydrobenzofuran-4-yl)vinyl)benzene-1,2-diol

(MeOH-*d*$_4$)

Carbon #	^{13}C (ppm)	^{1}H (ppm)	Coupling constants
2	115.9	6.62	
5	115.9	6.62	
6	120.6	6.43	8.1, 2.0 Hz (dd)
7	82.0	4.67	(br s)
8	82.0	4.67	(br s)
10	107.3	6.12	
12	102.4	6.16	
14	107.3	6.12	
2′	113.7	6.75	
5′	116.3	6.72	
6′	118.5	6.63	
7′	94.9	5.28	6.0 Hz (d)
8′	58.2	4.34	6.0 Hz (d)
10′	107.3	6.07	2.2 Hz (d)
12′	103.4	6.12	
14′	107.3	6.07	2.2 Hz (d)
2″	115.9	6.84	
5″	118.0	6.83	
6″	120.8	6.77	
7″	130.3	6.83	
8″	124.9	6.63	
12″	97.0	6.26	2.0 Hz (d)
14″	104.5	6.63	

(**Table S1. PPP$_{\mathbf{H\text{-}A}}$**)

(DMSO-d_6:pyridine-d_5, 4:1, v/v)

Carbon #	^{13}C (ppm)	^{1}H (ppm)	Coupling constants
2	115.4	6.78	
5	115.2	6.69	8.1 Hz (d)
6	119.3	6.53	8.3 Hz (br d)
7	79.6	4.91	m
8	79.6	4.91	m
10	105.3	6.18	
12	101.3	6.22	
14	105.3	6.18	
2′	112.6	6.84	
5′	115.4	6.78	
6′	116.5	6.63	6.3, 1.7 Hz (dd)
7′	92.7	5.34	4.3 Hz (d)
8′	55.3	4.52	4.3 Hz (d)
10′	106.2	6.21	2.2 Hz (d)
12′	102.8	6.24	
14′	106.2	6.21	2.2 Hz (d)
2″	114.6	7.02	1.5 Hz (d)
5″	116.7	6.87	8.3 Hz (d)
6″	119.5	6.92	8.3, 1.5 Hz (dd)
7″	128.8	6.95	16.2 Hz (d)
8″	123.8	6.80	
12″	96.1	6.39	1.5 Hz (d)
14″	103.6	6.76	

Table S2. PPP$_{H\text{-}B}$: (*E*)-5,5'-(3'-(3,4-Dihydroxyphenyl)-7-(3,5-dihydroxystyryl)-2,2',3,3'-tetrahydro-[2,6'-bibenzo[*b*][1,4]dioxine]-2',3-diyl)bis(benzene-1,3-diol)

(MeOH-d_4)

Carbon #	^{13}C (ppm)	^{1}H (ppm)	Coupling constants
2	115.9	6.61	
5	115.9	6.61	
6	120.6	6.42	8.2 Hz (br d)
7	82.0	4.66	(m)
8	82.0	4.66	(m)
10	107.3	6.07	2.0 Hz (d)
12	103.4	6.13	
14	107.3	6.07	2.0 Hz (d)
2′	117.4	6.84	
5′	117.4	6.84	
6′	122.1	6.65	
7′	81.6	4.85	8.0 Hz (d)
8′	82.0	4.78	8.0 Hz (d)
10′	107.5	6.15	2.2 Hz (d)
12′	103.7	6.17	
14′	107.5	6.15	2.2 Hz (d)
2″	115.7	7.15	1.5 Hz (d)
5″	118.0	6.95	
6″	121.0	7.08	8.5, 1.5 Hz (dd)
7″	129.0	6.95	
8″	128.5	6.88	
10″	105.8	6.45	
12″	102.8	6.16	
14″	105.8	6.45	

(**Table S2. PPP$_{H\text{-}B}$**)

(DMSO-d_6:pyridine-d_5, 4:1, v/v)

Carbon #	^{13}C (ppm)	^{1}H (ppm)	Coupling constants
2	115.5	6.76	
5	115.2	6.67	8.1 Hz (d)
6	119.3	6.50	
7	79.6	4.82	(m)
8	79.6	4.82	(m)
10	106.2	6.18	
12	102.8	6.24	
14	106.2	6.18	
2′	116.3	6.85	
5′	116.7	7.02	
6′	121.3	6.84	
7′	78.9	5.12	
8′	78.9	5.12	
10′	106.4	6.32	
12′	102.8	6.29	
14′	106.4	6.32	
2″	114.4	7.26	1.7 Hz (d)
5″	116.7	6.96	8.2 Hz (d)
6″	120.0	7.12	8.2 Hz (br d)
7″	127.5	7.03	
8″	127.5	7.03	
10″	104.5	6.55	
12″	102.1	6.31	
14″	104.5	6.55	

Table S3. SPP$_H$: (*E*)-4-(3-(3,5-Dihydroxyphenyl)-6-hydroxy-4-(2-(3-(4-hydroxy-3,5-dimethoxyphenyl)-2-(hydroxymethyl)-2,3-dihydrobenzo[*b*][1,4]dioxin-6-yl)vinyl)-2,3-dihydrobenzofuran-2-yl)benzene-1,2-diol

(MeOH-d_4)

Carbon #	^{13}C (ppm)	^{1}H (ppm)	Coupling constants
2	105.9	6.71	s
6	105.9	6.71	s
α	78.0	4.85	8.1, 2.1 Hz (dd)
β	80.1	4.01	(m)
γ1	62.1	3.67	12.4, 2.4 Hz (dd)
γ2	62.1	3.46	12.4, 2.1 Hz (dd)
OMe	56.7	3.85	(s)
2′	115.7	6.79	
5′	118.1	6.83	8.5 Hz (d)
6′	121.1	6.75	
7′	130.1	6.81	
8′	124.9	6.61	
12′	97.1	6.25	2.0 Hz (d)
14′	104.6	6.63	
2′′	113.8	6.75	
5′′	116.2	6.72	
6′′	118.5	6.62	
7′′	94.9	5.28	6.2 Hz (d)
8′′	58.2	4.33	6.2 Hz (d)
10′′	107.4	6.14	
12′′	102.2	6.15	
14′′	107.4	6.14	

(**Table S3. SPP$_H$**)

(DMSO-d_6:pyridine-d_5, 4:1, v/v)

Carbon #	^{13}C (ppm)	^{1}H (ppm)	Coupling constants
2	105.4	6.78	s
6	105.4	6.78	s
α	76.1	4.94	7.9, 2.1 Hz (dd)
β	78.3	4.18	(m)
γ1	60.2	3.61	
γ2	60.2	3.41	(m)
OMe	55.9	3.76	(s)
2′	114.6	7.00	1.6 Hz (d)
5′	116.7	6.88	8.4 Hz (d)
6′	119.7	6.89	8.4, 1.6 Hz (dd)
7′	128.8	6.93	16.4 Hz (d)
8′	123.6	6.77	4.4 Hz (d)
12′	96.1	6.38	1.8 Hz (d)
14′	103.6	6.75	
2″	112.7	6.83	
5″	115.5	6.78	
6″	116.5	6.62	8.1, 1.8 Hz (dd)
7″	92.7	5.34	4.4 Hz (d)
8″	55.3	4.50	4.4 Hz (d)
10″	105.4	6.18	2.7 Hz (t)
12″	101.1	6.19	2.7 Hz (d)
14″	105.4	6.18	2.7 Hz (t)

5. Data S1 (Assigned NMR spectra)

REFERENCES AND NOTES

1. W. Boerjan, J. Ralph, M. Baucher, Lignin biosynthesis. *Annu. Rev. Plant Biol.* **54**, 519–546 (2003).

2. W. D. H. Schneider, R. C. Fontana, H. M. Baudel, F. G. de Siqueira, J. Rencoret, A. Gutierrez, L. I. de Eugenio, A. Prieto, M. J. Martinez, A. T. Martinez, A. J. P. Dillon, M. Camassola, Lignin degradation and detoxification of eucalyptus wastes by on-site manufacturing fungal enzymes to enhance second-generation ethanol yield. *Appl. Energ.* **262**, 114493 (2020).

3. J. Ralph, K. Lundquist, G. Brunow, F. Lu, H. Kim, P. F. Schatz, J. M. Marita, R. D. Hatfield, S. A. Ralph, J. H. Christensen, W. Boerjan, Lignins: Natural polymers from oxidative coupling of 4-hydroxyphenyl- propanoids. *Phytochem. Rev.* **3**, 29–60 (2004).

4. R. Vanholme, B. Demedts, K. Morreel, J. Ralph, W. Boerjan, Lignin biosynthesis and structure. *Plant Physiol.* **153**, 895–905 (2010).

5. M. Campbell, R. R. Sederoff, Variation in lignin content and composition (Mechanisms of Control and Implications for the Genetic Improvement of Plants). *Plant Physiol.* **110**, 3–13 (1996).

6. A. Lourenço, J. Rencoret, C. Chemetova, J. Gominho, A. Gutierrez, J. C. del Rio, H. Pereira, Lignin composition and structure differs between xylem, phloem and phellem in *Quercus suber* L. *Front. Plant Sci.* **7**, 1612 (2016).

7. R. R. Sederoff, J. J. MacKay, J. Ralph, R. D. Hatfield, Unexpected variation in lignin. *Curr. Opin. Plant Biol.* **2**, 145–152 (1999).

8. R. Vanholme, B. De Meester, J. Ralph, W. Boerjan, Lignin biosynthesis and its integration into metabolism. *Curr. Opin. Biotechnol.* **56**, 230–239 (2019).

9. J. C. del Río, J. Rencoret, A. Gutiérrez, T. Elder, H. Kim, J. Ralph, Lignin monomers from beyond the canonical monolignol biosynthetic pathway: Another brick in the wall. *ACS Sustain Chem Eng* **8**, 4997–5012 (2020).

10. J. C. del Río, J. Rencoret, A. Gutiérrez, W. Lan, H. Kim, J. Ralph, Lignin Monomers Derived from the Flavonoid and Hydroxystilbene Biosynthetic Pathways, in *Recent Advances in Polyphenol Research*, J. Reed, V. de Freitas, S. Quideau, Eds. (Wiley-Blackwell, 2021), vol. 7, chap. 7, pp. 177–206.

11. J. Ralph, J. J. MacKay, R. D. Hatfield, D. M. O'Malley, R. W. Whetten, R. R. Sederoff, Abnormal lignin in a loblolly pine mutant. *Science* **277**, 235–239 (1997).

12. J. Ralph, C. Lapierre, F. Lu, J. M. Marita, G. Pilate, J. Van Doorsselaere, W. Boerjan, L. Jouanin, NMR evidence for benzodioxane structures resulting from incorporation of 5-hydroxyconiferyl alcohol into lignins of O-Methyltransferase-Deficient poplars. *J. Agric. Food Chem.* **49**, 86–91 (2001).

13. J. C. del Río, J. Rencoret, A. Gutiérrez, H. Kim, J. Ralph, Hydroxystilbenes are monomers in palm fruit endocarp lignins. *Plant Physiol.* **174**, 2072–2082 (2017).

14. J. Chong, A. Poutaraud, P. Hugueney, Metabolism and roles of stilbenes in plants. *Plant Sci.* **177**, 143–155 (2009).

15. J. Schröder, G. Schröder, Stilbene and chalcone synthases: Related enzymes with key functions in plant-specific pathways. *Z. Naturforsch. C. J. Biosci.* **45**, 1–8 (1990).

16. O. Yu, J. M. Jez, Nature's assembly line: Biosynthesis of simple phenylpropanoids and polyketides. *Plant J.* **54**, 750–762 (2008).

17. K. A. Roupe, C. M. Remsberg, J. A. Yáñez, N. M. Davies, Pharmacometrics of stilbenes: Seguing towards the clinic. *Curr. Clin. Pharmacol.* **1**, 81–101 (2006).

18. P. Jeandet, B. Delaunois, A. Conreux, D. Donnez, V. Nuzzo, S. Cordelier, C. Clément, E. Courot, Biosynthesis, metabolism, molecular engineering, and biological functions of stilbene phytoalexins in plants. *Biofactors* **36**, 331–341 (2010).

19. H. Piotrowska, M. Kucinska, M. Murias, Biological activity of piceatannol: Leaving the shadow of resveratrol. *Mutat. Res.* **750**, 60–82 (2012).

20. K. Xiao, H.-J. Zhang, L.-J. Xuan, J. Zhang, Y.-M. Xu, D.-L. Bai, in *Studies in Natural Products Chemistry*, R. Atta ur, Ed. (Elsevier, 2008), vol. 34, pp. 453–646.

21. B. C. Akinwumi, K. M. Bordun, H. D. Anderson, Biological activities of stilbenoids. *Int. J. Mol. Sci.* **19**, 791–725 (2018).

22. C. Li, X. Xu, Z. Tao, X. J. Wang, Y. Pan, Resveratrol dimers, nutritional components in grape wine, are selective ROS scavengers and weak Nrf2 activators. *Food Chem.* **173**, 218–223 (2015).

23. K. Shimoda, N. Kubota, D. Uesugi, H. Hamada, M. Tanigawa, H. Hamada, Synthesis and pharmacological evaluation of glycosides of resveratrol, pterostilbene, and piceatannol. *Ann. N. Y. Acad. Sci.* **1348**, 141–149 (2015).

24. M. A. Seyed, I. Jantan, S. N. A. Bukhari, K. Vijayaraghavan, A comprehensive review on the chemotherapeutic potential of piceatannol for cancer treatment, with mechanistic insights. *J. Agric. Food Chem.* **64**, 725–737 (2016).

25. R. M. Niles, C. P. Cook, G. G. Meadows, Y.-M. Fu, J. L. McLaughlin, G. O. Rankin, Resveratrol is rapidly metabolized in athymic (nu/nu) mice and does not inhibit human melanoma xenograft tumor growth. *J. Nutr.* **136**, 2542–2546 (2006).

26. M. I. Fernández-Marín, R. F. Guerrero, M. C. García-Parrilla, B. Puertas, T. Richard, M. A. Rodriguez-Werner, P. Winterhalter, J.-P. Monti, E. Cantos-Villar, Isorhapontigenin: A novel bioactive stilbene from wine grapes. *Food Chem.* **135**, 1353–1359 (2012).

27. Y. Fang, Y. Yu, Q. Hou, X. Zheng, M. Zhang, D. Zhang, J. Li, X. R. Wu, C. Huang, The Chinese herb isolate isorhapontigenin induces apoptosis in human cancer cells by down-regulating overexpression of antiapoptotic protein XIAP. *J. Biol. Chem.* **287**, 35234–35243 (2012).

28. J. Rencoret, H. Kim, A. B. Evaristo, A. Gutiérrez, J. Ralph, J. C. del Río, Variability in lignin composition and structure in cell walls of different parts of macaúba (*Acrocomia aculeata*) palm fruit. *J. Agric. Food Chem.* **66**, 138–153 (2018).

29. M. Lin, C.-S. Yao, in *Studies in Natural Products Chemistry*, R. Atta ur, Ed. (Elsevier, 2006), vol. 33, pp. 601–644.

30. C. Rivière, A. D. Pawlus, J.-M. Mérillon, Natural stilbenoids: Distribution in the plant kingdom and chemotaxonomic interest in Vitaceae. *Nat. Prod. Rep.* **29**, 1317–1333 (2012).

31. A. D. Pawlus, P. Waffo-Téguo, J. Shaver, J. Mérillon, Stilbenoid chemistry from wine and the genus *Vitis*, a review. *OENO One* **46**, 57–111 (2016).

32. M. H. Keylor, B. S. Matsuura, C. R. Stephenson, Chemistry and biology of resveratrol-derived natural products. *Chem. Rev.* **115**, 8976–9027 (2015).

33. S. A. Begum, M. Sahai, A. B. Ray, Non-conventional lignans: Coumarinolignans, flavonolignans, and stilbenolignans. *Fortschritte der Chemie organischer Naturstoffe = Progress in the chemistry of organic natural products. Progres dans la chimie des substances organiques naturelles* **93**, 1–70 (2010).

34. C. S. Chambers, K. Valentová, V. Kren, "Non-Taxifolin" derived flavonolignans: Phytochemistry and biology. *Curr. Pharm. Des.* **21**, 5849–5500 (2015).

35. C. S. Yao, M. Lin, L. Wang, Isolation and biomimetic synthesis of anti-inflammatory stilbenolignans from *Gnetum cleistostachyum*. *Chem. Pharm. Bull.* **54**, 1053–1057 (2006).

36. S.-H. Lam, S.-S. Lee, Unusual stilbenoids and a stilbenolignan from seeds of *Syagrus romanzoffiana*. *Phytochemistry* **71**, 792–797 (2010).

37. J. Rencoret, D. Neiva, G. Marques, A. Gutiérrez, H. Kim, J. Gominho, H. Pereira, J. Ralph, J. C. del Río, Hydroxystilbene glucosides are incorporated into Norway spruce bark lignin. *Plant Physiol.* **180**, 1310–1321 (2019).

38. D. M. Neiva, J. Rencoret, G. Marques, A. Gutierrez, J. Gominho, H. Pereira, J. C. del Rio, Lignin from tree barks: Chemical structure and valorization. *ChemSusChem* **13**, 4537–4547 (2020).

39. J. Ralph, P. F. Schatz, F. Lu, H. Kim, T. Akiyama, S. F. Nelsen, in *Quinone Methides*, S. Rokita, Ed. (Wiley-Blackwell, 2009), vol. 1, chap. 12, pp. 385–420.

40. R. Vanholme, K. Morreel, C. Darrah, P. Oyarce, J. H. Grabber, J. Ralph, W. Boerjan, Metabolic engineering of novel lignin in biomass crops. *New Phytol.* **196**, 978–1000 (2012).

41. J. Ralph, G. Brunow, P. J. Harris, R. A. Dixon, P. F. Schatz, W. Boerjan, in *Recent Advances in Polyphenol Research*, F. Daayf, A. El Hadrami, L. Adam, G. M. Ballance, Eds. (Wiley-Blackwell Publishing, 2008), vol. 1, chap. 2, pp. 36–66.

42. D. Lee, M. Cuendet, J. S. Vigo, J. G. Graham, F. Cabieses, H. H. S. Fong, J. M. Pezzuto, A. D. Kinghorn, A novel cyclooxygenase-inhibitory stilbenolignan from the seeds of *Aiphanes aculeata*. *Org. Lett.* **3**, 2169–2171 (2001).

43. S. Chand, M. G. Banwell, Biomimetic preparation of the racemic modifications of the stilbenolignan aiphanol and three congeners. *Aust. J. Chem.* **60**, 243–250 (2007).

44. M. Kobayashi, T. Mahmud, N. Yoshioka, K. Hori, H. Shiguya, I. Kitagawa, Indonesian medicinal plants. XVIII. Kompasinol A, a new stilbeno-phenylpropanoid from the bark of *Koompassia malaccensis* (Fabaceae). *Chem. Pharm. Bull.* **44**, 2249–2253 (1996).

45. N. I. Kulesh, V. A. Denisenko, O. B. Maksimov, Stilbenolignan from *Maackia amurensis*. *Phytochemistry* **40**, 1001–1003 (1995).

46. S.-H. Lam, J.-M. Chen, C.-J. Kang, C.-H. Chen, S.-S. Lee, α-Glucosidase inhibitors from the seeds of *Syagrus romanzoffiana*. *Phytochemistry* **69**, 1173–1178 (2008).

47. K. Freudenberg, Beiträge zur Erforschung des Lignins. *Angew. Chem.* **68**, 508–512 (1956).

48. Y. Tobimatsu, T. Takano, H. Kamitakahara, F. Nakatsubo, Reactivity of syringyl quinone methide intermediates in dehydrogenative polymerization. Part 2: pH effect in horseradish peroxidase-catalyzed polymerization of sinapyl alcohol. *Holzforschung* **64**, 183–192 (2010).

49. Y. Tobimatsu, T. Takano, H. Kamitakahara, F. Nakatsubo, Reactivity of syringyl quinone

methide intermediates in dehydrogenative polymerization I: High-yield production of synthetic lignins (DHPs) in horseradish peroxidase-catalyzed polymerization of sinapyl alcohol in the presence of nucleophilic reagents. *J. Wood Sci.* **56**, 233–241 (2010).

50. R. D. Hatfield, J. Ralph, J. H. Grabber, A potential role for sinapyl *p*-coumarate as a radical transfer mechanism in grass lignin formation. *Planta* **228**, 919–928 (2008).

51. B. Saake, D. S. Argyropoulos, O. Beinhoff, O. Faix, A comparison of lignin polymer models (DHPs) and lignins by ^{31}P NMR spectroscopy. *Phytochemistry* **43**, 499–507 (1996).

52. A. Tolbert, H. Akinosho, R. Khunsupat, A. K. Naskar, A. J. Ragauskas, Characterization and analysis of the molecular weight of lignin for biorefining studies. *Biofuels Bioprod. Biorefin.* **8**, 836–856 (2014).

53. K. Syrjänen, G. Brunow, Regioselectivity in lignin biosynthesis. The influence of dimerization and *cross-coupling Transactions **1***, 183–187 (2000).

54. J. Ralph, J. M. Marita, S. A. Ralph, R. D. Hatfield, F. Lu, R. M. Ede, J. Peng, S. Quideau, R. F. Helm, J. H. Grabber, H. Kim, G. Jimenez-Monteon, Y. Zhang, H.-J. G. Jung, L. L. Landucci, J. J. MacKay, R. R. Sederoff, C. Chapple, A. M. Boudet, in *Advances in Lignocellulosics Characterization*, D. S. Argyropoulos, Ed. (TAPPI Press, 1999), pp. 55–108.

55. H. Kim, J. Ralph, T. Akiyama, Solution-state 2D NMR of ball-milled plant cell wall gels in DMSO-d_6. *Bioenergy Res.* **1**, 56–66 (2008).

56. H. Kim, J. Ralph, Solution-state 2D NMR of ball-milled plant cell wall gels in DMSO-d_6/pyridine-d_5. *Org. Biomol. Chem.* **8**, 576–591 (2010).

57. D. Ando, F. Lu, H. Kim, A. Eugene, Y. Tobimatsu, R. Vanholme, T. Elder, W. Boerjan, J. Ralph, Incorporation of catechyl monomers into lignins: Lignification from the non-phenolic end via Diels–Alder cycloaddition? *Green Chem.* **23**, 8995–9013 (2021).

58. F. Lu, J. Ralph, Derivatization followed by reductive cleavage (DFRC method), a new method for lignin analysis: Protocol for analysis of DFRC monomers. *J. Agric. Food Chem.*

45, 2590–2592 (1997).

59. F. Lu, J. M. Marita, C. Lapierre, L. Jouanin, K. Morreel, W. Boerjan, J. Ralph, Sequencing around 5-hydroxyconiferyl alcohol-derived units in caffeic acid *O*-methyltransferase-deficient poplar lignins. *Plant Physiol.* **153**, 569–579 (2010).

60. T. Elder, L. Berstis, G. T. Beckham, M. F. Crowley, Density functional theory study of spirodienone stereoisomers in lignin. *ACS Sustain. Chem. Eng.* **5**, 7188–7194 (2017).

61. S.-C. Qi, L. Zhang, S. Kudo, K. Norinaga, J.-I. Hayashi, Theoretical study on hydrogenolytic cleavage of intermonomer linkages in lignin. *Chem. A Eur. J.* **121**, 2868–2877 (2017).

62. A. K. Sangha, J. M. Parks, R. F. Standaert, A. Ziebell, M. Davis, J. C. Smith, Radical coupling reactions in lignin synthesis: A density functional theory study. *J. Phys. Chem. B.* **116**, 4760–4768 (2012).

63. A. Beste, A. C. Buchanan, Kinetic simulation of the thermal degradation of phenethyl phenyl ether, a model compound for the β-O-4 linkage in lignin. *Chem. Phys. Lett.* **550**, 19–24 (2012).

64. S. Kim, S. C. Chmely, M. R. Nimlos, Y. J. Bomble, T. D. Foust, R. S. Paton, G. T. Beckham, Computational study of bond dissociation enthalpies for a large range of native and modified lignins. *J. Phys. Chem. Lett.* **2**, 2846–2852 (2011).

65. R. Parthasarathi, R. A. Romero, A. Redondo, S. Gnanakaran, Theoretical study of the remarkably diverse linkages in lignin. *J. Phys. Chem. Lett.* **2**, 2660–2666 (2011).

66. T. Elder, J. C. del Río, J. Ralph, J. Rencoret, H. Kim, G. T. Beckham, Radical coupling reactions of piceatannol and monolignols: A density functional theory study. *Phytochemistry* **164**, 12–23 (2019).

67. J. Ralph, H. Kim, F. Lu, J. H. Grabber, J.-C. Leplé, J. Berrio-Sierra, M. Mir Derikvand, L. Jouanin, W. Boerjan, C. Lapierre, Identification of the structure and origin of a thioacidolysis marker compound for ferulic acid incorporation into angiosperm lignins (and an indicator for

cinnamoyl-CoA reductase deficiency). *Plant J.* **53**, 368–379 (2008).

68. J. C. del Río, J. Rencoret, P. Prinsen, Á. T. Martínez, J. Ralph, A. Gutiérrez, Structural characterization of wheat straw lignin as revealed by analytical pyrolysis, 2D-NMR, and reductive cleavage methods. *J. Agric. Food Chem.* **60**, 5922–5935 (2012).

69. A. Fujimoto, Y. Matsumoto, H.-M. Chang, G. Meshitsuka, Quantitative evaluation of milling effects on lignin structure during the isolation process of milled wood lignin. *J. Wood Sci.* **51**, 89–91 (2005).

70. H. Kim, D. Padmakshan, Y. Li, J. Rencoret, R. D. Hatfield, J. Ralph, Characterization and elimination of undesirable protein residues in plant cell wall materials for enhancing lignin analysis by solution-state nuclear magnetic resonance spectroscopy. *Biomacromolecules* **18**, 4184–4195 (2017).

71. H. Kim, Q. Li, S. D. Karlen, R. Smith, R. Shi, J. Liu, C. Yang, S. Tunlaya-Anukit, J. P. Wang, H.-M. Chang, R. R. Sederoff, J. Ralph, V. Chiang, Monolignol benzoates incorporate into the lignin of transgenic *Populus trichocarpa* depleted in C3H and C4H. *ACS Sustain. Chem. Eng.* **8**, 3644–3654 (2020).

72. E. Kupče, R. Freeman, Compensated adiabatic inversion pulses: Broadband INEPT and HSQC. *J. Magn. Reson.* **187**, 258–265 (2007).

73. F. Lu, J. Ralph, The DFRC method for lignin analysis. Part 1. A new method for β-aryl ether cleavage: Lignin model studies. *J. Agric. Food Chem.* **45**, 4655–4660 (1997).

74. S. Y. Han, H. S. Lee, D. H. Choi, J. W. Hwang, D. M. Yang, J. G. Jun, Efficient total synthesis of piceatannol via (*E*)-selective Wittig-Horner reaction. *Synthetic Commun* **39**, 1425–1432 (2009).

75. T. Tsuji, T. Kataoka, M. Yoshioka, Y. Sendo, Y. Nishitani, S. Hirai, T. Maeda, W. Nagata, Synthetic studies on β-lactam antibiotics. VII. Mild removal of the benzyl ester protecting group with aluminum trichloride. *Tetrahedron Lett.* **20**, 2793–2796 (1979).

76. T. Akiyama, H. Hirofuji, S. Ozaki, $AlCl_3$–*N*,*N*-dimethylaniline: A novel benzyl and allyl ether cleavage reagent. *Bull. Chem. Soc. Jpn.* **65**, 1932–1938 (1992).

77. X. Vitrac, A. Bornet, R. Vanderlinde, J. Valls, T. Richard, J.-C. Delaunay, J.-M. Mérillon, P.-L. Teissédre, Determination of stilbenes (δ-viniferin, trans-astringin, trans-piceid, cis- and trans-resveratrol, ε-viniferin) in Brazilian wines. *J. Agric. Food Chem.* **53**, 5664–5669 (2005).

78. A. S. Dubrovina, K. V. Kiselev, Regulation of stilbene biosynthesis in plants. *Planta* **246**, 597–623 (2017).

79. K. Baba, T. Kido, M. Taniguchi, M. Kozawaqa, Stilbenoids from *Cassia garrettiana*. *Phytochemistry* **36**, 1509–1513 (1994).

80. T. Morikawa, F. Xu, H. Matsuda, M. Yoshikawa, Structures of novel norstilbene dimer, longusone A, and three new stilbene dimers, longusols A, B, and C, with antiallergic and radical scavenging activities from Egyptian natural medicine *Cyperus longus*. *Chem. Pharm. Bull.* **58**, 1379–1385 (2010).

81. W.-L. Li, K.-K. He, Y. Li, Z.-J. Hou, Total synthesis of (±)-shegansu B, gnetuhainin F, (±)-maackin A and (±)-cassigarol E. *Acta Chim. Sin.* **63**, 1607–1612 (2005).

82. K. Nakajima, H. Taguchi, T. Endo, I. Yosioka, The constituents of *Scirpus fluviatilis* (TORR.) A. GRAY. I. The structures of two new hydroxystilbene dimers, scirpusin A and B. *Chem. Pharm. Bull.* **26**, 3050–3057 (1978).

83. N. I. Kulesh, O. B. Maksimov, S. A. Fedoreev, V. A. Denisenko, V. P. Glasunov, T. V. Pokushalova, L. I. Glebko, About native components of extracts from *Maackia amurensis* wood. *Chem. Nat. Compd.* **35**, 575–579 (1999).

84. T. Shen, C.-F. Xie, X.-N. Wang, H.-X. Lou, in *Natural products: Phytochemistry, botany and metabolism of alkaloids, phenolics and terpenes*, K. G. Ramawat, J.-M. Mérillon, Eds. (Springer Berlin Heidelberg, 2013), pp. 1901–1949.

85. L. Nivelle, J. Hubert, E. Courot, N. Borie, J.-H. Renault, J.-M. Nuzillard, D. Harakat, C.

Clément, L. Martiny, D. Delmas, P. Jeandet, M. Tarpin, Cytotoxicity of labruscol, a new resveratrol dimer produced by grapevine cell suspensions, on human skin melanoma cancer cell line HT-144. *Molecules* **22**, 1941 (2017).

86. C. Ponzoni, E. Beneventi, M. R. Cramarossa, S. Raimondi, G. Trevisi, U. M. Pagnoni, S. Riva, L. Forti, Laccase-catalyzed dimerization of hydroxystilbenes. *Adv. Synth. Catal.* **349**, 1497–1506 (2007).

87. M. Sako, H. Hosokawa, T. Ito, M. Iinuma, Regioselective oxidative coupling of 4-hydroxystilbenes: Synthesis of resveratrol and ε-viniferin (*E*)-dehydrodimers. *J. Org. Chem.* **69**, 2598–2600 (2004).

88. S. S. Velu, I. Buniyamin, L. K. Ching, F. Feroz, I. Noorbatcha, L. C. Gee, K. Awang, I. A. Wahab, J.-F. F. Weber, Regio- and stereoselective biomimetic synthesis of oligostilbenoid dimers from resveratrol analogues: Influence of the solvent, oxidant, and substitution. *Chem. A Eur. J.* **14**, 11376–11384 (2008).

89. Y. Takaya, K. Terashima, J. Ito, Y.-H. He, M. Tateoka, N. Yamaguchi, M. Niwa, Biomimic transformation of resveratrol. *Tetrahedron* **61**, 10285–10290 (2005).

90. M. K. Yadav, K. Mailar, J. Nagarajappa Masagalli, S.-W. Chae, J.-J. Song, W. J. Choi, Ruthenium chloride–induced oxidative cyclization of trans-resveratrol to (±)-ε-viniferin and antimicrobial and antibiofilm activity against *Streptococcus pneumoniae*. *Front. Pharmacol.* **10**, (2019).

91. Y. Wei, P. Li, L. Ma, J. Li, Separation and purification of four stilbenes from *Vitis vinifera* L. cv. cabernet sauvignon roots through high-speed counter-current chromatography. *S. Afr. J. Enol. Vitic.* **35**, 226–233 (2014).

92. M. A. Khan, S. G. Nabi, S. Prakash, A. Zaman, Pallidol, a resveratrol dimer from *Cissus pallida*. *Phytochemistry* **25**, 1945–1948 (1986).
