# Supplementary material for: Biomimetic oxidative copolymerization of hydroxystilbenes and monolignols
Source: Sci Adv. 2023 Mar 8;9(10):eade5519. doi: 10.1126/sciadv.ade5519 (PMC9995074; doi:10.1126/sciadv.ade5519)
Supplement: Supplementary file 2 — Data S1 [file sciadv.ade5519_data_s1.zip › ade5519_Data_S1.pdf]

hkbb141X1-washed
PROTON.jr Acetone D:\\ hk 2

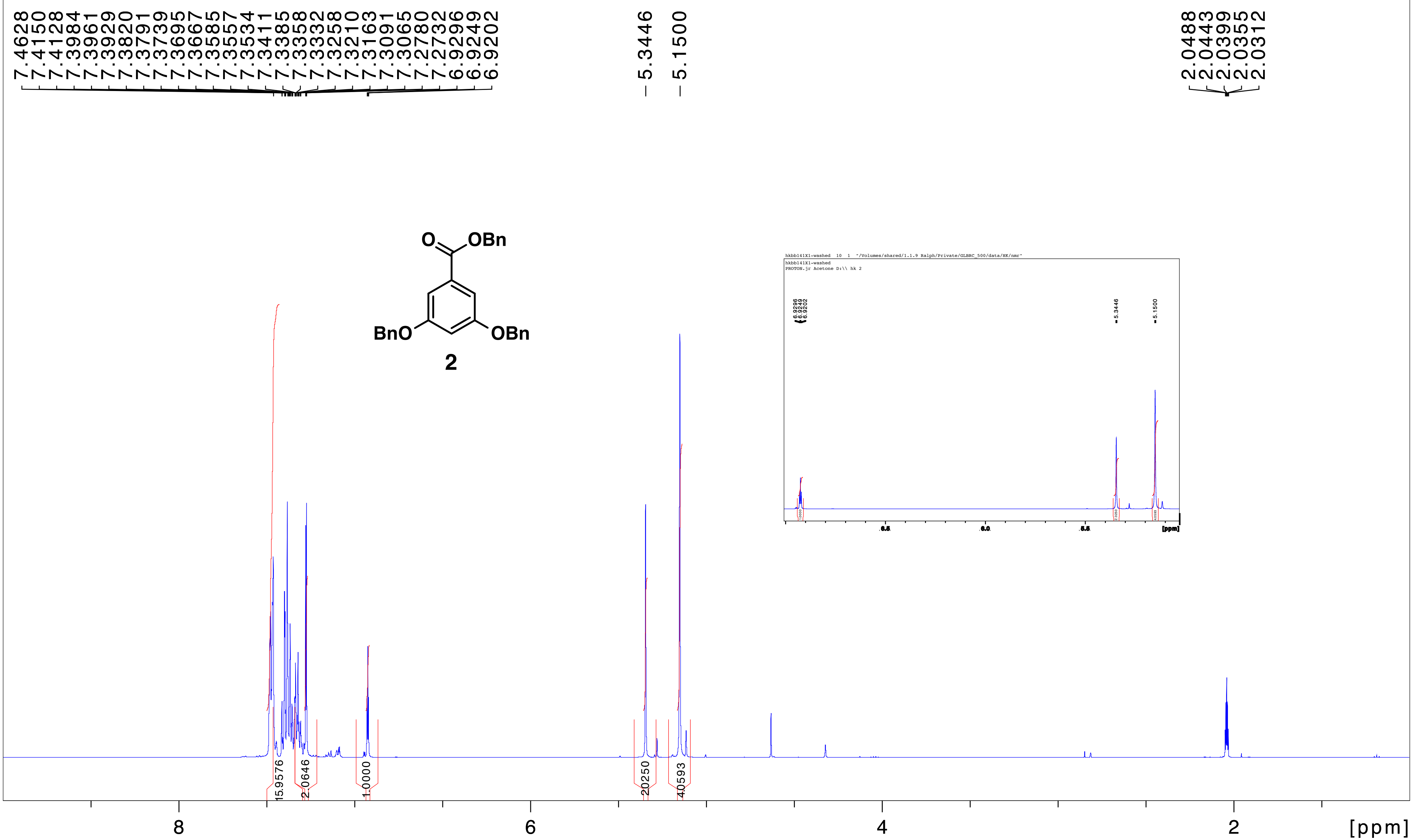

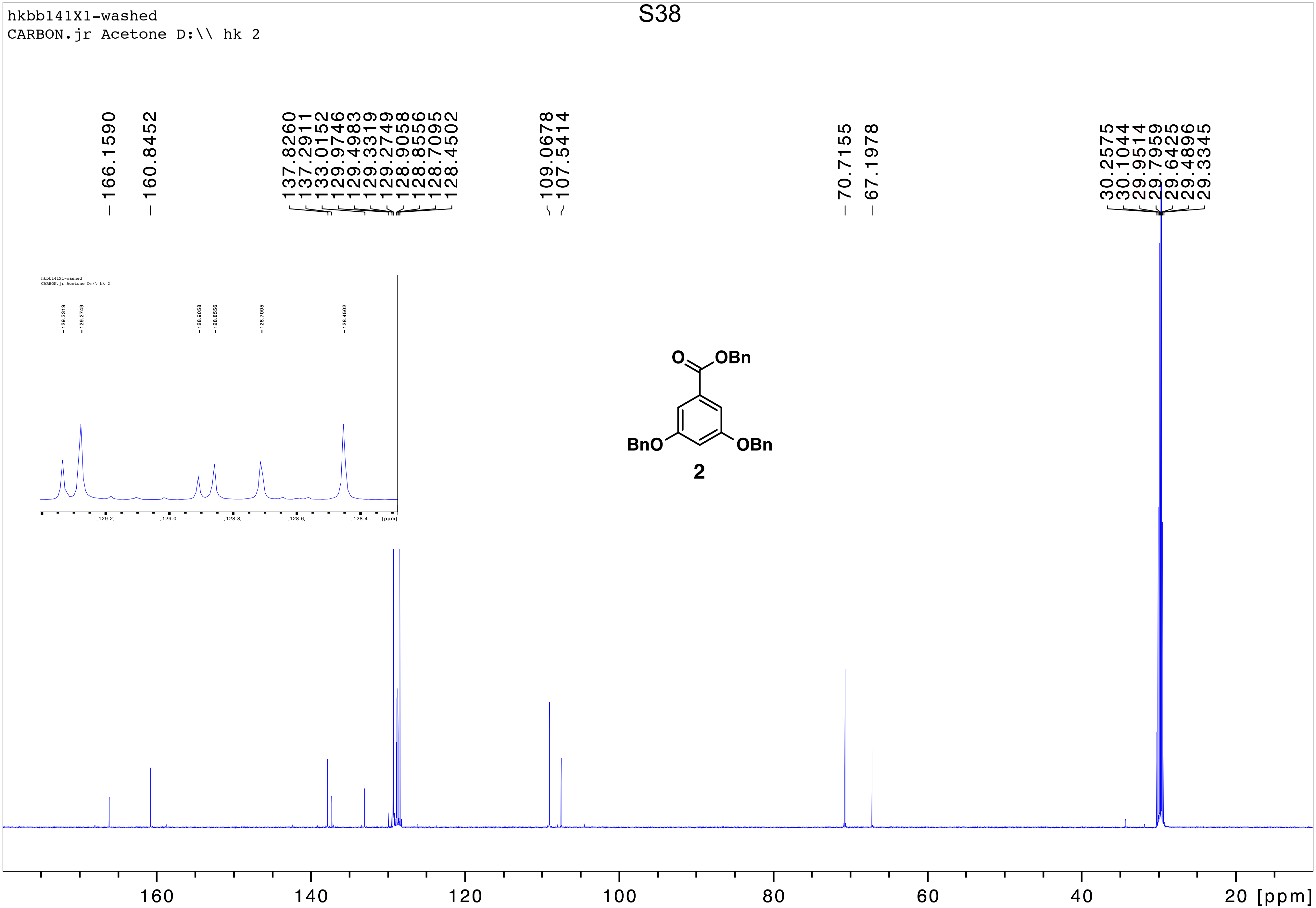
hkbb141X1-washed
CARBON.jr Acetone D:\\ hk 2
166.1590
160.8452
137.8260
137.2911
133.0152
129.9746
129.4983
129.3319
129.2749
128.9058
128.8556
128.7095
128.4502
109.0678
107.5414
70.7155
67.1978
30.2575
30.1044
29.9514
29.7959
29.6425
29.4896
29.3345
O
OBn
BnO
OBn
2
160
140
120
100
80
60
40
20 [ppm]

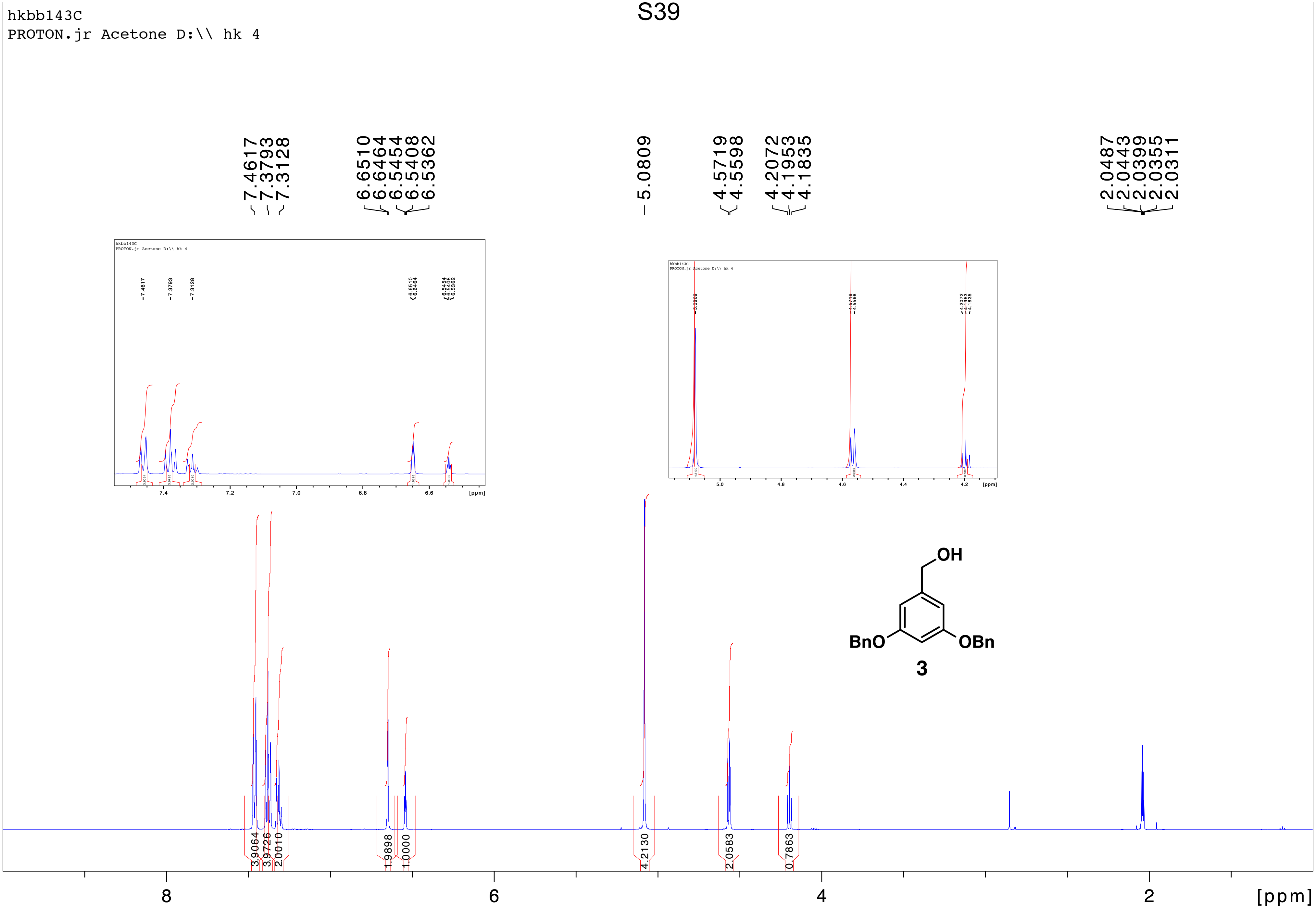
hkbb143C
PROTON.jr Acetone D:\\ hk 4
7.4617
7.3793
7.3128
6.6510
6.6464
6.5454
6.5408
6.5362
5.0809
4.5719
4.5598
4.2072
4.1953
4.1835
2.0487
2.0443
2.0399
2.0355
2.0311
3.9064
3.9726
2.0010
1.9898
1.0000
4.2130
2.0583
0.7863
8
6
4
2
[ppm]
OH
BnO
OBn

3

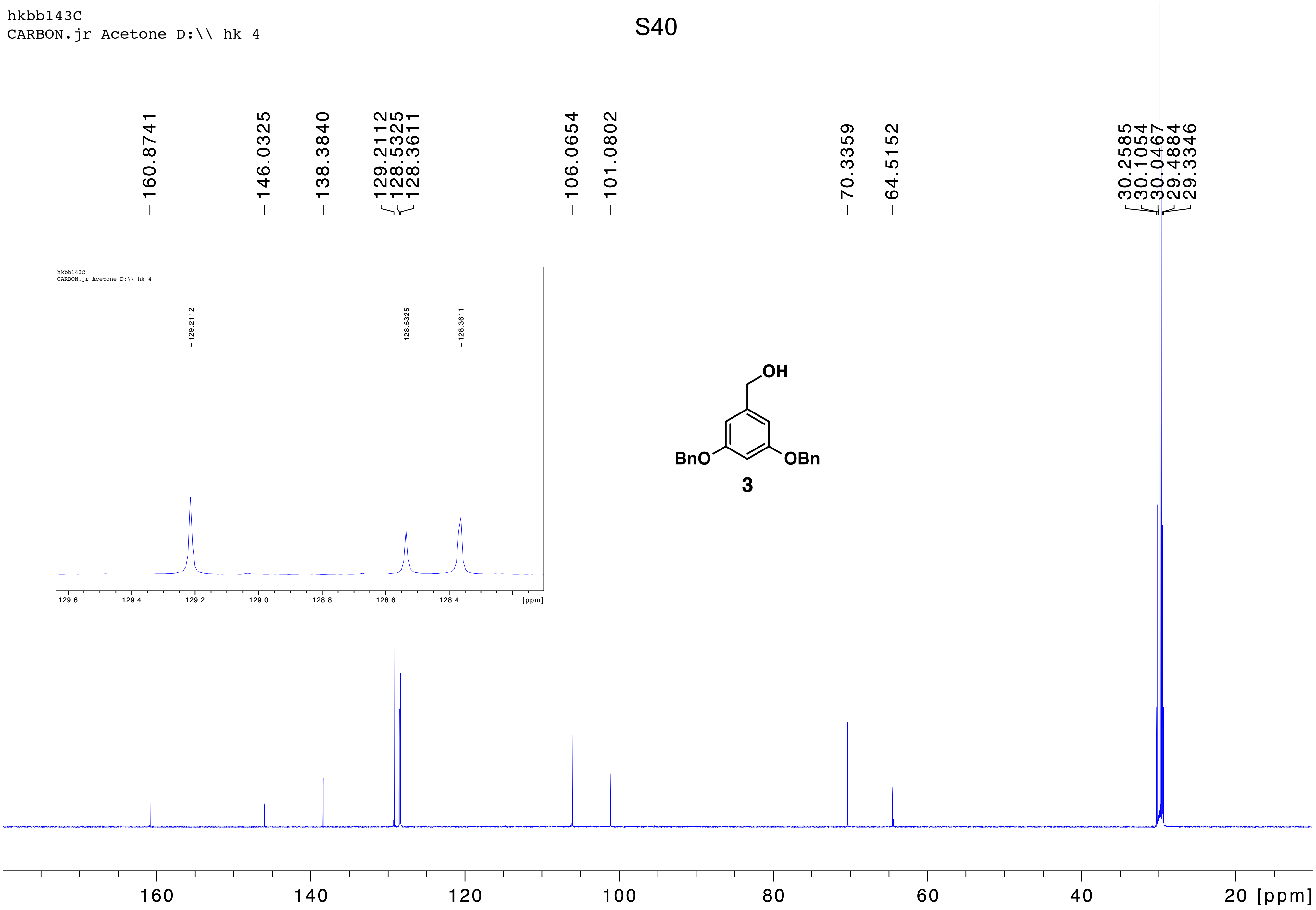
hkbb143C
CARBON.jr Acetone D:\\ hk 4
160.8741
146.0325
138.3840
129.2112
128.5325
128.3611
106.0654
101.0802
70.3359
64.5152
30.2585
30.1054
30.0467
29.4884
29.3346
129.6
129.4
129.2
129.0
128.8
128.6
128.4
[ppm]
OH
BnO
OBn
3
160
140
120
100
80
60
40
20 [ppm]

hkaa83-crude
3,5-Dibenzyloxybenzyl Bromide
PROTON.jr Acetone D:\\ hk 2

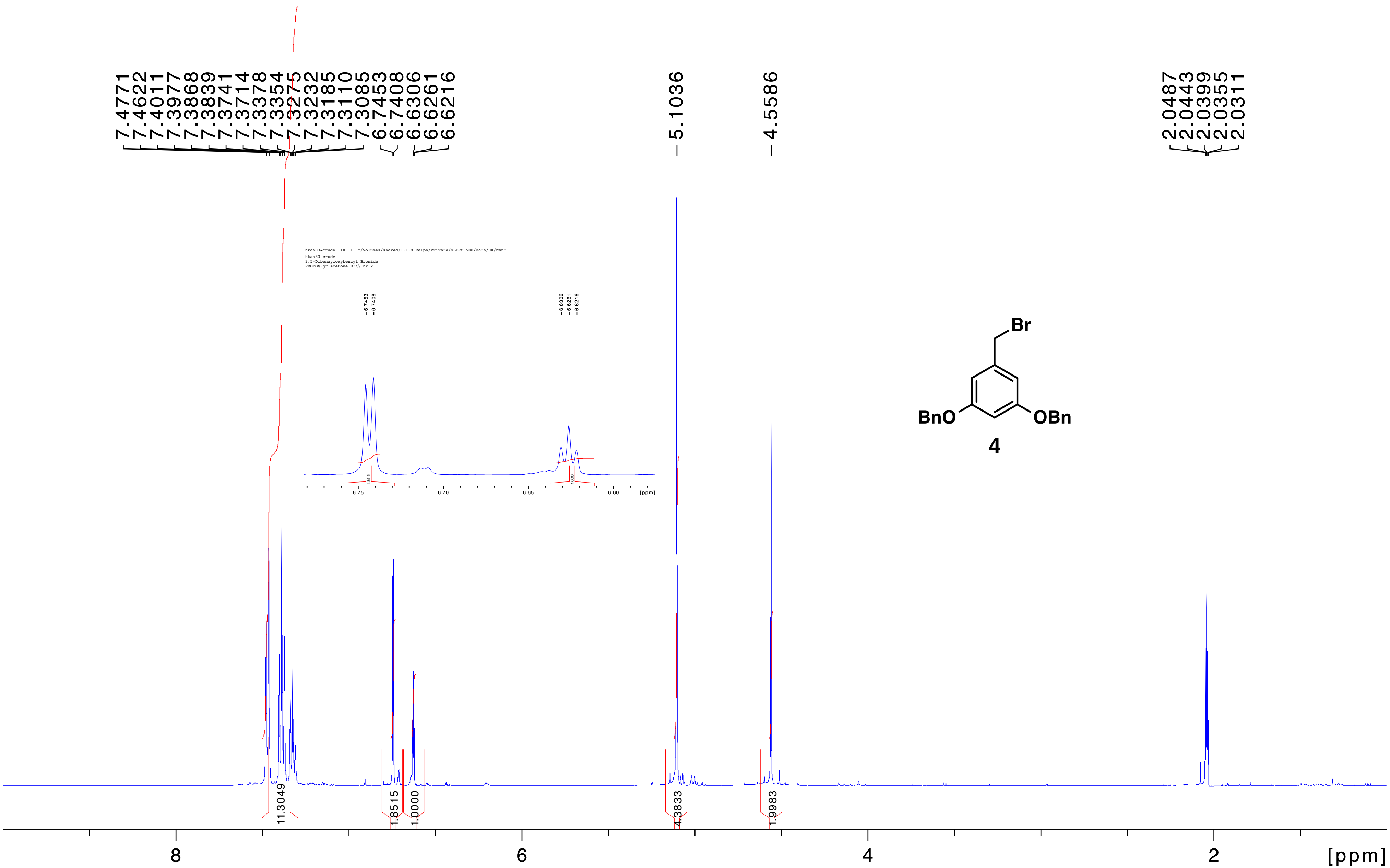

4

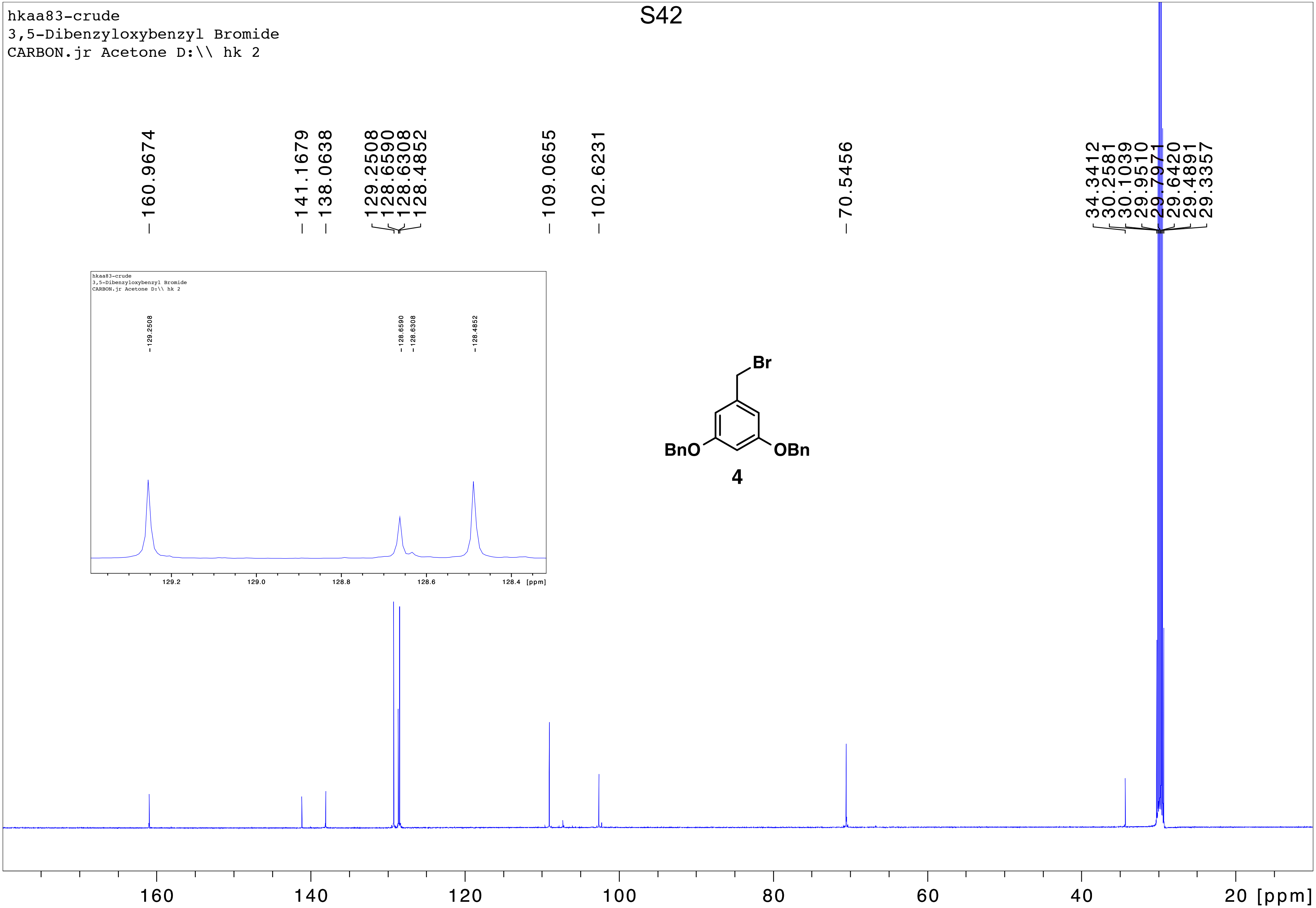
hkaa83-crude
3,5-Dibenzyloxybenzyl Bromide
CARBON.jr Acetone D:\\ hk 2
160.9674
141.1679
138.0638
129.2508
128.6590
128.6308
128.4852
109.0655
102.6231
70.5456
34.3412
30.2581
30.1039
29.9510
29.7971
29.6420
29.4891
29.3357
Br
BnO
OBn
4
160
140
120
100
80
60
40
20 [ppm]
129.2
129.0
128.8
128.6
128.4 [ppm]

hkcc5C
PROTON.jr Acetone D:\\ hk 5

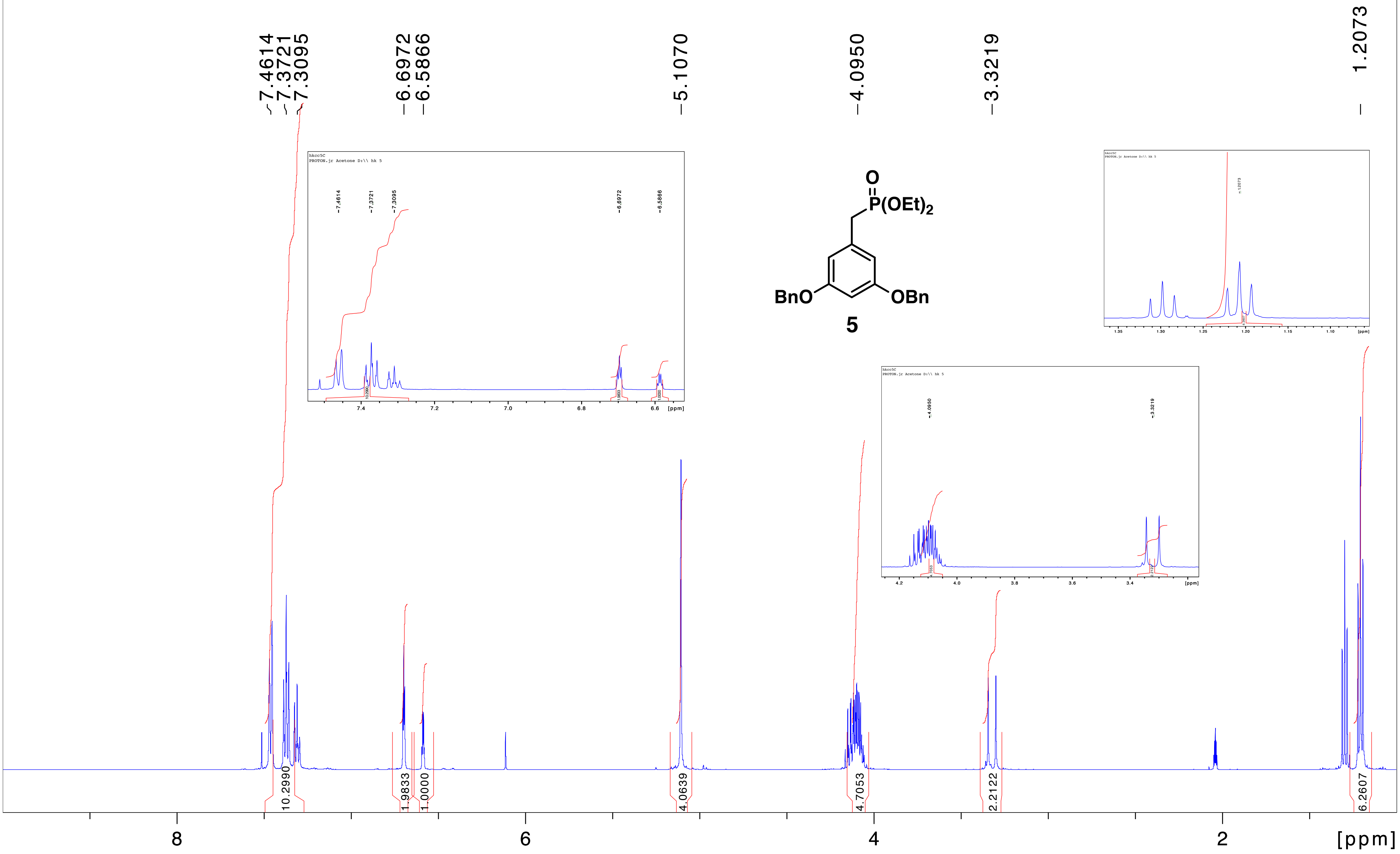

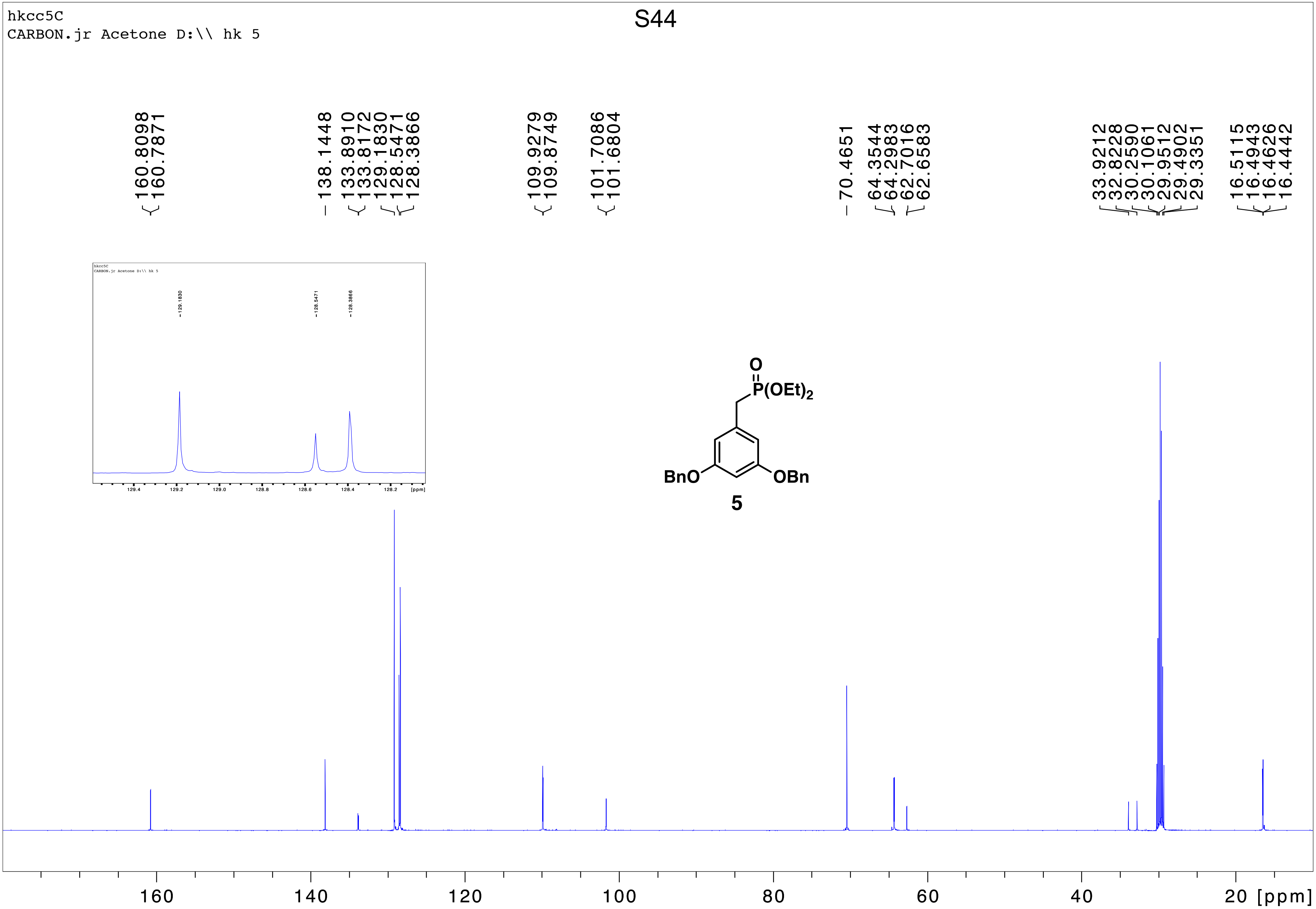
hkcc5C
CARBON.jr Acetone D:\\ hk 5
160.8098
160.7871
138.1448
133.8910
133.8172
129.1830
128.5471
128.3866
109.9279
109.8749
101.7086
101.6804
70.4651
64.3544
64.2983
62.7016
62.6583
33.9212
32.8228
30.2590
30.1061
29.9512
29.4902
29.3351
16.5115
16.4943
16.4626
16.4442
129.4
129.2
129.0
128.8
128.6
128.4
128.2
[ppm]
O
P(OEt)2
BnO
OBn
5
160
140
120
100
80
60
40
20 [ppm]

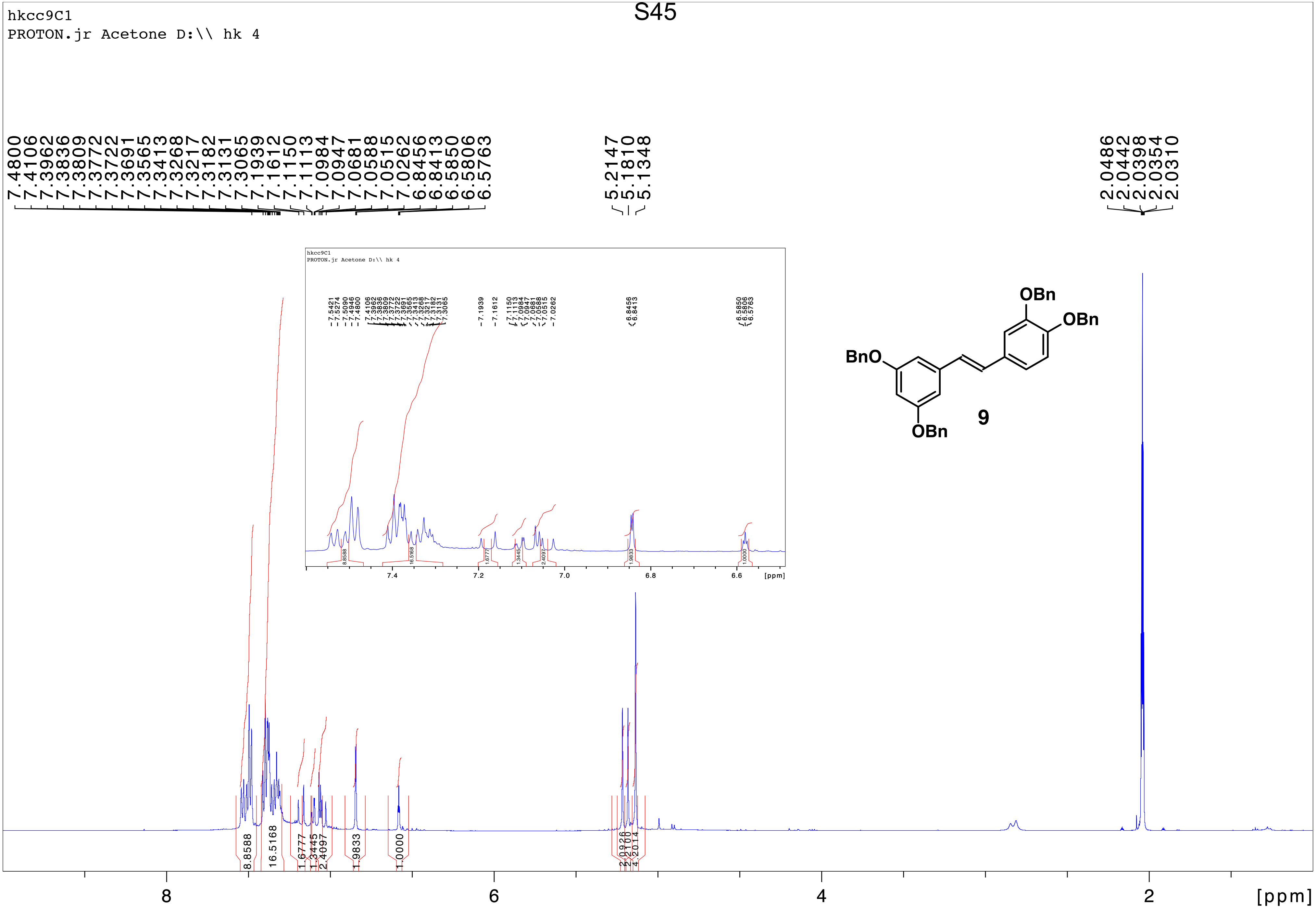
hkcc9C1
PROTON.jr Acetone D:\\ hk 4
7.4800
7.4106
7.3962
7.3836
7.3809
7.3772
7.3722
7.3691
7.3565
7.3413
7.3268
7.3217
7.3182
7.3131
7.3065
7.1939
7.1612
7.1150
7.1113
7.0984
7.0947
7.0681
7.0588
7.0515
7.0262
6.8456
6.8413
6.5850
6.5806
6.5763
5.2147
5.1810
5.1348
2.0486
2.0442
2.0398
2.0354
2.0310
8.8588
16.5168
1.6777
1.3445
2.4097
1.9833
1.0000
2.0926
2.2100
4.2014
BnO
OBn
OBn
OBn
9
8
6
4
2
[ppm]

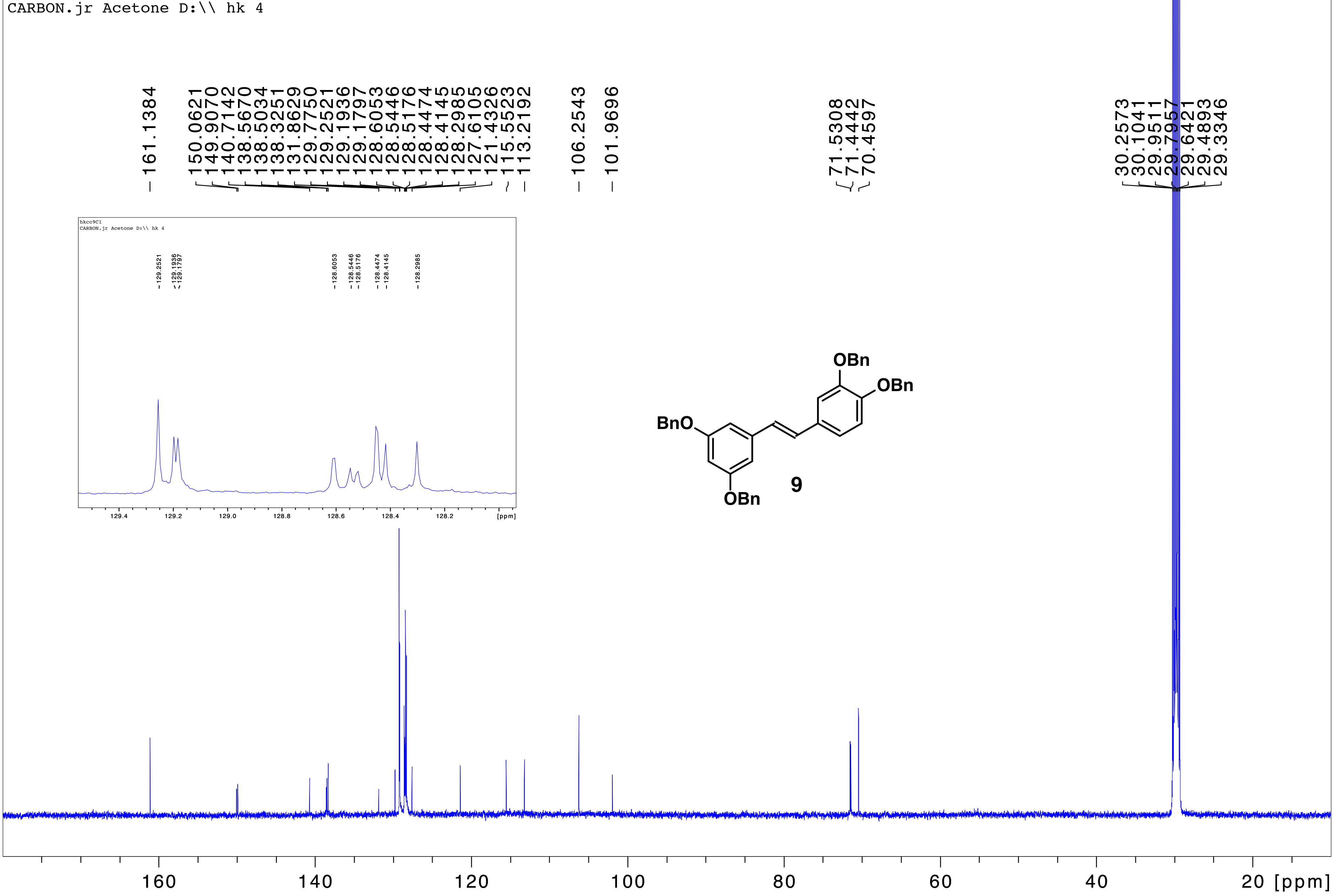
hkcc9C1
CARBON.jr Acetone D:\\ hk 4
161.1384
150.0621
149.9070
140.7142
138.5670
138.5034
138.3251
131.8629
129.7750
129.2521
129.1936
129.1797
128.6053
128.5446
128.5176
128.4474
128.4145
128.2985
127.6105
121.4326
115.5523
113.2192
106.2543
101.9696
71.5308
71.4442
70.4597
30.2573
30.1041
29.9511
29.7957
29.6421
29.4893
29.3346
BnO
OBn
OBn
OBn
9
160
140
120
100
80
60
40
20 [ppm]

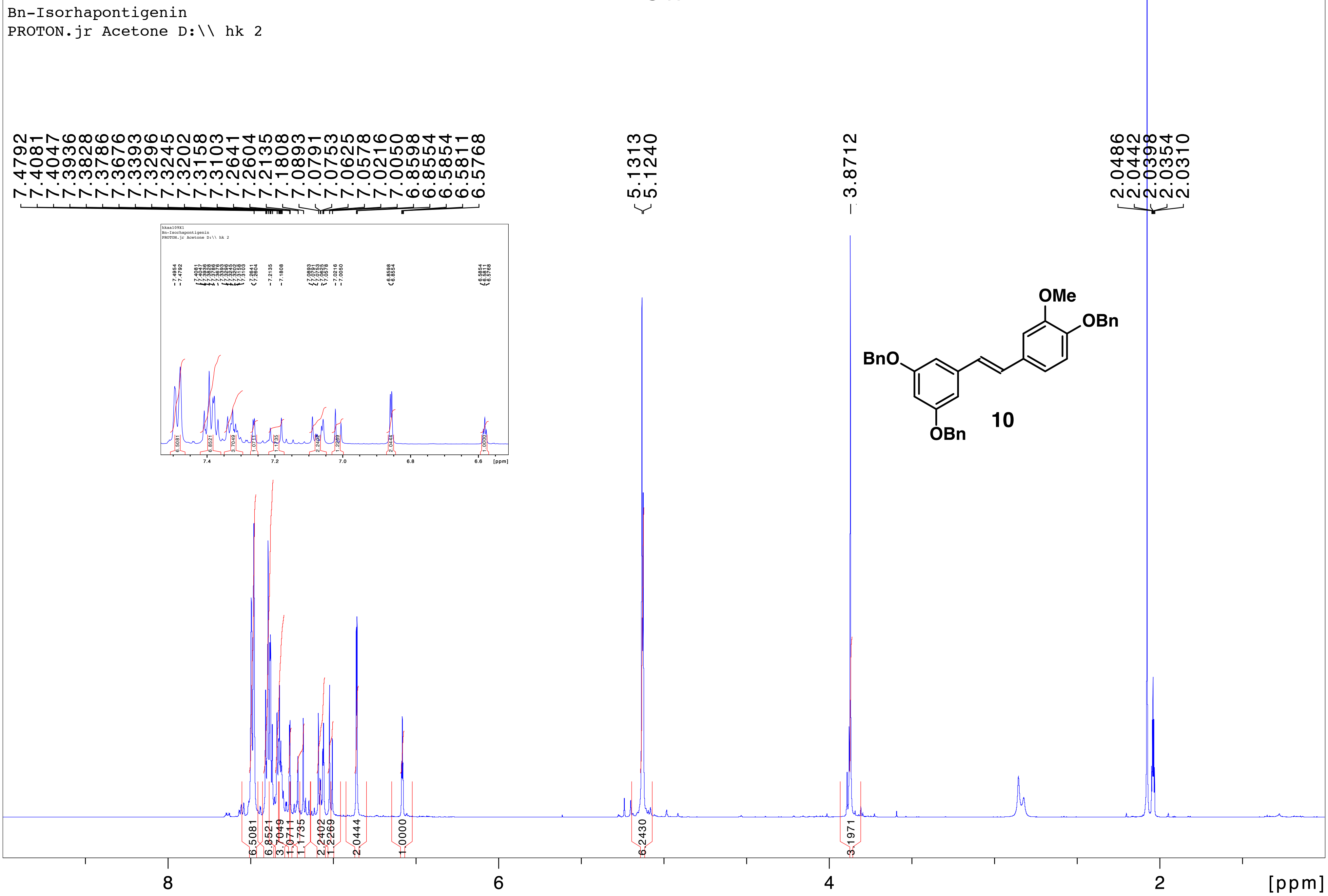
hkaa109X1
Bn-Isorhapontigenin
PROTON.jr Acetone D:\\ hk 2
7.4792
7.4081
7.4047
7.3936
7.3828
7.3786
7.3676
7.3393
7.3296
7.3245
7.3202
7.3158
7.3103
7.2641
7.2604
7.2135
7.1808
7.0893
7.0791
7.0753
7.0625
7.0578
7.0216
7.0050
6.8598
6.8554
6.5854
6.5811
6.5768
5.1313
5.1240
3.8712
2.0486
2.0442
2.0398
2.0354
2.0310
OMe
OBn
BnO
OBn
10
6.5081
6.8521
3.7049
1.0711
1.1735
2.2402
1.2269
2.0444
1.0000
6.2430
3.1971
8
6
4
2
[ppm]

hkaa109X1
Bn-Isorhapontigenin
CARBON.jr Acetone D:\\ hk 2

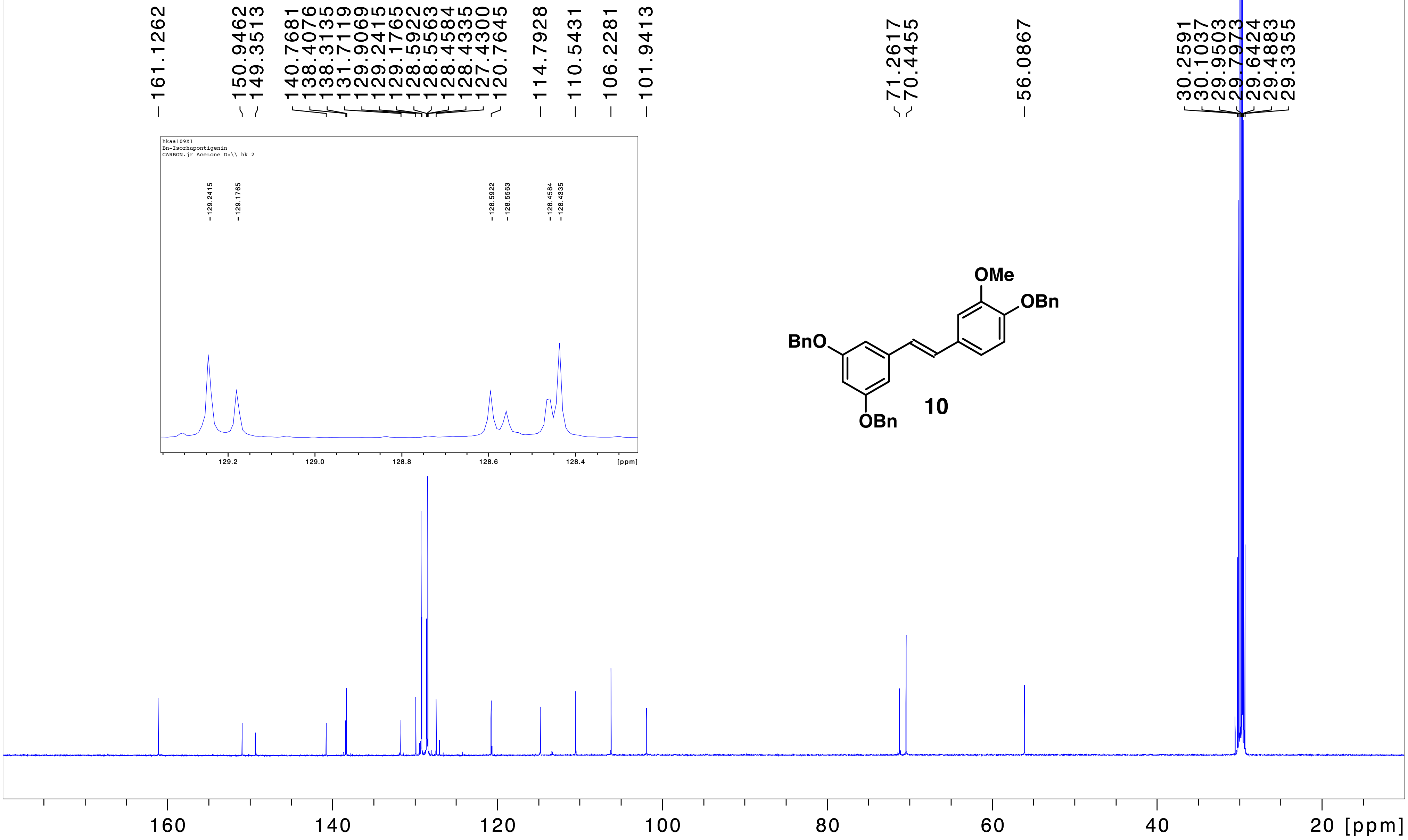

hkz53S
Piceatannol
(Acetone-d6)
PROTON.jr Acetone D:\\ hk 4

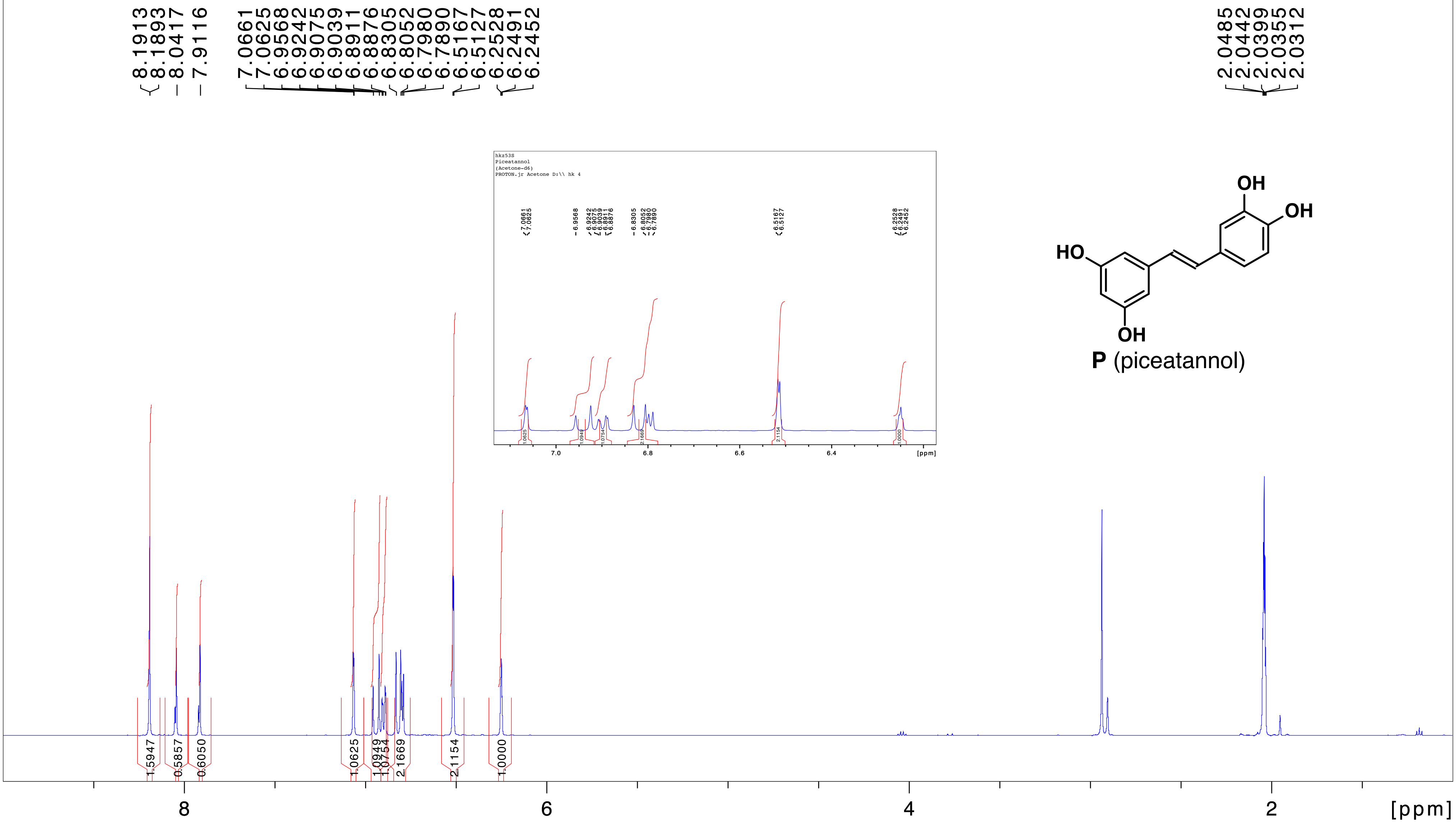

P (piceatannol)

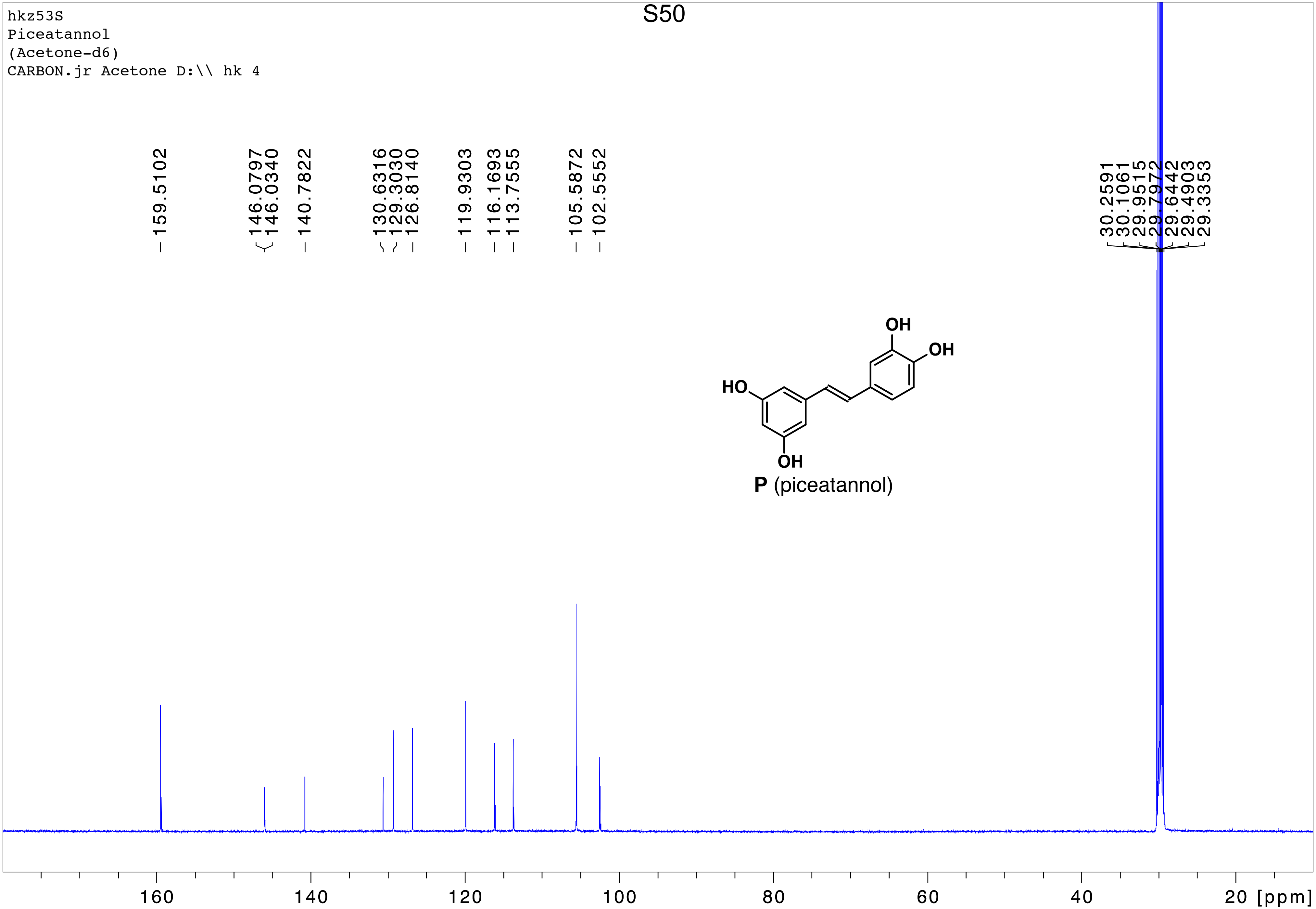
hkz53S
Piceatannol
(Acetone-d6)
CARBON.jr Acetone D:\\ hk 4
159.5102
146.0797
146.0340
140.7822
130.6316
129.3030
126.8140
119.9303
116.1693
113.7555
105.5872
102.5552
30.2591
30.1061
29.9515
29.7972
29.6442
29.4903
29.3353
HO
OH
OH
OH
P (piceatannol)
160
140
120
100
80
60
40
20 [ppm]

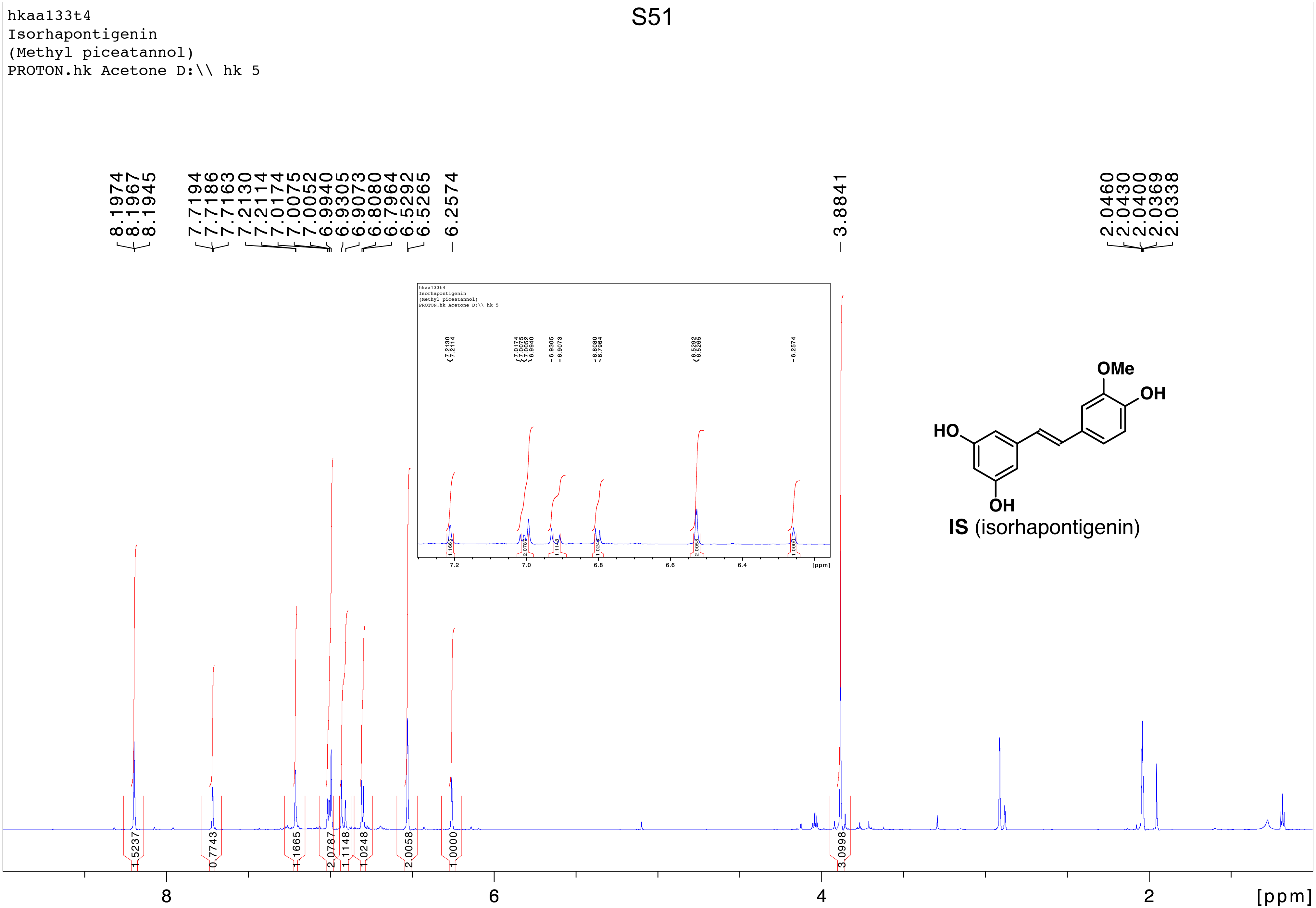

IS (isorhapontigenin)

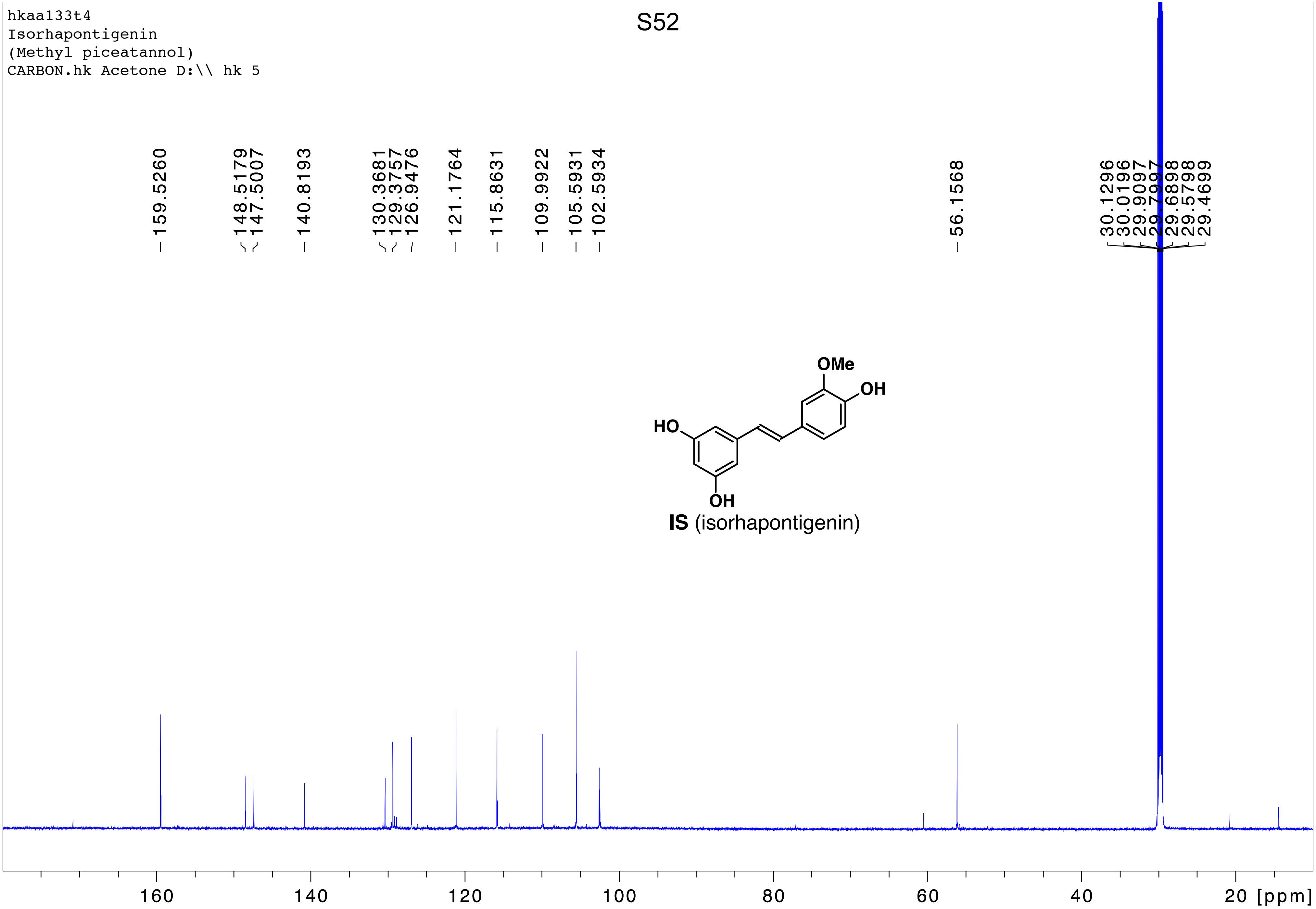
hkaa133t4
Isorhapontigenin
(Methyl piceatannol)
CARBON.hk Acetone D:\\ hk 5
159.5260
148.5179
147.5007
140.8193
130.3681
129.3757
126.9476
121.1764
115.8631
109.9922
105.5931
102.5934
56.1568
30.1296
30.0196
29.9097
29.7997
29.6898
29.5798
29.4699
OMe
OH
HO
OH
IS (isorhapontigenin)
160
140
120
100
80
60
40
20 [ppm]

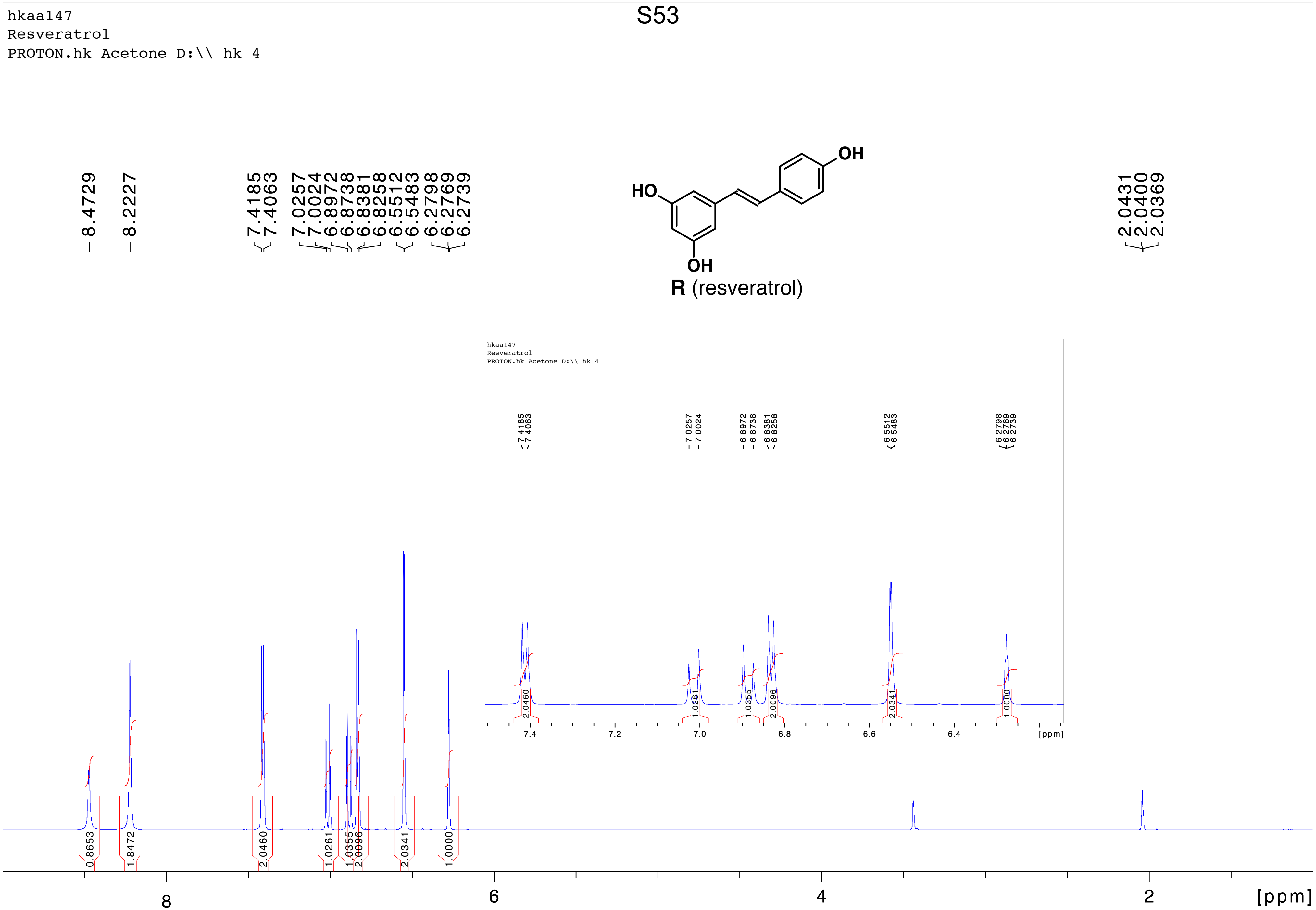
hkaa147
Resveratrol
PROTON.hk Acetone D:\\ hk 4
8.4729
8.2227
7.4185
7.4063
7.0257
7.0024
6.8972
6.8738
6.8381
6.8258
6.5512
6.5483
6.2798
6.2769
6.2739
2.0431
2.0400
2.0369
HO
OH
OH
R (resveratrol)
0.8653
1.8472
2.0460
1.0261
1.0355
2.0096
2.0341
1.0000
8
6
4
2
[ppm]
7.4
7.2
7.0
6.8
6.6
6.4

S54

hkaa147
Resveratrol
CARBON.hk Acetone D:\\ hk 4

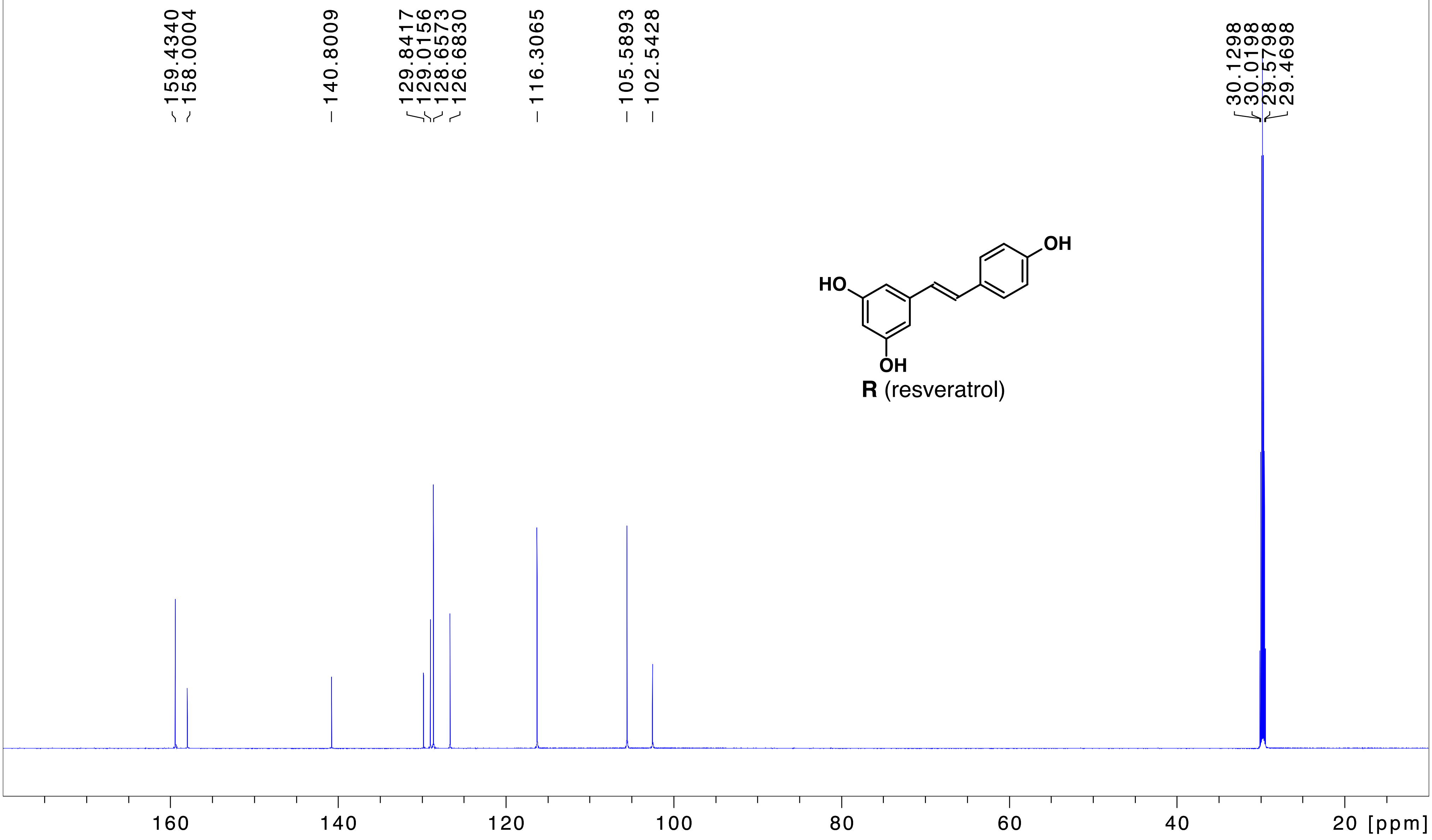

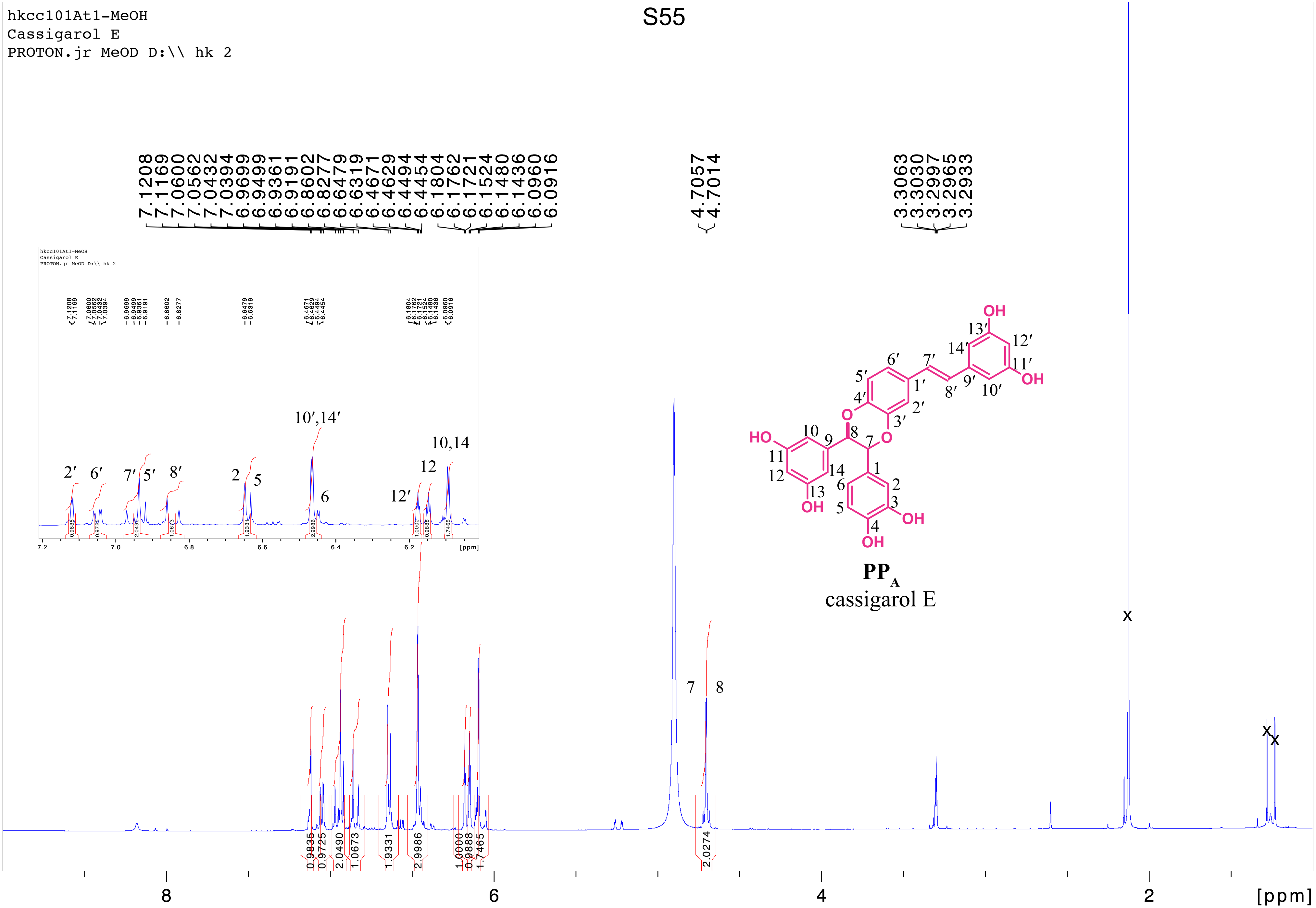

hkcc101At1-MeOH
Cassigarol E
PROTON.jr MeOD D:\\ hk 2
7.1208 7.1169 7.0600 7.0562 7.0432 7.0394 6.9699 6.9499 6.9361 6.9191 6.8602 6.8277 6.6479 6.6319 6.4671 6.4629 6.4494 6.4454 6.1804 6.1762 6.1721 6.1524 6.1480 6.1436 6.0960 6.0916
4.7057 4.7014
3.3063 3.3030 3.2997 3.2965 3.2933
2′ 6′ 7′ 5′ 8′ 2 5 10′,14′ 6 12′ 12 10,14
0.9835 0.9725 2.0490 1.0673 1.9331 2.9986 1.0000 0.9888 1.7465
7 8
2.0274
x
PP$_A$
cassigarol E
HO OH OH OH OH OH
8 6 4 2 [ppm]

hkcc101At1-MeOH
Cassigarol E
CARBON.jr MeOD D:\\ hk 2

S56

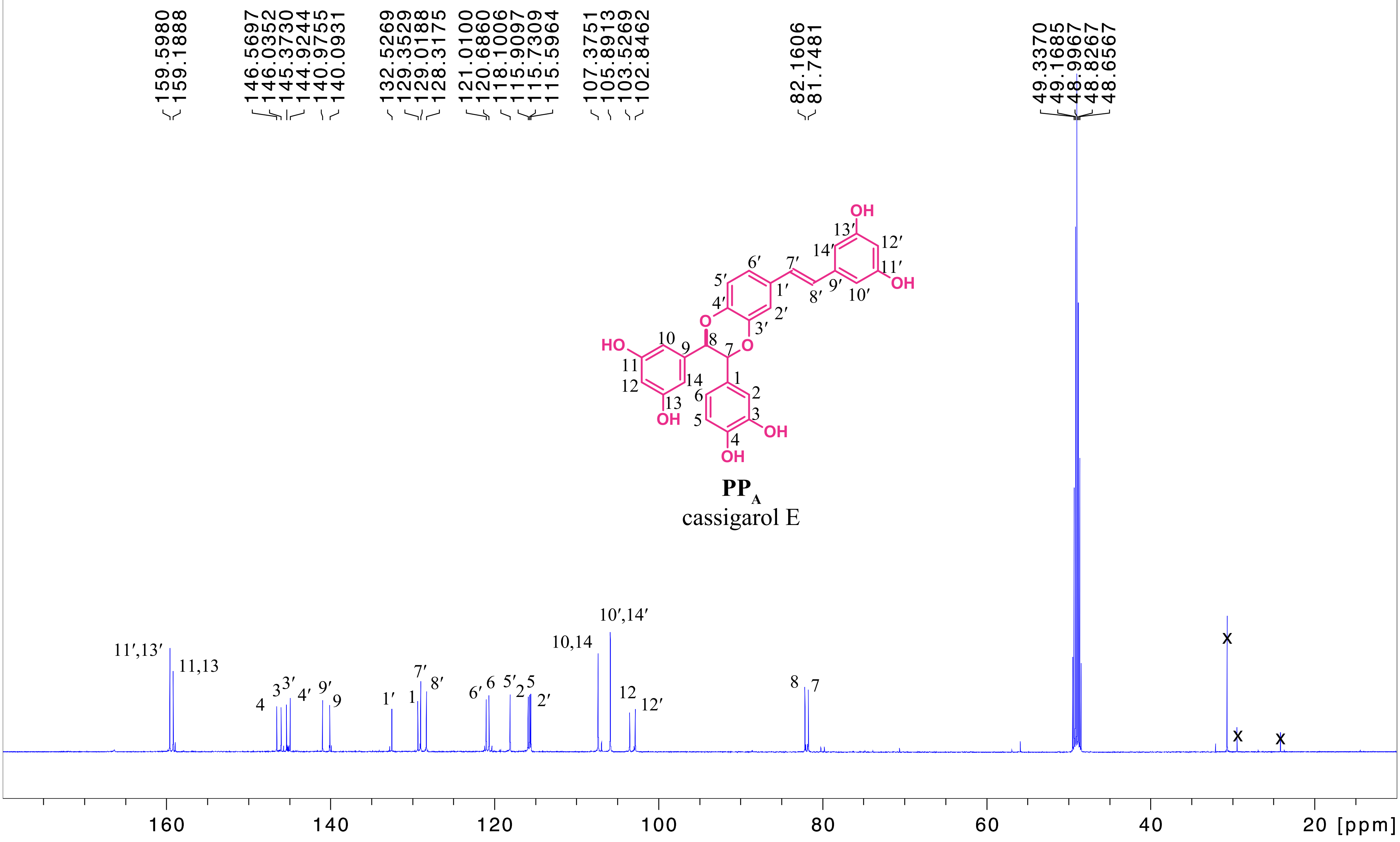

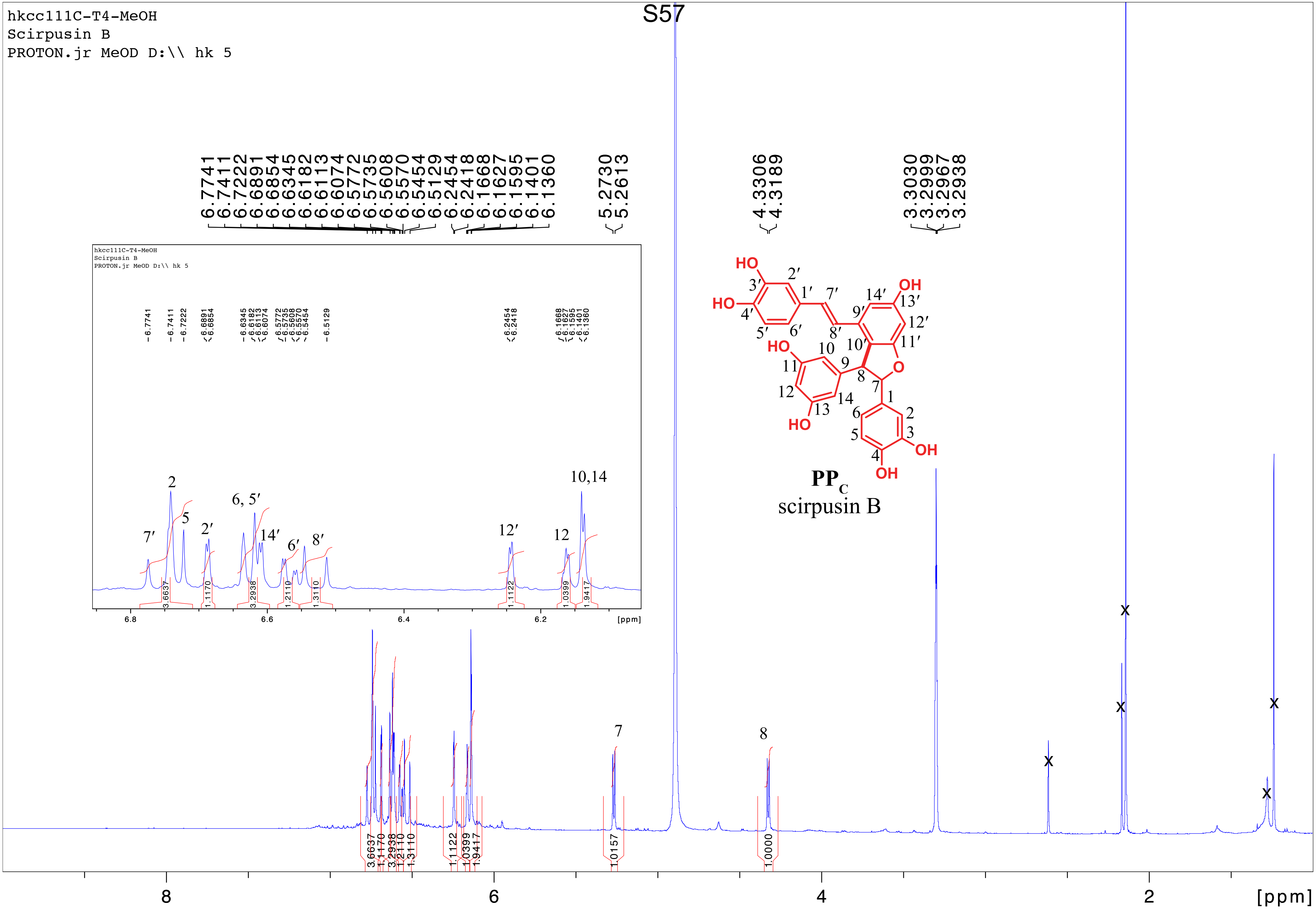
hkcc111C-T4-MeOH
Scirpusin B
PROTON.jr MeOD D:\\ hk 5
PP$_C$
scirpusin B
[ppm]

hkcc111C-T4-MeOH
Scirpusin B
CARBON.jr MeOD D:\\ hk 5

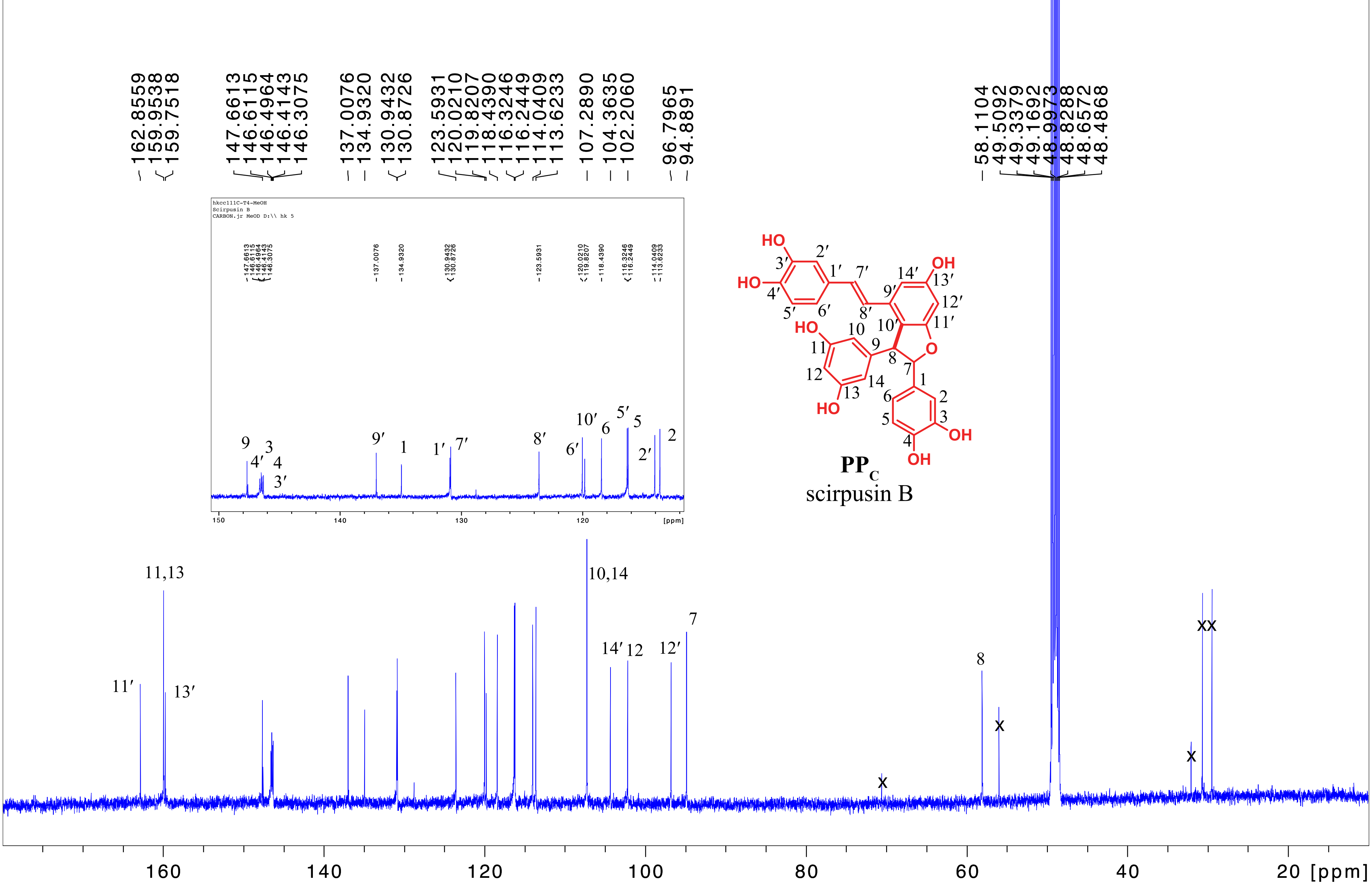

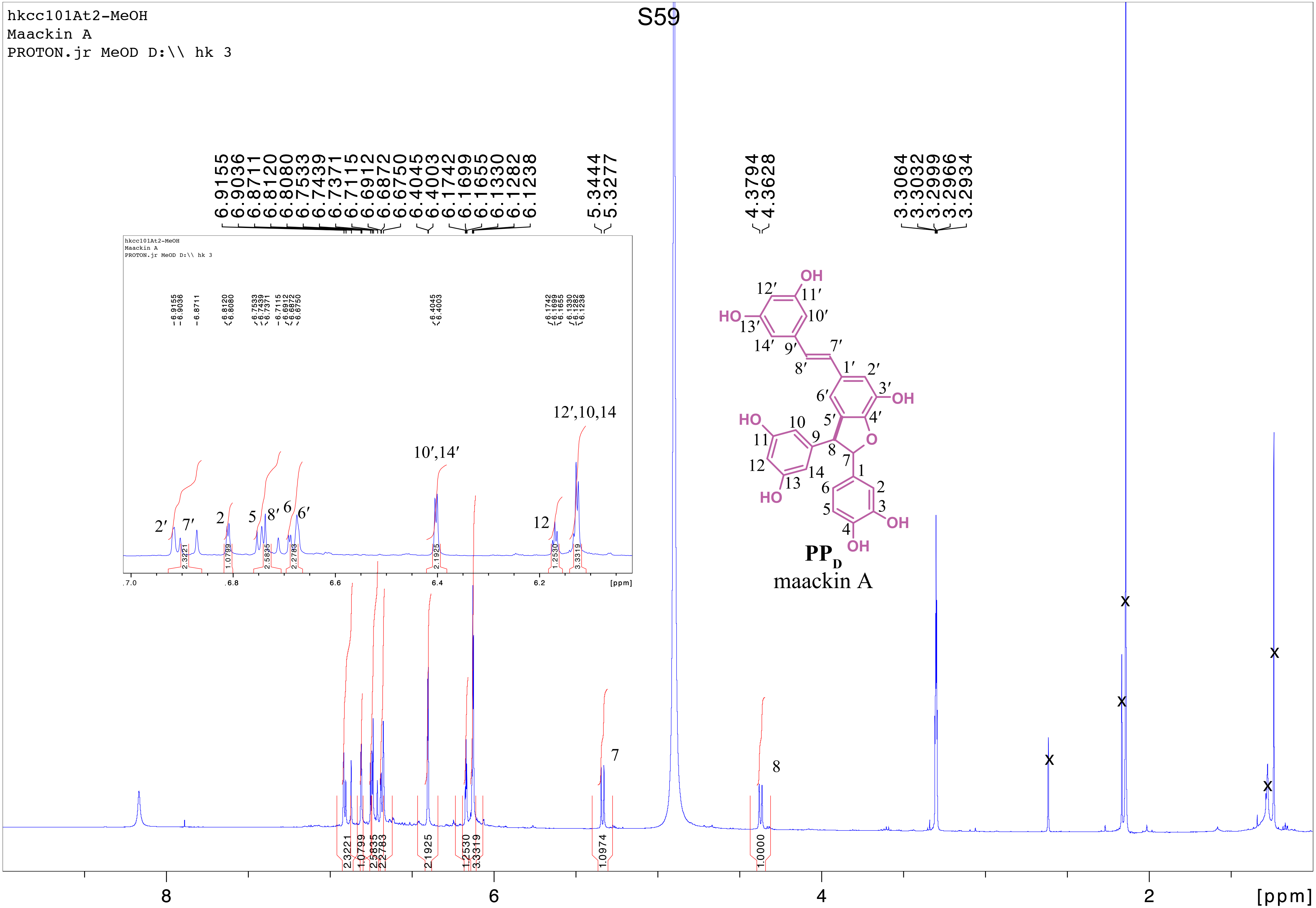
hkcc101At2-MeOH
Maackin A
PROTON.jr MeOD D:\\ hk 3
6.9155
6.9036
6.8711
6.8120
6.8080
6.7533
6.7439
6.7371
6.7115
6.6912
6.6872
6.6750
6.4045
6.4003
6.1742
6.1699
6.1655
6.1330
6.1282
6.1238
5.3444
5.3277
4.3794
4.3628
3.3064
3.3032
3.2999
3.2966
3.2934
2′
7′
2
5
8′
6
6′
10′,14′
12
12′,10,14
2.3221
1.0799
2.5835
2.2783
2.1925
1.2530
3.3319
1.0974
1.0000
7
8
x
PP$_D$
maackin A
8
6
4
2
[ppm]

hkcc101At2-MeOH
Maackin A
CARBON.jr MeOD D:\\ hk 3

S60

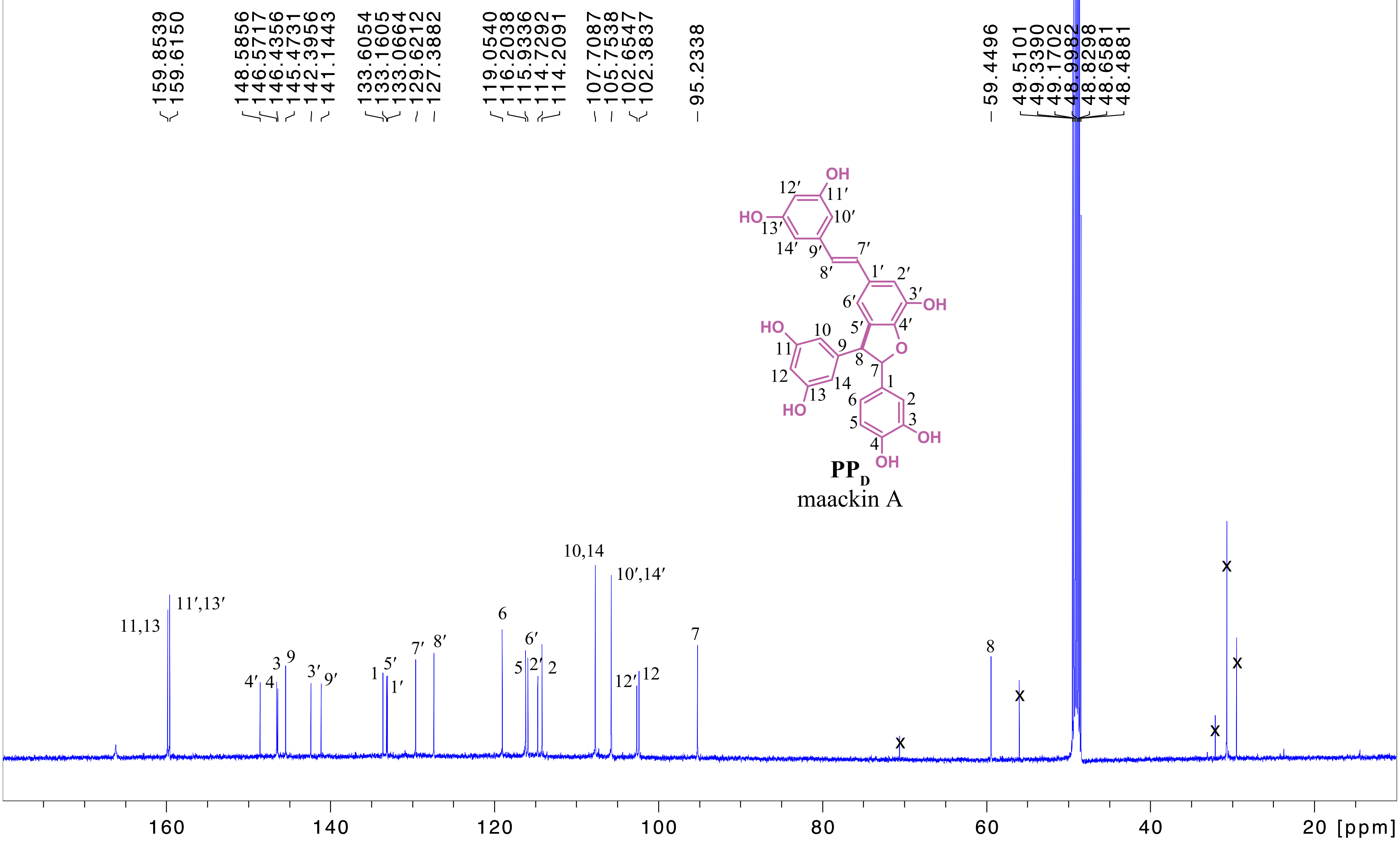

hkcc101At3-MeOH
CARBON.jr MeOD D:\\ hk 4

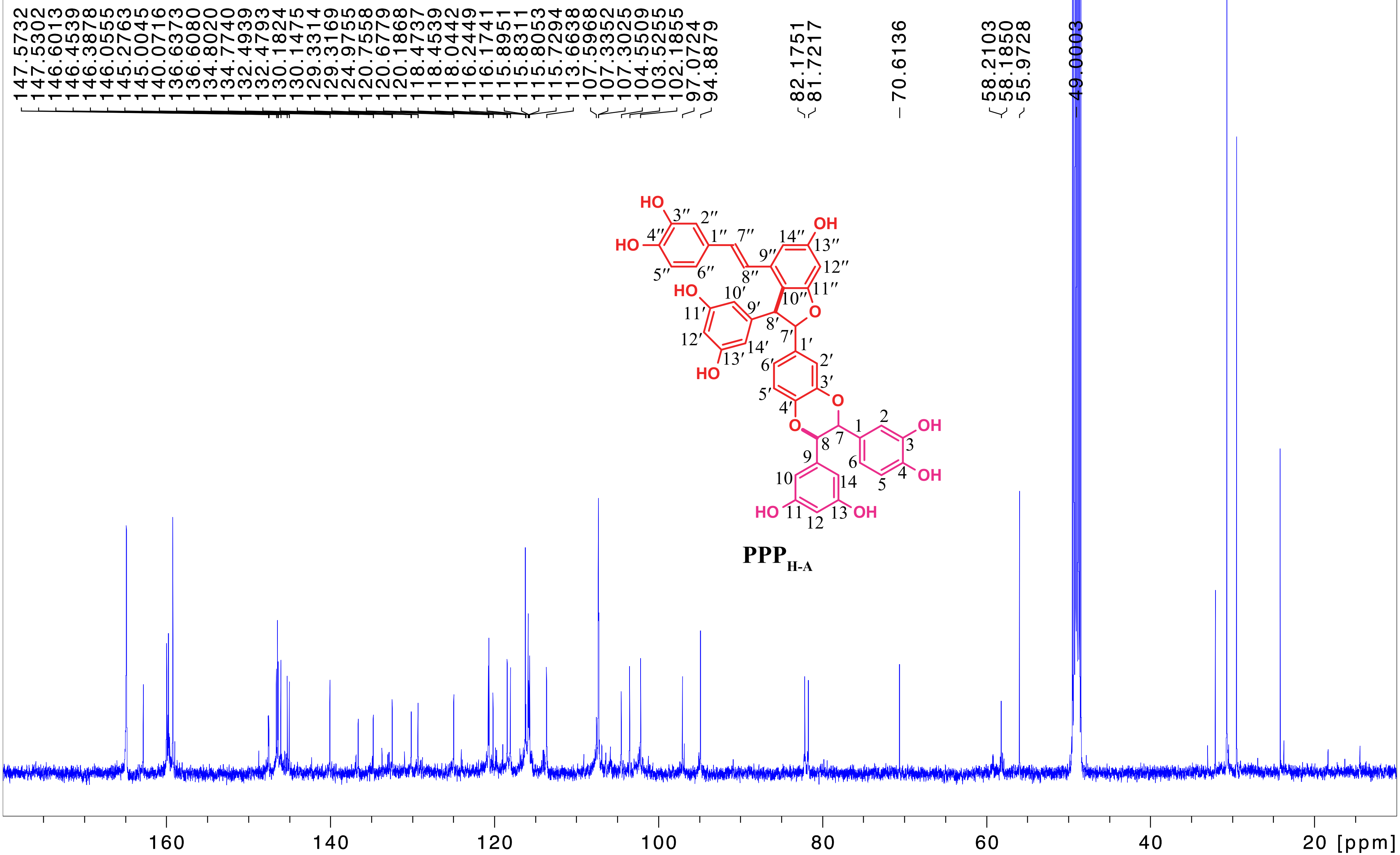

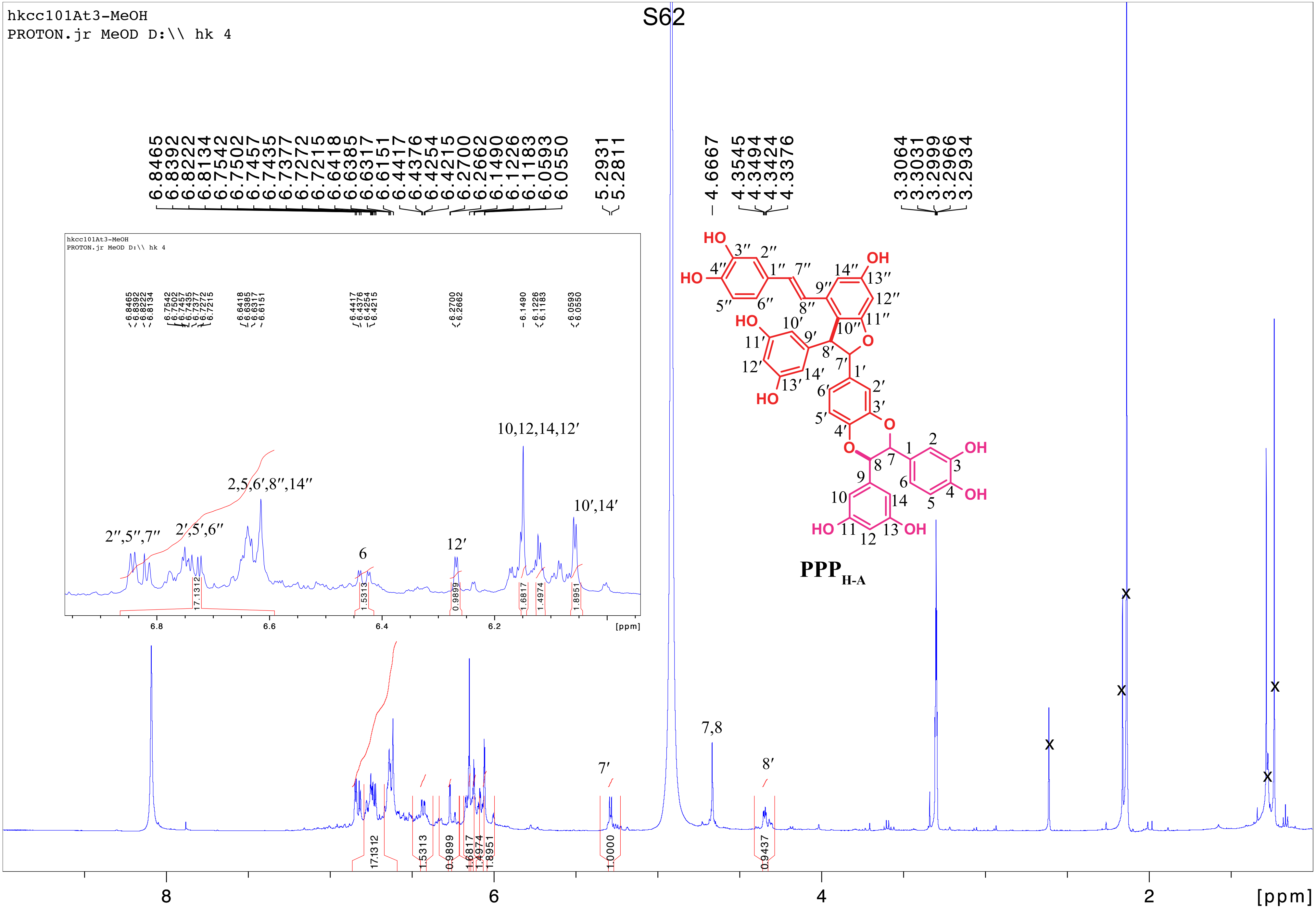
hkcc101At3-MeOH
PROTON.jr MeOD D:\\ hk 4
6.8465 6.8392 6.8222 6.8134 6.7542 6.7502 6.7457 6.7435 6.7377 6.7272 6.7215 6.6418 6.6385 6.6317 6.6151 6.4417 6.4376 6.4254 6.4215 6.2700 6.2662 6.1490 6.1226 6.1183 6.0593 6.0550
5.2931 5.2811
4.6667
4.3545 4.3494 4.3424 4.3376
3.3064 3.3031 3.2999 3.2966 3.2934
2″,5″,7″
2′,5′,6″
2,5,6′,8″,14″
6
12′
10,12,14,12′
10′,14′
17.1312
1.5313
0.9899
1.6817
1.4974
1.8951
1.0000
0.9437
7′
7,8
8′
PPP_H-A
[ppm]

S63

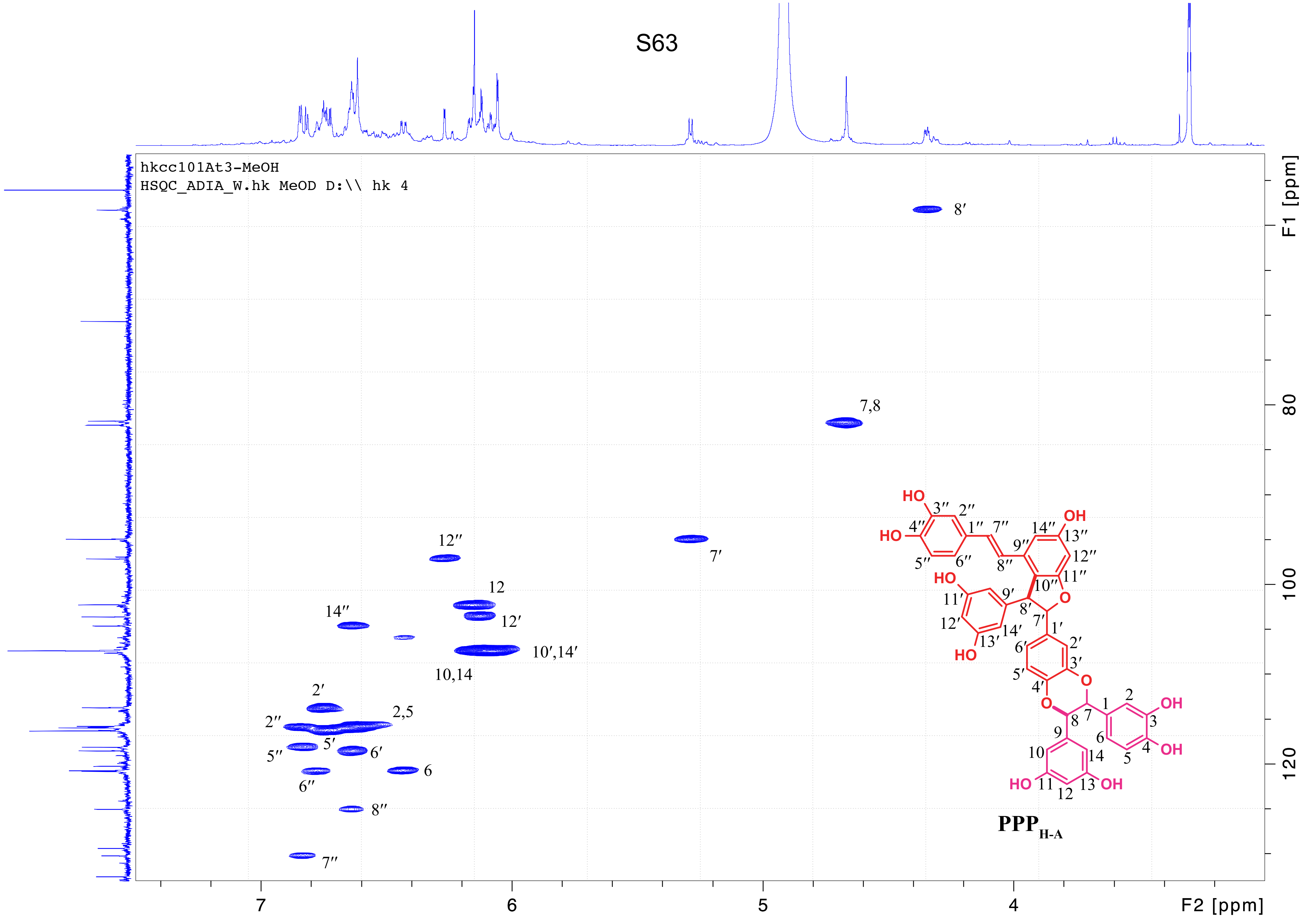
hkcc101At3-MeOH
HSQC_ADIA_W.hk MeOD D:\\ hk 4
F1 [ppm]
F2 [ppm]
8′
7,8
7′
12″
12
12′
14″
10′,14′
10,14
2′
2,5
2″
5′
6′
5″
6
6″
8″
7″
PPP$_{H-A}$

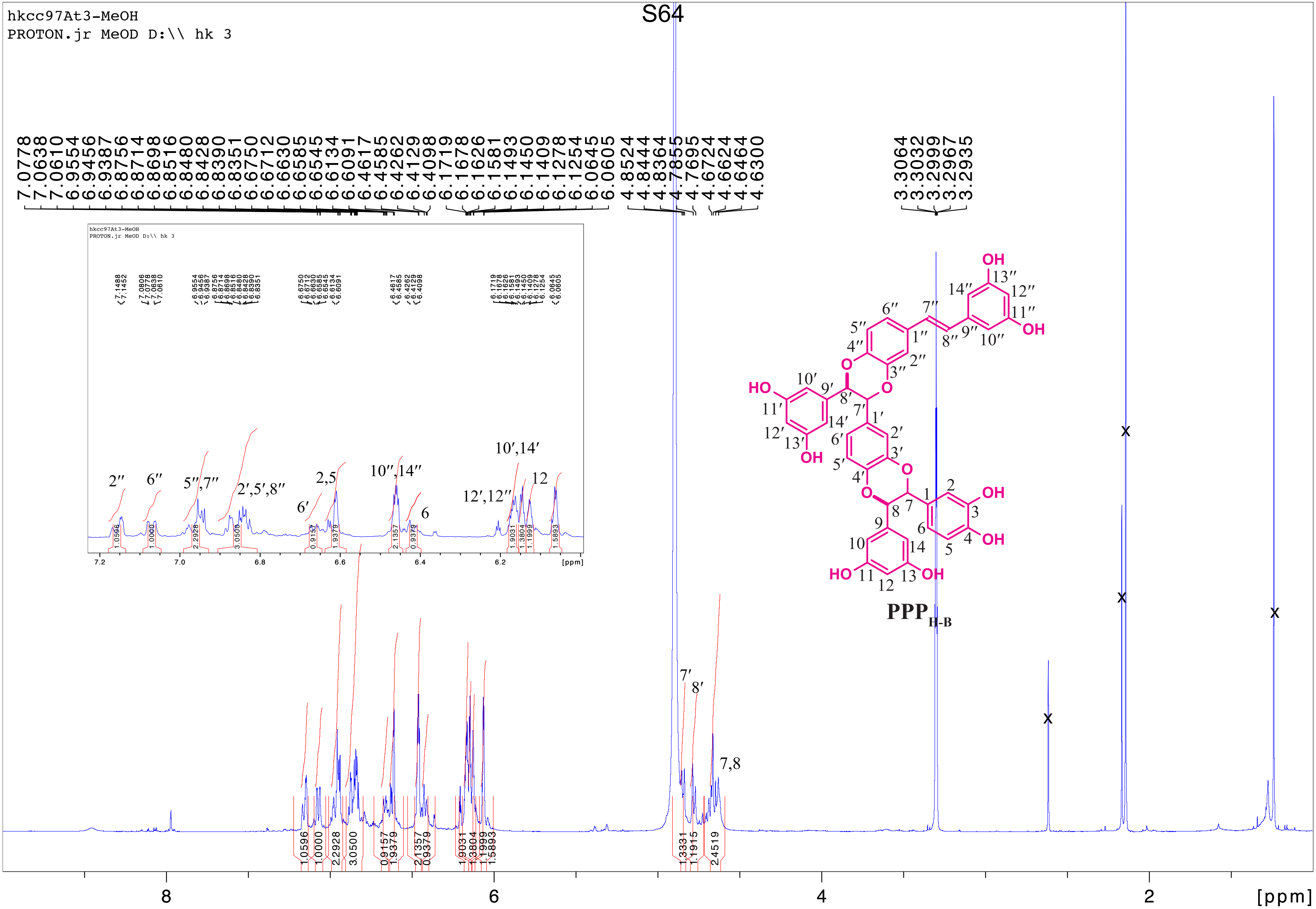

hkcc97At3-MeOH
PROTON.jr MeOD D:\\ hk 3
7.0778 7.0638 7.0610 6.9554 6.9456 6.9387 6.8756 6.8714 6.8698 6.8516 6.8480 6.8428 6.8390 6.8351 6.6750 6.6712 6.6630 6.6585 6.6545 6.6134 6.6091 6.4617 6.4585 6.4262 6.4129 6.4098 6.1719 6.1678 6.1626 6.1581 6.1493 6.1450 6.1409 6.1278 6.1254 6.0645 6.0605 4.8524 4.8444 4.8364 4.7855 4.7695 4.6724 4.6624 4.6464 4.6300
3.3064 3.3032 3.2999 3.2967 3.2935
2″ 6″ 5″,7″ 2′,5′,8″ 6′ 2,5 10″,14″ 6 12′,12″ 10′,14′ 12
7′ 8′ 7,8
PPP$_{H-B}$
[ppm]

hkcc97At3-MeOH
CARBON.jr MeOD D:\\ hk 3

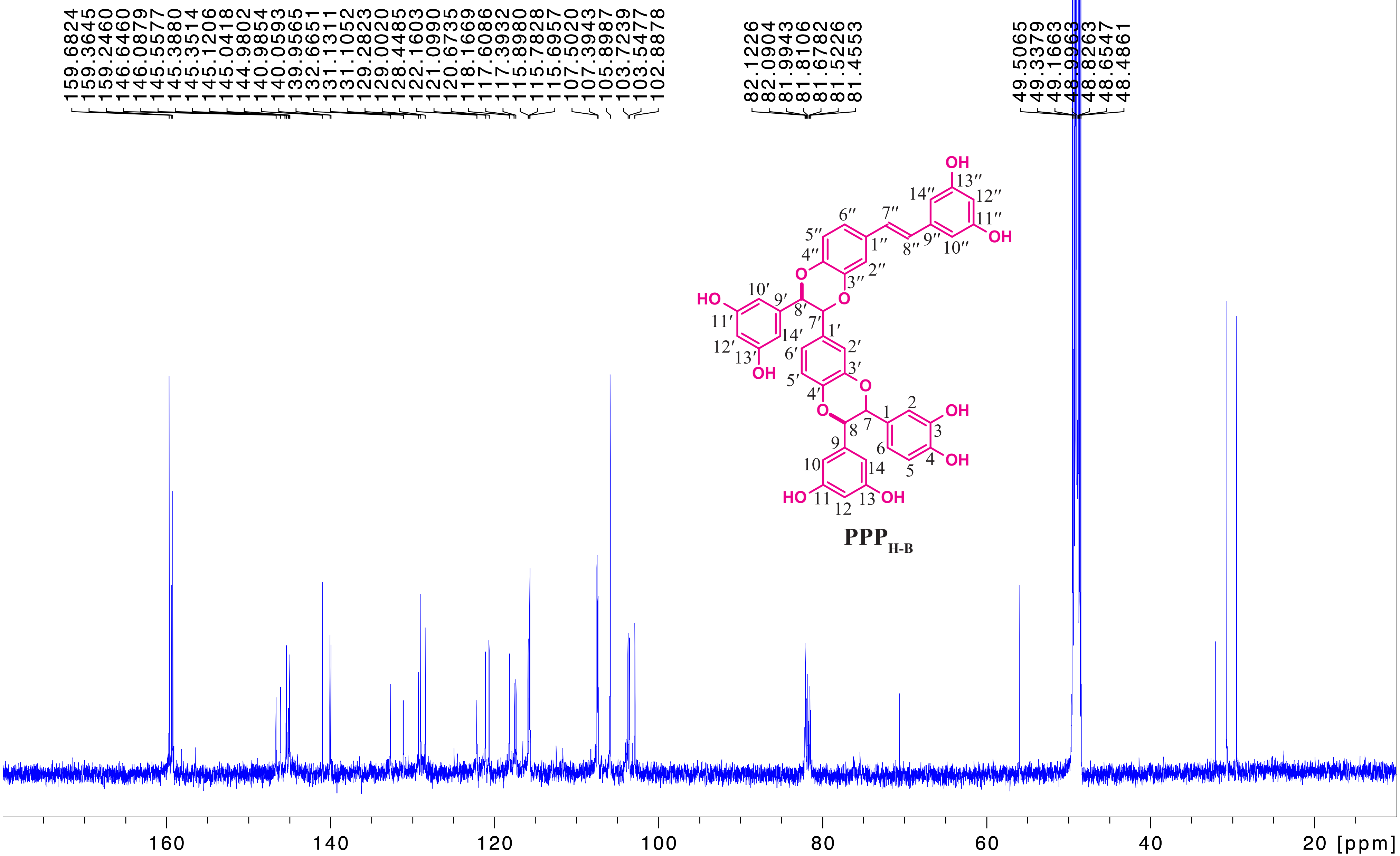

PPP$_{H\text{-}B}$

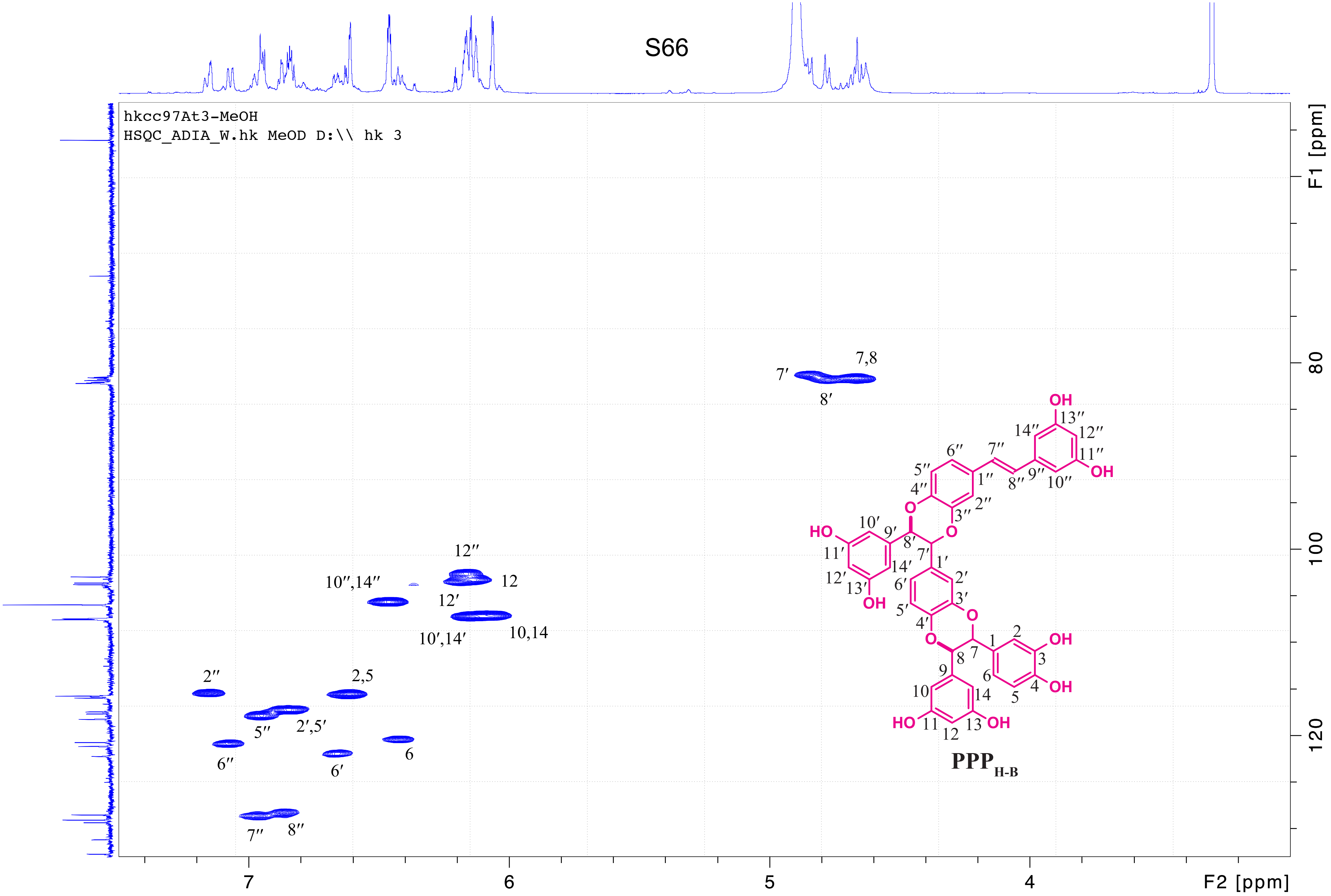
hkcc97At3-MeOH
HSQC_ADIA_W.hk MeOD D:\\ hk 3
F1 [ppm]
F2 [ppm]
7′
7,8
8′
12″
10″,14″
12
12′
10′,14′
10,14
2″
2,5
5″
2′,5′
6″
6′
6
7″
8″
PPP$_{H-B}$

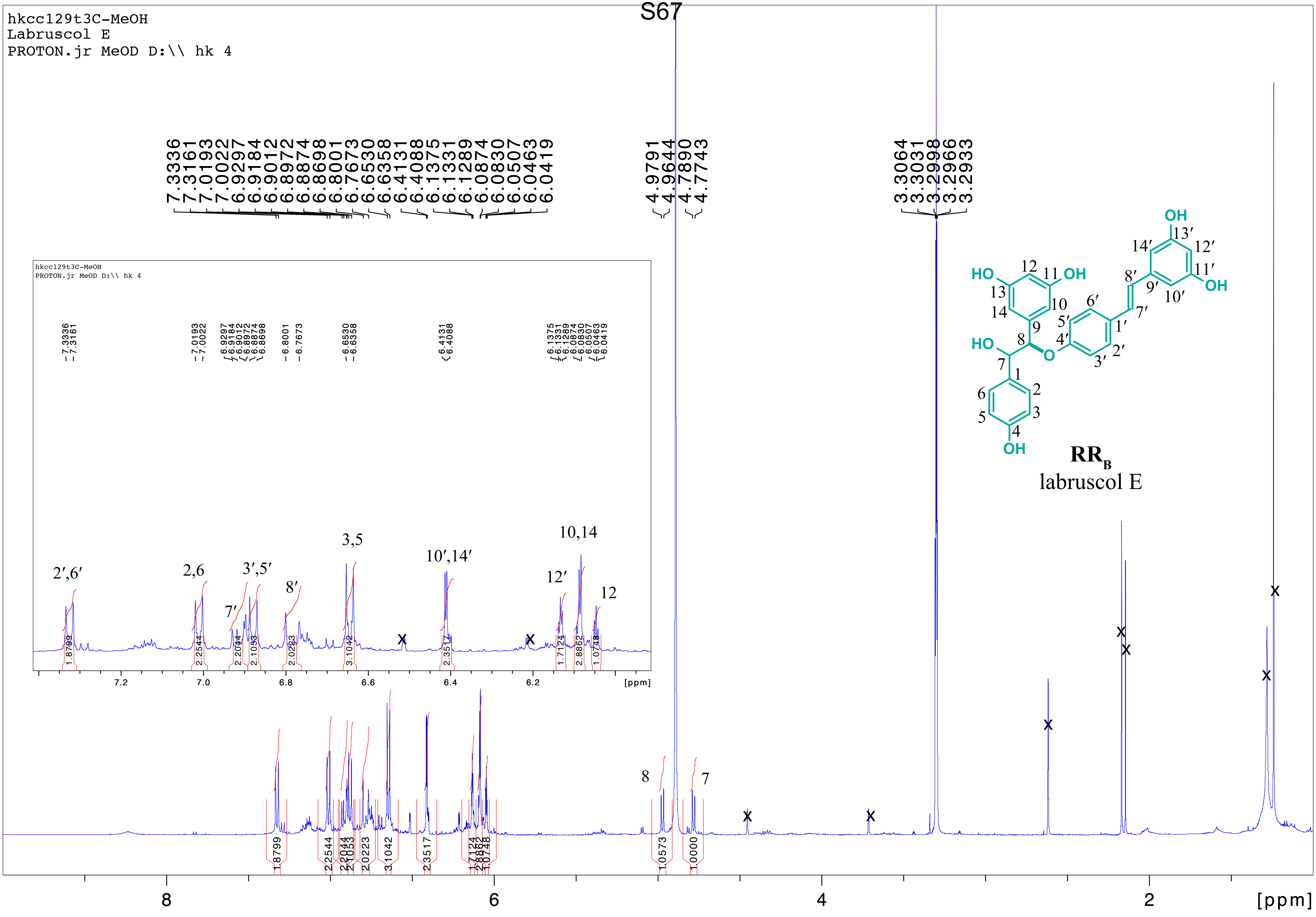

hkcc129t3C-MeOH
Labruscol E
PROTON.jr MeOD D:\\ hk 4
7.3336
7.3161
7.0193
7.0022
6.9297
6.9184
6.9012
6.8972
6.8874
6.8698
6.8001
6.7673
6.6530
6.6358
6.4131
6.4088
6.1375
6.1331
6.1289
6.0874
6.0830
6.0507
6.0463
6.0419
4.9791
4.9644
4.7890
4.7743
3.3064
3.3031
3.2998
3.2966
3.2933
2′,6′
2,6
7′
3′,5′
8′
3,5
10′,14′
12′
10,14
12
1.8799
2.2544
2.2044
2.1053
2.0223
3.1042
2.3517
1.7124
2.8862
1.0748
1.0573
1.0000
8
7
RR_B
labruscol E
[ppm]

S68

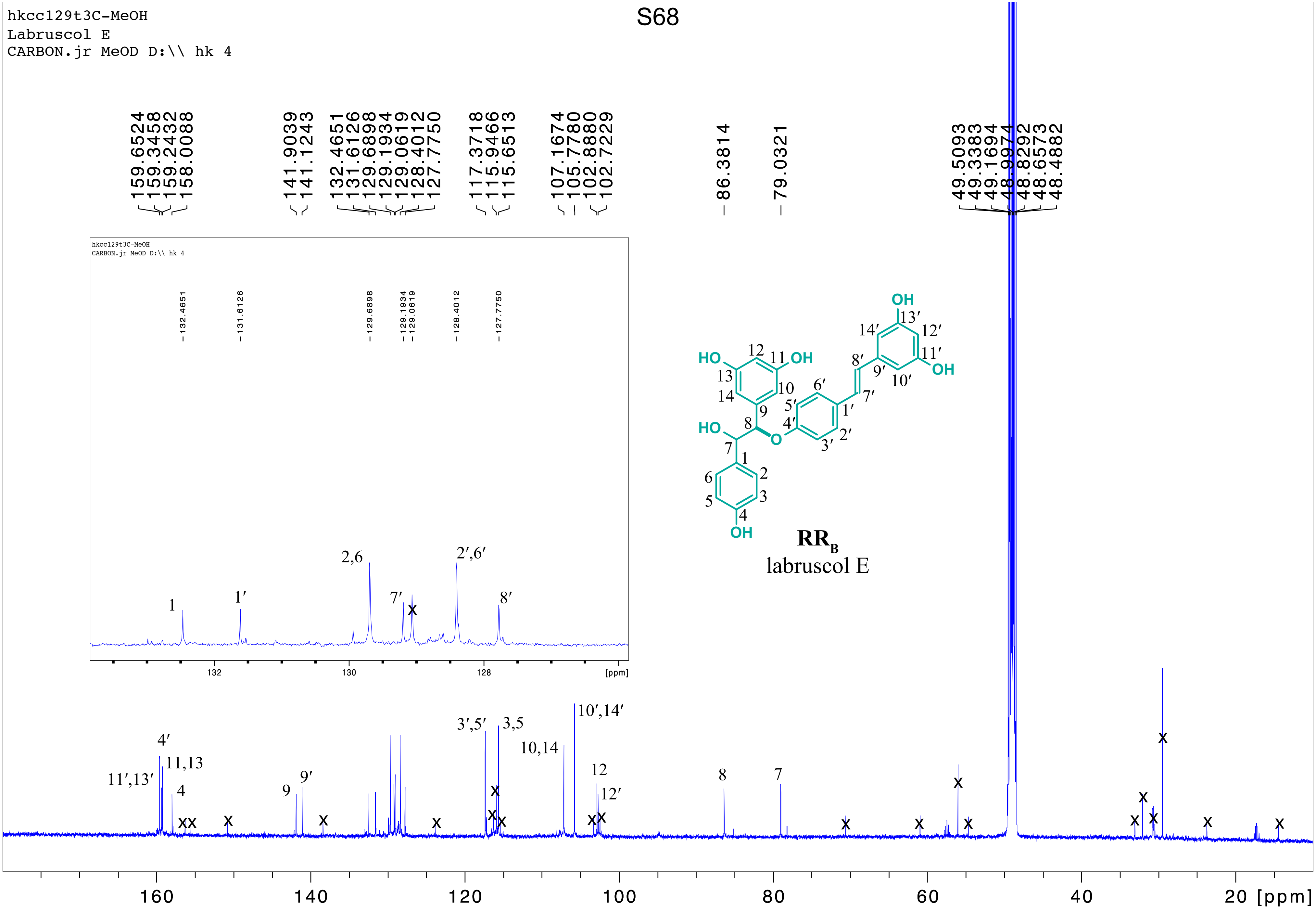

hkcc129t3C-MeOH
Labruscol E
CARBON.jr MeOD D:\\ hk 4
159.6524
159.3458
159.2432
158.0088
141.9039
141.1243
132.4651
131.6126
129.6898
129.1934
129.0619
128.4012
127.7750
117.3718
115.9466
115.6513
107.1674
105.7780
102.8880
102.7229
86.3814
79.0321
49.5093
49.3383
49.1694
48.9974
48.8292
48.6573
48.4882
RR_B
labruscol E
HO
OH
O
1
1′
2,6
7′
2′,6′
8′
4′
11,13
11′,13′
4
9
9′
3′,5′
3,5
10,14
10′,14′
12
12′
8
7
[ppm]

S69

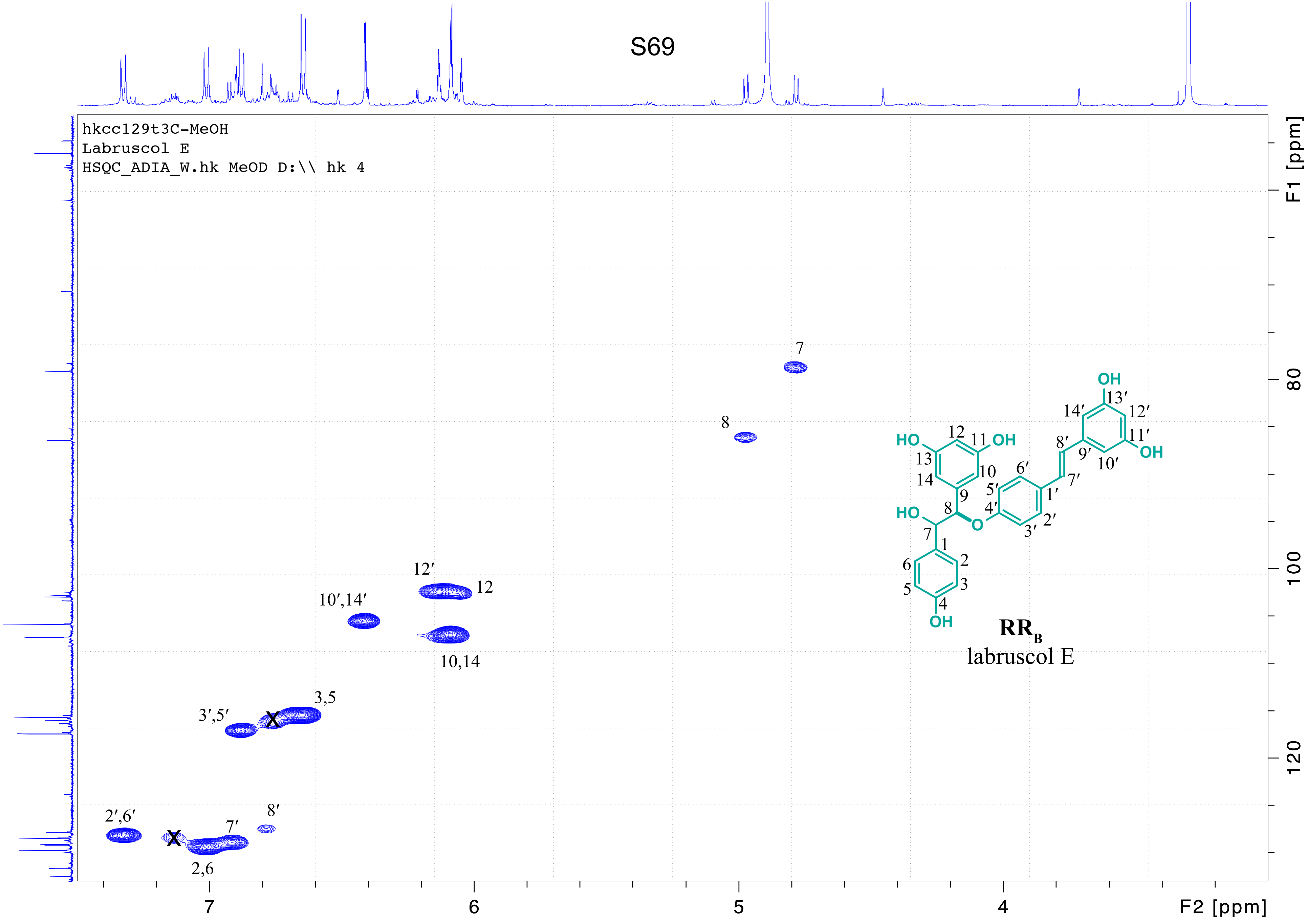

hkcc129t3C-MeOH
Labruscol E
HSQC_ADIA_W.hk MeOD D:\\ hk 4
7
8
12′
12
10′,14′
10,14
3,5
3′,5′
X
2′,6′
X
7′
8′
2,6
RR_B
labruscol E
F1 [ppm]
F2 [ppm]
80
100
120
7
6
5
4

hkcc127At1-MeOH
ε-Viniferin
PROTON.jr MeOD D:\\ hk 2

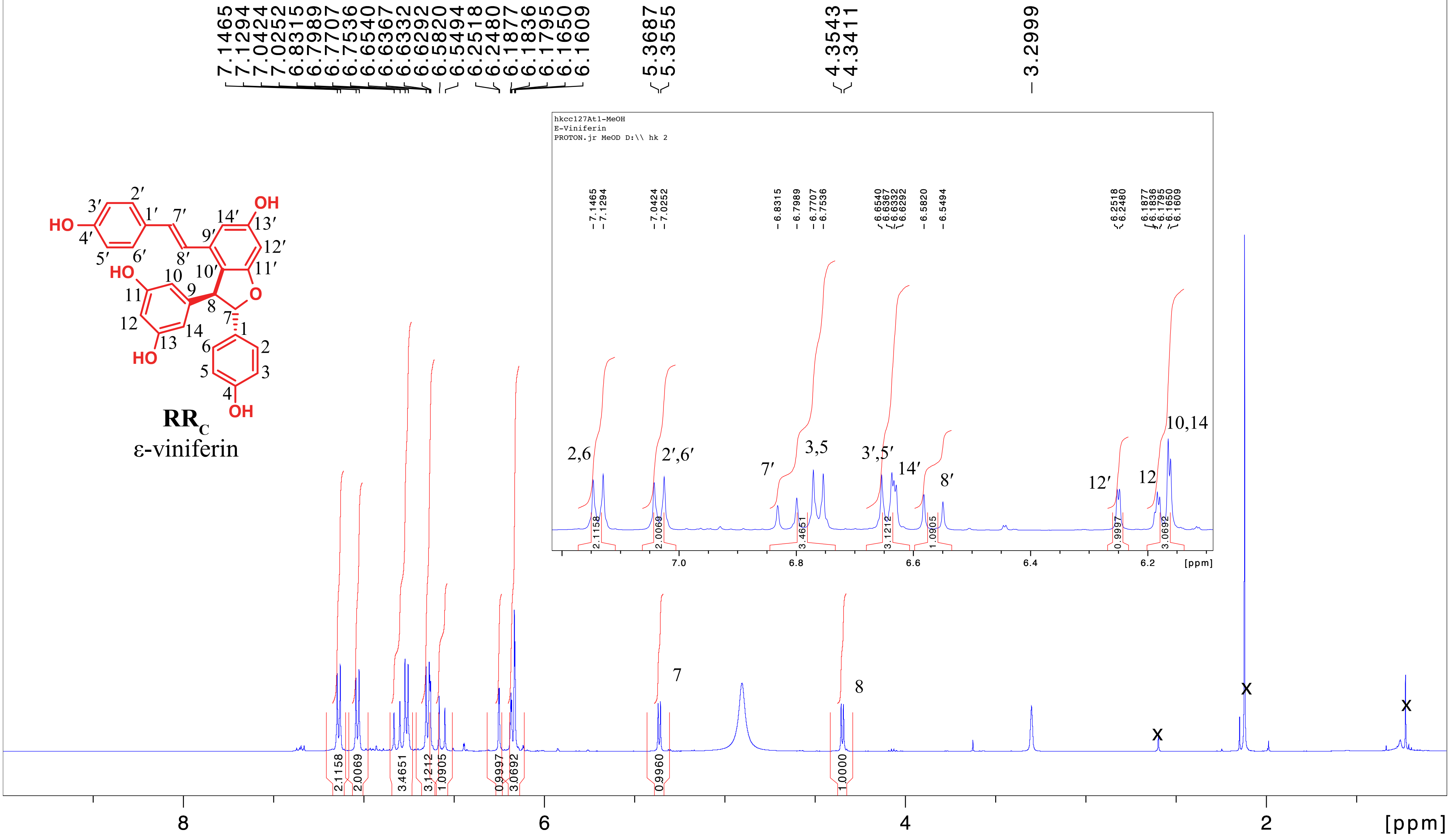

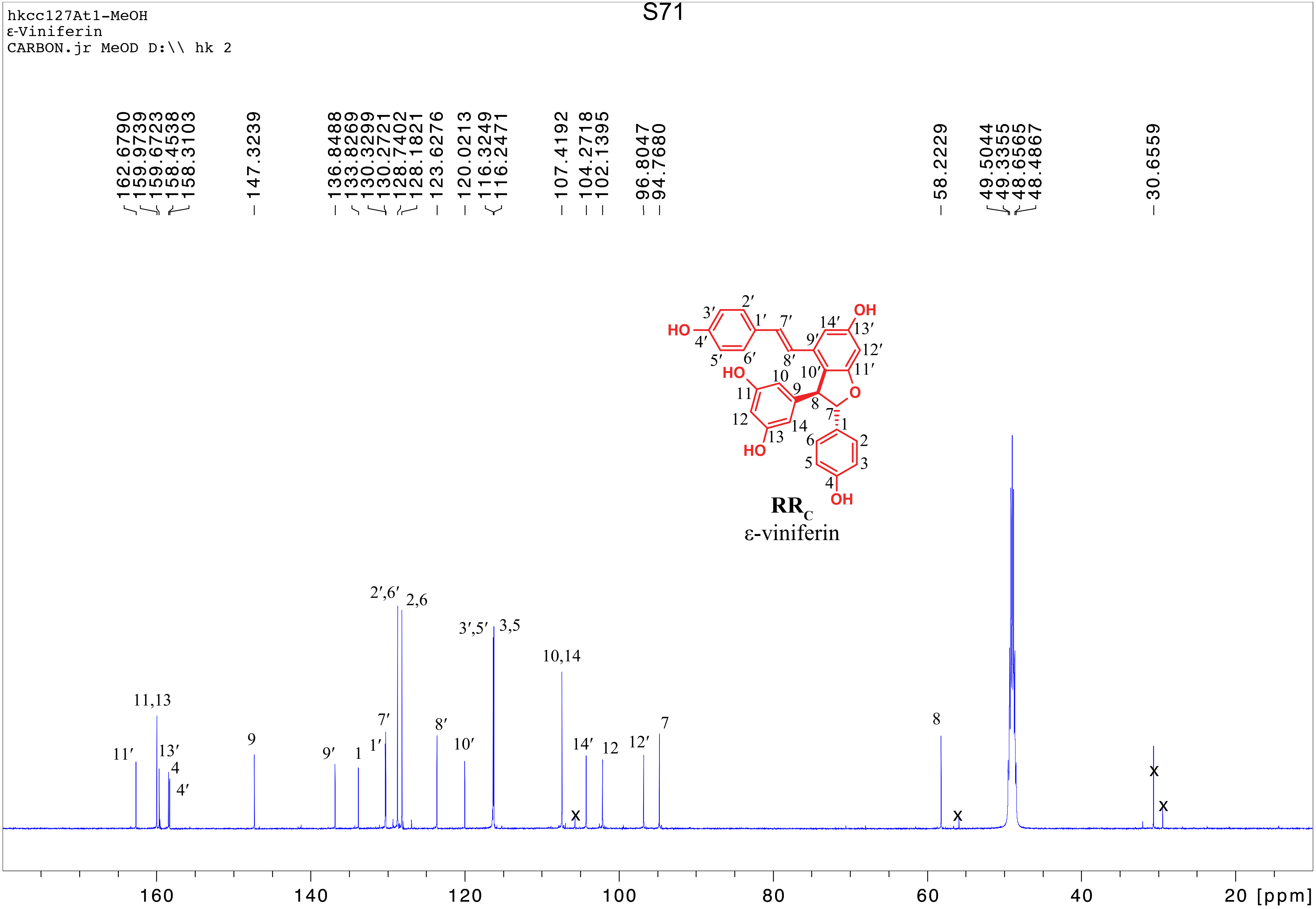
hkcc127At1-MeOH
ε-Viniferin
CARBON.jr MeOD D:\\ hk 2
162.6790
159.9739
159.6723
158.4538
158.3103
147.3239
136.8488
133.8269
130.3299
130.2721
128.7402
128.1821
123.6276
120.0213
116.3249
116.2471
107.4192
104.2718
102.1395
96.8047
94.7680
58.2229
49.5044
49.3355
48.6565
48.4867
30.6559
RR_C
ε-viniferin
11′
11,13
13′
4
4′
9
9′
1
1′
7′
2′,6′
2,6
8′
10′
3′,5′
3,5
10,14
14′
12
12′
7
8
x
[ppm]

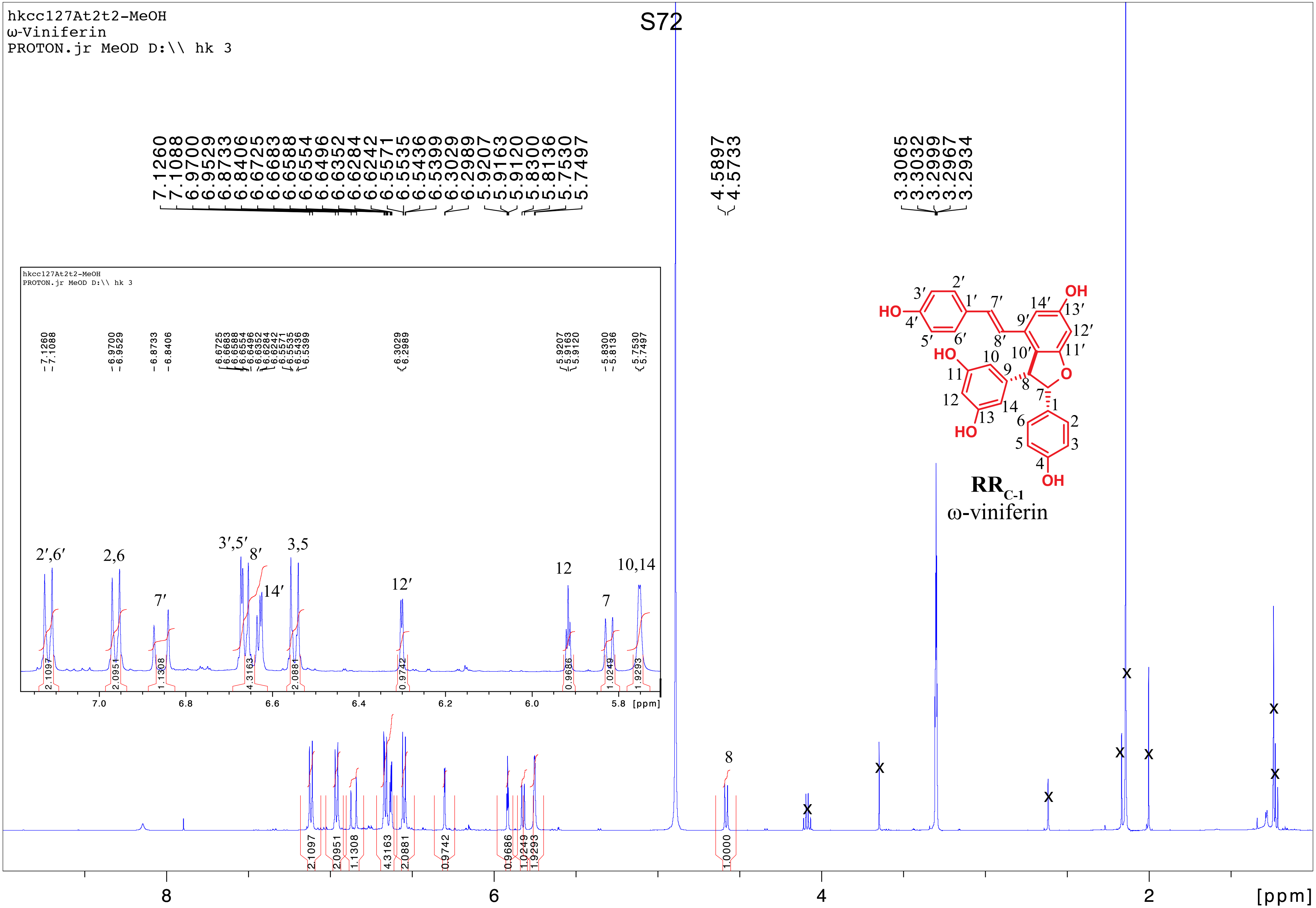
hkcc127At2t2-MeOH
ω-Viniferin
PROTON.jr MeOD D:\\ hk 3
7.1260 7.1088 6.9700 6.9529 6.8733 6.8406 6.6725 6.6683 6.6588 6.6554 6.6496 6.6352 6.6284 6.6242 6.5571 6.5535 6.5436 6.5399 6.3029 6.2989 5.9207 5.9163 5.9120 5.8300 5.8136 5.7530 5.7497
4.5897 4.5733
3.3065 3.3032 3.2999 3.2967 3.2934
2′,6′ 2,6 7′ 3′,5′ 8′ 14′ 3,5 12′ 12 7 10,14
2.1097 2.0951 1.1308 4.3163 2.0881 0.9742 0.9686 1.0249 1.9293 1.0000
8
RR$_{C-1}$
ω-viniferin
[ppm]

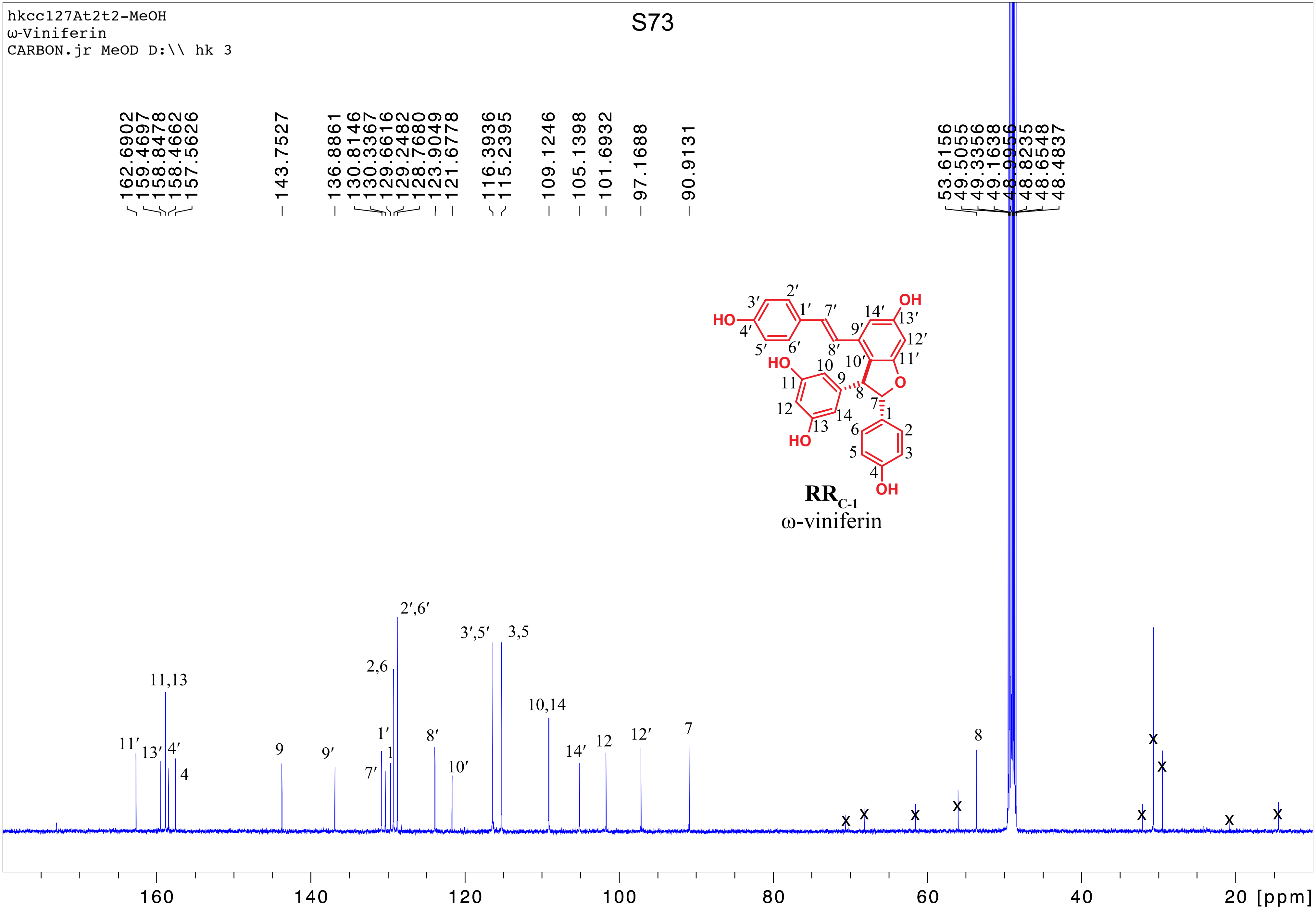
hkcc127At2t2-MeOH
ω-Viniferin
CARBON.jr MeOD D:\\ hk 3
162.6902
159.4697
158.8478
158.4662
157.5626
143.7527
136.8861
130.8146
130.3367
129.6616
129.2482
128.7680
123.9049
121.6778
116.3936
115.2395
109.1246
105.1398
101.6932
97.1688
90.9131
53.6156
49.5055
49.3356
49.1638
48.9956
48.8235
48.6548
48.4837
RR_{C-1}
ω-viniferin
11′
13′
11,13
4′
4
9
9′
7′
2,6
1′
1
2′,6′
8′
10′
3′,5′
3,5
10,14
14′
12
12′
7
8
160
140
120
100
80
60
40
20 [ppm]

hkcc129t2C-MeOH
Delta-Viniferin
PROTON.jr MeOD D:\\ hk 3

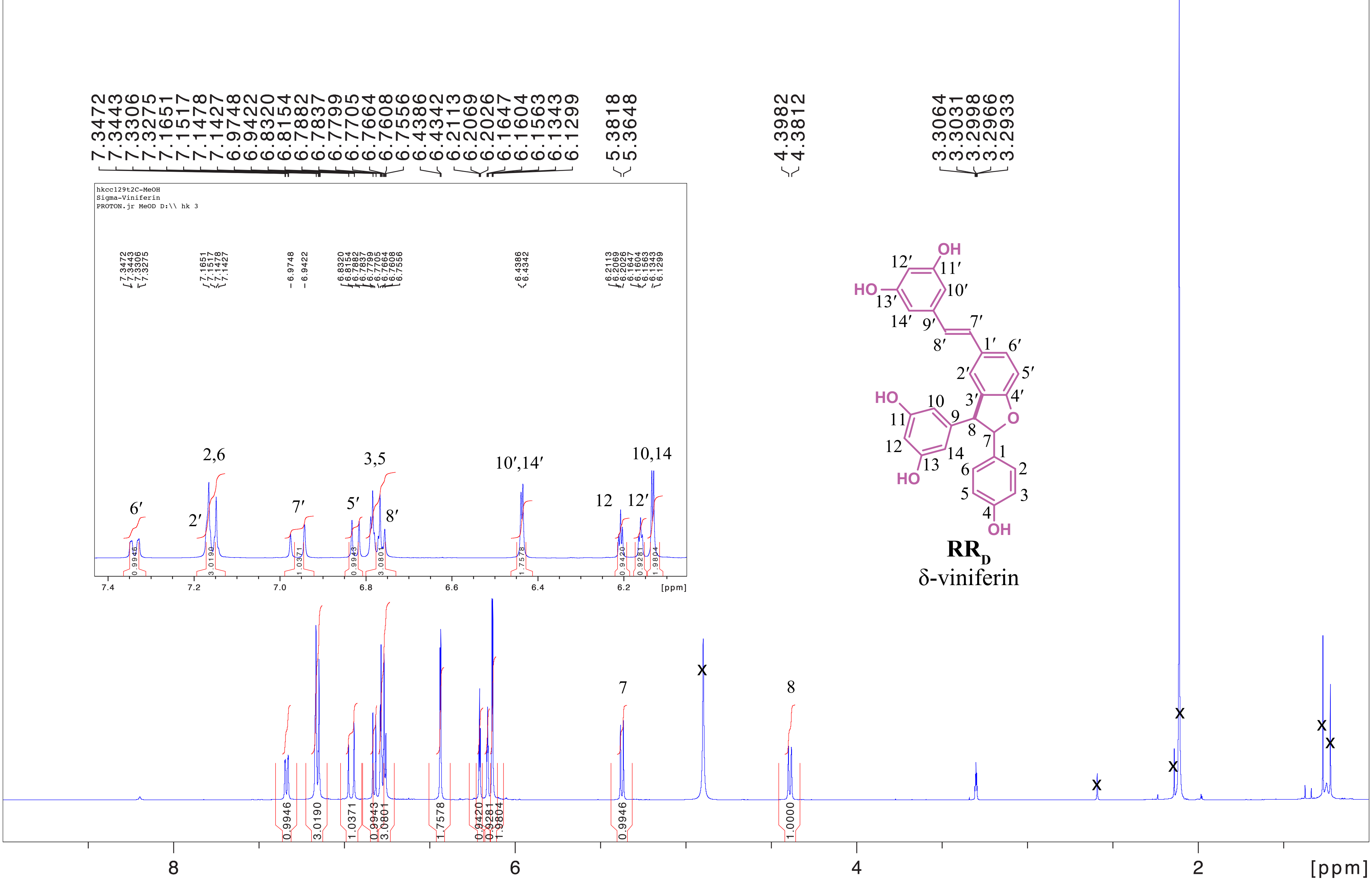

hkcc129t2C-MeOH
Delta-Viniferin
CARBON.jr MeOD D:\\ hk 3

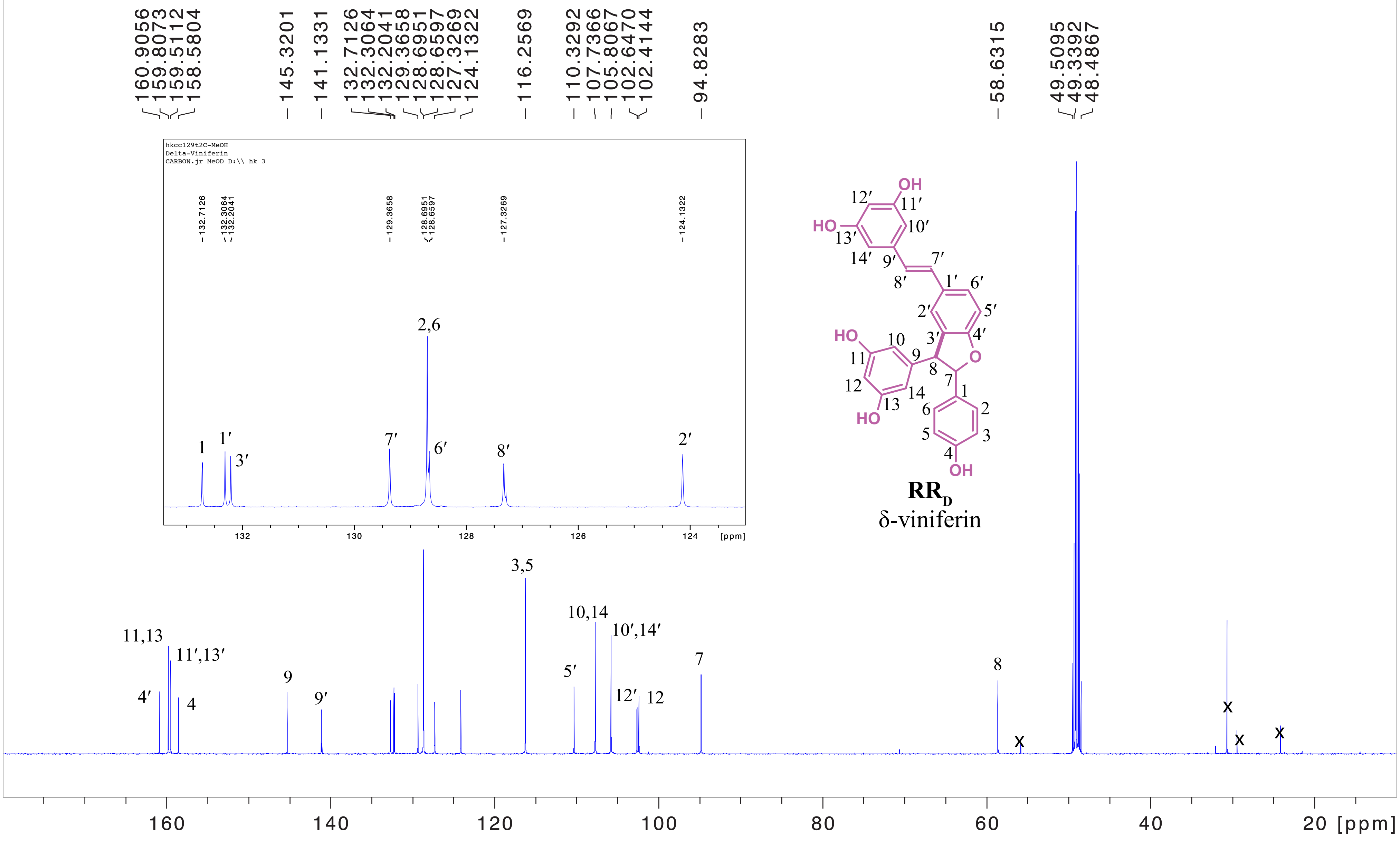

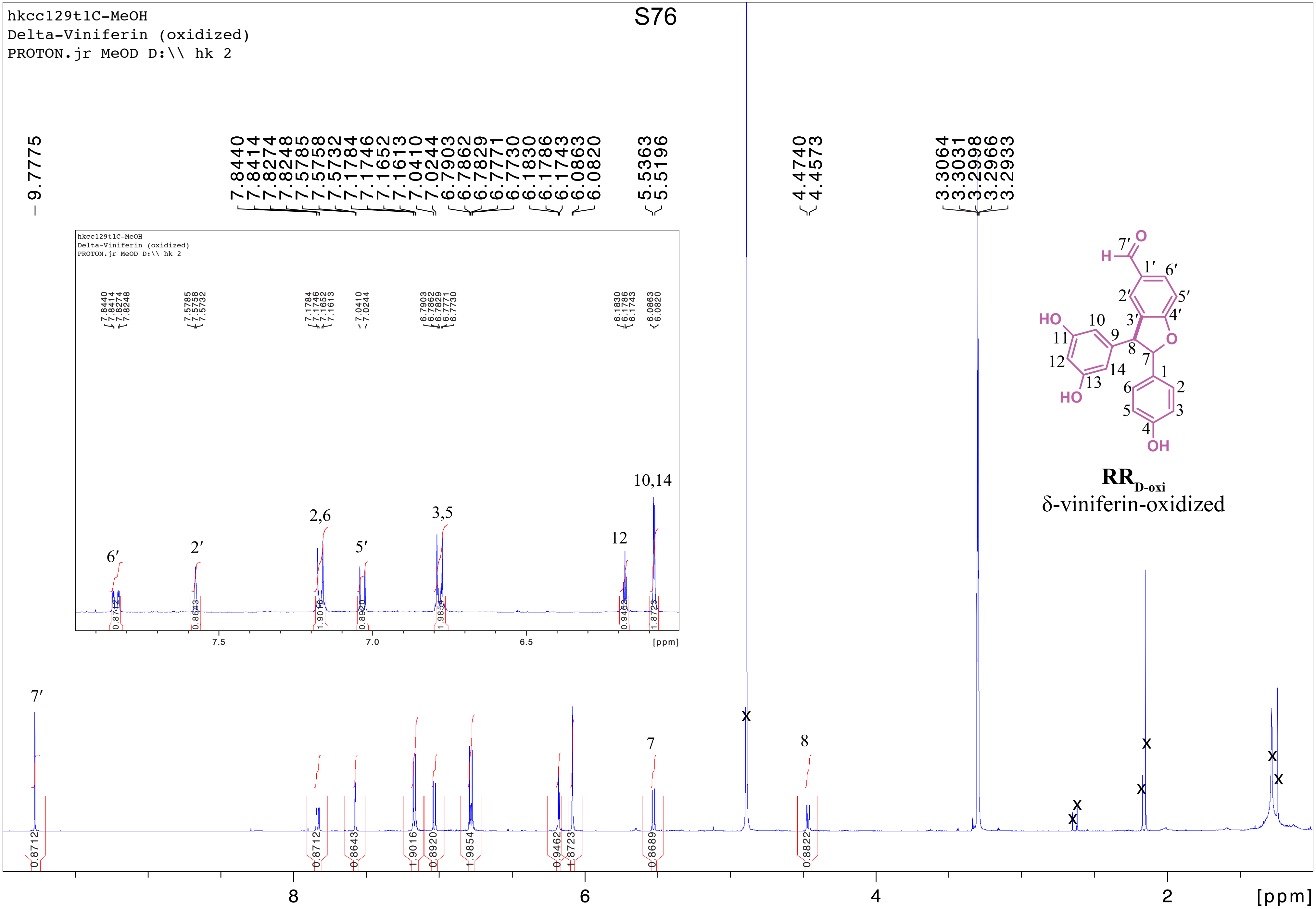
hkcc129t1C-MeOH
Delta-Viniferin (oxidized)
PROTON.jr MeOD D:\\ hk 2
9.7775
7.8440 7.8414 7.8274 7.8248 7.5785 7.5758 7.5732 7.1784 7.1746 7.1652 7.1613 7.0410 7.0244 6.7903 6.7862 6.7829 6.7771 6.7730 6.1830 6.1786 6.1743 6.0863 6.0820
5.5363 5.5196
4.4740 4.4573
3.3064 3.3031 3.2998 3.2966 3.2933
7′
6′
2′
2,6
5′
3,5
12
10,14
7
8
0.8712
0.8712
0.8643
1.9016
0.8920
1.9854
0.9462
1.8723
0.8689
0.8822
0.8742
7.5
7.0
6.5
[ppm]
8
6
4
2
[ppm]
RR$_{D-oxi}$
δ-viniferin-oxidized

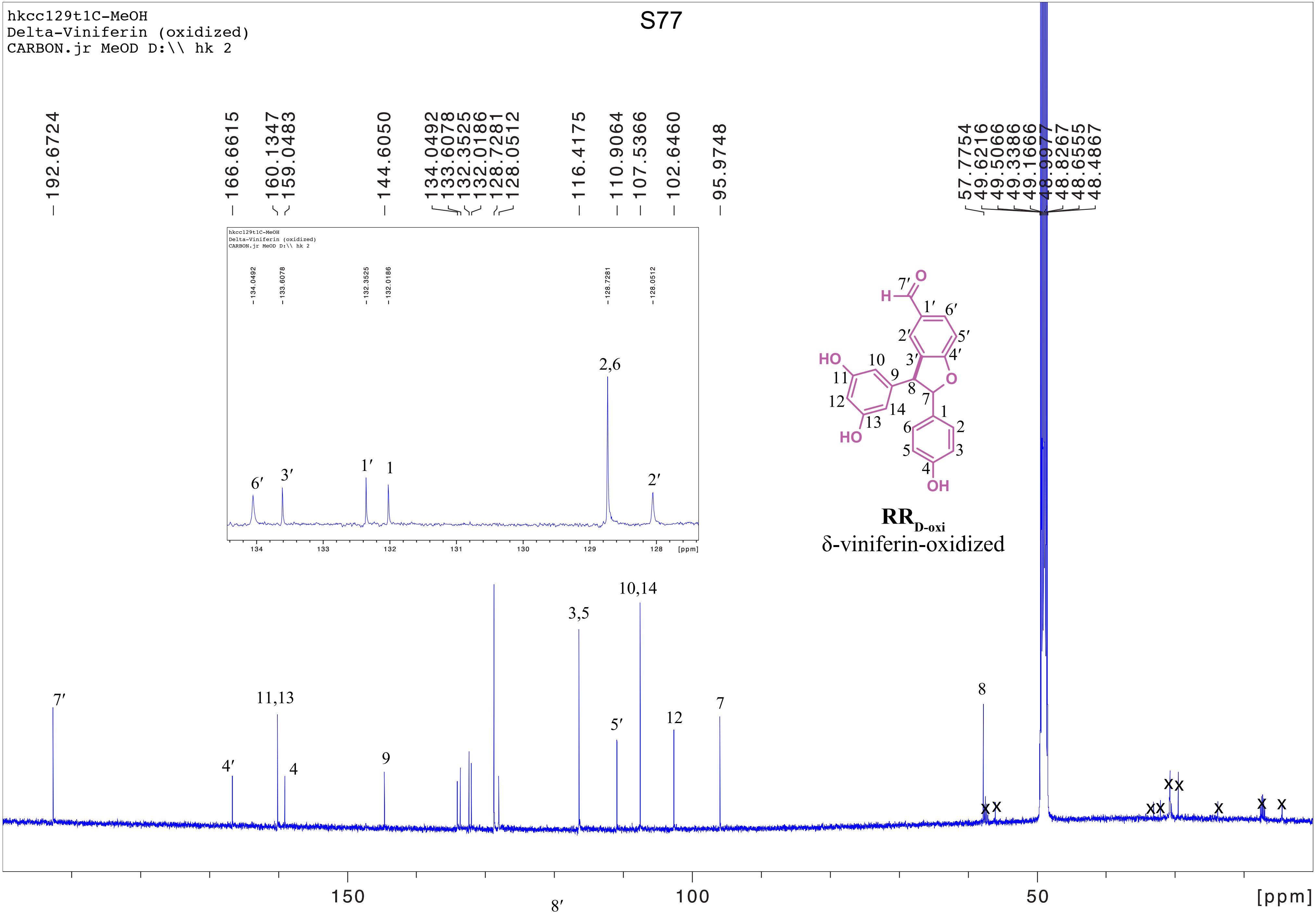
hkcc129t1C-MeOH
Delta-Viniferin (oxidized)
CARBON.jr MeOD D:\\ hk 2
192.6724
166.6615
160.1347
159.0483
144.6050
134.0492
133.6078
132.3525
132.0186
128.7281
128.0512
116.4175
110.9064
107.5366
102.6460
95.9748
57.7754
49.6216
49.5066
49.3386
49.1666
48.9977
48.8267
48.6555
48.4867
RR$_{D-oxi}$
δ-viniferin-oxidized
7′
4′
11,13
4
9
3,5
10,14
5′
12
7
8
2,6
6′
3′
1′
1
2′
8′
150
100
50
[ppm]

hkcc127At4t1-MeOH
Pallidol
PROTON.jr MeOD D:\\ hk 4

S78

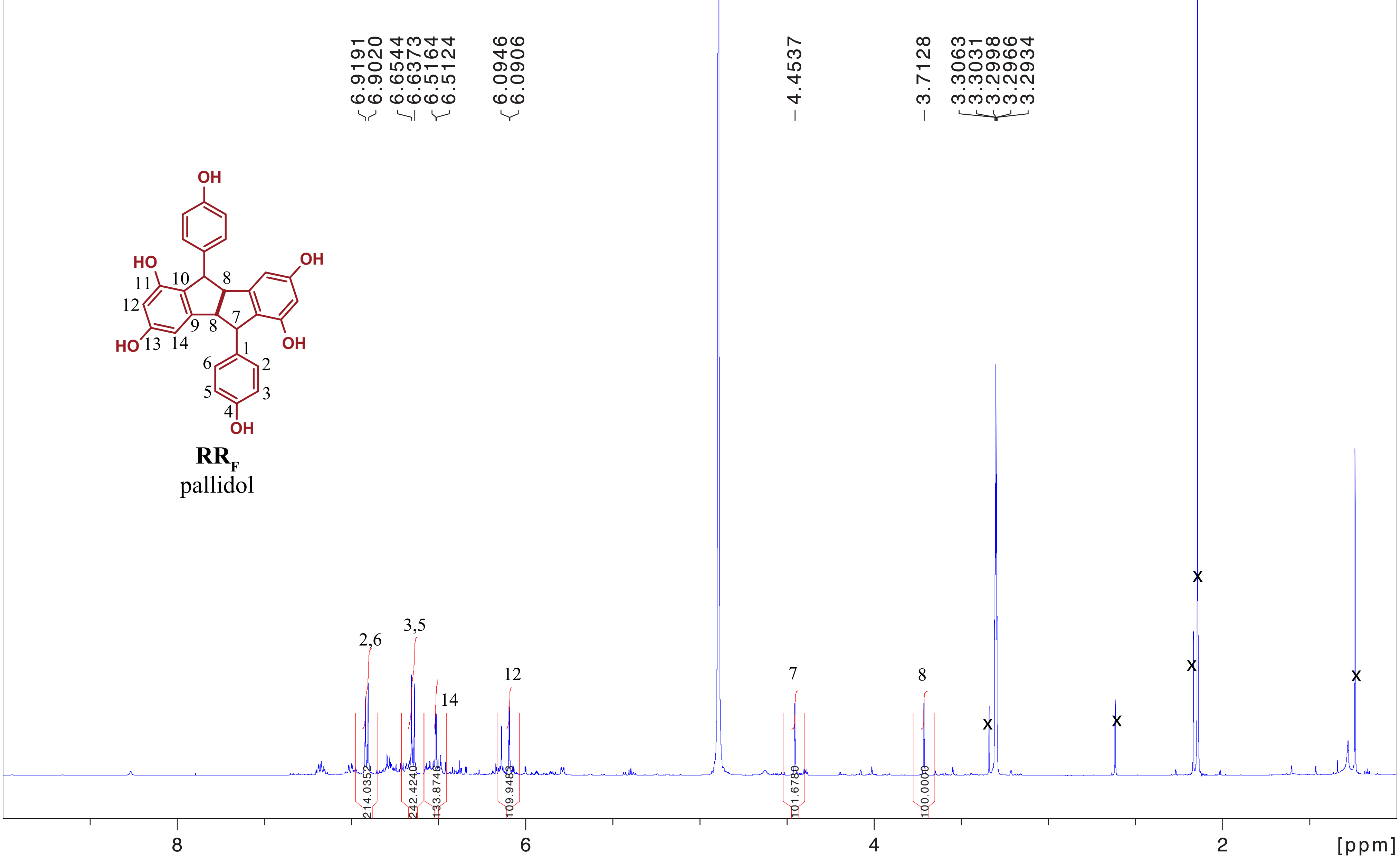

hkcc127At4t1-MeOH
Pallidol
CARBON.jr MeOD D:\\ hk 4

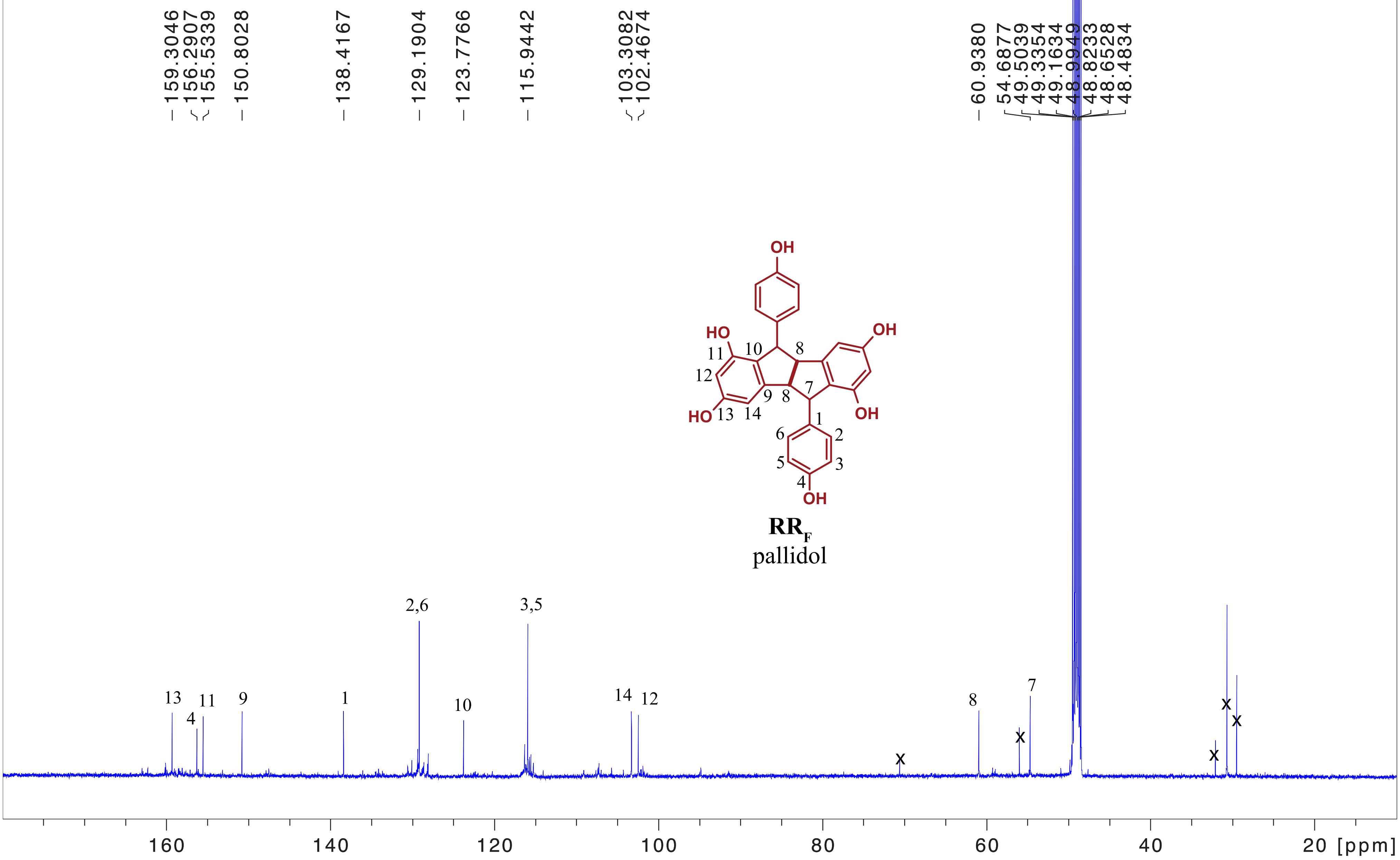

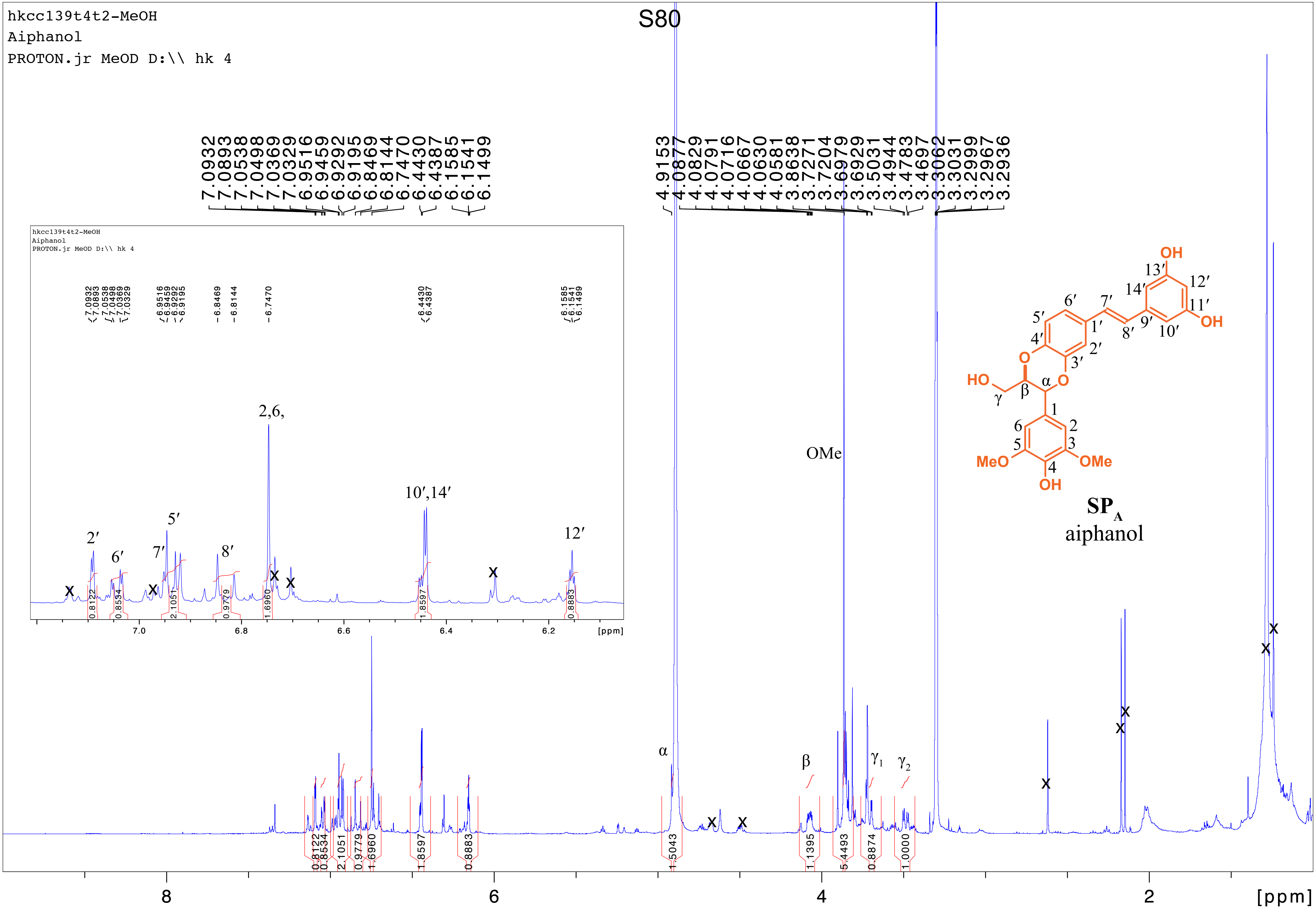
hkcc139t4t2-MeOH
Aiphanol
PROTON.jr MeOD D:\\ hk 4
7.0932
7.0893
7.0538
7.0498
7.0369
7.0329
6.9516
6.9459
6.9292
6.9195
6.8469
6.8144
6.7470
6.4430
6.4387
6.1585
6.1541
6.1499
4.9153
4.0877
4.0829
4.0791
4.0716
4.0667
4.0630
4.0581
3.8638
3.7271
3.7204
3.6979
3.6929
3.5031
3.4944
3.4783
3.4697
3.3062
3.3031
3.2999
3.2967
3.2936
2′
6′
7′
5′
8′
2,6,
10′,14′
12′
α
β
OMe
γ1
γ2
HO
OH
MeO
OMe
SPA
aiphanol
[ppm]

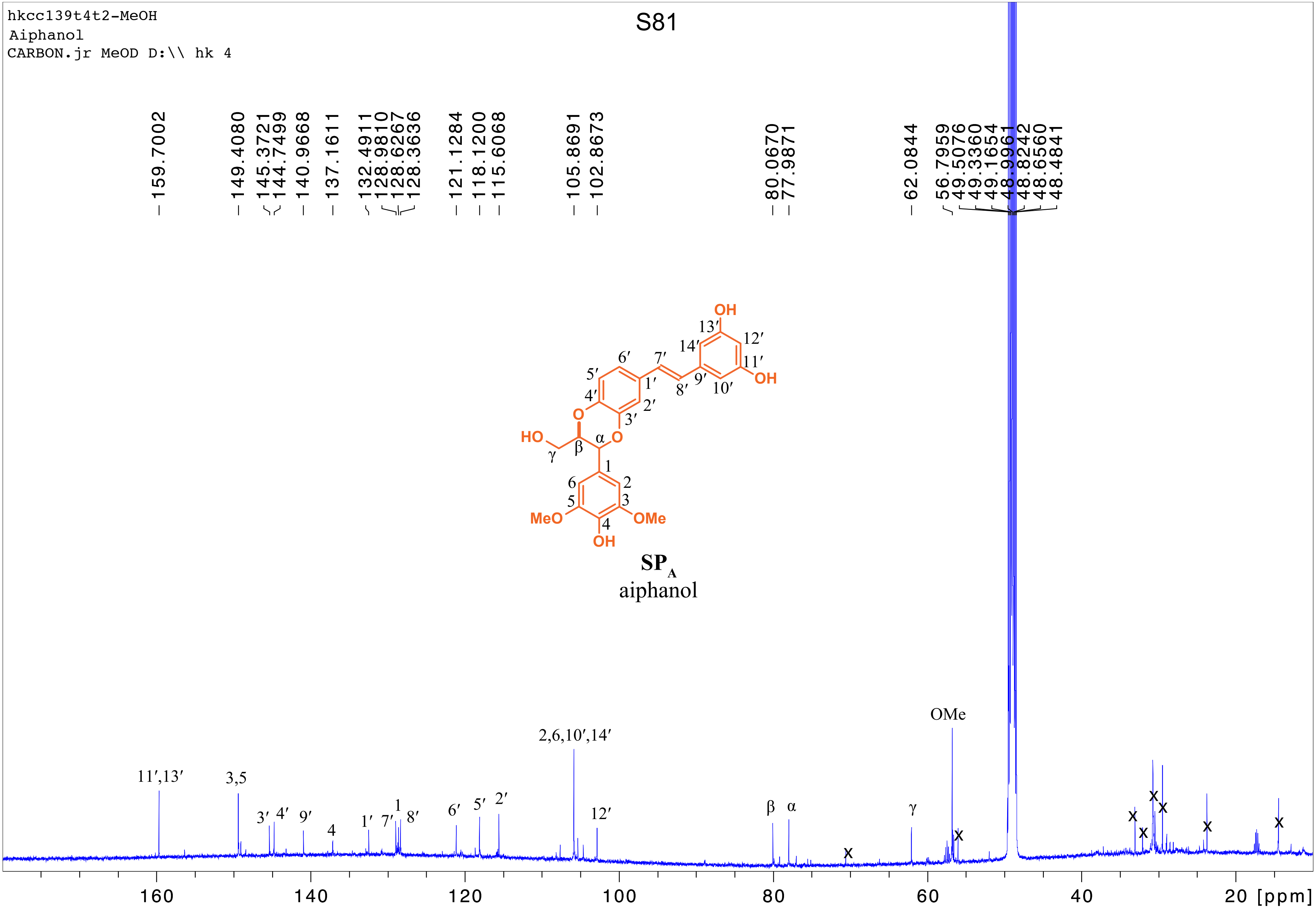

hkcc139t4t2-MeOH
Aiphanol
CARBON.jr MeOD D:\\ hk 4
159.7002
149.4080
145.3721
144.7499
140.9668
137.1611
132.4911
128.9810
128.6267
128.3636
121.1284
118.1200
115.6068
105.8691
102.8673
80.0670
77.9871
62.0844
56.7959
49.5076
49.3360
49.1654
48.9961
48.8242
48.6560
48.4841
SP
A
aiphanol
OH
OH
HO
MeO
OMe
OH
11′,13′
3,5
3′
4′
9′
4
1′
7′
1
8′
6′
5′
2′
2,6,10′,14′
12′
β
α
γ
OMe
160
140
120
100
80
60
40
20 [ppm]

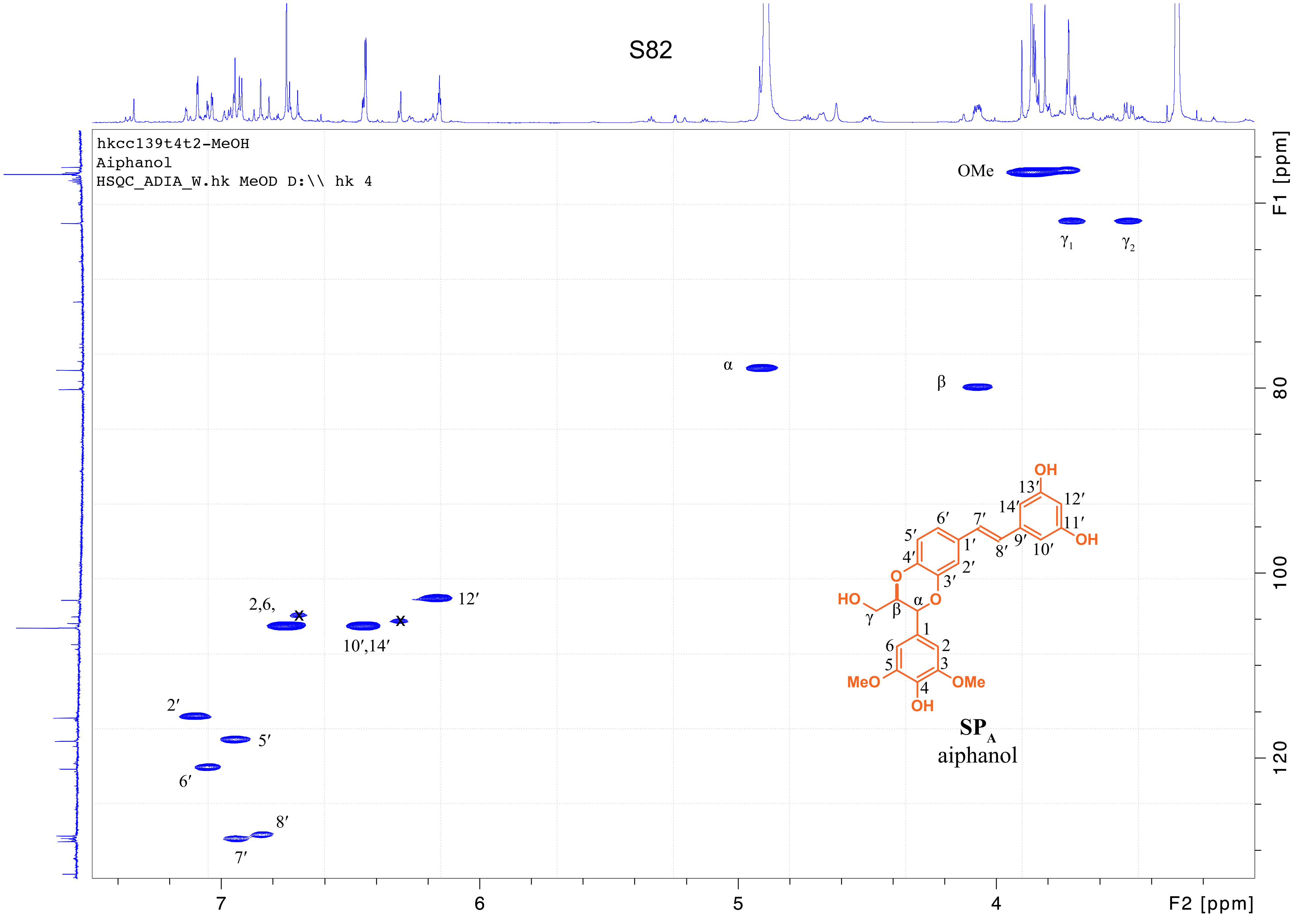

hkcc139t4t2-MeOH
Aiphanol
HSQC_ADIA_W.hk MeOD D:\\ hk 4
OMe
γ1
γ2
α
β
2,6,
12′
10′,14′
2′
5′
6′
8′
7′
SPA
aiphanol
F1 [ppm]
F2 [ppm]

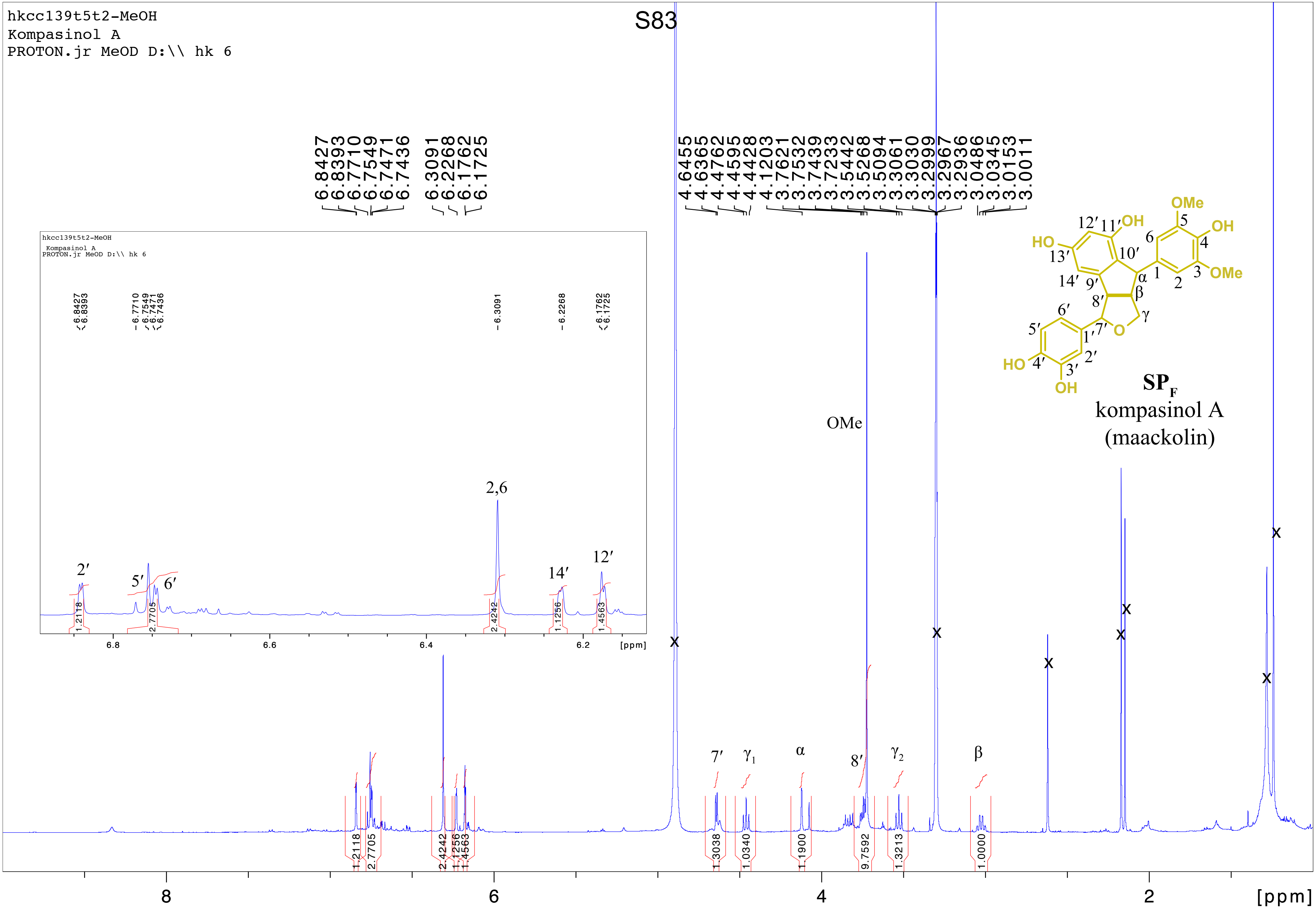

hkcc139t5t2-MeOH
Kompasinol A
PROTON.jr MeOD D:\\ hk 6
6.8427 6.8393 6.7710 6.7549 6.7471 6.7436 6.3091 6.2268 6.1762 6.1725
4.6455 4.6365 4.4762 4.4595 4.4428 4.1203 3.7621 3.7532 3.7439 3.7233 3.5442 3.5268 3.5094 3.3061 3.3030 3.2999 3.2967 3.2936 3.0486 3.0345 3.0153 3.0011
2′
5′
6′
2,6
14′
12′
OMe
7′
γ_1
α
8′
γ_2
β
1.2118 2.7705 2.4242 1.1256 1.4563
1.3038 1.0340 1.1900 9.7592 1.3213 1.0000
SP_F
kompasinol A
(maackolin)
[ppm]
8
6
4
2

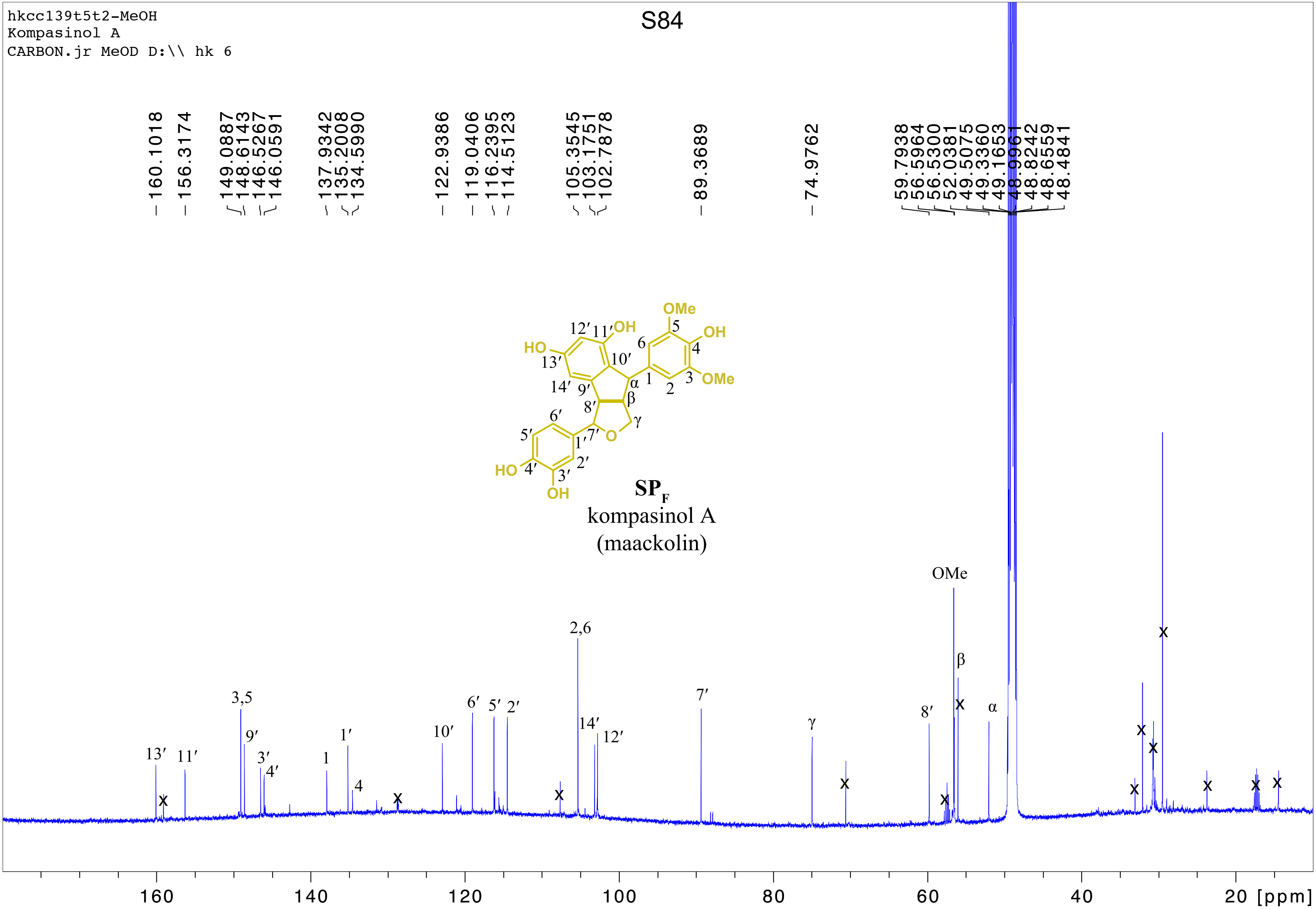
hkcc139t5t2-MeOH
Kompasinol A
CARBON.jr MeOD D:\\ hk 6
160.1018
156.3174
149.0887
148.6143
146.5267
146.0591
137.9342
135.2008
134.5990
122.9386
119.0406
116.2395
114.5123
105.3545
103.1751
102.7878
89.3689
74.9762
59.7938
56.5964
56.5300
52.0381
49.5075
49.3360
49.1653
48.9961
48.8242
48.6559
48.4841
SP_F
kompasinol A
(maackolin)
OMe
2,6
β
3,5
7′
6′
5′
2′
14′
12′
10′
9′
1′
13′
11′
3′
4′
1
4
γ
8′
α
x
160
140
120
100
80
60
40
20 [ppm]

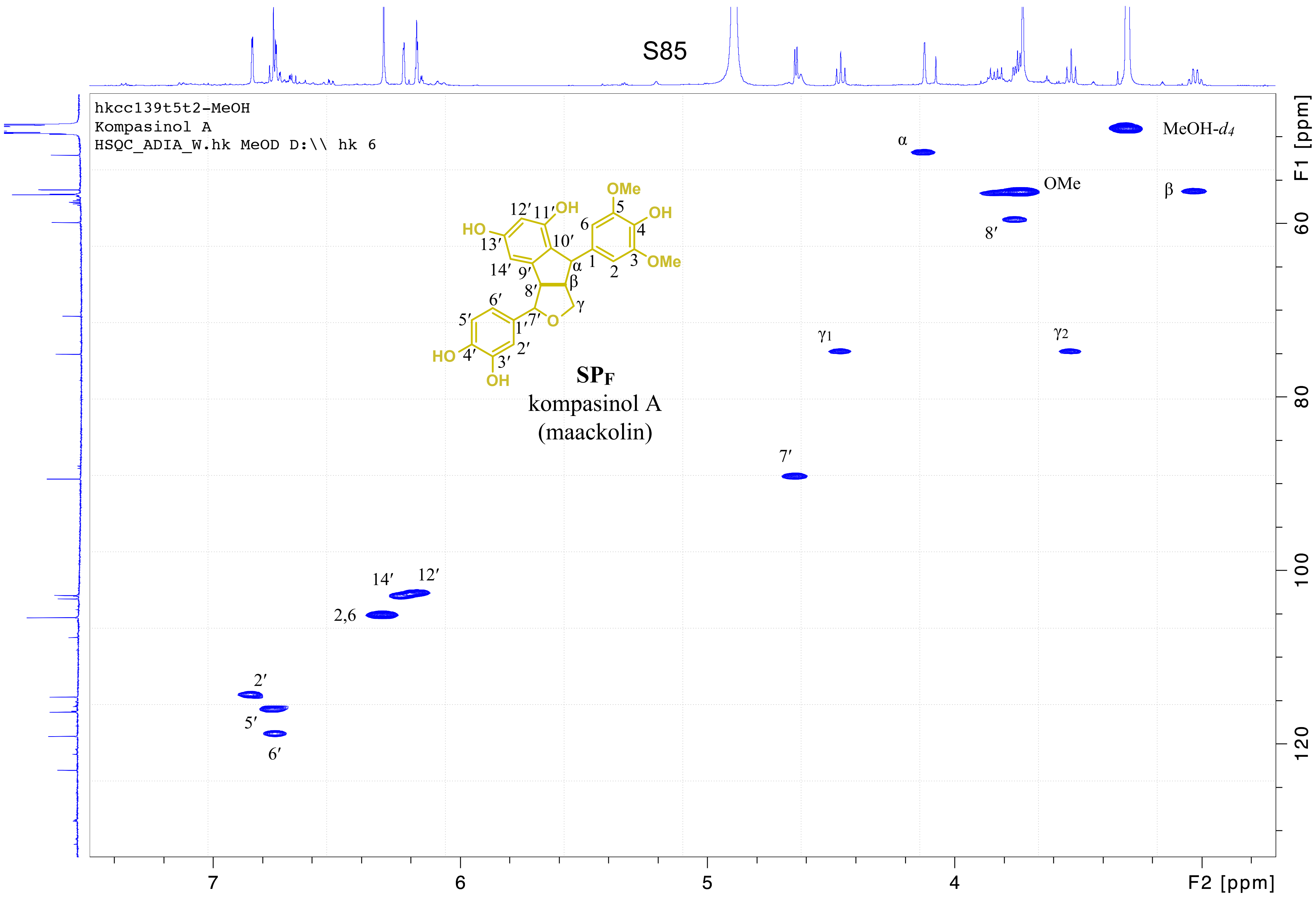
hkcc139t5t2-MeOH
Kompasinol A
HSQC_ADIA_W.hk MeOD D:\\ hk 6
SPF
kompasinol A
(maackolin)
MeOH-d4
α
OMe
β
8′
γ1
γ2
7′
14′
12′
2,6
2′
5′
6′
F1 [ppm]
F2 [ppm]
60
80
100
120
7
6
5
4

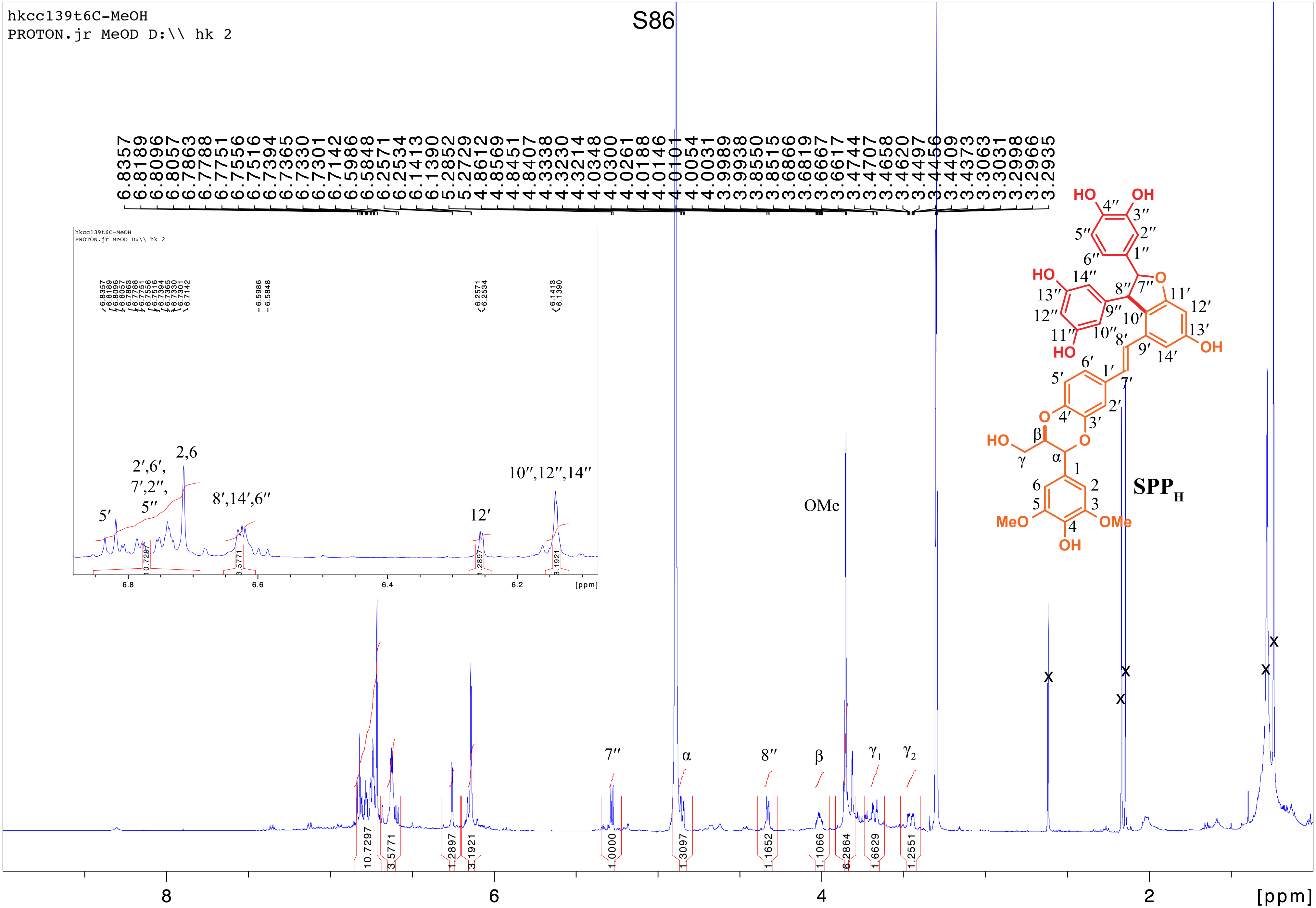
hkcc139t6C-MeOH
PROTON.jr MeOD D:\\ hk 2
SPP$_H$
OMe
7″
α
8″
β
γ1
γ2
5′
2′,6′, 7′,2″, 5″
2,6
8′,14′,6″
12′
10″,12″,14″
10.7297
3.5771
1.2897
3.1921
1.0000
1.3097
1.1652
1.1066
6.2864
1.6629
1.2551
[ppm]

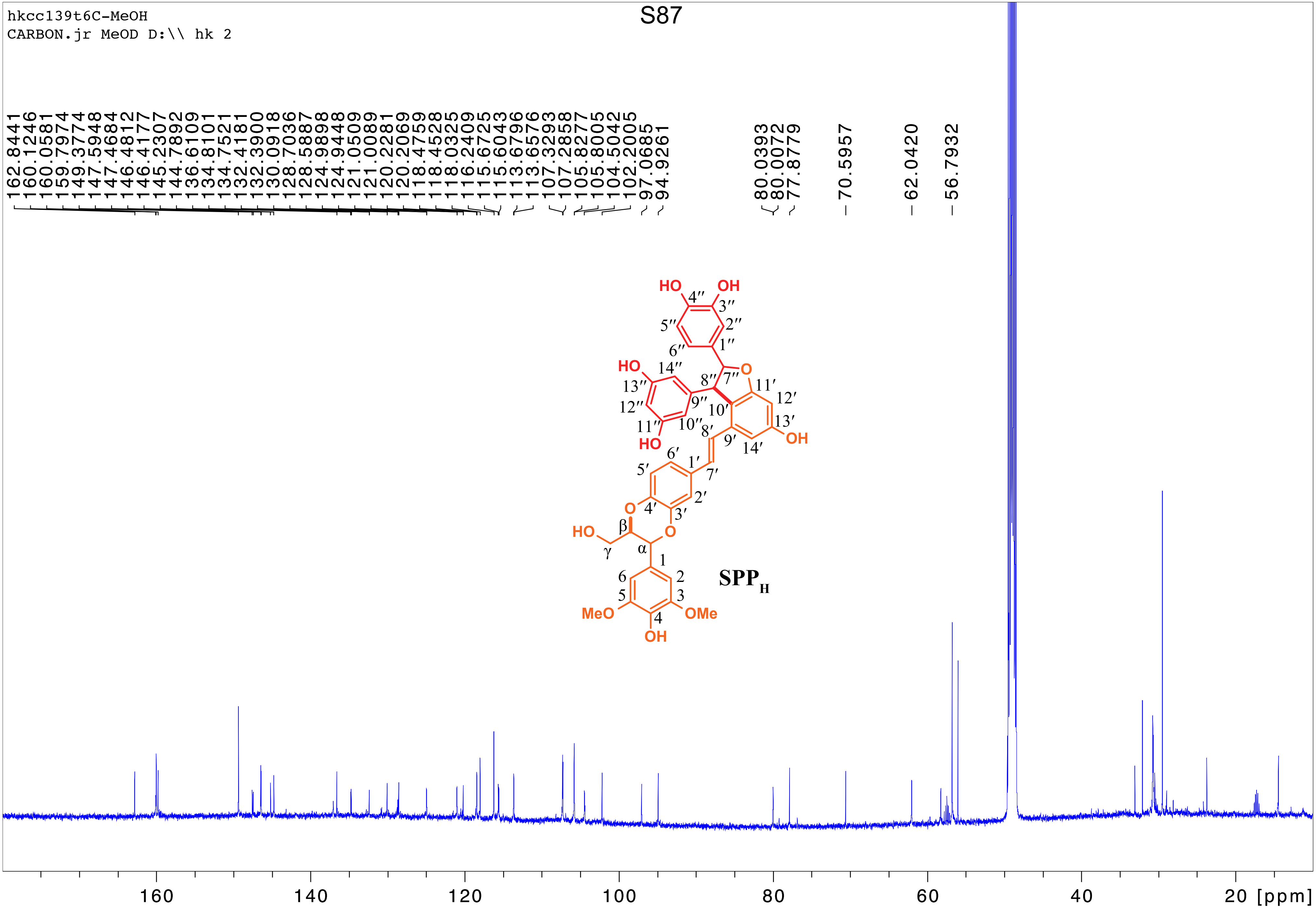

hkcc139t6C-MeOH
CARBON.jr MeOD D:\\ hk 2
162.8441
160.1246
160.0581
159.7974
149.3774
147.5948
147.4684
146.4812
146.4177
145.2307
144.7892
136.6109
134.8101
134.7521
132.4181
132.3900
130.0918
128.7036
128.5887
124.9898
124.9448
121.0509
121.0089
120.2281
120.2069
118.4759
118.4528
118.0325
116.2409
115.6725
115.6043
113.6796
113.6576
107.3293
107.2858
105.8277
105.8005
104.5042
102.2005
97.0685
94.9261
80.0393
80.0072
77.8779
70.5957
62.0420
56.7932
SPP$_H$
160
140
120
100
80
60
40
20 [ppm]

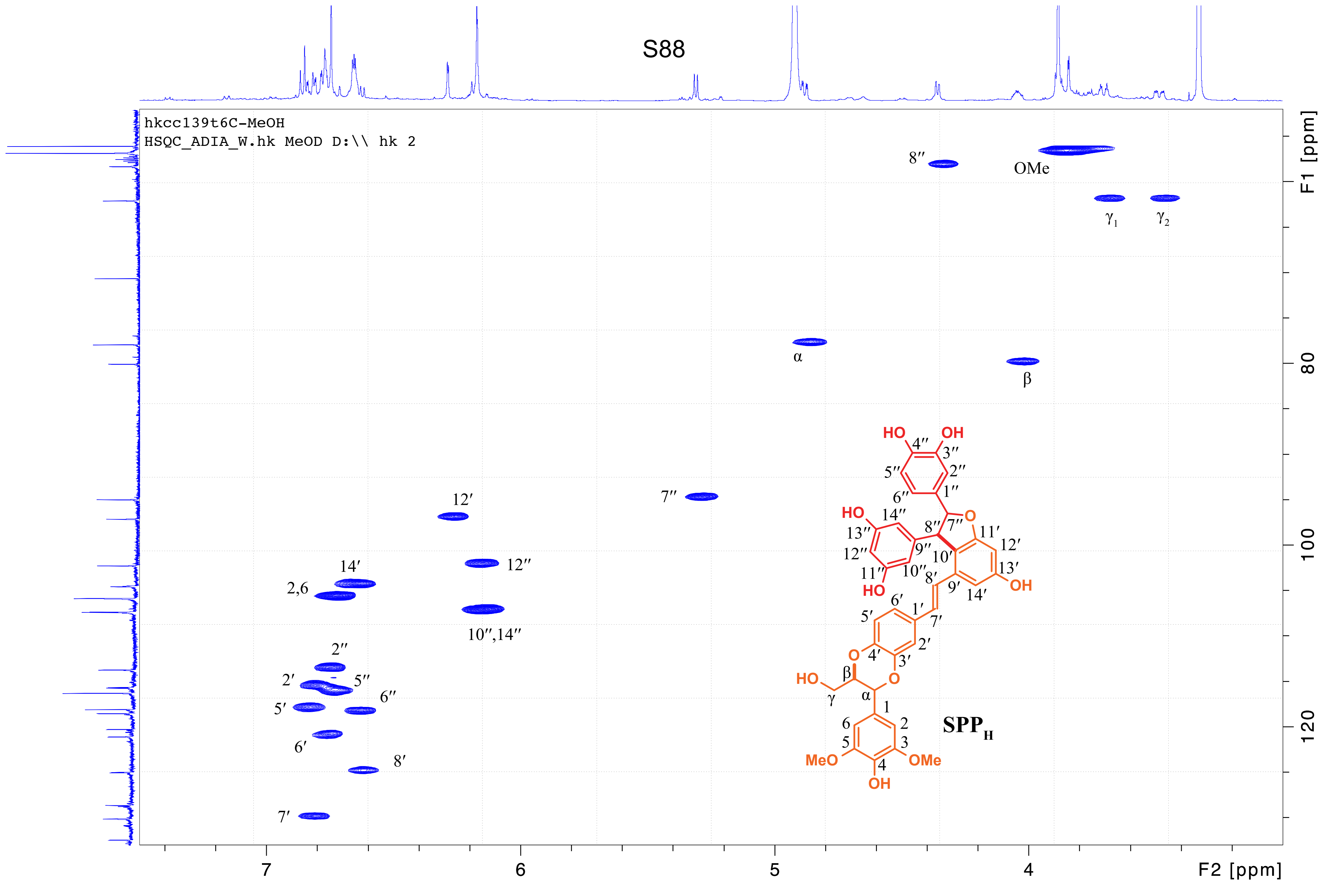

hkcc139t6C-MeOH
HSQC_ADIA_W.hk MeOD D:\\ hk 2
8″
OMe
γ1
γ2
α
β
7″
12′
12″
14′
2,6
10″,14″
2″
2′
5″
6″
5′
6′
8′
7′
SPPH
F1 [ppm]
F2 [ppm]

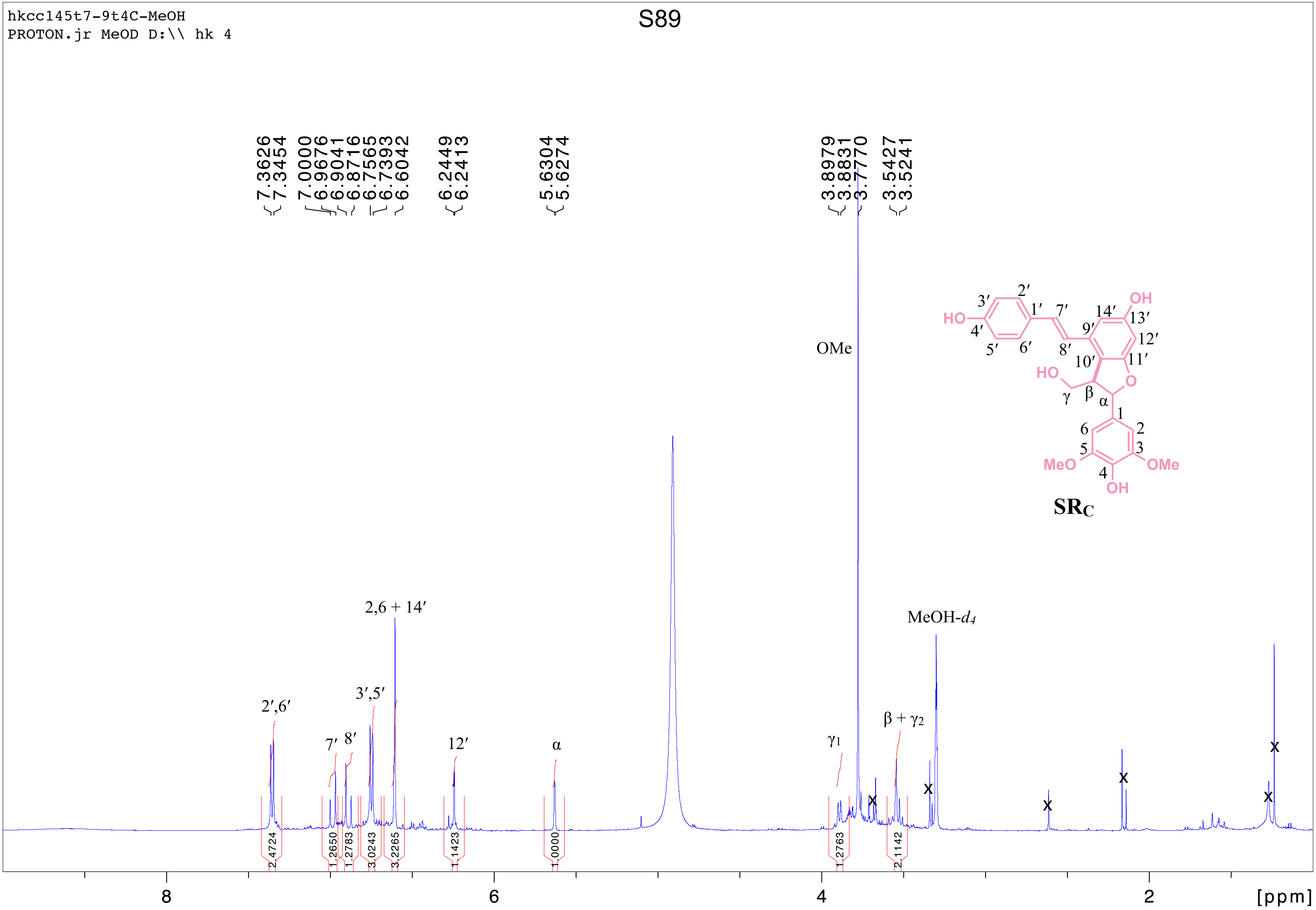
hkcc145t7-9t4C-MeOH
PROTON.jr MeOD D:\\ hk 4
7.3626
7.3454
7.0000
6.9676
6.9041
6.8716
6.7565
6.7393
6.6042
6.2449
6.2413
5.6304
5.6274
3.8979
3.8831
3.7770
3.5427
3.5241
OMe
MeOH-d4
2,6 + 14′
3′,5′
2′,6′
7′
8′
12′
α
γ1
β + γ2
x
HO
OH
O
MeO
OMe
SRC
2.4724
1.2650
1.2783
3.0243
3.2265
1.1423
1.0000
1.2763
2.1142
8
6
4
2
[ppm]

S90

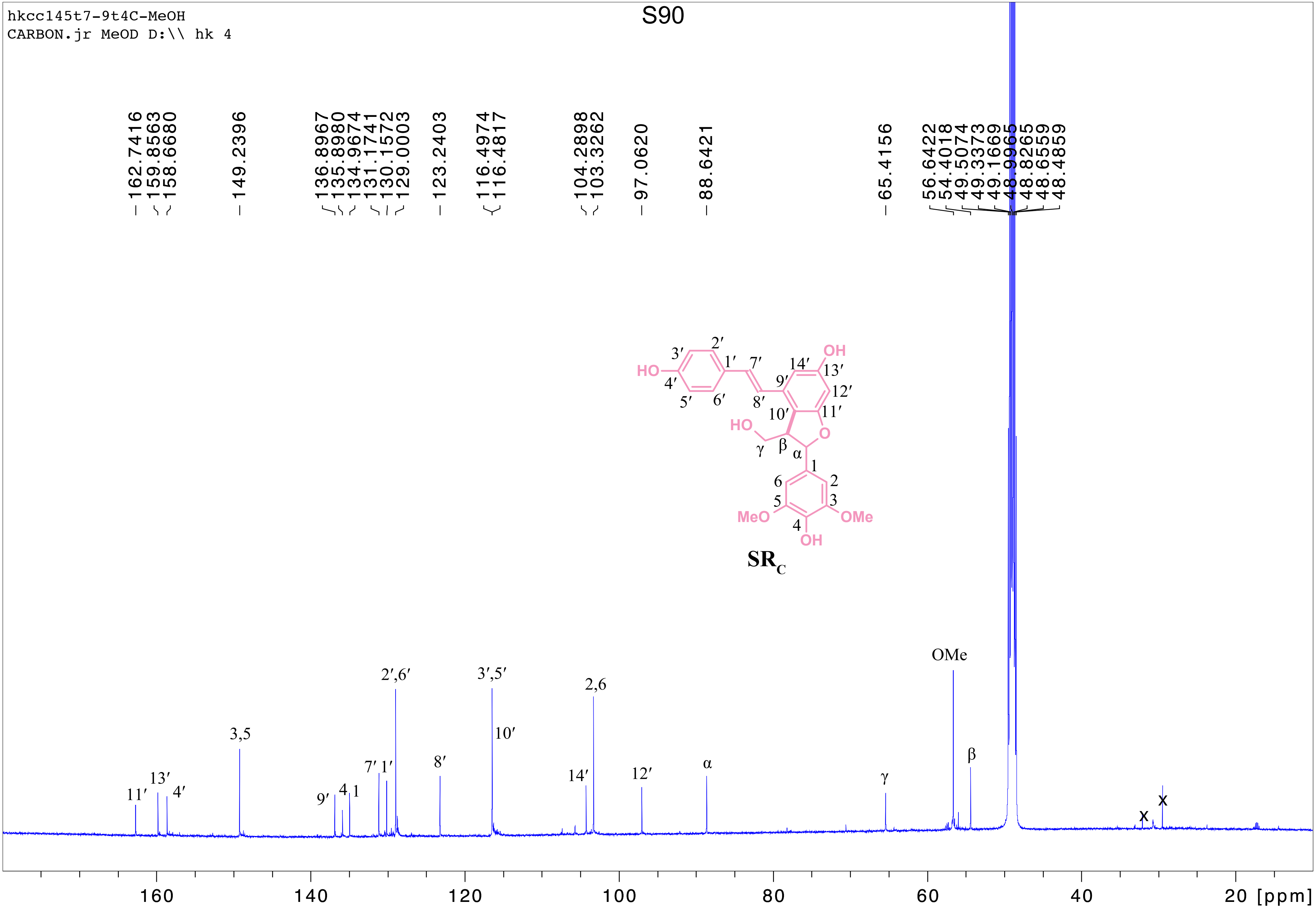
hkcc145t7-9t4C-MeOH
CARBON.jr MeOD D:\\ hk 4
162.7416
159.8563
158.6680
149.2396
136.8967
135.8980
134.9674
131.1741
130.1572
129.0003
123.2403
116.4974
116.4817
104.2898
103.3262
97.0620
88.6421
65.4156
56.6422
54.4018
49.5074
49.3373
49.1669
48.9965
48.8265
48.6559
48.4859
SR$_C$
11′
13′
4′
3,5
9′
4
1
7′
1′
2′,6′
8′
3′,5′
10′
14′
2,6
12′
α
γ
OMe
β
x
x
160
140
120
100
80
60
40
20 [ppm]

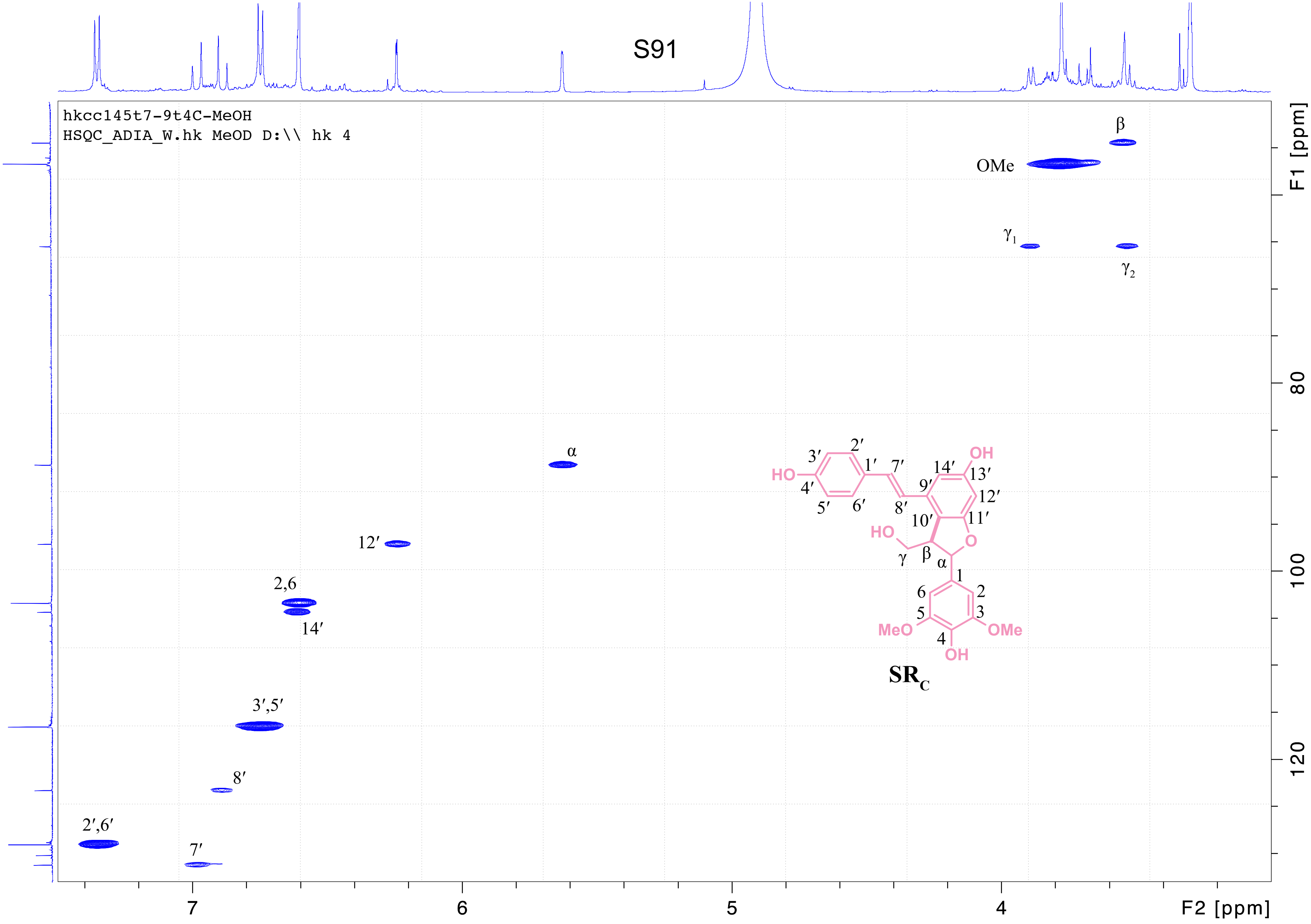
S91
hkcc145t7-9t4C-MeOH
HSQC_ADIA_W.hk MeOD D:\\ hk 4
β
OMe
γ1
γ2
α
12′
2,6
14′
3′,5′
8′
2′,6′
7′
SRC
F1 [ppm]
F2 [ppm]

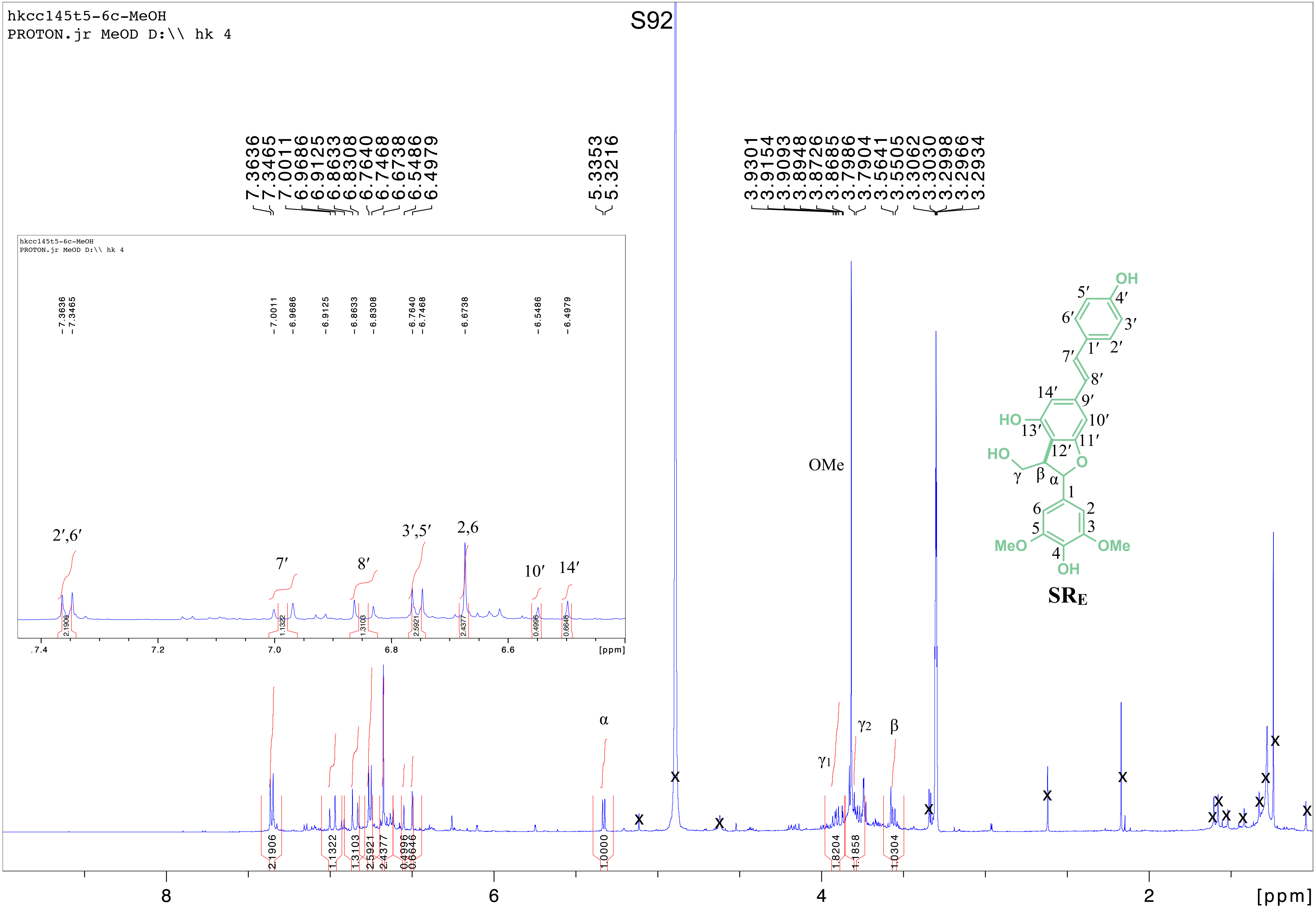

hkcc145t5-6c-MeOH
PROTON.jr MeOD D:\\ hk 4
7.3636
7.3465
7.0011
6.9686
6.9125
6.8633
6.8308
6.7640
6.7468
6.6738
6.5486
6.4979
5.3353
5.3216
3.9301
3.9154
3.9093
3.8948
3.8726
3.8685
3.7986
3.7904
3.5641
3.5505
3.3062
3.3030
3.2998
3.2966
3.2934
2′,6′
7′
8′
3′,5′
2,6
10′
14′
2.1906
1.1322
1.3103
2.5921
2.4377
0.4996
0.6646
α
OMe
γ1
γ2
β
1.0000
1.8204
1.1858
1.0304
OH
HO
HO
MeO
OMe
OH
SRE
[ppm]

hkcc145t5-6c-MeOH
CARBON.jr MeOD D:\\ hk 4

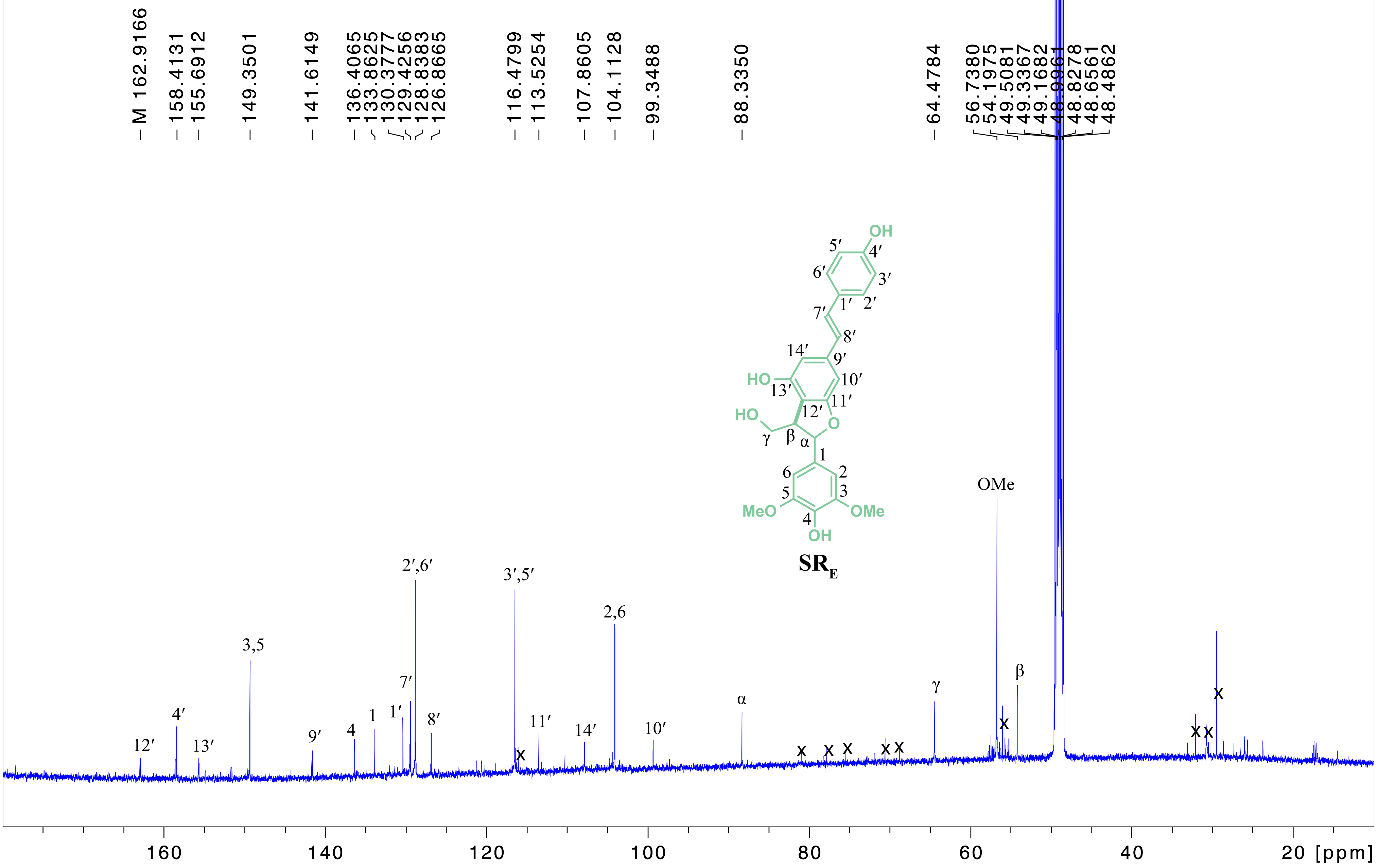

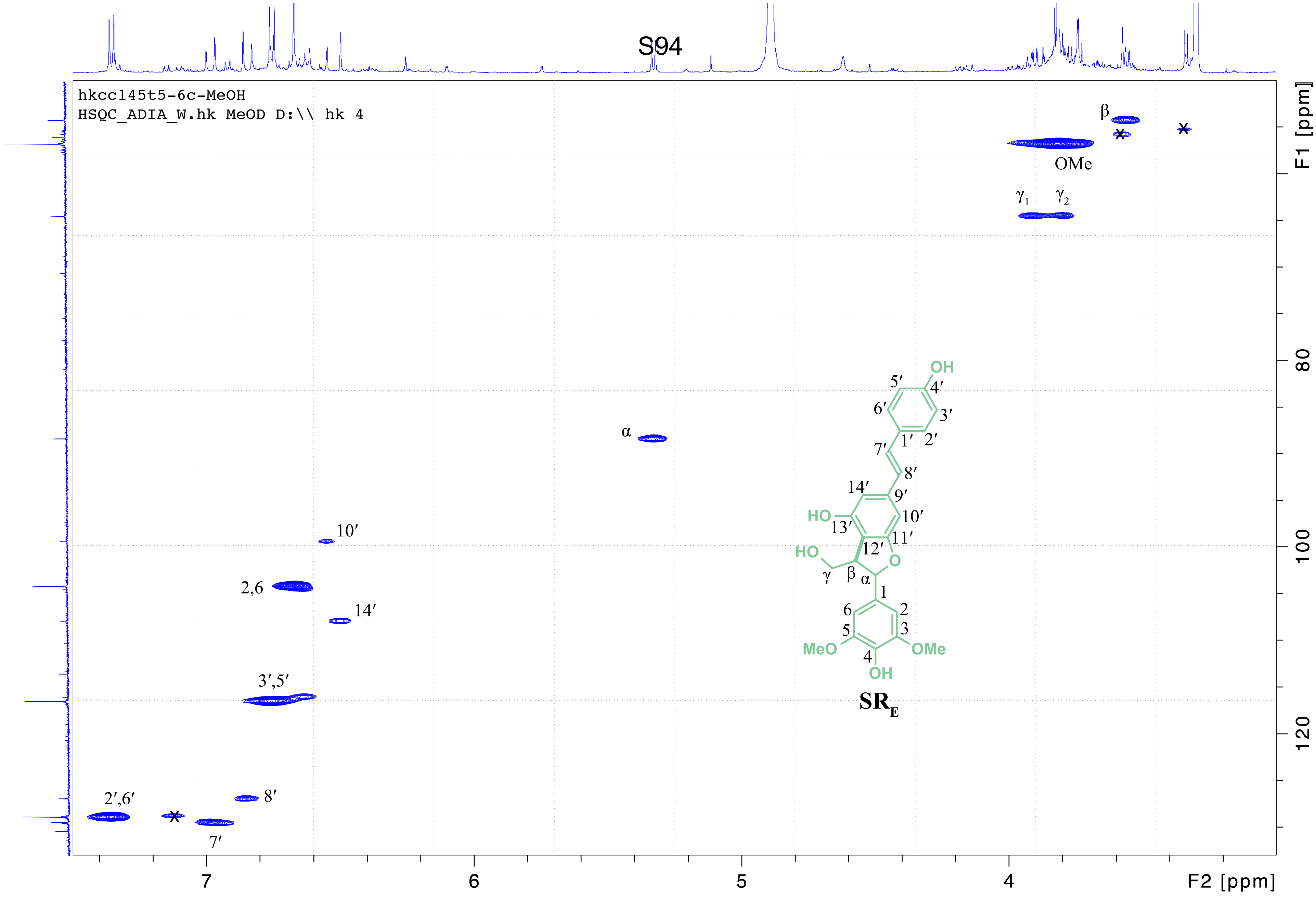
hkcc145t5-6c-MeOH
HSQC_ADIA_W.hk MeOD D:\\ hk 4
β
OMe
γ1 γ2
α
10′
2,6
14′
3′,5′
2′,6′
8′
7′
OH
4′
5′
6′
3′
1′
2′
7′
8′
14′
9′
HO
13′
10′
11′
12′
HO
O
γ β α
1
6
2
5
3
MeO
OMe
4
OH
SR_E
F1 [ppm]
F2 [ppm]
